# Supplementary material for: Additions to the genus Anthinus occurring in Minas Gerais and Goiás regions, Brazil, with description of five new species, one of them in the new related genus Catracca (Gastropoda, Eupulmonata, Strophocheilidae)
Source: PLoS One. 2022 Aug 24;17(8):e0273067. doi: 10.1371/journal.pone.0273067 (PMC9401170; doi:10.1371/journal.pone.0273067)
Supplement: Attachment — Submitted filename: 2022 Anthinus Answer to Editor.pdf [file pone.0273067.s001.pdf]

Response to Reviewers

The present document has the answers IN RED to the comments of the three referees, related to the second round of analysis of my paper now titled: **"Additions to the genus *Anthinus* occurring in Minas Gerais and Goiás regions, Brazil, with description of five new species, one of them in the new related genus *Catracca* (Gastropoda, Eupulmonata, Strophocheilidae)."**

The Reviewer #2 was very positive and understanding, approving the paper. The other two Reviewers (#1 and #3), on the other hand, were less friendly, rejecting the paper. However, I think both referees did not completely understand my answers, and that those were requests, and not intransigent denials. Anyway, I answer them below. I did not include the first round of criticisms/answers (PONE-D-21-27031, email 11/25/2021). If it is required, I will add them as soon as I am informed.

It is important to emphasize that the new version practically attended all three reviewers' requirements, looking like worthy for publication.

6. Review Comments to the Author

Reviewer #1: It pains me to see that the author has made only minor cosmetic changes to the Introduction and the Discussion and did not address any of the major problems raised by myself and the other reviewers, like the creation of a pointless genus-level taxon and the complete lack of a phylogenetic structure and molecular data. In almost all cases, the author just stated "that is what I have always done, therefore it is right" or "this is what has always been done in malacology, therefore it is right", without addressing any of the issues. While it is true that some practices in science and in taxonomy in particular date back to the 18th century, they are not necessarily correct or even acceptable in our present 21st century community. For instance, the author commented that a DNA analysis can only be performed after the species has been named, which is preposterous. The DNA analysis is an immediate manner of uncovering information to support or refute the hypothesis of whether the purported OTUs represent new a species or not. The inclusion of a molecular analysis alone could save this manuscript. In addition, the author also did not fully address the ethical and competing interests in naming the species by presenting a necessary disclaimer. The author did not correct any of the figures, keeping his images without scale, which makes proper comparison of the shells very difficult.

The new version, presently submitted, attends all this referee's requirements. The single one that does not meet them is the introduction of molecular analysis. Despite the molecular analysis undoubtedly being an additional data, the already presented conchological, morphological, anatomical, histological and biogeographic approach are convincing enough for basing all the conclusions.

Example: the new version includes a phylogenetic analysis to attend this referee. Although it can be considered provisional, as there are not too many strophocheilid species anatomically known in detail, the analysis clearly additionally supports the

conclusions and the description of the new genus. This can be checked in the submitted new version.

I really sorry that this referee did not consider all my justifications adequate. Anyway, I am afraid he/she did not understand them completely. This is a conclusion I realized by some of the above mentioned assertives, which were not what I stated. An example is "that is what I have always done, therefore it is right" or "this is what has always been done in malacology, therefore it is right". This no way is my point! That I said is, with more than 300 published papers in international journals (Plos included) and ~3,000 anatomic descriptions (check my CV website), I already have a usual, orthodox model which I follow in my papers. This orthodoxy facilitates any interested reader to find what he/she is interested in. At no point did I stated “therefore I am right”. This is a dogmatic thought and even an offence, which I securely do not apply as justification. Another example of my answer’s misunderstanding is: “DNA analysis can only be performed after the species has been named”. I never said that, I only stated that any complementary analysis (molecular included) should be more intelligible if all taxa are already well-established taxonomically. That “only be” are not my words.

The plates of shells usually do not bear scale bars. The direct length (L) is put in the legend. This bakes the plates clean and esthetically more presentable, without losing information. All partial views and anatomical images do have scale bars.

Anyway, and despite these misunderstandings, I think the new version attends the immense majority of this referee’s requirements.

Reviewer #2: The revised manuscript is adequate, but still could be improved. That the author has published over 300 papers is acknowledged, but there is always opportunity to improve. Seems the author has not fully taken on the suggestions (that is all they are, suggestions) from the reviewers. I am happy to recommend acceptance, but there are two points that need to be addressed:

This referee was much more understanding. Even though I always try to follow suggestions, they are suggestions as the referee said, consequently non-obligatory. If well-justified, some assertions can be not or only partially accepted.

1. No information is given on the specimens examined for the anatomical work. The ‘material examined’ listed for each species mentions only shell material. Were the specimen(s) examined anatomically for Catracca uhlei, for example, from the type locality or from elsewhere? – this is an important distinction.

I double-checked this and the new version has this information clear. In Material Examined, “shell” is only shell, thus conchologically analyzed. “Specimen” is an entire individual, shell plus soft parts, thus worthy both for conchology and anatomy. All “specimens” were anatomically investigated.

2. Discussion. ‘Specific debate’
The reviewers suggested some alternative formats for presentation of the text relating to shell dimension data – e.g. tabulation or ordination biplots. The author has not accepted

the recommendations and retained the rather laborious text, at great loss of clarity. I think the author should reconsider.

This new version has its Discussion divided into topics. I think this is adequate. However, if the referee or the editors suggest more subdivisions or another "less laborious" model, I will be happy in modifying that part.

There are a number of editorial corrections to be made through out the text - spelling, etc. In Acknowledgements, Barker not Narker

Okay, everything was double-checked. The text was corrected by two different language softwares, and additionally was corrected by a native English speaker Mr. Lee Kremer.

Reviewer #3: The author got a lot of constructive suggestions how to improve his manuscript from three reviewers. He rejected almost all of them.
If he considers "proper measurements" "not necessary in the present context" (!), if he is not able to summarize the differences between his putative species in a single sentence or an easy to read table but hides them in his terribly lengthy text and if he makes higher taxa on a merely phenetic basis without even considering to think about phylogeny, the paper has to be rejected. This is the worst "revision" I have ever seen.

The new version, presently submitted, attends all this referee's requirements. I am sorry this referee did not like my previous revision. Possibly he/she did not read my explanations completely (most were requests, not intransigent rejections) and did not check that I did not "rejected almost all of them", on the contrary, a majority was accepted. It is important to emphasize that "a merely phenetic basis" is ~99% of Mollusca taxonomy, and the base of my career. I wonder if no dataset is more trustable than others in Biology. This does not sound like a good argumentation. Anyway, to attend to this criticism, the new submitted version has a phylogenetic analysis. It is an additional support for the conclusions.

The distinctions amongst the studied species (new or not) are exposed in a taxon viewpoint – the Diagnoses; in a comparative viewpoint – the Distinctive Descriptions; and in a debatable context – the Discussion. What else must I do to "summarize the differences between his putative species"? A table? It is now present, along with the Phylogenetic analysis.
