# Supplementary material for: Additions to the genus Anthinus occurring in Minas Gerais and Goiás regions, Brazil, with description of five new species, one of them in the new related genus Catracca (Gastropoda, Eupulmonata, Strophocheilidae)
Source: PLoS One. 2022 Aug 24;17(8):e0273067. doi: 10.1371/journal.pone.0273067 (PMC9401170; doi:10.1371/journal.pone.0273067)
Supplement: Attachment — Submitted filename: PONE-D-22-00958.pdf [file pone.0273067.s002.pdf]

PLOS ONE

# Additions to the genus Anthinus occurring in Minas Gerais and Goiás regions, Brazil, with description of five new species, one of them in the new related genus Catracca (Gastropoda, Eupulmonata, Strophocheilidae).

--Manuscript Draft--

| | |
|---|---|
| **Manuscript Number:** | PONE-D-22-00958 |
| **Article Type:** | Research Article |
| **Full Title:** | Additions to the genus Anthinus occurring in Minas Gerais and Goiás regions, Brazil, with description of five new species, one of them in the new related genus Catracca (Gastropoda, Eupulmonata, Strophocheilidae). |
| **Short Title:** | Additions to Anthinus and new Catracca (Strophocheilidae) in Brazil |
| **Corresponding Author:** | Luiz Ricardo L. Simone, Dr.<br>Museu de Zoologia da Universidade de Sao Paulo<br>São Paulo, São Paulo BRAZIL |
| **Keywords:** | Mollusca; Pulmonata; Morphology; Anatomy; Taxonomy; Systematics; Brazil; biogeography; Phylogeny; evolution |
| **Abstract:** | Four new species of the strophocheilid genus Anthinus are described from Brazil, being A. synchondrus from region of Unaí, Minas Gerais; A. vailanti from the region of Brasilândia de Minas, Minas Gerais; A. morenus from Paracatu region, Minas Gerais; and A. savanicus from Formosa region, Goiás. Another similar snail from Itacarambi, Minas Gerais, is described as a new genus and species: Catracca uhlei . All species are described conchologically and anatomically, with distinctions explored in almost all structures. For comparative purposes, a similar anatomical investigation on the Anthinus type species, A. multicolor , from Rio de Janeiro, is also performed. A detailed comparative analysis and discussion is provided, including taxonomical and initial phylogenetic inferences. The preliminary phylogenetic analysis is based on the more detailly anatomically known species in the literature and in the presently study. A bulimulid, a simpulopsid and two dorcasiids are outgroups. The preliminary cladogram already shows a monophyletic Strophocheilidae (14 synapomorphies), divided into two also monophyletic subfamilies: Megalobuliminae (14 synapomorphies) and Strophocheilinae (5 synapomorphies). The new Catracca resulted as the most basal strophocheilid branch. Mirinaba and Anthinus also resulted monophyletic (3 and 6 synapomorphies respectively. Register ZooBank: urn:lsid:zoobank.org:pub:FC4DD323-EF6A-404B-9755-F124F9DBB6D4 |
| **Order of Authors:** | Luiz Ricardo L. Simone, Dr. |
| **Additional Information:** | |
| **Question** | **Response** |
| **Financial Disclosure**<br><br>Enter a financial disclosure statement that describes the sources of funding for the work included in this submission. Review the submission guidelines for detailed requirements. View published research articles from *PLOS ONE* for specific examples.<br><br>This statement is required for submission and **will appear in the published article** if the submission is accepted. Please make sure it is accurate. | No |

*Powered by Editorial Manager® and ProduXion Manager® from Aries Systems Corporation*

| | |
|---|---|
| **Unfunded studies**<br>Enter: *The author(s) received no specific funding for this work.*<br><br>**Funded studies**<br>Enter a statement with the following details:<br>• Initials of the authors who received each award<br>• Grant numbers awarded to each author<br>• The full name of each funder<br>• URL of each funder website<br>• Did the sponsors or funders play any role in the study design, data collection and analysis, decision to publish, or preparation of the manuscript?<br>• **NO** - Include this sentence at the end of your statement: *The funders had no role in study design, data collection and analysis, decision to publish, or preparation of the manuscript.*<br>• **YES** - Specify the role(s) played.<br><br>* typeset | |
| **Competing Interests**<br><br>Use the instructions below to enter a competing interest statement for this submission. On behalf of all authors, disclose any competing interests that could be perceived to bias this work—acknowledging all financial support and any other relevant financial or non-financial competing interests.<br><br>This statement is required for submission and **will appear in the published article** if the submission is accepted. Please make sure it is accurate and that any funding sources listed in your Funding Information later in the submission form are also declared in your Financial Disclosure statement.<br><br>View published research articles from *PLOS ONE* for specific examples. | NO authors have competing interests |

*Powered by Editorial Manager® and ProduXion Manager® from Aries Systems Corporation*

| | |
|---|---|
| **NO authors have competing interests**<br><br>Enter: *The authors have declared that no competing interests exist.*<br><br>**Authors with competing interests**<br><br>Enter competing interest details beginning with this statement:<br><br>*I have read the journal's policy and the authors of this manuscript have the following competing interests: [insert competing interests here]*<br><br>* typeset | |
| **Ethics Statement**<br><br>Enter an ethics statement for this submission. This statement is required if the study involved:<br><br>• Human participants<br>• Human specimens or tissue<br>• Vertebrate animals or cephalopods<br>• Vertebrate embryos or tissues<br>• Field research<br><br>Write "N/A" if the submission does not require an ethics statement.<br><br>General guidance is provided below. Consult the submission guidelines for detailed instructions. **Make sure that all information entered here is included in the Methods section of the manuscript.** | Not applicable |

Powered by Editorial Manager® and ProduXion Manager® from Aries Systems Corporation

| | |
|---|---|
| **Format for specific study types**<br><br>**Human Subject Research (involving human participants and/or tissue)**<br>• Give the name of the institutional review board or ethics committee that approved the study<br>• Include the approval number and/or a statement indicating approval of this research<br>• Indicate the form of consent obtained (written/oral) or the reason that consent was not obtained (e.g. the data were analyzed anonymously)<br><br>**Animal Research (involving vertebrate animals, embryos or tissues)**<br>• Provide the name of the Institutional Animal Care and Use Committee (IACUC) or other relevant ethics board that reviewed the study protocol, and indicate whether they approved this research or granted a formal waiver of ethical approval<br>• Include an approval number if one was obtained<br>• If the study involved *non-human primates*, add *additional details* about animal welfare and steps taken to ameliorate suffering<br>• If anesthesia, euthanasia, or any kind of animal sacrifice is part of the study, include briefly which substances and/or methods were applied<br><br>**Field Research**<br><br>Include the following details if this study involves the collection of plant, animal, or other materials from a natural setting:<br>• Field permit number<br>• Name of the institution or relevant body that granted permission | |
| **Data Availability**<br><br>Authors are required to make all data underlying the findings described fully available, without restriction, and from the time of publication. PLOS allows rare exceptions to address legal and ethical concerns. See the PLOS Data Policy and FAQ for detailed information. | Yes - all data are fully available without restriction |

Powered by Editorial Manager® and ProduXion Manager® from Aries Systems Corporation

| | |
|---|---|
| A Data Availability Statement describing where the data can be found is required at submission. Your answers to this question constitute the Data Availability Statement and **will be published in the article**, if accepted.<br><br>**Important:** Stating 'data available on request from the author' is not sufficient. If your data are only available upon request, select 'No' for the first question and explain your exceptional situation in the text box.<br><br>Do the authors confirm that all data underlying the findings described in their manuscript are fully available without restriction? | |
| **Describe where the data may be found in full sentences. If you are copying our sample text, replace any instances of XXX with the appropriate details.**<br><br>• If the data are **held or will be held in a public repository**, include URLs, accession numbers or DOIs. If this information will only be available after acceptance, indicate this by ticking the box below. For example: *All XXX files are available from the XXX database (accession number(s) XXX, XXX.).*<br>• If the data are all contained **within the manuscript and/or Supporting Information files**, enter the following: *All relevant data are within the manuscript and its Supporting Information files.*<br>• If neither of these applies but you are able to provide **details of access elsewhere**, with or without limitations, please do so. For example:<br><br>*Data cannot be shared publicly because of [XXX]. Data are available from the XXX Institutional Data Access / Ethics Committee (contact via XXX) for researchers who meet the criteria for access to confidential data.*<br><br>*The data underlying the results presented in the study are available from (include the name of the third party* | All relevant data are within the manuscript and its Supporting Information files. |

| | |
|---|---|
| *and contact information or URL).*<br>• This text is appropriate if the data are owned by a third party and authors do not have permission to share the data.<br><br>* typeset | |
| Additional data availability information: | |

Manuscript

Click here to access/download;Manuscript;2022 Anthinus Coltro Plos clean.docx

# Additions to the genus *Anthinus* occurring in Minas Gerais and Goiás regions, Brazil, with description of five new species, one of them in the new related genus *Catracca* (Gastropoda, Eupulmonata, Strophocheilidae).

**Luiz Ricardo L. Simone**

Museu de Zoologia da Universidade de São Paulo, Cx. Postal 42391; 04218-970 São Paulo, SP, Brazil; lrsimone@usp.br; lrlsimone@gmail.com; OrcID: 0000-0002-1397-9823

## Abstract

Four new species of the strophocheilid genus *Anthinus* are described from Brazil, being *A. synchondrus* from region of Unaí, Minas Gerais; *A. vailanti* from the region of Brasilândia de Minas, Minas Gerais; *A. morenus* from Paracatu region, Minas Gerais; and *A. savanicus* from Formosa region, Goiás. Another similar snail from Itacarambi, Minas Gerais, is described as a new genus and species: *Catracca uhlei*. All species are described conchologically and anatomically, with distinctions explored in almost all structures. For comparative purposes, a similar anatomical investigation on the *Anthinus* type species, *A. multicolor*, from Rio de Janeiro, is also performed. A detailed comparative analysis and discussion is provided, including taxonomical and initial phylogenetic inferences. The preliminary phylogenetic analysis is based on the more detailly anatomically known species in the literature and in the presently study. A bulimulid, a simpulopsid and two dorcasiids are outgroups. The preliminary cladogram already shows a monophyletic Strophocheilidae (14 synapomorphies), divided into two also monophyletic subfamilies: Megalobuliminae (14 synapomorphies) and Strophocheilinae (5 synapomorphies). The new *Catracca* resulted as the most basal strophocheilid branch. *Mirinaba* and *Anthinus* also resulted monophyletic (3 and 6 synapomorphies respectively. Register ZooBank: urn:lsid:zoobank.org:pub:FC4DD323-EF6A-404B-9755-F124F9DBB6D4

## Introduction

The Strophocheilidae genus *Anthinus* Albers, 1850 ([1] pp:148) has as the type species *Helix multicolor* Rang, 1831 [2] (Subsequent Designation by Martens [3]: 189-190). In an early monographic treatment of Strophocheilidae, Bequaert ([4]: 196-202), provided detailed taxonomic history of the genus. Anthinus characteristically includes the smallest strophocheilids, with adult specimens with shells around 20-30 mm, usually having a tall, bluntly pointed spire, relatively small aperture in a "bulimoid-shape", and a mosaic of brown-beige spots [4, 5]. All four valid *Anthinus* species are reported previously reviewed and illustrated by Simone ([6]: 201-202). Nonetheless, the published anatomical information on *Anthinus* has remained poor, restricted to sparse information and few illustrations [5, 7]. The geographic occurrence of *Anthinus* in the southeastern Brazilian Atlantic Rainforest environment, endorsed by Simone ([6]) was from the south region of Minas Gerais-Mato Grosso do Sul in Brazil (latitude ~15°S) to northern Argentina and Uruguay (latitude ~32°S). A doubtful occurrence in Tobago has been reported ([6] and references therein; [8]).

In recent years, some samples identified as *Anthinus* have been collected in expeditions by the naturalist José Coltro Jr. and colleagues, in regions from north of Minas Gerais, to Goiás (Brazilian midwest) and in several points in northeast Brazil (latitudes ~15°S to ~17°S). This material is used in the present paper. These regions have a dryer environment, including a dryer version of the Atlantic Rainforest, Cerrado and Caatinga semi-arid biomes.

Examination of this material revealed five undescribed species, more than doubling the number of presently known *Anthinus* species. Anatomical information on the type species, *A. multicolor*, collected in Rio de Janeiro, is also included for improving the comparative scenario. The shell of one of the new species, initially appeared to belong to a unicolored *Anthinus*. It has, however, a greatly different set of anatomical features, making necessary the description of a new genus. The description of the type species is, the remaining descriptions and illustrations, to minimize redundancy, are mostly presented compared to it. The published anatomical information on *Anthinus* is scanty, restricted to some sparse information and illustrations [5, 8]; it is explored in the discussion.

The family Strophocheilidae has had an unstable taxonomic history [4, 5], but usually it has been considered endemic from South America, with relatives in Africa – the Dorcasiidae; and in Asia – the Acavidae; all grouped in Acavoidea (e.g., [9]), or sometimes in Strophocheiloidea (e.g., [10]). South American members have traditionally been assigned to two families: Strophocheilidae and Megalobulimidae [5]. More recently these two families have been treated as subfamilies in Strophocheilidae, and, together with Acavidae and Dorcasiidae, placed in Rhytidoidea, which has five other families [11, 12].

This paper has as main objective the formal introduction of the new strophocheilids taxa. The anatomical base of all of them will certainly be useful for future taxonomic revisions and for phylogenetic inferences. Despite these aspects are part of a large revisional project in such this study is inserted, an initial phylogenetic treatment is provided in this paper, based on the species with anatomy more detailly known in the literature and in the herein studied species. The main intention is not to present a "phylogeny of the Strophocheilidae", as many more species would be necessary, but to provide a better scenario for a discussion and the base for the new taxa.

## Material and methods

**Material.** A complete list of material examined follows each species' description. Material comprised of dry shells or shells and specimens preserved in 70% EtOH. All listed specimens were used to compose the descriptions; those that are only shells (sh) were examined at a conchological level; all complete specimens (shell and soft parts – spm) were extracted and dissected.

**Methods and scope.** The anatomical drawings are an average of all examined specimens. Color photos were obtained by digital cameras, either hand-held or attached to the dissecting microscope. Shell measurements were obtained with a digital caliper, in a minimum of 10 adult shells. The specimens were dissected by standard techniques [13] under dissecting stereomicroscopes, with the specimen immersed under the fixative. All drawings were obtained with the aid of a camera lucida; initially penciled, afterwards inked; usually drawings produced for each species include data derived from several specimens. The type and voucher material are deposited in Museu de Zoologia da Universidade de São Paulo (MZSP) malacological collection. The specimens usually are relatively easily extracted from their shells, except for the holotype of *Anthinus synchondrus*, in this case a 'cesarean' (a small window) needed to be done

in the last whorl. SEM work on the material is standard, with pieces metalized by gold, and examined in the Laboratory of Electron Microscopy of the MZSP. Anatomical terminology, particularly of the odontophore muscles, follows Simone [13]. The collector team works for a company called Femorale [www.femorale.com; http://www.femorale.com/femorale/index.asp]. The places of collection are not protected areas, which do not require special permits. The collections, anyway, are supported by the general/permanent license IBAMA-Sisbio 10560-2, which permits extraction of wildlife samples for scientific purposes. As most of the studied material was collected by non-scientific expeditions, no further data beyond coordinates and place names were available. Thus, details on vegetation, climate, soil, rainfall, etc., were not available, but, when relevant, these data were extracted from the literature or official websites. For comparison of the presently studied species with those already known, the large MZSP collection was consulted, as a previous curator, Dr. José L.M. Leme, was a strophocheilid specialist. Collections of other European and American museums were also consulted in my trips seeking type specimens, some of them illustrated in a catalogue [6]. However, despite high effort, types of not all *Anthinus* species were found. In these cases, voucher material of previous revisions was preferentially studied. Despite having material for a total review of the genus Anthinus available, the previous Bequaert's review [4] is so well done that nothing actually new could be informed at shell level. Despite being relatively antique, Bequaert's review [4] is still valid in species' level, and few additional information was published on *Anthinus* after that. Thus, the present paper focuses on the description of the new taxa and the anatomical inedited features. The type species, however, is included, extending to anatomical features and improving the comparative taxonomic scenario. ~~Although additional approaches can be added to any taxonomical paper, such as multivariate statistical analyses of shell measurements, molecular sequencing, etc., the presently presented anatomical investigation was so informative and convincing that it already bases very well the taxonomic conclusions. Of course, other approaches, molecular in particular, will be explored in future papers, considering an already well-based taxa provided by the present paper. It is important to emphasize that most of the land snails taxonomy is based only on their shells alone; the present paper goes further and presents convincing anatomical descriptions.~~ The chosen model to do the taxonomic remarks on each described species is reuniting all of them in a single Discussion, which ends this paper. This is interpreted as a more intelligible, fluent, and economic manner to explore the distinctions and similarities of the addressed taxa. Rather than individual remarks after each description.

**Phylogenetic analysis.** In a family such as Strophocheilidae, that bears almost a hundred of known species, a phylogenetic study is only worthy if it includes a large number of representatives. This is presently not possible, as few species are known beyond their shells; and shells alone cannot ~~base~~ a well-resolved phylogeny [13]. Anyway, a small set of strophocheilids has their anatomy known in sufficient details for an initial phylogenetic inference. They are (1) *Mirinaba antoninensis* (Morretes, 1952) [14, 15]; (2) *Mi. cadeadensis* (Morretes, 1952) [15]; (3) *Strophocheilus debilis* Bequaert, 1948 [5]; (4) *Megalobulimus lopesi* Leme, 1984 [16]; (5) *Me. grandis* (Martens, 1885) [16]; (6) *Me. parafragilior* Leme & Indrusiak, 1990 [17]; (7) *Me. proclivis* (Martens, 1888) [18]; (8) *Me. dryades* Fontenelle, Simone & Cavallari, 2021 [19]; (9) *Me. oblongus* (Müller, 1774) and (10) *Me. conicus* (Bequaert, 1948) [20]. As remote outgroups, a bulimulid (*Leiostracus carnavalescus* Simone & Salvador, 2016 [21]) and a simpulopsid (*Rhinus botocudus* Simone & Salvador, 2016 [21]), and close outgroups a pair of African dorcasiids (*Dorcasia alexandri* Gray, 1838 and *Trigonephrus porphyrostoma* (Melvill & Ponsonby, 1891) [22]) were chosen. All six species studied herein were also included. The phylogenetic methodology is the same as reported by Simone [13, 23], that basically consists of the matrix mounted in Nexus, analyzed by programs TNT and PAUP. All of them resulted in a single cladogram shown below. Thus, beyond the species herein studied, the present preliminary phylogeny is based upon already published data of the above mentioned 14 species, as well as

additional examination of their voucher material deposited in MZSP in some unstudied structures, such as, e.g., the odontophore. The list of characters is in Appendix 1; and respective matrix in Appendix 2. It is important to emphasize that the shown phylogeny is not to be interpreted as “the phylogeny of the Strophocheilid”. It has only the intention of demonstrating that the description of the new genus – *Catracca* – is necessary.

13. **Nomenclatural acts.** The electronic edition of this article conforms to the requirements of the amended International Code of Zoological Nomenclature, and hence the new names contained herein are available under that Code from the electronic edition of this article. This published work and the nomenclatural acts it contains have been registered in ZooBank, the online registration system for the ICZN. The ZooBank LSIDs (Life Science Identifiers) can be resolved and the associated information viewed through any standard web browser by appending the LSID to the prefix "http://zoobank.org/". The LSID for this publication is: urn:lsid:zoobank.org:pub:FC4DD323-EF6A-404B-9755-F124F9DBB6D4. The electronic edition of this work was published in a journal with an ISSN, and has been archived and is available from the following digital repositories: PubMed Central, LOCKSS, ResearchGate.

**Abbreviations in the Figures**: **aa**, anterior aorta; **ac**, albumen chamber; **ad**, albumen gland duct; **af**, anal folded subterminal portion; **ag**, albumen gland; **an**, anus; **as**, accessory genital gland; **au**, auricle; **bc**, bursa copulatrix; **bd**, bursa copulatrix duct; **bg**, buccal ganglion; **bm**, buccal mass; **br**, subradular membrane; **bv**, blood vessel; **cc,** cerebral commissure; **ce**, cerebral ganglion; **cd**, cerebral node; **cn**, cerebro-pedal and cerebro-pleural connectives; **cv**, pulmonary (efferent) vein; **da**, digestive gland anterior lobe; **dc**, dorsal chamber of buccal mass; **dd**, gastric duct to digestive gland; **df**, dorsal folds of buccal mass; **dg**, digestive gland posterior lobe; **di**, diaphragm or pallial floor; **dl**, left esophageal duct to digestive gland; **dr**, right esophageal duct to digestive gland; **eh**, epiphallus; **eo**, spermoviduct; **es**, esophagus; **ey**, eye; **fo**, free oviduct; **fp**, genital pore; **ft**, foot; **gm**, genital muscle; **go**, gonad; **hd**, hermaphrodite duct; **hp**, hump of free oviduct; **in**, intestine; **jw**, jaw; **kf**, kidney anterior fold; **ki**, kidney; **lf**, lateral fold of buccal mass; **m1–m10**, extrinsic and intrinsic odontophore muscles; **mb**, mantle border (edge); **mf**, mantle fold; **mj**, jaw and peribuccal muscles; **mo**, mouth; **mp**, prerectal muscle; **ne**, nephrostome; **nr**, nerve ring; **oc**, odontophore cartilage; **od**, odontophore; **om**, ommatophore; **on**, optical nerve; **ou**, ommatophore muscle; **pa**, penis anterior chamber; **pc**, pericardium; **pe**, penis; **pf**, penis fold; **pg** pedal gland; **pm**, penis muscle; **pn**, pneumostome; **pp**, pedal ganglion; **pr**, penis aperture; **ps**, penis posterior chamber; **pt**, prostate; **pu**, pulmonary cavity; **pv**, pneumostome inner flap; **ra**, radula; **rn**, radular nucleus; **rs**, radular sac; **rt**, rectum; **sa**, salivary gland aperture; **sc**, subradular cartilage; **sd**, salivary gland duct; **se**, septum between esophagus and odontophore; **sf**, stomach inner fold; **sg**, salivary gland; **sh**, penis central sphincter; **sm**, secondary cephalic muscles; **sp**, sperm inner longitudinal fold; **sr**, seminal receptacle; **st**, stomach; **sw**, stomach muscular walls; **te**, ventral cephalic tentacle; **tg**, integument; **te**, tentacle; **tm**, tentacle muscle; **tn**, tentacular nerve; **un**, union of mantle border with nuchal surface; **ur**, urinary gutter; **ut**, uterus; **vd**, vas deferens; **ve**, ventricle; **vf**, vaginal fold; **vg**, vagina.

Additionally in the text, the following abbreviations are used: **L**, length; **sh**, empty dry shell; **spm**, complete specimen (shell and soft parts); **W**, width. Institutions: **MNRJ**: Museu Nacional da Universidade Federal do Rio de Janeiro; **MZSP**: Museu de Zoologia da Universidade de São Paulo; **USNM**: National Museum of Natural History, Smithsonian Institution.

## Results

### Comparative conchology and anatomy

## Systematics

### Genus *Anthinus* Albers, 1850

*Bulimus* (*Anthinus*) Albers, 1850[1]: 148; Martens, 1860[3]: 189.

*Auris* (*Anthinus*): Pilsbry, 1895[24]: 95.

*Gonyostomus* (*Anthinus*): Bequaert, 1948[4]: 196; Oliveira et al., 1968[8]: 10.

*Anthinus*: Morretes, 1949[25]: 145; Leme, 1973[5]: 332; Simone, 2006[6]: 201.

**Diagnosis.** Shell of ~30 mm, apex blunt, aperture ~half of shell length. Protoconch of 3-4 whorls, sculptured by delicate reticulation of spiral and axial lines. Color mosaic of brown, beige and cream wide spots, with tendency of spiral organization in squared irregular spots. Head-foot lacking labial flange. Lung with not-protruded vessels, dark pigmented. Odontophore pair m5 ~half originated from cartilages and ~half from m4, free from m2. Pair of anterior (esophageal) ducts to digestive gland, located posteriorly, practically from stomach; both components (dr, dl) with about same caliber, left duct much longer. Prerectal pallial muscle (mp) usually present. Anus turned externally in pneumostome right corner. Seminal receptacle strongly curved. Spermoviduct accessory gland (as) present. Penis with single inner chamber; epiphallus short, rounded, vas deferens insertion subterminal, inner surface papillate; inner main chamber with main pair of strong longitudinal folds, sulcus between these folds continuous to epiphallus. Other details in [4, 5].

**List of included species.** *Anthinus multicolor* (Rang, 1831) (type species); *A. albolabiatus* (Jaeckel, 1927); *A. miersi* (Sowerby, 1838); *A. turnix* (Gould, 1846); *A. synchondrus* new species; *A. vailanti* new species; *A. morenus* new species; *A. savanicus* new species.

**Taxonomic discussion.** see Discussion item.

### *Anthinus multicolor* (Rang, 1831) Figs. 1-4

Previous synonymy see Bequaert (1948[4]: 196-197). Complement:

*Helix* (*Cochlogena*) *multicolor* Rang, 1831[2]: 55 (pl. 3, fig. 1).

*Gonyostomus* (*Anthinus*) *multicolor:* Bequaert, 1948[4]: 196-198 (pl. 4, fig. 5; pl. 9, figs 3, 7; pl. 13, fig. 6; Pl. 17, fig. 4).

*Anthinus multicolor:* Morretes, 1949[25]: 145; Leme, 1973[5]: 332; Abbott, 1989[26]: 76 (fig.); Salgado & Coelho, 2003[27]: 156; Simone, 2006[6]: 202 (fig. 760).

*Gonyostomus multicolor:* Oliveira et al., 1968[8]: 10; 1981[28]: 341.

**Types.** whereabouts unknown [4].

**Type locality.** São Paulo State and near Corcovado, Rio de Janeiro.

**Diagnosis.** Shell about 35 mm; color usual mosaic of brown and beige spots, lacking spiral alignment; spire angle ~40°, protoconch of 2.5 whorls; peristome ~52% of shell length and ~55% of width; width ~46% of length. Mantle edge with simple, rounded fold. Reno-pericardial area small-sized. Pre-rectal pallial muscle absent. Odontophore with cartilages ~90% fused with each

other; pair m4 ~50% originated from cartilage, remaining on m4. Jaw plate broad, thick. Radula with short, hook-like mesocone, rachidial different from neighboring teeth. Seminal receptacle widely curved, hermaphrodite duct inserting in its base, curved. Accessory genital gland in spermoviduct small; with pair of similar-shaped sperm-grooves. Free oviduct with distal hump. Penis length ~50% of spermoviduct; penis muscle mostly inserted to penis' tip, with small branch to epiphallus and another small branch to adjacent vas deferens; epiphallus papillae tall, uneven, extending along entire penis chamber; large pair of penial inner folds wide and low, covered by papillae.

**Redescription**

**Shell.** (Figs. 1A-E) proper description in [4]. Complement: Adult shell around 35 mm, conical-oval; apex blunt; wider on last whorl; width ~46% shell length. Walls relatively thin. Color sometimes entirely brown ([6]: 202), but usually with reddish beige spiral band along inferior half of each spire whorl, or central region of body whorl (Figs. 1A-C); white spots randomly splayed, overlapping remaining colors, usually coalescent near superior suture, and also inferior third of last whorl (Figs. 1B-C), barely forming irregular axial bands; small white punctiform spots splayed in inferior half of spire whorls and middle region of body whorl. Spire angle ~40°. Protoconch of 2.5 whorls (Figs. 1D-E), ~12% of length, width of ~6.1 mm, slightly taller than wide; first whorl smooth, gradually spiral narrow aligned punctuations appearing (Figs. 1D-E), lacking axial cords except for relatively regular axial undulations, ~3 per mm, last protoconch whorl with spiral sculpture more spaced, ~8 in last protoconch whorl. Teleoconch of ~2 whorls, profile slightly convex; sculpture similar to that of protoconch last whorl, spiral punctuations (10 in penultimate whorl) and axial undulations; last 1.5 whorls lacking spiral punctuations, only having smooth growth lines (Fig. 1C). Whorls profile weakly convex (Figs. 1A-C). Suture well-marked, not channeled (Fig. 1C). Aperture slightly prosocline (~10° from longitudinal general axis) (Fig. 1B), elliptic; ~52% of shell length, ~55% of shell width. Peristome continuous, reflected, thick, color red (Fig. 1A); outer lip arched, lacking middle tooth; inner lip with inferior half almost straight, superior half as broad, weak callus with ~2/5 of peristome length (Fig. 1A), weakly convex. Inferior half of inner lip covering umbilicus. Umbilicus relatively wide, ~10% of inferior area. Body whorl ~70% shell length; uniform with spire.

**Head-foot.** (Fig. 2B) of normal shape. Color uniformly pale. Columellar muscle thick, 1.5 whorls in length. Inner arrangement of columellar annexed muscles relatively complex. Main columellar bundle (cm) occupying ventral floor of haemocoel, relatively flat, wide slightly broader than half of foot width. Pair of secondary cephalic muscles (sm) with middle and anterior regions approximately double thickness and half width those of columellar main bundle (left muscle slightly broader than right one); originating gradually along dorsal surface of main columellar bundle, running anteriorly on main bundle; anterior third of each muscle with four parallel branches, two medial branches broad (~2/3 of its basal width) inserting in ventral region of perioral region. Branches 3 and 4 respectively tentacular and ommatophore muscles; ommatophore muscles (ou) narrower, mode lateral, inserting in tip of ommatophore; tentacular muscle (tm) longer, inserted in tip of tentacles. Pedal gland (pg) protruding in posterior region of buccal area.

**Mantle organs.** (Fig. 2A) Mantle border thick, lacking pigments. Pneumostome (pn) protected by simple right ventral flap (pv), width ~1/5 of aperture length. Dorsal fold well developed (mf), occupying ~1/3 of dorsal mantle edge length; rounded. Pneumostome (pn) ~1/10 of shell aperture length, bearing exclusively air entrance and urinary gutter. Anus (an) separate aperture located at right, adjacent to pneumostome. Lung of 1.2 whorls in length, wide and elongated; right side ~1.5 times longer than left side. Pulmonary venation well-developed; posterior region of pulmonary vein (cv) protruded; left half only having longitudinal vessels, right half mostly having perpendicular vessels, except for oblique vessels in region preceding pneumostome. Pulmonary

vein (cv) running longitudinally across pallial cavity roof medially or towards right, anterior end trifurcated. Reno-pericardial area beige, slightly triangular, located posteriorly within pallial cavity, its posterior abutting wall of visceral cavity, occupying ~10% of cavity length and ~40% of its width (details below). Rectum (rt) wide. Urinary gutter (ur) broad, smooth, lacking clear transverse folds; running along rectum; anterior urinary gutter surrounding left anal aperture.

**Visceral mass.** (Fig. 2A) ~3 whorls in length. Both digestive gland lobes brown in color; anterior lobe (da) flattened, occupying ~1/5 of visceral volume, located just posteriorly to pallial cavity, continuous to kidney. Posterior lobe (dg) larger, extending 2 spiral whorls, occupying ~60% of visceral volume. Stomach ~1/15 of visceral volume, located between both digestive gland lobes, about 3/4 whorl posterior to pallial cavity (st). Digestive tubes (described below) surrounding anterior lobe of digestive gland. Gonad multi-lobed, cream color, encased between posterior lobe of digestive gland and columella, occupying ~1/3 whorl, ~1/10 of visceral volume.

**Circulatory and excretory systems.** (Fig. A) Pericardium (pc) ~twice as long as wide, located obliquely between middle and left thirds of posterior end of pallial roof, appressed against right lateral side of kidney; occupying ~5% of lung area. Auricle located anteriorly, as continuation from pulmonary vein (cv); ventricle located posteriorly, larger. Kidney (ki) simple, dorso-ventrally flattened; size reported above; somewhat triangular, width ~2/3 of length; internally organized as two distinct regions – a longitudinal hollow cavity on left; filled by sponge-like renal tissue, clearly U-shaped (convexity left) in section. Nephropore (ne) small, longitudinal slit at anterior-left corner of kidney, directed towards right; protected by curved anterior projection of kidney (kf). Urinary gutter (ur) reported above.

**Digestive system.** (Figs. 2A) Mouth (mo) and oral tube wide, muscular. Jaw plate (Figs. 1G-H) thick; cutting edge blunt, sigmoid; lacking inner reinforcement, immersed in jaw thickness. Buccal mass spherical, occupying~1/5 of haemocoel volume. Dorsal surface of oral cavity with well-developed pair of dorsal folds, width of each ~1/3 of dorsal wall width; separated from each other by dorsal chamber (dc) as wide as folds, but broader anteriorly. Odontophore (od) with ~60% of buccal mass volume. Odontophore muscles: **mj**, jaw and peribuccal muscles originating in outer-ventral surface of odontophore cartilages, running towards dorsal, splaying in dorsal wall of oral tube; **m1**, jugal muscles covering entirely haemocoelic structures, more concentrated close to mouth; **m1v**, small pair of ventral protractors jugal muscles, originating in ventral surface of haemocoel close to mouth, running towards posterior, inserting in ventral-posterior region of odontophore close to m2 insertion; **m2**, strong pair of retractor muscles of buccal mass, or radular muscles, originating as single bundle in columellar muscle posterior end, running anteriorly close to median line along ~60% of haemocoel length, inserting as two different bundles in ventro-posterior edge of odontophore, surrounding at some distance radular nucleus; **m3d**, thin layer of longitudinal fibers immersed in dorso-posterior wall of odontophore, preceding esophageal origin; **m4**, main pair of dorsal tensor muscles of radula, very thick, originating in postero-medial region of odontophore cartilages, surrounding outside and medially cartilages, inserting in subradular membrane in its region correspondent to buccal cavity; **m5**, pair of thick auxiliary dorsal tensor muscles of radula, originating half on posterior-medial surface of m4, and half on portion in postero-ventral region of odontophore cartilages, running towards median line covering m4, inserting in subradular membrane by side of m4 insertion; **m6**, horizontal muscle absent; **m7**, absent; **m10**, pair of narrow ventral odontophore protractor muscles, originating in ventro-anterior region of haemocoel, just ventral to mouth, running towards posterior along buccal mass length, inserting in latero-posterior surface of odontophore close to m2 insertions; **m11**, pair of narrow ventral tensor muscles of radula absent. Odontophore non-muscular structures: oc, pair of odontophore cartilages flattened, rather elliptical, anterior region slightly projected anteriorly close to median line, ~1.2 times longer than wide, fused with each other along ~90% in their anterior-medial edge, posterior end roughly rounded; **sc**, subradular cartilage, with expanding

region in buccal cavity protecting subradular membrane. Radular sac short, not extending beyond odontophore.

**Radula.** (Fig. 3) Slightly longer than odontophore; with rachidian teeth, and ~30 pairs of lateral/marginal teeth; no clear distinction between lateral and marginal teeth (Fig. 3A), marginal teeth only slightly narrower than lateral teeth, and more inclined medially (Figs. 3A, B: left side); all teeth with base as long as mesocone, articulating neighboring rows (Figs. 3C); mesocone arched, curved inwards and posterior, ~1.5 longer than wide, apex rounded, base reinforcement slightly more developed, subterminal; no secondary cusps. **Rachidian** tooth (Figs 3B, C: arrow) as large as lateral teeth; base ~twice longer than wide, slightly flattened, barely triangular, with arched posterior edge articulating with neighbor tooth and pair of distal reinforcements; mesocone reduced, ~half size of those of lateral teeth, rounded, covering ~1/3 of base; width and length similar to those of lateral teeth. **Lateral teeth** similar to rachidian, except in being slightly longer, longer asymmetrical mesocone, arched towards medial region; base trapezoid, with medial concavity articulating with neighbor medial tooth and single distal reinforcement (Fig. 3C). **Marginal teeth** starting with no clear boundary with lateral teeth (Figs. 3A, B), occupying ~1/3 of each side; shaped similarly to lateral teeth, except for being slightly narrower and more inclined medially, row practically straight (Fig. 3A: left side).

Salivary glands small, covering ~1/5 of esophagus length, in its anterior quarter region (Fig. 2A: sg), forming two rounded, white, thin masses. Each salivary duct differentiable in middle and anterior side of glands, with ~1/12 of esophageal width (sd). Salivary duct running in both sides of esophageal origin, penetrating buccal mass wall in region close to buccal ganglia, running immersed in buccal dorsal wall along ~1/3 its length. Salivary ducts opening as small slits in located in posterior region of lateral edges of wide dorsal folds.

Esophagus ~1-whorl long, with thin, flaccid walls (Fig. 2A: es); anterior 1/2 weakly broader, crop-like; inner surface simple, smooth. Stomach (st) narrow, curved, weakly bulging; position and size described above (visceral mass); gastric walls thin, weakly muscular; inner surface mostly smooth, but with chitinous inner cover well-developed. Esophageal insertion on right side, intestinal origin on left side, both close to columella. Duct to anterior lobe of digestive gland at short distance from esophagus and intestine intersection (dr) narrow, running shortly towards right, bifurcating; additional left duct (dl) originated in main duct base with ~1/3 of its width, running narrowly towards left and anterior along digestive gland lobe compressed by first intestinal loop (da), periodically possessing short ramifications along its length. Duct to posterior lobe of digestive gland located short distance from intestinal origin, posterior to above-described duct, directed towards opposite side (dd) as wide as anterior duct. Intestine (in) initially as wide as esophageal insertion, gradually narrowing up to ~half that width along its wide sigmoid loop in anterior lobe of digestive gland (in). Rectum and anus position described above (pallial cavity) (rt, an). Anus sessile, as slit in right end of mantle edge directly turned outside; inner surface with 8–10 simple longitudinal, relatively tall folds.

**Reproductive system.** (Fig. 2C, 4) Gonad position described above (visceral mass), composed of 8–10 lobes with minute digitiform acini. Hermaphroditic duct (Fig. 2C: hd) broad; coiled portions occupying anterior 2/3, with very strong coils; insertion strongly curved (Figs. 2C, 4B: hd). Seminal receptacle (Figs. 2C, 4B: sr) relatively large, softly and amply curved (convexity left, wide), insertion of hermaphroditic duct more anterior, at middle level of seminal receptacle duct. Fertilization complex simple, located at narrow and elongated base of seminal receptacle as duct of seminal receptacle; slightly shorter than length of receptacle. Fertilization complex totally immersed in albumen gland, inserting in posterior end of spermoviduct, by side of albumen gland duct, relatively wide, twice wider than receptacle’s duct. Albumen gland (ag) solid, white, elliptical, more-or-less same size as gonad (~1/3 whorl). Albumen gland duct subterminal, connected to distal end of spermoviduct (Fig. 4B), in lateral, small albumen chamber (Fig. 4D:

ac); narrowly connected to distal end of spermoviduct (Fig. 4B: eo). Spermoviduct (Figs. 2C, 24A-B: eo) of ~1.5 whorl in length, slightly narrower than albumen gland, ~10 times longer than wide; prostate wide (pt); glandular walls in uterus only along lateral side (Figs. 4B, E: ut), remaining ~2/3 of uterine inner surface relatively thin-glandular. Presence of accessory genital gland (Figs. 2B, E: as) lying along spermoviduct, flanking masculine furrows (sp), bulging along prostate (pt) side. Pair of masculine furrows lying along accessory genital gland (Figs. 4B, E: sp). Vas deferens originating in simple aperture slightly posterior to end of uterine level (Fig. 4A: vd). Vas deferens not coiled (Figs. 2C, 4C: vd). Genital muscles not detectable. Penis slightly shorter than half of spermoviduct length (Fig. 2C: pe); penis muscle inserted only between penis and epiphallus (Fig. 2C: pm), with small branch connected to vas deferens in its region preceding its insertion (Fig. 4C: pm). Epiphallus (eh) ~1/6 penis' length; vas deferens also inserted in middle level of outer epiphallus wall (Fig. 4C: vd). Epiphallus inner surface with high layer of papillae (Fig. 4D: eh). Internal penial arrangement of folds similar, but with fewer and wider folds, pair of more developed (main) folds (Fig. 4D: pf) not so developed, being wider and lower; papillae slightly smaller than those inside epiphallus also lying along entire distal 2/3 of penis (Figs. 2F, 4D).

**Central nervous system.** (Fig. 2A: nr) More detailed in following studied species, being noteworthy cerebral commissure wide; cerebral node (cd) not so developed; all connectives slightly shorter and all ganglia slightly larger.

**Distribution.** Apparently restricted to Rio de Janeiro region. Remaining records to other states possibly are misidentifications (personal observation, upcoming study).

**Habitat.** Atlantic Rainforest.

**Measurements.** (in mm) MZSP 152925#2 (Figs. 1A-C): 30.2 by 13.3.

**Material examined.** BRAZIL. **Rio de Janeiro**; São Fidélis, Serra da Sapateira, 21°39'S 41°46'W, MZSP 152925, 9 specimens (2 with only shells) (Femorale leg., 2010).

**Taxonomic remarks.** See Discussion item.

## *Anthinus synchondrus* new species (Figs. 5–8)

**ZooBank:** urn:lsid:zoobank.org:act:64ABD2D2-71C4-49D7-AA69-4321AA8595EB

**Types.** Holotype MZSP 152074. Paratypes: MZSP 152252, 2 shells, MZSP 152075, 1 shell from type locality. BRAZIL. **Minas Gerais**; Unaí (W.V. Matos col, iv/2020), Gueda, 16°16'04"S 46°39'43"W, 647-650 m altitude, MZSP 152236, 1 shell.

**Type locality.** BRAZIL. **Minas Gerais**; Unaí, Pedra da Fartura, 16°31'37"S 46°49'18"W, 628-750 m altitude [Weslley Vailant de Matos col, iv.2020].

**Etymology.** The specific epithet is derived from Greek words *syn*, a prefix meaning "together", and *chondros*, meaning "gristle, cartilage". This is an allusion to the total fusion of the odontophore cartilages (Fig. 6D).

**Diagnosis.** Shell about 40 mm; color mosaic of brown-beige spots; spire angle ~35°, protoconch of 3.2 whorls; peristome ~48% of shell length and ~50% of width; width ~54 of length. Mantle edge with projected fold. Reno-pericardial area large. Pre-rectal pallial muscle present. Odontophore with cartilages totally fused; pair m4 ~50% originated from cartilage, remaining on m4. Jaw plate narrow. Radula with elongated, spatula-like mesocone, rachidial slightly different from neighboring teeth. Seminal receptacle tightly curved, hermaphrodite duct inserting in its

base perpendicularly. Accessory genital gland in spermoviduct large; with two similar sperm-grooves. Penis ~60% of spermoviduct; penis muscle with pair of small branches in epiphallus; epiphallus papillae low, uniform, restricted to it; pair of larger folds composed of transverse subfolds.

**Distinctive description**

**Shell.** (Figs. 5A–L) Adult shell around 40 mm, conical-oval; apex blunt; wider at middle of last whorl; width ~54% of shell length. Walls weakly thick. Dorso-ventrally weakly flattened (Fig. 5E). Color light beige and brown squared spots, both predominating (Fig. 5C) or slightly brown predominating (Figs. 5H, J); brown spots slightly randomly distributed along 4-5 spiral, wide, rather isometric bands in last whorl, 2-3 spiral bands in penultimate whorl; usual concentration of brown pigment preceding peristome. Spire angle ~35°. Protoconch of 3.2 whorls (Figs. 5B, J, K), ~23% of length, width of ~7 mm, ~twice taller than wide; first whorl smooth, gradually axial and spiral narrow cords appearing (Figs. 5K, L), initially both predominating, gradually axial cords slightly stronger, from suture to suture, interspaced slightly broader than each cord, ~10 per mm; transition to teleoconch clear, slightly prosocline (Figs. 5B, H, K). Teleoconch sculpture similar to that of protoconch last whorl, as axial, uniform, complete cords and growth lines, gradually fading in last whorl (Figs. 5C, H, J); of ~2.3 whorls. Whorls profile weakly convex (Figs. 5G-J) (Figs. 5A-C, F, I, J). Suture well-marked, weakly channeled (Figs. 5J, K, L). Aperture slightly prosocline (~5° from longitudinal general axis) (Fig. 5B), oval; ~48% of shell length, ~50% of shell width. Peristome well-reflected, thick, color variating from pale beige (Figs. 5A, G) to white (Fig. 5I); outer lip arched, lacking middle tooth; inner lip with inferior half widely concave, superior half as narrow, weak callus with ~1/3 of peristome length (Figs. 5A, I), usually concave, rarely convex (Fig. 5G). Inferior half of inner lip intensely covering umbilicus (Figs. 5A, D, G, I). Umbilicus narrow (Fig. 5B). Body whorl ~55% of shell length; usually uniform with spire (Figs. 5A, G, I).

**Head-foot.** Similar features as *A. multicolor*, including columellar muscle having only two layers; i.e., secondary head muscles (ou, tm) originating direct from pair of secondary muscles (tm).

**Mantle organs.** (Fig. 6A) most characters similar to *A. multicolor*. Distinctions and remarks following. Mantle edge dorsal fold well developed (mf), with elongated edge pointed to left. Anus directly opening externally (ventrally), preceded by right longitudinal folds (Fig. 5N: af). Urinary gutter (ur) slightly broader, with slightly taller perpendicular folds.

**Visceral mass.** Similar features as preceding species.

**Circulatory and excretory systems.** (Fig. 6A) Most features similar to *A. multicolor*; remarks following. Pericardium (pc) lightly more elongated, ~3-times as long as wide. Kidney (ki) tissue clearly U-shaped (convexity left). Nephropore (ne) also protected by curved anterior projection of kidney. Urinary gutter (ur) slightly broader, mainly along its middle portion. Narrow urinary gutter surrounding left edge of anus (an) also present.

**Digestive system.** (Figs. 6C–8A) General features similar to those of *A. multicolor*, distinctions and interesting data following. Jaw slender, with uniform width along its length (Figs. 5M, 8A: jw), weak central beak; transverse ribs very weak, almost imperceptible; commarginal sculpture wanting. Buccal mass and odontophore (Fig. 6D) lacking m1a, m1v; **m3**, only detected in latero-dorsal region (Figs. 6C, 8A: m3); **m2**, slightly thinner, insertion narrow on m4 base (Fig. 6D); **m4**, very thick, relatively short; **m5**, thin and elongated, origin about half on m4 and half on posterior region of cartilages; **m7**, small narrow pair, origin in posterior end of cartilages, running inside radular sac, splaying along its inner surface. Pair of odontophore cartilages entirely fused with each other along their inner-ventral edge (Fig. 6D: oc). **Radula** (Fig. 7) with same general features of preceding species; with rachidian teeth, and ~40 pairs of lateral/marginal teeth; no

clear distinction between lateral and marginal teeth (Fig. 7C); differing in having longer, rather rectangular mesocone, ~3 times longer than wide, covering ~1/3 of posterior tooth (Figs. 7B-C), mesocone apex rounded; long mesocone reinforced by longitudinal fold in inner concavity, continuous with base; base with similar characters as preceding species. **Rachidian** tooth (Figs 7A-C: arrow) slightly narrower than lateral teeth, with mesocone with ~half size of neighboring mesocones (Fig. 7C). **Lateral teeth** with weak medial inclination, rarely with bifid cutting edge (Fig. 7A: right side). **Marginal teeth** starting with no clear boundary with lateral teeth; shaped similarly to lateral teeth, except for being weakly smaller and narrower, row slightly more inclined (Fig. 7A: left side) along ~1/3 of each side. Salivary glands apertures as long longitudinal slits (Fig. 8A: sa), located in posterior region of lateral edges of wide dorsal folds (df). Esophagus broader all along its length (Fig. 6C: es). Stomach (st) ~50% larger, walls with considerable muscular walls; stomach inner surface (Fig. 6B) with fold (sf) running between anterior duct to digestive gland (dr) and intestine (in) more developed. Esophageal-anterior duct to digestive gland also bifid (Fig. 6C), left duct (dl) almost as wide as right duct (dr), much longer. Anus turned externally (Fig. 6A: an); inner surface also with 8–10 simple, longitudinal, subterminal, tall folds (Fig. 5N: af).

**Reproductive system.** (Figs. 8B-F) General characters similar to those described for *A. multicolor*, distinctions and remarks following. Hermaphroditic duct (Fig. 8B: hd) with broader coiled portions along its middle and distal thirds, with fewer but wider coils; insertion lacking curve (Fig. 8C). Seminal receptacle (Figs. 8B-C: sr) large, strongly and tightly curved (convexity left), forming distal blunt beak disposed pointing to hermaphroditic duct insertion. Spermoviduct (Figs. 8B-C: eo) with glandular walls in uterus only in ventral side (Fig. 8F: ut), remaining walls relatively thin-glandular. Presence of accessory genital gland (Figs. 8D, F: as) lying along spermoviduct, flanking masculine furrows (sp), bulging along prostate (pt) side. Pair of masculine furrows narrow, one lying along accessory genital gland, and other along its medial edge, ~half deep (Figs. 8D, F: sp). Vas deferens (vd) originating in simple junction of masculine furrows (Fig. 8D: vd), no vaginal fold. Vas deferens relatively broad, intensely coiled in its portion on free oviduct and vagina (Fig. 8B: vd). Penis slightly longer than half of spermoviduct length (Fig. 8B: pe); penis muscle inserting terminally, short, broad (Figs. 8B, E: pm), with pair of narrow branches connected to epiphallus. Epiphallus (eh) ~1/5 penis' length; vas deferens inserted subterminally on middle level of epiphallus wall (Fig. 8E: vd). Epiphallus inner surface as thick glandular, uniform layer of papillae. Internal penial surface with single chamber (Fig. 8E); arrangement of inner folds as in Fig. 8E, with 8-10 longitudinal wide folds, almost no interspace, in basal half; these folds gradually coalescent in middle penial region, additionally appearing narrower transverse folds in distal region; these folds forming two aligned bulged portions in penis side, producing pair of longitudinal cancelled wide folds (Fig. 8E: pf), separated by narrow furrow continuous with epiphallus aperture.

**Central nervous system.** Nerve ring located across buccal mass (Fig. 6C: nr). Pair of cerebral ganglia (ce) almost fused; cerebral commissure slightly narrower than ganglia; each ganglion about as wide as adjacent esophageal section; several wide nerves originating in cerebral antero-lateral region. Cerebral node or gland (cd) located in postero-medial quadrant, with ~1/8 each ganglion's size. On each side, parallel, rather narrow connectives (cn) running between cerebral ganglion and ventrally located fused pedal and pleural ganglia (pp), accompanied by blood vessels (bv) issuing from anteriorly-directed aorta. At least six pairs of nerves originating from anterior side of pedal-visceral ganglion complex. Pair of statocysts not seen.

**Distribution.** So far known in region of Unaí, Minas Gerais, Brazil.

**Habitat.** Cerrado biome, semi-dry forest, 628-750 m altitude.

**Measurements.** (length and width in mm) Holotype MZSP 152074 (Figs. 5A-F): 36.9 by 19.7; paratype MZSP 152252 (Figs. 5I-J): 36.4 by 19.6.

**Material examined.** Types (reported above).

**Taxonomic remarks.** See Discussion item.

***Anthinus vailanti* new species** (Figs. 9-12)

**ZooBank:** urn:lsid:zoobank.org:act:5CF7693D-4C22-4E66-9FCA-B0B821981E0B

**Types.** Holotype MZSP 154382. Paratypes: MZSP 152118, 3 spm, MZSP 152244, 2 shells, MZSP 152119, all from type locality.

**Type locality.** BRAZIL. **Minas Gerais**; North of Brasilândia de Minas, 16°59'29"S 46°00'29"W, 630-635 m altitude [Weslley Vailant de Matos col, iv.2020].

**Etymology.** The specific epithet is in honor of Weslley Vailant de Mattos, from Alcobaça, Bahia, the field collector responsible for most of the new discoveries.

**Diagnosis.** Shell about 30 mm; color mostly monochromatic; spire angle ~50°, protoconch of 3 whorls; peristome ~55% of shell length and ~60% of width; width ~60 of length. Mantle edge with pointed fold. Reno-pericardial area medium-sized. Pre-rectal pallial muscle present. Odontophore with cartilages ~90% fused with each other; pair m4 ~80% originated from cartilage, remaining on m4. Jaw plate broad, with central fold. Radula with elongated, spatula-like mesocone, rachidial slightly different from neighboring teeth. Seminal receptacle widely curved, hermaphrodite duct inserting in its middle region, perpendicularly. Accessory genital gland in spermoviduct large; with single, coiled sperm-groove. Penis ~60% of spermoviduct; penis muscle with pair of small branches in epiphallus; epiphallus papillae of medium size, not-uniform, extending to penial chamber; pair of larger folds simple, fused with each other proximally.

**Distinctive description**

**Shell.** (Figs. 9A–J) Adult shell around 30 mm, conical-oval; apex blunt; wider at middle of last whorl; width ~60% of shell length. Walls thin. Dorso-ventrally weakly flattened (Fig. 9J). Color uniform reddish brown in ~half of specimens (Figs. 9A-G); to sparse squared pale brown spots on uniform cream base in other half of specimens (Figs. 9H-J), spots less dense than preceding species, but with similar remaining features, with areas denser (Fig. 9J) and areas almost lacking spots (Fig. 9I). Periostracum heavy, deciduous in some areas, mainly in apex (Figs. 9A-G). First spire whorls white in superior half, gradually becoming pale brown in inferior half (Figs. 9D-E). Spire angle ~50°. Protoconch of 3.0 whorls (Figs. 9B, E), ~20% of length, width of ~8.8 mm, slightly taller than wide; first whorl smooth, gradually axial and spiral narrow cords appearing (Figs. 9D-E), initially both predominating, gradually axial cords slightly stronger, from suture to suture, interspaced slightly broader than each cord, ~6 per mm, last protoconch whorl with spiral sculpture more spaced, producing rectangular spaced with axial lines; transition to teleoconch clear, slightly prosocline (Fig. 9E). Teleoconch sculpture similar to that of protoconch last whorl, as axial, uniform, complete cords and growth lines, gradually small punctuations appearing on axial lines before penultimate whorl, aligned in order to produce spiral punctuated lines, ~20 in penultimate whorl (Fig. 9E), lying next to peristome (Figs. 9C, F, J); of ~2.2 whorls. Whorls profile weakly convex (Figs. 9A-J). Suture well-marked, weakly channeled (Figs. 9I). Aperture slightly prosocline (~20° from longitudinal general axis) (Fig. 9B), elliptic; ~55% of shell length, ~60% of shell width. Peristome reflected, thick, color white (Figs. 9A, F, H); outer lip arched,

lacking middle tooth; inner lip with inferior half weakly concave, superior half as narrow, weak callus with ~2/3 of peristome length (Figs. 9A, F, H), almost straight. Inferior half of inner lip covering umbilicus. Umbilicus relatively wide (Fig. 9J). Body whorl ~80% of shell length; usually uniform with spire (Figs. 9F-J) to slightly broader (Figs. 9A-C).

**Head-foot.** Similar features as *A. multicolor*, except for more developed pedal glands, bulging in haemocoel floor.

**Mantle organs.** Most characters similar to *A. multicolor*. Except for urinary gutter ~5% broader,

**Visceral mass.** Similar features as preceding species.

**Circulatory and excretory systems.** Most features similar to *A. multicolor*; except for renal anterior fold proportionally smaller, ~half size. Renal glandular lobe also U-shaped, but with ventral branch with double thickness of dorsal branch.

**Digestive system.** General features similar to those of *A. multicolor*. Remarks following. Jaw plate (Fig. 9K-L) much broader and thicker; cutting edge blunt, with median transverse reinforcement (Fig. 9L) in its ventral side. Buccal mass possessing m1v pair. Pair of salivary ducts slightly broader. Odontophore (Fig. 10A) differing in having **m5** pair only ~20% originating from cartilages, remaining originating on m4; **m7** pair originated in dorsal-inner surface of posterior region of cartilages; odontophore cartilages (oc) only ~90% fused, with small posterior remaining notch. **Radula** (Fig. 11) similar to that of *A. synchondrus*, with ~50 pairs of lateral/marginal teeth (Figs. 11A, D); distinctions following. **Rachidian** tooth (Figs. 11B, E: arrow) further smaller and narrower; mesocone slightly claviform. **Lateral teeth** not inclined, pointing posteriorly only; mesocone slightly more elongated, covering ~1/3 of neighboring posterior tooth. **Marginal teeth** inclined outside, located in more arched row (Figs. 11C, D: left side). Anus subterminal folds taller and stronger, 4-5 in number only; occupying ~10% of rectum length.

**Reproductive system.** (Figs. 10B-D, 12A–B) General characters similar to those described for *A. multicolor*, distinctions and remarks following. Hermaphroditic duct (Fig. 10C: hd) also broad; coiled portions occupying almost its entire length, except for short stretch at both ends; insertion lacking curve (Fig. 10B). Seminal receptacle (Figs. 10B–C: sr) relatively small, strongly and widely curved (convexity left), insertion of hermaphroditic duct in middle of concavity. Spermoviduct (Figs. 10B, C: eo) with narrower prostate (pt); glandular walls in uterus only along latero-ventral side (Figs. 10D, 12A: ut), remaining ~1/3 of uterine inner surface relatively thin-glandular. Accessory genital gland (Figs. 10B, D, 12A: as) also present and large. Only single detectable masculine furrow lying along accessory genital gland (Figs. 10D, 12A: sp). Vas deferens originating in simple aperture in end of uterine level (Fig. 12A: vd), also lacking vaginal fold. Vas deferens also coiled in its portion on feminine tubes, but less intensely (Fig. 10C: vd). Presence of pair of genital muscles (Fig. 10C: gm) in ventral side of penis base. Penis slightly longer than half of spermoviduct length (Fig. 10C: pe); penis muscle also with pair of narrow branches connected to epiphallus (Figs. 10C, 12B: pm). Epiphallus (eh) ~1/6 penis' length; vas deferens also inserted subterminally on middle level of epiphallus wall (Fig. 12B: vd). Epiphallus inner surface with high layer of papillae (Fig. 12B: eh). Internal penial arrangement of folds similar, but with pair of more developed (main) folds (Fig. 12B: pf) narrower and slightly taller; papillae similar to those inside epiphallus also lying at side of these main folds (Fig. 12B).

**Central nervous system.** With similar attributes as preceding species.

**Distribution.** So far known from type locality.

**Habitat.** Cerrado biome, semi-dry forest, 630-635 m altitude.

**Measurements.** (in mm) Holotype MZSP 154382 (Figs. 9A-E): 29.0 by 17.7; paratypes MZSP 152118 (Figs. 9F-G): 27.9 by 17.7; MZSP 152244 (Figs. 9H-J): 29.5 by 16.5.

**Material examined.** Types (reported above).

**Taxonomic remarks.** See Discussion item.

### *Anthinus morenus* new species (Figs. 13–15)

**ZooBank:** urn:lsid:zoobank.org:act:A34D18E0-47B6-40C8-A3C8-7FF3367E3AE5

**Types.** Holotype MZSP 151891. Paratypes: MZSP 152047, 12 spm from type locality (3 with only shells), MZSP 152048, 2 shells. BRAZIL. **Minas Gerais**; Paracatu, Gruta Sapezal, 16°48'03"S 46°53'47"W, 700-710 m altitude, MZSP 152195, 3 shells (WV Matos col., iv.2020).

**Type locality.** BRAZIL. **Minas Gerais**; Paracatu, São José do Sapezal, 16°48'03"S 46°53'47"W, 700-710 m altitude [Weslley Vailant de Matos col, iv.2020].

**Etymology.** The specific epithet is in apposition, a Latinization based on the Portuguese word "moreno", meaning dark-brown colored, an allusion to the dark shell color.

**Diagnosis.** Shell about 35 mm; color usual mosaic of brown and beige spots; spire angle ~50°, protoconch of 3 whorls; peristome ~51% of shell length and ~51% of width; width ~60 of length. Mantle edge with simple, rounded fold. Reno-pericardial area small-sized. Pre-rectal pallial muscle present. Odontophore with cartilages ~50% fused with each other; pair m4 ~40% originated from cartilage, remaining on m4. Jaw plate narrow, lacking central beak. Radula with elongated, spatula-like mesocone, rachidial rather similar to neighboring teeth. Seminal receptacle tightly curved, hermaphrodite duct inserting in its base, perpendicularly. Accessory genital gland in spermoviduct large; with pair of thick-walled sperm-grooves. Penis ~50% of spermoviduct; penis muscle mostly inserted to epiphallus; epiphallus papillae tall, restricted to it; single, large inner fold.

**Description**

**Shell.** (Figs. 13A-H, J-K) Adult shell around 35 mm, conical-oval; apex blunt; greatest width on last whorl; width ~60% shell length. Walls relatively thin. Color beige, with three spiral, equidistant brown bands up to penultimate whorl, five in last whorl; mosaic of sparse, apparently random transverse (axial) spots, irregular in size, normally limited to two neighboring spiral bands, but sometimes extending further (Fig. 13C, K). Spire angle ~50°. Slightly flattened dorso-ventrally, dorso-ventral axis ~80% of latero-lateral axis (Fig. 13E). Protoconch of 3 whorls (Figs. 13B, F, G, K), ~20% of length, width of ~7 mm; first whorl smooth, gradually axial narrow cords appearing (Figs. 13G-H), from suture to suture, interspaced similar to each cord, ~10 per mm; transition to teleoconch clear, slightly prosocline (Figs. 13B, F, G, K). Teleoconch of ~2.5 whorls; sculpture similar to that of protoconch last whorl in its first whorl, as axial, uniform, complete cords and growth lines (Fig. 13F); after first teleoconch whorl axial cords gradually disappearing, being substituted by rather irregular axial undulations and minute spiral aligned punctuations (Figs. 13C, F, J), ~25 spiral lines in penultimate whorl, interval between lines equivalent to their width up to double of it; spiral punctuated lines relatively uniformly covering surface up to peristome (Figs. 13B, C, K). Whorls profile slightly convex. Suture well-marked, slightly channeled (Figs. 13B, F, J). Aperture slightly prosocline (~7° from longitudinal general axis) (Fig. 13B), elliptic; ~51% of shell length, ~51% of shell width. Peristome reflected, thick, pure white (Figs. 13A, B) to pale beige (Fig. 13J); outer lip arched, lacking tooth (Figs. 13A, J); inner lip

uniformly arched, concave; callus narrow, in superior third (Figs. 13A, J). Inferior 2/3 of inner lip partially covering umbilicus. Umbilicus relatively narrow (Fig. 13D). Body whorl ~84 shell length; usually looking wider than if spire had uniform growth (Figs. 13C, K).

**Head-foot.** (Fig. 14A) of normal shape, with characters similar to *A. multicolor*. Differing in pair of secondary cephalic muscles thicker, broader, and separated from each other almost up to their posterior end. Pedal gland not protruding in haemocoel.

**Mantle organs.** (Fig. 13L) most features similar to *A. multicolor*, differences following. Pallial roof uniformly pigmented dark (pu). Except for pulmonary vein (cv), all remaining pulmonary vessel not protruded, only visible with light passing through roof. Secondary mantle edge fold (mf) smaller, not pointed at left. Lung longer, almost 2 whorls. Reno-pericardial structures smaller, occupying ~10% of roof area. Urinary gutter smooth, lacking transverse folds.

**Visceral mass.** of similar attributes of preceding species.

**Circulatory and excretory systems.** (Fig. 13L) with same features as preceding species, except in being ~40% smaller.

**Digestive system.** General features similar to those of preceding species. Remarks following. Jaw plate (Fig. 13I) narrow, relatively thinner, including its cutting edge. Pair of salivary glands ~30% less developed. Odontophore (Fig. 14B) differing in having **m5** pair with narrower origin, ~40% originating from cartilages, remaining originating on m4; **m7** absent; odontophore cartilages (oc) only ~50% fused, with long posterior notch separating both. **Radula** (Fig. 15) mostly similar to *A. synchondrus,* with ~60 pairs of lateral/marginal teeth; distinctions and remarks following. Mesocone inner reinforcement clearly shown in Fig. 15C (central column, with teeth in profile), running along mesocone, becoming thicker and attaching to base. All teeth narrower and more elongated, ~4-5 times longer than wide (Figs. 15B, F); some rare column bifid (Fig. 15C: right side). **Rachidian** tooth of difficult visualization (Figs. 15A, B, E), ~1/3 narrower and shorter than lateral tooth, and slightly dislocated anteriorly in relation to its row (Fig. 15D: arrow). **Lateral teeth** with very elongated mesocone, widely curved posteriorly (Fig. 15D). **Marginal teeth** with no clear boundary with lateral teeth, as gradual diminishment in lateral regions (Figs. 15E-F, inferior region).

**Reproductive system.** (Figs. 14C-F) General characters similar to those described for preceding species, distinctions and remarks following. Hermaphroditic duct (Fig. 14D: hd) also broad; coiled portions occupying almost its entire length, except for short stretch at both ends (Fig. 14E). Seminal receptacle (Figs. 14D-E: sr) relatively small, strongly and tightly curved (convexity left), insertion of hermaphroditic duct in receptacle base, lacking curve (Fig. 14E). Spermoviduct (Figs. 14D, E: eo) with glandular walls in uterus along its entire outer side (Figs. 14E, F: ut). Accessory genital gland (Figs. 14E, F: as) also present and large. Pair of masculine furrows narrow, one lying along accessory genital gland, and other along its medial edge, ~half deep (Figs. 14E, F: sp). Vas deferens originating in simple aperture in end of uterine level, also lacking vaginal fold. Vas deferens also coiled in its portion on feminine tubes, but less intensely (Fig. 14D: vd). Presence of single genital muscle (Fig. 14D: gm) in ventral side of vaginal base. Penis ~half of spermoviduct length (Fig. 14D: pe); penis muscle mostly inserted in epiphallus (Figs. 14D: pm) with additional small portion connected to penis distal end. Epiphallus (eh) ~1/4 penis' length; vas deferens also inserted subterminally in middle level of epiphallus wall (Figs. 14C, D: vd). Epiphallus inner surface with high layer of papillae (Fig. 14C: eh). Internal penial arrangement of folds similar, but with single more developed (main) fold (Fig. 14C: pf) broad and slightly taller; penial wall substantially thick muscular, inner set of folds (Fig. 14C) softer and simpler.

**Central nervous system.** With equivalent attributes as preceding species; except having longer cerebral commissure

**Distribution.** Paracatu region, Minas Gerais.

**Habitat.** Cerrado biome, semi-dry forest, 700-710 m altitude.

**Measurements.** (length and width in mm) Holotype MZSP 152891 (Figs. 13A-H): 35.8 by 21.0; paratype MZSP 152047#1 (Figs. 13I-L): 34.8 by 20.8.

**Material examined.** Types (reported above).

**Taxonomic remarks.** See Discussion item.

***Anthinus savanicus* new species** (Figs. 16-19)

**ZooBank:** urn:lsid:zoobank.org:act:7816D208-22FE-4C19-BEE5-ED2DD68B3FAD.

**Types.** Holotype MZSP 154391. Paratypes MZSP 153897, 6 spm, MZSP 152171, 4 shells, all from type locality.

**Type locality.** BRAZIL. **Goiás**; north of Formosa, 15°21'50"S 47°27'08"W [Weslley Vailant de Matos col, ix.2012].

**Etymology.** The specific epithet is derived from the regional biome, called *Savana Acidentada* (rugged savanna).

**Diagnosis.** Shell about 32 mm; color usual mosaic of brown and beige spots; spire angle ~50°, protoconch of 2.8 whorls; peristome ~48% of shell length and ~51% of width; width ~60% of length. Mantle edge with projected fold. Reno-pericardial area large-sized. Pre-rectal pallial muscle present. Odontophore with cartilages ~85% fused with each other; pair m4 ~50% originated from cartilage, remaining on m4. Jaw plate broad, thick. Radula with elongated, spatula-like mesocone, rachidial rather similar to neighboring teeth. Seminal receptacle tightly curved, hermaphrodite duct inserting in its base, curved. Accessory genital gland in spermoviduct small; with pair of sperm-grooves, one being T-shaped. Penis length ~80% of spermoviduct; penis muscle mostly inserted in penis distal end, with thick branch to epiphallus; epiphallus papillae tall, restricted to it; large pair of penial inner folds at end of secondary folds.

**Distinctive description**

**Shell.** (Figs. 16A–F, I–K) Adult shell around 32 mm, conical-oval; apex blunt; wider at middle of last whorl; width ~60% of shell length. Walls slightly thick. Dorso-ventrally slightly flattened (Figs. 16D, E). Color light beige and brown squared spots, both predominating; brown spots randomly distributed along 4-5 spiral, wide, rather isometric bands in last whorl, 2-3 spiral bands in penultimate whorl; usual concentration of brown pigment preceding peristome (Figs. 16C, J). Spire angle ~50°. Protoconch of 2.8 whorls (Figs. 16B, D, F, J), ~19% of length, width of ~7.7 mm, slightly taller than wide; 5-6 narrow, spiral lines present since first whorl, equally spaced, gradually more spiral and axial narrow lines appearing (Fig. 16F), initially spiral lines predominating, gradually in last whorl both predominating, gradually axial cords slightly stronger, from suture to suture, interspaced by similar width as lines', ~7 per mm; transition to teleoconch clear, slightly prosocline (Figs. 16B, F, J). Teleoconch sculpture similar to that of protoconch's last whorl, as axial, uniform, complete cords and growth lines; cords containing punctuations, aligned with their neighboring cords producing spiral punctuated cords (Figs. 16A, B, I, J); ~18 lines in penultimate whorl. Suture well-marked, weakly channeled (Figs. 16J, K). Aperture slightly prosocline (~10° from longitudinal general axis) (Fig. 16B), elliptical; ~48% of shell length, ~51% of shell width. Peristome reflected, thick, color white (Figs. 16A, I, K); outer lip arched, lacking middle tooth; inner lip with inferior half shallowly concave, superior half as

narrow, weak callus with ~2/5 of peristome length (Figs. 16A, I, K), usually convex. Inferior half of inner lip intensely covering umbilicus. Umbilicus relatively wide (Fig. 16E). Body whorl ~80% of shell length; uniform with spire (Figs. 16C, J, K).

**Head-foot.** (Fig. 16H) Similar features as preceding species.

**Mantle organs.** (Fig. 16H) most characters similar to preceding species. Distinctions and remarks following. Pallial cavity ~0.75 of whorl. Mantle edge dorsal fold (mf) with very elongated edge pointed to left. Urinary gutter (ur) slightly narrow, transverse folds low.

**Visceral mass.** Similar features as preceding species.

**Circulatory and excretory systems.** (Fig. 16H) Most features similar to preceding species; except in being proportionally larger, occupying ~40% of pallial cavity (pc, ki).

**Digestive system.** (Figs. 17A-B) General features similar to those of preceding species, distinctions and interesting data following. Jaw strong, well-arched, with central inner reinforcement (Fig. 16G), weak central beak; transverse ribs very weak, almost imperceptible; commarginal sculpture wanting. Buccal mass and odontophore (Fig. 17B) lacking m1a, m1v and m3d; **m3p**, well-developed, extending along base of esophageal origin; **m4**, very thick, relatively short; **m5**, thin and short, origin about half on m4 and half on posterior region of cartilages; **m7**, absent. Pair of odontophore cartilages ~85% fused, with small posterior medial notch separating both cartilages (Fig. 17B: oc). **Radula** (Fig. 18) virtually similar to preceding *A. morenus*, with ~50 pairs of lateral teeth; but with all teeth slightly shorter and wider (Figs. 18A, C, D), mesocone reinforcement (Fig. 18B) and bifid column (Fig. 18A: center) also detected. **Rachidian** tooth (Fig 18E: arrow) similar to lateral teeth, except in being slightly smaller. **Marginal teeth** also turned outside, but proportionally larger, more similar to lateral teeth (Fig. 18E: left side). Salivary ducts narrow, elongated (Fig. 17A: sd). Esophagus broader all along its length, inner folds longitudinal, rather irregular (Fig. 17A: es). Stomach (st) large, walls considerably muscular; stomach inner surface (Fig. 17A) lacking developed fold, only having small transverse folds (sf) close to duct to digestive gland (dl). Esophageal-anterior left duct (dl) also broad and long.

**Reproductive system.** (Figs. 17C, D, 19A–C) General characters similar to those described for preceding species, distinctions and remarks following. Hermaphroditic duct (Fig. 17C: hd) with coiled portions only in distal half, with fewer and simpler coils; insertion with strong curve (Figs. 17C, 15A: hd). Seminal receptacle (Figs. 17C, 19A: sr) large, strongly and tightly curved (convexity left), forming distal blunt beak pointing to hermaphroditic duct insertion. Spermoviduct (Figs. 17C, 19A, B: eo) with glandular walls in uterus relatively low and splayed (Fig. 17D: ut). Accessory genital gland relatively smaller (Figs. 17D, 19B: as), only present in basal 2/3 of spermoviduct, distal third lacking this gland (Fig. 19A); masculine furrows composed of pair of separated folds flanking wide groove (Figs. 17D, 19B: sp). Vas deferens (vd) originating in simple junction of masculine furrows (Fig. 19B: vd), no vaginal fold. Vas deferens relatively broad, coiled only in some sparce portions (Fig. 17C: vd); becoming very narrow along penial region. Penis almost as long as spermoviduct length (Fig. 17C: pe); penis muscle inserting as pair of relatively same-sized wide branches attached both, to epiphallus and to distal penial end (Fig. 17C: pm). Epiphallus (eh) ~1/6 penis' length; vas deferens inserted subterminally on middle level of epiphallus wall. Epiphallus inner surface as thick glandular, uniform layer of papillae (Fog. 10C: eh. Internal penial surface with single chamber (Fig. 19C); arrangement of inner folds as in Fig. 19C, with 8-10 longitudinal wide, low folds, almost no interspace, along entire penis length; these folds distally as pair of longitudinal multilobed folds (Fig 19C: pf) restricted to single side, with wide, smooth groove separating them; this groove continuous to epiphallus aperture (Fig. 19C).

**Central nervous system.** with equivalent attributes as preceding species, except for cerebral pair of ganglia more closely located, with wider commissure; and for slightly more antero-posteriorly elongated cerebral and pedal pair of ganglia.

**Distribution.** So far known the type locality.

**Habitat.** Cerrado biome.

**Measurements.** (length and width in mm) Holotype MZSP 154391 (Figs. 16A–C): 32.5 by 20.3; Paratype MZSP 153897 (Figs. 16I–L) 31.5 by 18.7; Paratype MZSP 152171 (Fig. 16K): 35.3 by 20.1.

**Material examined.** Types (reported above).

**Taxonomic remarks.** See Discussion item.

## Genus *Catracca* new genus

**ZooBank.** urn:lsid:zoobank.org:act:C9AEAD6E-EA30-4584-A884-AB967AE8CE6A

**Diagnosis.** shell of relatively thick walls; spire tall, with blunt tip; narrowly umbilicate. Protoconch of three whorls; first whorl smooth; narrow close-placed axial cords uniformly disposed in remaining whorls, from suture to suture. Peristome deflected, callus narrow. Color uniform, lacking spots. Pallial cavity lacking septum, weak vascular network. Kidney with anterior projection protecting nephrostome. Odontophore with pair m5 originated part from pair m4 and part from pair m2. Stomach with walls relatively muscular. Seminal receptacle balloon-like, non-curved. Spermoviduct lacking accessory genital gland. Epiphallus short, rounded, amply connected to penis. Penis internally comprising two chambers separated by strong and complex folded, transverse, ring-like, its distal edge comprising tall fold with well-developed sphincter.

**Type species.** ***Catracca uhlei*** new species.

**List of included taxa.** ***Catracca uhlei***.

**Etymology.** The genus' name is in apposition and is derived from the Portuguese word for ticket gate, *catraca*, as a make-up word; anyhow, its central region usually has a reinforced core sustaining the bars, this piece resembles the described shell. Gender: feminine.

**Taxonomic discussion.** see Discussion item.

### ***Catracca uhlei*** **new species** (Figs. 20–24)

**ZooBank:** urn:lsid:zoobank.org:act:F8EEDDB8-F57F-40C2-831E-5F667C529BCF

**Type specimens.** Holotype MZSP 151900. Paratypes: MZSP 151901, 17 shells, MNRJ 23647, 1 shell, USNM 1661730, 1 shell, all from type locality. BRAZIL. **Minas Gerais**; Itacarambi (W.V. Matos col), entrance of Parque Nacional do Peruaçu (ii.2019), 15°10'30"S 44°13'24"W, 530 m altitude, MZSP 152161, 21 shells, MZSP 152222, 15 shells, MZSP 152242, 11 shells, Vargem Grande, 15°00'50"S 44°04'34"W, 466 m altitude, MZSP 151871, 3 specimens (7.ii.2020); Januária, border of Parque Nacional do Peruaçu, 14°57'55"S 44°04'21"W, 485 m altitude, MZSP 151893, 1 specimen, MZSP 151892, 10 shells (i.2020).

**Type locality.** BRAZIL. **Minas Gerais**; West of Itacarambi, 15°00’38”S 44°07’10”W, 540-550 m altitude [Weslley Vailant de Matos col, ii.2019].

**Etymology.** The specific epithet is in honor of Mauricio Sergio Uhle, São Paulo, a shell collector and sponsor of expeditions.

## Description

**Shell.** (Figs 20A–I) Adult shell around 45 mm, conical-oval; apex blunt; wider on last whorl; width ~1/2 shell length. Walls relatively thick. Color uniform light beige or yellowish pale beige; some few specimens with reddish-brown tone in inferior third of spire whorls (Fig. 20F). Spire angle ~40°. Protoconch of 3 whorls (Figs. 20B, E), ~23% of length, width of ~9 mm; first whorl smooth, gradually axial narrow cords appearing (Figs. 20H, I), running from suture to suture, interspaces similar to each cord’s width, ~10 per mm; transition to teleoconch distinct, slightly prosocline (Figs. 20E, H). Teleoconch of ~2 whorls, profile slightly convex; sculpture similar to that of protoconch last whorl, as axial, uniform, complete cords and growth lines, slightly more irregular on last whorl (Figs. 20A, C, D, G). Suture well-marked, slightly channeled (Figs. 20B, C, E, G). Aperture slightly prosocline (~10° from longitudinal general axis) (Fig. 20B), oval; ~45% of shell length, ~50% of shell width. Peristome continuous, reflected, thick, same color as remaining shell; outer lip arched, usually with small, blunt tooth medially (Figs. 20A, D, G) absent in younger adults (Fig. 20F); inner lip with inferior half almost straight, superior, parietal half appressed to body whorl, with thick callus, sometimes planar in last whorl (Figs. 20D, F) sometimes flanked by shallow outer furrow (Figs. 20A, F). Inferior half of inner lip partially covering umbilicus. Umbilicus relatively wide (Figs. 20A, D, G), ~10% of inferior area. Body whorl ~2/3 shell length; usually regularly increasing in size (Figs. 20A, D, F), more rarely wider (Fig. 20G).

**Head-foot.** (Fig. 21B) of normal shape. Color uniformly pale. Columellar muscle thick, 1.5 whorls in length. Inner arrangement of columellar annexed muscles relatively complex. Main columellar bundle (cm) occupying ventral floor of haemocoel, relatively flat, wide slightly broader than half of foot width. Pair of secondary cephalic muscles (sm) with middle and anterior regions approximately double thickness and half width those of columellar main bundle (left muscle slightly broader than right one); originating gradually along dorsal surface of main columellar bundle, running anteriorly on main bundle, inserting in ventral region of perioral region. Pair of tentacular/ommatophore muscles originating between posterior and middle thirds of main columellar muscle bundle, between both secondary cephalic muscles; gradually thickening and bifurcating towards anterior; strong bifurcation halfway between their detected origin and anterior end, dorso-lateral branches becoming short and strong ommatophore muscles (ou), inserting near tip of ommatophore (om); ventro-medial branches running towards anterior, dorsally to secondary cephalic muscles, halfway newly bifurcating, lateral branch becoming short tentacular muscles (tm), inserting in tip of tentacles (te); medial branch running further towards anterior, inserting dorsally to insertion of secondary cephalic muscles. Pedal gland large, curved, protruding in ventral region of buccal area (Fig. 21C: pg).

**Mantle organs.** (Fig. 21A) Mantle border thick, lacking pigments. Pneumostome (pn) protected by simple right ventral flap (pv), width ~1/5 of aperture length. Dorsal fold well developed (mf), occupying ~1/3 of dorsal mantle edge length; left end pointed, projected beyond its base. Pneumostome (pn) ~1/10 of shell aperture length, bearing exclusively air entrance and urinary gutter. Anus (an) separate aperture located at right, adjacent to pneumostome. Lung of 1.5 whorls in length, wide and elongated; right side ~1.5 times longer than left side. Pulmonary vessels conspicuous, but low; 6-7 stronger vessels perpendicularly disposed at right; 6-7 main vessels at left radially disposed; all pulmonary vessels visible, but low, i.e., weakly protruded. Pulmonary vein (cv) running longitudinally across pallial cavity roof medially or towards right, anterior end

trifurcated. Reno-pericardial area beige, slightly triangular, located posteriorly within pallial cavity, its posterior abutting wall of visceral cavity, occupying ~30% of cavity length and ~40% of its width (details below). Rectum (rt) wide. Urinary gutter (ur) narrow, running along rectum; anterior urinary gutter surrounding left anal aperture.

**Visceral mass.** (Fig. 21C) ~3 whorls in length. Both digestive gland lobes brown in color; anterior lobe (da) flattened, occupying ~1/5 of visceral volume, located just posteriorly to pallial cavity, continuous to kidney. Posterior lobe (dg) larger, extending 2 spiral whorls, occupying ~60% of visceral volume. Stomach ~1/15 of visceral volume, located between both digestive gland lobes, about 3/4 whorl posterior to pallial cavity (st). Digestive tubes (described below) surrounding anterior lobe of digestive gland. Gonad multi-lobed, cream color, encased between posterior lobe of digestive gland and columella, occupying ~1/3 whorl, ~1/10 of visceral volume.

**Circulatory and excretory systems.** (Fig. 21A) Pericardium (pc) ~twice as long as wide, located obliquely between middle and left thirds of posterior end of pallial roof, appressed against right lateral side of kidney; occupying ~5% of lung area. Auricle (au) located anteriorly, as continuation from pulmonary vein (cv); ventricle (ve) located posteriorly, larger. Kidney (ki) simple, dorso-ventrally flattened; size reported above; somewhat triangular, width ~2/3 of length; internally organized as two distinct regions – a longitudinal hollow cavity on left; filled by sponge-like renal tissue on right ~2/3 from posterior to anterior ends, being gradually broader towards anterior. Nephropore (ne) small, longitudinal slit at anterior-left corner of kidney, directed towards right; protected by curved anterior projection of kidney (kf). Urinary gutter (ur) composed of small folds perpendicular to rectum axis, flanking almost entire rectum left edge, starting at short distance from pulmonary postero-right end, ending in pneumostome as narrow urinary groove flanking left edge of anus (an).

**Digestive system.** (Figs. 21C–23B) Oral tube wide, muscular (Figs. 21E: mj). Radula features (Fig. 22) below. Jaw simple, thick, dark brown, exposed portion horseshoe-shaped, thicker at middle (Fig. 20J); surface having only coaxial low undulations and growth lines; located ai anterior end of buccal dorsal chamber (Fig. 23B: jw). Buccal mass spherical, occupying~1/5 of haemocoel volume (Fig. 21C: bm). Dorsal surface of oral cavity with well-developed pair of dorsal folds (Fig. 23B: df), width of each ~1/3 of dorsal wall width; separated from each other by dorsal chamber (dc) as wide as folds, but broader anteriorly. Odontophore with ~60% of buccal mass volume (Figs 21E, 23B: od). Odontophore muscles (Figs 21E–23B): **mj**, jaw and peribuccal muscles originating in outer-ventral surface of odontophore cartilages (Fig. 23A: mj), running towards dorsal, splaying in dorsal wall of oral tube (Fig. 21E: mj); **m1**, jugal muscles covering entirely haemocoelic structures, more concentrated close to mouth; **m1a**, small pair of lateral muscles, origin in latero-ventral region of haemocoel, running dorsally short distance, inserting in lateral surface of odontophore; **m1l**, small pair of lateral protractor muscles, origin in lateral region of buccal area, running towards posterior along 2/3 buccal mass length, inserting in lateral region of odontophore, anterior to m1a; **m1v**, small pair of ventral protractors jugal muscles, originating in ventral surface of haemocoel close to mouth, running towards posterior, inserting in ventral-posterior region of odontophore close to m2 insertion (Fig. 21E: m1v); **m2**, strong pair of retractor muscles of buccal mass, or radular muscles, originating as single bundle in columellar muscle posterior end (Fig. 21B: m2), running anteriorly close to median line along ~60% of haemocoel length, inserting as two different bundles in ventro-posterior edge of odontophore, surrounding at some distance radular nucleus (Fig. 23A: m2); **m3d**, thin layer of longitudinal fibers immersed in dorso-posterior wall of odontophore, preceding esophageal origin (Fig. 21E); **m3p**, thin layer of dorso-ventral fibers covering posterior odontophore wall, ventral to esophagus (Fig. 21E); **m4**, main pair of dorsal tensor muscles of radula, thick, originating in postero-medial region of odontophore cartilages (Fig. 23A: m4), surrounding outside and medially cartilages, inserting in subradular membrane in its region correspondent to buccal cavity; **m5**, pair of thick

auxiliary dorsal tensor muscles of radula, originating on posterior-medial surface of m4 and short portion in postero-ventral region of odontophore cartilages (Fig. 23A: m5), , running towards median line covering m4, inserting in subradular membrane by side of m4 insertion; **m6**, horizontal muscle absent (Fig. 23A); **m7**, absent; **m10**, pair of narrow ventral odontophore protractor muscles, originating in ventro-anterior region of haemocoel, just ventral to mouth, running towards posterior along buccal mass length (Fig. 21E: m10), inserting in latero-posterior surface of odontophore close to m2 insertions; **m11**, pair of narrow ventral tensor muscles of radula absent. Odontophore non-muscular structures (Figs. 23A-B): **oc**, pair of odontophore cartilages flattened, rather elliptical, anterior region slightly projected anteriorly close to median line, ~1.2 times longer than wide, fused with each other along ~half in their anterior-medial edge (Fig. 23A: oc), posterior end roughly rounded; **sc**, subradular cartilage, with expanding region in buccal cavity protecting subradular membrane. Radular sac short, not extending beyond odontophore.

**Radula.** (Fig. 22) Slightly longer than odontophore; with rachidian teeth, and ~45 pairs of lateral/marginal teeth; no clear distinction between lateral and marginal teeth (Fig. 22A), marginal teeth only slightly narrower than lateral teeth, and more inclined medially (Fig. 22C: right side); all teeth with base as long as mesocone, articulating neighboring rows (Figs. 22D, F); mesocone arched, curved inwards and posterior, ~twice longer than wide, apex bluntly pointed; no secondary cusps. **Rachidian** tooth (Figs. 22E-F: arrow) as large as lateral teeth; base ~twice longer than wide, slightly flattened, barely triangular, with arched posterior edge articulating with neighbor tooth and pair of distal reinforcements; mesocone ~half of base, symmetrical, located slightly dislocated posteriorly from adjacent lateral teeth row (Figs. 22E-F). **Lateral teeth** similar to rachidian (Figs. 22B, D), except in being slightly longer, asymmetrical, arched towards medial region; base trapezoid, with medial concavity articulating with neighbor medial tooth and single distal reinforcement (Fig. 22F). **Marginal teeth** starting with no clear boundary with lateral teeth (Figs. 22A, C) occupying ~1/3 of each side; shaped similarly to lateral teeth, except for being slightly narrower and more inclined medially (Fig. 22C).

Salivary glands covering esophagus in its region preceding its anterior quarter (Fig. 21C: sg), forming two elongated, white, thin masses. Each salivary duct differentiable in middle and anterior side of glands, with ~1/12 of esophageal width (Figs. 21C, E, 4B: sd). Salivary duct running in both sides of esophageal origin, penetrating buccal mass wall in region close to buccal ganglia (Figs. 21E, 23B: sd), running immersed in buccal dorsal wall along ~1/3 its length (Fig. 23B: sd). Salivary ducts opening as small slits in middle level of dorsal folds, on their lateral side (Fig. 23B: sa).

Esophagus ~1-whorl long, with thin, flaccid walls (Fig. 21C: es); anterior 1/3 clearly broader, crop-like; posterior 2/3 narrower; inner surface simple, with 5–10 simple longitudinal folds. Stomach (Fig. 21C: st) narrow, curved, almost not bulging; position and size described above (visceral mass); gastric walls relatively thick muscular (Fig. 21D: sw); inner surface mostly smooth (Fig. 21D), except for narrow longitudinal fold along smaller curvature (sf), connecting esophageal duct to digestive gland with intestinal origin; series of low, right folds flanking anterior side of longitudinal fold. Esophageal insertion on right side, intestinal origin on left side, both close to columella. Duct to anterior lobe of digestive gland at short distance from esophagus and intestine intersection (Figs. 21C, D: dr) wide, running shortly towards right, bifurcating; additional left duct (Fig. 21C: dl) originated in main duct base with ~1/3 of its width, running narrowly towards left and anterior along digestive gland lobe compressed by first intestinal loop (da), periodically possessing short ramifications along its length. Duct to posterior lobe of digestive gland located short distance from intestinal origin, posterior to above-described duct, directed towards opposite side (Figs. 21C, D: dd) slightly wider than anterior duct. Intestine (in) initially as wide as esophageal insertion, gradually broadening up to ~double that width along its

wide sigmoid loop in anterior lobe of digestive gland (Fig. 21C: in). Rectum and anus position described above (pallial cavity) (Fig. 21A: rt, an). Anus sessile, as slit in right end of mantle edge directly turned outside; inner surface with 8–10 simple longitudinal, relatively tall folds.

**Reproductive system.** (Fig. 23C-24B) Gonad position described above (visceral mass), composed of 8–10 lobes with minute digitiform acini (Fig. 21C: go). Hermaphroditic duct (Fig. 23C: hd) narrow and weakly coiled in posterior half, abruptly becoming wider (up to 3-times wider), more intensely coiled in anterior half, coiling diminishing only close to insertion (Fig. 24D: hd). Seminal receptacle (Figs. 23C, D: sr) large, oval, balloon-like sac, ~twice longer than wide, ~three times wider than hermaphroditic duct. Fertilization complex simple, located at narrow and elongated base of seminal receptacle (Fig. 23D) as duct of seminal receptacle; slightly shorter than length of receptacle. Fertilization complex totally immersed in albumen gland, inserting in posterior end of spermoviduct, by side of albumen gland duct, relatively wide, twice wider than receptacle's duct. Albumen gland (Fig. 23C, D: ag) solid, white, elliptical, more-or-less same size as gonad (~1/3 whorl). Albumen gland duct subterminal, connected to distal end of spermoviduct (Fig. 23D: ad), in lateral, large albumen chamber (Fig. 23D: ac); widely connected to distal end of spermoviduct (Fig. 23D: eo). Spermoviduct (eo) of ~1.5 whorl in length, slightly narrower than albumen gland, ~10 times longer than wide. Prostate gland occupying ~3/4 of spermoviduct surface and ~1/2 its volume (Figs. 23C, E: pt). Uterus occupying ~3/4 of spermoviduct space, external walls thick-glandular (Figs. 23C, E: ut), inner surface mostly covered by wide oblique folds (Fig. 23D: ut). Sperm groove entirely composed of two tall folds (Figs. 23D, E: sp), becoming vas deferens some distance posterior to genital pore (~1/15 of remaining spermoviduct length) (Figs. 23C, F: vd), with ~1/10 of anterior spermoviduct width, running outside of penis shield (Fig. 23C). Vagina ~1/20 spermoviduct length (Figs. 23C, F: vg); inner surface simple, with 8-10 longitudinal, low, wide folds (Fig. 23F: vg). Bursa copulatrix almost as long as spermoviduct length; bursa duct ~half of adjacent spermoviduct width in its origin, gradually narrowing towards posterior end (Fig. 23C: bd); bursa oval, ~1/3 of albumen gland size (Fig. 23C: bc), located encased between pericardium and adjacent intestinal loop. Penis ~3/4 of spermoviduct length, ~2/3 its anterior width (Fig. 23C: pe); penis muscle inserting terminally, short, broad (Figs. 23C, 24A: pm), with no branch on epiphallus. Epiphallus ~1/7 penis' length, tip rounded, located laterally (Fig. 24A: eh) inner surface with smooth, widely opening to penis posterior chamber (Fig. 24B: eh). Vas deferens inserted subterminally in penis tip, in intersection with epiphallus (Figs. 24A, B: vd). Internal penial surface with two clear chambers (Fig. 24B), strong transverse septum dividing penis in about similar-sized posterior (ps) and anterior (pa) chambers; transverse septum having strong sphincter in edge (sh), and proximal blunt projection bulging in a side of anterior chamber (pf); this transverse septum swelling middle portion of penis externally (Figs. 23C, 24A); posterior penis chamber with wide furrow running from opposite side of epiphallus aperture, up to region close to transverse septum (Fig. 24B: ps); anterior penis chamber with strong, wide longitudinal fold located at base of blunt projection (pf), tapering towards anterior; low, wide longitudinal folds covering entire surface of anterior penis chamber (pa). Genital pore with radial arrangement of muscles, with pair of more developed muscles (Fig. 23C: gm) running dorsally and posteriorly, connecting with middle region of columellar muscle.

**Central nervous system.** (Fig. 24C) Nerve ring located across buccal mass (Fig. 21C: nr). Pair of cerebral ganglia (ce) almost fused; cerebral commissure slightly narrower than ganglia; each ganglion about as wide as adjacent esophageal section; several wide nerves originating in cerebral antero-lateral region. Cerebral node or gland (cd) located in postero-medial quadrant, with ~1/8 each ganglion's size. On each side, parallel, rather narrow connectives (cn) running between cerebral ganglion and ventrally located fused pedal and pleural ganglia (pp), accompanied by blood vessels (bv) issuing from anteriorly-directed aorta. At least six pairs of nerves originating from anterior side of pedal-visceral ganglion complex. Pair of statocysts not seen.

**Distribution.** Minas Gerais region around Parque Nacional Cavernas do Peruaçu, so far from Itacarambi (north) to São João da Ponte (south).

**Habitat.** Limestone outcrops, Cerrado forest, 466-759 m altitude.

**Measurements.** (length and width in mm) holotype MZSP 151900 (Figs. 20A-C): 44.2 by 23.5 (Figs. 20A-C); paratypes MZSP 151901#1 (Fig. 20D): 42.8 by 20.7; #2: 43.0 by 24.4 (Fig. 20G); MZSP 151893: 47.1 by 25.3 (Fig. 20F).

**Material examined.** Types (reported above). BRAZIL. **Minas Gerais** (W.V. Matos col); Itacarambi, Vargem Grande, 15°00'50"S 44°04'34"W, 466 m altitude, MZSP 151869, 3 shells (7.ii.2020); São João da Ponte, near Olímpio Campos, 15°50'44"S 44°00'03"W, 759 m altitude, MZSP 151811, 1 shell (i.2020).

**Taxonomic remarks.** See Discussion item.

## Discussion

15. The taxon descriptions presented above include comparative remarks, which shows the detailed differences amongst the taxa herein studied. This is particularly evidenced in given diagnoses. The present section is, thus, more focused on the most interesting features and those more relevant to taxonomy and present/future phylogenetic approaches to resolving relationships among South American strophocheilids.

### Generic debate

As reported above, *Anthinus* appears to encompass the smallest strophocheilids [6], and those with a slightly "Bulimulus"-like, bulimoid shell, i.e., a fusiform shell with a relatively small aperture. These features easily differentiate the genus from the remaining cofamiliar genera that are bigger and with proportionally larger aperture. *Catracca*, introduced here, actually breaks this rule, as it is similar to *Anthinus* in these features, so much so that samples were initially identified as belong to that genus. Despite the *Catracca* shell lacking the colorful pattern of *Anthinus*, the discovery of *A. vailanti*, which also possesses a rather monochromatic shell, affects the generic separation only based on conchology. The anatomical features were
16. fundamental to determine both as separate genera.

The set of characters reported here in diagnoses of both genera are sufficient to individualize them amongst the strophocheilids. However, it is recognized that the remaining genera do not have the same level of details known. Despite Leme [5] having redefined all strophocheilid (and megalobulimid) genera, including their anatomy, the level of details was not the same as those reported herein. Contrary to Leme's [5] definition of *Anthinus*, some characteristics were not confirmed in the present study. In particular, the pair of colored bands of the antero-dorsal region of the animal were not found. Further, all examined *Anthinus* species possessed the genital accessory gland (e.g., Figs. 4E, 10D, 14F: as) that was considered absent, and being an exclusive feature of the megalobulimines. Other characteristics reported in Leme's [5] diagnosis are accurate, such as the weakly muscular stomach lacking inner folds (e.g., Fig. 6B), the curvature of the seminal receptacle (e.g., Figs. 8C, 24B: sr) (which is also shared with other strophocheilines), and the shape of the epiphallus (e.g., Fig. 4C: eh). Other interesting characteristics that now can be added to the *Anthinus* diagnosis include the low profile of the pulmonary vessels, usual for species of smaller size, dark pigmented (e.g., Fig. 13L); the double origin of the odontophore muscle pair m5 (e.g., Fig. 6D); the posterior insertion of the anterior (esophageal) duct to the digestive gland, inserted practically into the stomach (e.g., Fig. 6B: dr), its bifurcation, with both components of similar caliber (e.g., Fig. 6C: dr, dl); the presence of the

pallial, pre-rectal muscle (another supposed exclusive character of the megalobulimines); and the penis inner arrangement of folds forming a pair of stronger posterior folds, the furrow between which is continuous with the epiphallus aperture.

The new genus *Catracca* shares some characteristics with *Anthinus*, distinguishing both from other strophocheilids, such as the relatively small shell aperture; the small but distinct umbilicus (Figs. 20A, D); the configuration of the robust head musculature (Fig. 21B); the posterior position of the anterior duct of the digestive gland (Fig. 21C, despite the left branch, dl, is much narrower); and of the nerve ring (Fig. 24C). However, *Catracca* has a set of exclusive characters that justify its introduction, reported in its diagnosis above, some commented below. In some aspects, the *Catracca* protoconch resembles those of *Megalobulimus* Miller, 1878 in being blunt, wide and with uniform axial sculpture (Figs. 20E, H, I). It differs, however, in being much more elongated and of smaller absolute size. The other strophocheilid genera have the protoconch with delicate reticulated sculpture and much more acuminate [4, 6]; *Catracca* actually has the bluntest perspective, with a proportionally widest protoconch amongst the strophocheilines, producing a somewhat mamillated shell. From the anatomical diagnostic characters of *Catracca*, which particularly distinguish it from *Anthinus*, the more interesting are the thick muscular stomach (Fig. 21D: sw), which in muscular development is inferior only to the gizzard-like stomach of *Megalobulimus* [5]; the odontophore pair m5 entirely originated from the pair m4; the sac-like, non-curved seminal receptacle (Fig. 23D: sr); the absence of accessory genital gland in the spermoviduct (Fig. 23E); and by the pair of vaginal folds (Fig. 23F: vf). However, the more outstanding distinctive feature of *Catracca* is its inner penial arrangement (Fig. 24B). The large transverse septum with well-developed sphincter in its edge (sh) is unique amongst the strophocheilids. A structure approaching this was only found only in *Mirinaba* Morretes, 1955, particularly in *M. planidens* (Michelin, 1831) ([5]: fig. 54). However, in the latter, the transverse septum is much lower, thicker, and lacks the sphincter. Besides, the *Mirinaba* copulatory organ has an elongated epiphallus, more similar to those of *Megalobulimus*. The epiphallus in *Catracca* is most similar to that of *Anthinus*, short and rounded (Fig. 23A: eh); it differs, however, in lacking inner papillae cover (e.g., Fig. 14C, 20C: eh). Moreover, in *Catracca* the vas deferens inserts in the border between the epiphallus and penis (Figs. 23A-B), while in *Anthinus* the vas deferens inserts in the middle level of the outer epiphallus wall (e.g., Fig. 8E: vd). The penis muscle insertion is restricted to the penis wall in *Catracca* (Fig. 23A), while some branches of it insert on the epiphallus in *Anthinus* (e.g., Figs. 8B, 17C: pm).

Interestingly, in *Anthinus*, the radula of the type species *A. multicolor* looks more like
17. that of *Catracca uhlei* than those of remaining studied congeners. ~~Both,~~ *A. multicolor* and *C. uhlei* share short, blunt mesocones, particularly those of the rachidian teeth (Figs. 3C, 22F: arrow), while the mesocone of the remaining *Anthinus* species are much more elongated, flattened, spatula-like, with the rachidian more similar to lateral. The radulae of both genera differ from those of the megalobulimines in lacking broad mesocones, of almost the same size as the bases. The radulae of the other strophocheilid genera are not known.

The presence of supposedly megalobulimine characteristics in *Anthinus*, such as the pre-rectal pallial muscle (e.g., Fig. 17A: mp) and the accessory genital gland (e.g., Fig. 17D: as); as well as the megalobulimine supposed exclusive characters in *Catracca*, such as the protoconch (Fig. 20E) and the sac-like seminal receptacle (Fig. 23D: sr) are of difficult interpretation in respect to generic interrelationships. In the provisional phylogenetic treatment below, the accessory genital gland resulted in a convergence between megalobulimines and *Anthinus*. The same result (convergence) was obtained for the regular narrow axial cords in the protoconch shared between *Catracca* and some megalobulimines. The sac-like seminar receptable, on the other hand, appears to be plesiomorphic in the family.

**Specific debate**

A gallery of shells of all known *Anthinus* species are assembled in Figs. 25A–H, 26A–H, based on representatives (mostly holotypes – D–H), as well as of *Catracca uhlei* (Fig. 25I, 26I holotype). In that, the most outstanding feature is the monochromia of *A. vailanti* (Fig. 25F, 26F) and of *C. uhlei*, which already sets them apart from the remaining that possess characteristic beige-brown spots or blotches forming weakly-defined, spiral interrupted bands, i.e., pale spots or blotches on the beige-brown background. However, some specimens of *A. vailanti* possess some evidence of such banding (Figs. 9H-I). After that, the present debate focuses on *Anthinus* species, considering that the diagnosis of each species already brings the set of characters exclusive of each species that supported their description. The present discussion focuses in the characters, every numerical parameter is based on a N from 10 to 20 specimens and is an integer value. Related to the shell elongation, *A. multicolor* is the slenderer (width/length W/L index = 46%) (Figs. 1A–C, 25C), with a group orbiting W/L ~50%, such as *A. synchondrus* (Fig. 25E – 54%), *A. albolabiatus* (Fig. 25A – 49%), *A. miersi* (Fig. 25B – 51%) and *A turnix* (Fig. 25D – 55%); while another group has shells broader, encompassing species with W/L = 60%, such as *A. vailanti*, *A. morenus* (Fig. 25G) and *A. savanicus* (Fig. 25H). In respect to the spire angulation, *A. synchondrus* has the sharpest pointed spire, ~35°, followed by *A. multicolor* ~40°, and *A. albolabiatus* ~42°; *A, turnix* has the broader spire, with ~58°, followed by *A. miersi* ~55°; while the remaining species (*A. morenus*, *A. vailanti* and *A. savanicus*) have ~50°. The number of protoconch whorls is relatively constant amongst the species, with ~3 whorls; however, that of *A. synchondrus* looks slightly longer, with 3.2 whorls (Figs. 5K, L); while those of *A. multicolor* and *A. turnix* look shorter, with ~2.5 whorls, followed by *A. savanicus* with ~2.8 whorls (Fig. 16F). The protoconch absolute size, in mm, is also an important parameter, as usually is relatively constant within each species; the largest protoconch is that of *C. uhlei*, with 9 mm of diameter; it is followed by that of *A. vailanti*, with 8.8 mm; in the range of ~7 mm there are *A. savanicus* – 7.7 mm, *A. albolabiatus* – 7.5 mm, and *A. synchondrus* and *A. morenus* with 7.0 mm; the range of ~6 mm starts with *A. turnix* – 6.8 mm. *A. miersi* – 6.3 mm, ending with the smallest protoconch of *A. multicolor* – 6.1 mm. In relation to the aperture, the amplest is that of *A. turnix*, with aperture length occupying 60% if shell length, and 67% of shell width; it is followed by *A. miersi* – 58%-60% respectively; the smallest proportional peristome is that of *C. uhlei* – 45%-50%, followed by *A. synchondrus* – 48%-50% and *A. savanicus* – 48%-51%; the remaining species are in the range of peristome size about half of the shell's size, as follows: *A. morenus* – 51%-51%, *A. albolabiatus* – 52%-54%, *A. multicolor* – 52%-55%, and *A. vailanti* – 55%-60%. The shell's absolute size is also an interesting parameter, with *A. miersi* being the larger, with an average of 53 mm, followed by *A. turnix* – 50 mm; the smallest species is *A. vailanti*, with average size of 30 mm, followed by *A. savanicus* – 32 mm, and *A. morenus* – *A. multicolor*, both with 35 mm; *A. synchondrus* – 40 mm, and *A. albolabiatus* – 45 mm, are intermediary. The average size of *C. uhlei* is 45 mm.

Regarded to *Anthinus multicolor*, a more detailed discussion on the loss of the types, geographic distribution, and shell variation, is found elsewhere [4]. In the original description [29], Rang reported that the examined material came from Brazil, particularly Saint-Paul province (the State of São Paulo), and from near Corcovado, which today is a place inside the city of Rio de Janeiro, State of Rio de Janeiro. Bequaert [4] reported the species as occurring in these two states (Rio de Janeiro and São Paulo), and figured a topotype ([4]: pl. 4, figs. 3, 7; MCZ 74441, from Corcovado), which is similar to the original illustration ([29]: pl 3, fig. 1). The specimen has irregular dark spots in its shell, lacking apparent spiral alignment as usually have the other species (Fig. 25). The shell illustrated by Simone [6] (Fig. 25C) was collected in Teresópolis, located close to Serra dos Órgãos, contiguous with the Corcovado rainforest. It also shows the same spot patterns of the previous illustrations [4, 29]. The presently studied sample were collected in São Fidélis, a place ~80km far from Corcovado, but also apparently in the same biome, as it is close

to the Desengano State Park. The shells of the presently studied sample (Figs. 1A-C) also have the irregular, not spirally aligned spots, but they possess a wide spiral clear band along the middle level of the last whorl. This band is not present in all specimens of that lot, and, thus, is interpreted as variation. The non-alignment of the dark spots is, thus, an interesting species' distinctive character.

Related to anatomy, beyond the species described herein, there is some information only on *A. miersi* (radula, pallial roof and genitalia [7]) and the generic statement by Leme [5]. The anatomical discussion on the other species is, thus, precluded, and even on *A. miersi* the details are not equivalent. The mantle edge of the studied *Anthinus* has the usual pattern of most pulmonates, which includes a small left fold from the pneumostome (e.g., Fig. 21A: mf, which is pointed to the left); this fold has shown relatively constant in each species and of taxonomical value, in *A. synchondrus* and in *A. savanicus*, it has an elongated projection (Figs. 6A, 16H: mf); it is pointed in *A. vailanti*, and simply rounded in *A. morenus* and *A. multicolor* (Figs. 13L, 22A). The pallial cavity is interestingly darkly pigmented in all species (e.g., Fig. 134H: pu), in which the pulmonary vessels are difficult to discern. The different pattern of lung vascularization is evident in the drawings represented for each species. Furthermore, the relative size of the reno-pericardial area varies: *C. uhlei* and *A. vailanti* (Fig. 21A), considered of medium size (~1/4-1/5 of total cavity area); while *A. synchondrus* (Fig. 6A) and *A. savanicus* (Fig. 16H) can be considered large, occupying ~1/3 of lung area; and *A. morenus* (Fig. 13L), and *A. multicolor* (Fig. 2A), can be considered small (about 1/5 of lung area). The pallial pre-rectal muscle, as referred above, is supposedly a megalobulimine characteristic [5], but found in all here studied *Anthinus* examined here (Figs. 6C, 17A: mp) except for *A. multicolor* (Fig. 2A). In the digestive system, the jaw plate shows interesting comparative data; it is deeper and broad in *C. uhlei* (Fig. 20J), *A. vailanti* (Figs. 9K-L), *A. savanicus* (Fig. 16G), *A. multicolor* (Figs. 1G-H), and possibly in *A. miersi* [7]; however, it is shallow and wide in *A. synchondrus* (Fig. 5M), and *A. morenus* (Fig. 13I); moreover, a ventral additional medial fold reinforces the jaw plate exclusively in *A. vailanti* (Fig. 9L), and the jaw lacks a median anterior projection or beak only in that of *A. morenus* (Fig. 13I). Related to the odontophore intrinsic and extrinsic musculature, they have shown lightly similar; the most variable pairs are the m1, m3 and m7, which can be modified, present or absent according to the species (see descriptions), however, the variation found in pair m5 appears to be more interesting; it is 100% originated from m4 in *C. uhlei*, but it is ~50% originated from m4 and 50% from cartilages in *A. synchondrus*, *A. savanicus* and *A. multicolor*, it is ~60% originated from m4 in *A. morenus*, and ~20% in *A vailanti* (Fig. 10A). The degree of odontophore cartilages fusion along the ventral-inner edge is also of taxonomic interest, both cartilages are ~50% fused in *C. uhlei* (Fig. 23A) and *A. morenus* (Fig. 14B); ~90% fused in *A. vailanti* (Fig. 10A) and *A. multicolor*; ~85% fused in *A. savanicus* (Fig. 17B); while the cartilages are entirely fused in *A. synchondrus* (Fig. 6D). The radular teeth's mesocone is short, compared to those of the other genera of the family, in *C. uhlei* (Fig. 22), *A. multicolor* (Fig. 23), and *A. miersi* [7]; however, it is elongated, spatula-like in *A. synchondrus* (Fig. 7), *A. vailanti* (Fig. 11), *A. morenus* (Fig. 15), and *A. savanicus* (Fig. 18). This mesocone elongation looks something exclusive of this subgroup of *Anthinus* amongst the known strophocheilids. An additional character is easily differentiating the rachidian tooth and its neighbors; the species with short mesocone, the differentiation between both is easy, while those with elongated mesocone, the rachidian is very similar to the neighboring teeth, being of difficult differentiation; from those, *A. synchondrus* and *A. vailanti* with certain effort it is possible to point out which is the rachidian column; however, this is very difficult in *A. morenus* and *A. savanicus*. Another interesting feature of the examined species is the posterior position of the anterior duct to the digestive gland, called the esophageal duct, which connects to the anterior lobe of the digestive gland. This esophageal duct originates practically from the stomach, while at least in *Megalobulimus*, it is far from the stomach; this feature is not known in the other

strophocheilid genera. Beyond the odd position of the esophageal duct to digestive gland, there is the strong bifurcation of it, i.e., it bifurcates shortly after its origin. This characteristic is found both, in *Catracca* and in *Anthinus*, being only different in size; both branches have relatively similar caliber in *Anthinus* (e.g., Fig. 6C: dr, dl), while the left branch is narrower in *Catracca*; this does not occur at least in *Megalobulimus*, but this is unknown in other related genera.

The genital system usually is very informative in heterobranchs, but it has shown relatively conservative in strophocheilids, differing only in small details reported below. The presence of the supposedly exclusive megalobulimine accessory genital gland in spermoviduct (e.g., Figs. 8D, F: as) of the *Anthinus* is extraordinary. The seminal receptacle offers some distinction of the examined species, being balloon-like in *Catracca*, as well in megalobulimines [5], and strongly curved in *Anthinus*; however, the seminal receptacle is amply, widely curved in *A. vailanti* (Fig. 10B: sr) and *A. multicolor* (Fig. 4B); while it is tightly curved in *A. synchondrus* (Fig. 8C), *A. morenus* (Fig. 14E), and *A. savanicus* (Fig. 19A). The insertion of the hermaphrodite duct (hd) in the seminal receptacle is in its base in all studied species, except for *A. vailanti*, in which it is inserted in the middle of its concave edge; this insertion is strongly curved in *C. uhlei*, *A. savanicus* and *A. multicolor*, while it is straight, rather perpendicular in *A. synchondrus*, *A. vailanti* and *A. morenus*. The penis relative size is ~80% of the spermoviduct in *A. savanicus* (Fig. 17C: pe), ~60% in *A. synchondrus* (Fig. 8B) and *A. vailanti* (Fig. 10C), and ~50% in *C. uhlei* (Fig. 23C), *A. morenus* (Fig. 14D), *A. multicolor* (Fig. 2C), and *A. miersi* [7]. The accessory genital gland in the spermoviduct is relatively small in *A savanicus* (Fig. 4B: as) and *A. multicolor*, while it is large in the other three species. The number and form of the spermgrooves in the spermoviduct is also variable according to the species; they are double and similar-sized in *C. uhlei* (Fig. 23E: sp) and *A. multicolor* (Fig. 4E), they are also double, but asymmetrical in *A. synchondrus* (Fig. 8F), it is single and with a spiral fold in *A. vailanti* (Fig. 10D), they are double but of different thickness in *A. morenus* (Fig. 14F), they are double, but one of them is T-shaped in *A. savanicus* (Fig. 17D). In the vaginal-free oviduct cavity, *C. uhlei* has a double vaginal fold (Fig. 4F: vf), while *A. multicolor* has a hump (Fig. 4C: hp); the internal pattern of folds is different in each species, and are schematized in their respective drawings (Figs. 4A, 8D, 12A, 19B). The insertion of the penis muscle shows also interesting distinctions, it is only inserted in the penis wall in *C. uhlei* (Fig. 24A: pm), it has two small branches additionally connected to epiphallus in *A. synchondrus* (Fig. 8B), and *A. vailanti* (Fig. 10C), it has a larger component connected to epiphallus in *A. morenus* (Fig. 14D), it has a single and broad branch to epiphallus in *A. savanicus* (Fig. 17C), and it has a small branch connected to epiphallus and another to vas deferens in *A. multicolor* (Figs. 4C-D). As told above, all *Anthinus* have a papillae cover in epiphallus lumen, but the papillae are small and uniform in *A. synchondrus* (Fig. 8E), of medium size in *A. vailanti* (Fig. 12B), and tall in *A. morenus* (Fig. 14C), *A. savanicus* (Fig. 19C) and *A. multicolor* (Fig. 4D); additionally, the papillae extend further along the penial chamber in *A. multicolor,* and along an adjacent area in *A. vailanti*; besides, the papillae of *A. multicolor* are not uniform. The internal pattern of penial folds is also distinctive, *A. morenus* is the only species in having a single and simple large posterior fold (Fig. 14C: pf); all remaining *Anthinus* have a pair of parallel folds in a side: *A. synchondrus* has these folds composed of a series of transverse threads (Fig. 8E); *A. vailanti* is the only in having this pair fused in the proximal region (Fig. 12B); *A. savanicus* has this pair formed by the end of the secondary folds of the remaining penial inner surface (Fig. 19C); while *A. multicolor* has these folds wide and covered by papillae (Fig. 4D). Interestingly, this pair of folds has its intermediary furrow continuous with the aperture of the epiphallus. All these internal structures are exteriorized through copulation, including the complex structure of *Catracca* (Fig. 24B). To understand how they work during copula is a fascinating issue for future investigation.

**Anatomical and broader taxonomical debate**

As referred above, details of the some remaining strophocheilid genera are still unknown, and what is known is mostly focused in megalobulimines. This precludes further inferences on the intrinsic and extrinsic interrelationships and phylogeny of the taxa. Leme (e.g., [5, 10, 22]) had that as a main professional goal, but it remains unfinished. The intention is to find a character or a set of characters supporting each taxon, which, based on lack of studies, so far is impossible. Megalobuliminae, firstly introduced as a family [5] really looks like a subdivision of Strophocheilidae, possessing the largest species (regarded as an anti-predatory strategy [30]), and supported at least by the pulmonary septum, buccal flange, pre-rectal septum in intestine, pre-rectal pallial muscle and accessory genital gland in the spermoviduct [5]. However, the presence of these two last characteristics in *Anthinus*, here presented, shows that these concepts must be reevaluated. Within strophocheilids, only *Megalobulimus* (so far the single genus of Megalobuliminae, with almost a hundred species), and *Anthinus* (this study) have samples with anatomical description sufficiently detailed for supporting more detailed comparative analyses like phylogeny. The other six genera [5, 6] are totally unknown or only have some few structures known.

The central nervous system (nerve ring) has shown relatively conservative in both genera studied here, all of them are similar (e.g., Fig. 24C), varying only in some small details and proportions, as reported above. At least in its main features, the nerve ring of the studied species is very similar to that of the megalobulimines [10] and even of the Bulimulidae (e.g., [31]).

A phenotypical base to understand the relationship of the Strophocheilidae with the other related families is also difficult for the same reason – lack of anatomical knowledge. At the time Strophocheilidae was only closely related to Dorcasiidae (African) and Acavidae (Asian) within Acavoidea [9], a group that apparently lays large eggs, produced by a thick walled, glandular spermoviduct, the phenotypic analysis was difficult. The scenario became more complicated with the present conception of these families as part of Rhytidoidea, with five additional families, based on molecular approaches [11, 12, 32]. The plan is to perform studies on other genera of all these families in a similar level of details of the present one, to produce a morphological database enough for a phylogenetic analysis. Only then a confrontation of the present taxonomy will be possible, and, possibly, to provide morphological bases for all subdivisions. The traditional Acavoidea above mentioned looks to be a morphologically well-supported taxon by above mentioned characters. However, the present Rhytidoidea still needs a better phenotypic support, as the fusion of Acavoidea with Rhytidoidea [11] is only based upon two papers with incongruent results, one of them did not have the Acavoidea as a focus [32], and another is an unpublished dissertation [33].

**Phylogenetic analysis**

Once more it is important to emphasize that the present phylogenetic analysis is not concerned with resolving the family Strophocheilidae, nor any other higher taxa. The main intention, using already published data, is to demonstrate that a new genus was necessary to be introduced, and the species does not fit in any of the known genera.

The computational processing of the Matrix (Appendix 2), which was based on the given list of 56 characters/126 states (Appendix 1) (see Material and Methods and [13]), resulted in a single cladogram (Fig. 28) with the following indices: length (L)= 283; consistency index (CI)= 68; retention index (RI)= 89.

In the cladogram (Fig. 28), the position of *Catracca uhlei* (after node 3) clearly shows that it does not belong to any known genera. It is recognized that some strophocheilid genera are not represented in the cladogram, but all of them (i.e., *Gonyostomus* Beck, 1837; *Speironepion*

Bequaert, 1948; and the Andean *Austroborus* Parodiz, 1949 and *Chiliborus* Pilsbry, 1926) are beforehand reasonably different. The isolated position of *Catracca* in the base of Strophocheilidae (node 3), with two autapomorphies (character 7: *Megalobulimus oblongus*-like protoconch; character 52: transverse septum in the penis) is compatible with the generic separation.

Additionally, some few provisional conclusions are possible. The genus *Anthinus* resulted monophyletic (node 7), supported by six synapomorphies, such as the mosaic shell color (character 4); special arrangement of head muscles (ch. 11); the spatula-like radular mesocone (ch. 29), with rounded tip (ch. 30); the loss of the talon (ch. 42), and of the spermoviduct accessory gland (ch. 44). *Anthinus multicolor*, the type species, resulted as first branch, with two autapomorphies, and separated from the other congeners by three autapomorphies. The other four *Anthinus* species introduced herein resulted in successive branches, with *A. vailanti* at base, and *A. synchondrus–A. morenus* together as terminals.

Other interesting provisional results are the monophyly of the South American Strophocheilidae (node 2 – 14 synapomorphies), separated from the African Dorcasiidae (node 15 – 3 synapomorphies). The dichotomy of the family into Megalobuliminae (node 11 – 15 synapomorphies) and Strophocheilinae (node 3 – 5 synapomorphies) is also corroborated. Also noticeable is the division of the megalobulimines, in which what Leme [5] informally called as "*M. ovatus* group" (node 14 – 1 synapomorphy) and *"M. oblongus* group (node 12 – 2 synapomorphies). These possibly can be an indicative of genetic separation, an issue that is in analysis.

The present provisional cladogram achieved its goals, mainly concerned to *Catracca* allocation, as well as shows that *Anthinus* is monophyletic and has a perceptible internal arrangement that can arouse future subgeneric divisions. The increment of this cladogram with more taxa is an in-progress project.

**Ecologic-distribution information**

The discovery of several new taxa in central regions of Brazil has indicated how weak the knowledge level on the Brazilian malacofauna is. With human activities, possibly species have been lost even before they were at least known. Several Brazilian land snails have shown to be endemic from small areas (personal observations), which is an important factor for conservation efforts and politics. The taxa described in this paper are examples that lots of Brazilian species and even genera still need to be discovered or better-known. Knowing the fauna is the first step to raise protective rules to them and their environments. Thus, their descriptions are relatively urgent.

The type localities of all *Anthinus* and *Catracca* are shown in the map of Fig. 27. In that it is possible to see that there is a concentration in the northwest region of Minas Gerais (MG) (and adjacent region of Goiás – GO), and another in Rio de Janeiro (RJ). The single type locality far from those is that of *A. albolabiatus*, in east of Rio Grande do Sul (RS). Reports to *Anthinus* in literature and collections are not common, but samples and citations of *Anthinus* have been collected from south of Minas Gerais to North Argentina and Uruguay, sometimes even identified at a specific level. As the areas in such the *Anthinus* samples were better studied – Rio de Janeiro and NW Minas Gerais (Fig. 27) show that several species with restricted distribution occur in relatively small areas, the question arises: are the *Anthinus* from the other regions belonging to undescribed species? As in the moment there is no possibility of checking this, no answer is possible.

## Acknowledgements

A special thanks to José Coltro Jr and his team of collectors for the collect and donation of most studied samples of the present paper. To Lee Kremer for the thoughtful correction of the text. To Gary M. Barker and the other two anonymous referees, and the Editor for the comments, criticisms and improvements. To Lara Guimarães, MZSP, for the assistance in SEM examinations. The collects were performed under the IBAMA-Sisbio license 10560-2.

## References

1. Albers JC. Die Heliceen, nach natürlicher Verwandtschaft. Verlag von Th.Chr.Fr. Enslin. Berlin, 262 pp; 1850.
2. Rang S. Descriptions des coquilles terrestres recueillies pedant un voyage à la côte occidentale d'Afrique, et au Brésil. Ann Sci Natur. 1831; 24: 1-63, pls. 1-3.
3. Martens Ev. Albers Die Heliceen, nach natürlicher Verwandtschaft (second edition). Verlag von Wilhelm Engelmann. Leipzig, 359 pp; 1860.
4. Bequaert JC. Monograph o the Strophocheilidae, a Neotropical family of terrestrial mollusks. Bull Mus Comp Zool. 1948, 100(1): 1–210, 32 pls.
5. Leme JLM. Anatomy and systematics of the Neotropical Strophocheiloidea (Gastropoda, Pulmonata) with the description of a new family. Arq Zool. 1973; 23(5): 295–337.
6. Simone LRL. Land and freshwater Molluscs of Brazil. São Paulo, EGB/ FAPESP; 2006.
7. Oliveira MP, Almeida EL, Vieira I, Oliveira MH. Comunicação n° 3: sobre o *Gonyostomus* multicolor miersii (Sowerby). Trab Setor Malac Univ Fed Juiz de Fora. 1968: 7-20.
8. Salvador RB, Colley E, Simone LRL. Terrestrial mollusks from the region of Corumbá and Maciço de Urucum, SW Brazil. J Conch. 2018; 43(1): 71–88.
9. Fontenelle JH, Miranda MS. Aspects of biology of *Megalobulimus paranaguensis* (Gastropoda, Acavoidea) in the coastal plain of the Brazilian southeast. Iheringia sér Zool. 2017; 107: e2017004; DOI: 10.1590/1678-4766e2017004.
10. Simone LRL, Leme JLM. Two new species of Megalobulimidae (Gastropoda, Strophocheiloidea) from north São Paulo, Brazil. Iheringia sér Zool. 1998; 85: 189-203.
11. Bouchet P, Rocroi JP, Hausdorf B, Kaim A, Kano Y, Nützel A, Parkhaev P, Schrödl M, Strong EE. Revised classification, nomenclator and typification of gastropod and monoplacophoran families. Malacologia. 2017; 61(1-2): 1–526.
12. MolluscaBase eds. MolluscaBase. Rhytidoidea Pilsbry, 1893. Website accessed at: http://www.molluscabase.org/aphia.php?p=taxdetails&id=890459 on 2021-02-10; 2021.
13. Simone LRL. Phylogeny of the Caenogastropoda (Mollusca), based on comparative morphology. Arq Zool. 2011; 42: 161–323.
14. Leme JLM, Castro RL, Indrusiak LF. Contribuição anatômica e histológica para o conhecimento de *Mirinaba antoninensis* (Morretes, 1952) (Gastropoda, Strophocheilidae). Pap Avul Zool. 1979; 32(14): 183–191.
15. Indrusiak LF, Leme JLM. Anatomia comparada de três espécies de *Mirinaba* Morretes, 1952 (Gastropoda, Strophocheilidae) do Estado do Paraná, Brasil. Acta Biol Paranaense 1985; 14: 163–180.
16. Leme JLM. *Megalobulimus lopesi* sp.n., uma nova espécie de Pulmonata terrestre da mata atlântica brasileira (Mollusca, Gastropoda, Megalobulimidae). Mem Inst Oswaldo Cruz 1989; 84(suppl. 4): 313–318.
17. Leme JLM, Indrusiak LF. *Megalobulimus parafragilior*, sp.n., uma nova espécie de Pulmonata terrestre da Serra do Mar (Gastropoda, Megalobulimidae). Pap Avul Zool 1990; 37(5): 97–105.

18. Leme JLM, Indrusiak F. Anatomia e considerações sobre *Megalobulimus proclivis* (Martens, 1888) (Gastropoda, Megalobulimidae). Iheringia (Zool) 1995; 78: 19–27.
19. Fontenelle JH, Simone LRL, Cavallari DC. *Megalobulimus dryades*, a new species from the Atlantic Forest in southeastern Brazil, and redescription of *Megalobulimus gummatus* (Gastropoda: Strophocheilidae). Pap Avul Zool 2021; 61: e20216144. Doi 10.11606/1807-0205/2021.61.44.
20. Silva FS, Simone LRL. Anatomy of two species of *Megalobulimus* (Strophocheilidae, Megalobuliminae) from Center-South of Bahia, Brazil. Pap Avul Zool (in press).
21. Simone LRL, Salvador RB. Taxonomical study on a sample of land snails from Nanuque (Minas Gerais, Brazil), with descriptions of three new species. Stutt Beit Naturk A 2016; N Ser 9: 9–30.
22. Leme JLM. Sobre a posição taxonômica de *Dorcasia* e *Trigonephrus* (Gastropoda, Pulmonata). Pap Avul Zool. 1980; 34(2): 11–19.
23. Simone LRL. Bosquejos de filogenia. Clube de Autores. Curitiba, 60 pp. https://www.google.com.br/books/edition/Bosquejos_De_Filogenia/b5TtDwAAQBAJ?hl=pt-BR&gbpv=0
24. Pilsbry HA. American bulimi and bulimuli – *Strophocheilus*, *Plekocheilus*, Auris, *Bulimulus*. Man Conch.1895-1896; (2)10: 1-213, 51 pls.
25. Morretes FL. Ensaio de catálogo dos moluscos do Brasil. Arq Mus Paran. 1949; 7: 1–216.
26. Abbott RT. Compendium of landshells. American Malacologists. Hong Kong, 240 pp; 1989
27. Salgado NC, Coelho ACS. Moluscos terrestres do Brasil (gastrópodes operculados ou não, exclusive Veronicellidae, Miladicae e Limacidae). IN Barrientos, Z & Monge-Nájera, J. [ed.] Malacología Latinamericana. Rev Biol Trop. 2003; 51(suppl.3): 149–189.
28. Oliveira MP, Rezende GJR, Castro GA. Catálogo dos moluscos da Universidade Federal de Juiz de Fora. Sinonímia de família, gênero e espécie. MEC, UFJF. Juiz de Fora, 520 pp, 1981.
29. Rang S. Description des coquilles terrestres recucillies pendand un voyage à la côte occidentale d'Afrique, et au Brésil. Ann Sci Nat. 1831: 24: 5–63.
30. Simone LRL. Physical defense strategies of South American land snails. Malacopedia. 2018; 1: 3–11. http://www.moluscos.org/trabalhos/Malacopedia/01-02%20Simone%202018%20Physical%20strategies%20of%20defense.pdf
31. Simone LRL, Belz CE, Gernet MV. A new species of *Drymaeus* endemic from Currais Archipelago, Paraná, Brazil (Pulmonata, Bulimulidae). Pap Avul Zool. 2020; 60: e20206057. DOI 10.11606/1807-0205/2020.60.57.
32. Herbert DG, Moussalli A, Griffiths OL. Rhytididae (Eupulmonata) in Madagascar: reality or conjecture? J Moll Stud. 2015; 81(2): 259–268
33. Teasdele LC. Phylogenomics of the pulmonate land snails. PhD Thesis, University of Melbourne. 199 pp. http://hdl.handle.net/11343/128240; 2017.

Legends

**Fig 1. *Anthinus multicolor*, shell and some anatomical structures, sample MZSP 152925**. (A-C) shell of specimen #2 (L 30.2 mm), frontal, right and dorsal views. (D) same, detail of apex, apical-slightly dorsal views. (E) same, protoconch and part of first teleoconch whorl in profile. (F) penis, inner surface, detail of middle and distal thirds showing papillae. (G) jaw, ventral view. (H) same, anterior view. Scales= 1 mm.

**Fig 2. *Anthinus multicolor*, anatomy**. (A) pallial cavity, partially uncoiled visceral mass and digestive system, mostly ventral view, inner lip of pneumostome sectioned and deflected upwards. (B) head-foot, dorsal view, most of dorsal integument and inner organs removed, main

concern to retractile/columelar musculature, some right-anterior structures sectioned. (C) genital system, mostly dorsal view. Scales = 3 mm.

**Fig 3.** ***Anthinus multicolor*****, radula in SEM**, MZSP 152925. (A) wide view, scale= 200 µm; (B) detail of central region, scale= 50 µm; (C) same, higher magnification, scale= 30 µm. White arrows indicating rachidian column.

**Fig 4.** ***Anthinus multicolor*****, anatomy**. (A) anterior half of genital tubes, opened longitudinally. (B) carrefour region of genitalia and posterior region of spermoviduct, albumen gland seen as transparent. (C) penis, ventral view. (D) same, opened longitudinally showing inner surface. (E) spermoviduct, transverse section in its middle region. Scales= 1 mm.

**Fig 5.** ***Anthinus synchondrus*****, shell of types and some anatomical photos**. **(A-F)** holotype MZSP 152074 shell (L 36.9 mm). (A) frontal view. (B) right view. (C) dorsal view. (D) anterior-slightly ventral view. (E) apical view (W 19.7 mm). (F) frontal view after extraction of specimen by an artificial hole. (G-H) paratype MZSP 152236, (L 40.8 mm), frontal and dorsal views. (I-J) 2 paratypes MZSP 152252 shell, frontal views (L 36.4, 36.2 mm). (K) holotype, detail of protoconch and part of first teleoconch whorl, profile. (L) same, detail of apical region. (M) jaw of holotype in situ, ventral view. (N) detail of right-anterior corner of pallial roof of holotype, ventral view, distal portion of rectum sectioned longitudinally. Scales= 1 mm.

**Fig 6.** ***Anthinus synchondrus*****, anatomy**. (A) pallial cavity roof, ventral view, pneumostome ventral lip sectioned and deflected upwards, portion of pallial floor also shown. (B) stomach opened longitudinally, ventral view. (C) digestive tubes mostly as in situ, ventral view, topology of some adjacent structures also shown. (D), odontophore, dorsal view, superficial layer of muscles and structures removed, left muscles deflected, radula deflected to left. Scales= 2 mm.

**Fig 7.** ***Anthinus synchondrus*****, radula in SEM**, holotype MZSP 152074. (A) wider view, scale= 200 µm; (B) detail of central region, scale= 200 µm; (C) same, higher magnification, scale= 50 µm. White arrows indicating rachidian column.

**Fig 8.** ***Anthinus synchondrus*****, anatomy**. (A) foregut opened longitudinally, ventral view, odontophore deflected to right. (B) genital system, mostly dorsal view. (C) same, detail of carrefour, ventral view, seen if albumen gland was transparent. (D) genital system, anterior end opened longitudinally, penis only partially shown. (E) penis and epiphallus opened longitudinally. (F) spermoviduct, transverse section in its middle level. Scales = 2 mm.

**Fig 9.** ***Anthinus vailanti*****, shells and jaw**. (A-E) holotype MZSP 152891 (L 29.0 mm). (A) frontal view. (B) right view. (C) dorsal view. (D) detail of apex, right-slightly apical view. (E) protoconch and adjacent teleoconch whorl, right-slightly apical view. (F-G) paratype MZSP 152188 (L 27.9 mm) frontal and dorsal views. (H-J) paratype MZSP 152244 (L 29.5). (H) frontal view. (I) dorsal view. (J) anterior-slightly left view showing umbilicus. (K) holotype's jaw plate, ventral view. (L) same, anterior view. Scales= 0.5 mm

**Fig 10.** ***Anthinus vailanti*****, anatomy**. (A) odontophore, dorsal view, superficial layer of muscles and structures removed, most muscles deflected, radular ribbon deflected to left. (B) middle region of genital system isolated, only topology of albumen gland shown. (C) genital system, mostly ventral view. (D) spermoviduct, transverse section in its middle level. Scales= 2 mm.

**Fig 11.** ***Anthinus vailanti*****, radulae in SEM**, Paratypes MZSP 152118, 2 specimens. (A) wider vision, scale= 200 µm; (B) detail of central region, scale= 50 µm; (C) wide left view, scale= 100 µm; (D) wide view of another specimen, scale= 200 µm; (E) detail of central region, scale= 50 µm; (F) wide left view, scale= 200 µm. White arrows indicating rachidian column.

**Figs 12. *Anthinus vailanti*, anatomy**. (A) genital system, detail of anterior region opened longitudinally, penis and other adjacent structures only partially shown. (B) penis and epiphallus opened longitudinally. Scales= 2 mm.

**Fig 13. *Anthinus morenus*, shell of some types and anatomy**. (A-H) Holotype MZSP 152891 (L 35.8 mm). (A) Frontal view. (B) Right view. (C) Dorsal view. (D) Anterior view (W 21.0 mm). (E) Apical view. (F) Detail of protoconch and fist teleoconch whorl, profile. (G) Same, higher magnification. (H) Same, detail of apex. (I-L) Paratype MZSP 152047#1. (I) Jaw, in situ, ventral view. (J) Shell, frontal view (L 34.8 mm). (K) Shell, dorsal view. (L) Pallial cavity, ventral view, dorsal part of mantle edge and deflected to right, part of mantle floor also shown. Scales= 2 mm.

**Fig 14. *Anthinus morenus*, anatomy**. (A) Head-foot, head, digestive and genital structures removed, dorsal integument removed, main concern to main head-foot musculature. (B) Odontophore, dorsal view, superficial layer of muscles and structures removed, most muscles deflected. (C) Penis, sectioned longitudinally to show inner surface. (D) Genital system, mostly ventral view. (E) Middle region of genital system isolated, only topology of albumen gland shown. (F) Spermoviduct, transverse section in its middle level. Scales= 2 mm.

**Fig 15. *Anthinus morenus*, Radula in SEM**, paratype MZSP 152047. (A) wide view, scale= 200 µm; (B) detail of central region, scale= 200 µm; (C) detail of damaged region, with teeth in profile, scale= 50 µm; (D) detail of right region, scale= 50 µm; (E) wide view, scale= 500 µm; (F) detail of left region, scale= 200 µm. White arrows indicating rachidian column.

**Fig 16. *Anthinus savanicus*, shell of types and anatomical details**. (A-H) holotype MZSP 154391 (L 32.5 mm). (A) shell, frontal view. (B) right view. (C) dorsal view. (D), apical view. (E) inferior (umbilical) view. (F) protoconch and part of first teleoconch, profile, scale= 2 mm. (G) jaw, ventral view (L 1.7 mm). (H) almost entire specimen, mostly dorsal view, pallial cavity removed and deflected right (ventral view), head-foot deflected below, visceral mass slightly uncoiled, scale= 5 mm. (I-J) paratype MZSP 153897 (L 31.5 mm), frontal and dorsal views. (K) paratype MZSP 152171 (L 35.3 mm), frontal view.

**Fig 17. *Anthinus savanicus*, anatomical drawings**. (A) midgut as in situ, ventral view, some adjacent structures or their topology also shown, stomach and esophagus opened longitudinally. (B) odontophore, dorsal view, outer layer of structures removed, pair of odontophore cartilages deflected, both m4 and left m5 (right in Fig.) also deflected. (C) genital structures, mostly ventral view, most vas deferens (dv) detached. (D) spermoviduct, transverse section of its middle level. Scales= 2 mm.

**Fig 18. *Anthinus savanicus*, radulae in SEM**. (A) holotype MZSP 154391. Detail of central region, scale= 50 µm; (B) same, detail of column in profile, scale= 50 µm; (C) same, detail of right region, scale= 200 µm; (D) paratype MZSP 153897, detail of right region, scale= 50 µm; (E) same, central region, scale= 100 µm. White arrows indicating rachidian column.

**Fig 19. *Anthinus savanicus*, anatomy, genital system**. (A) carrefour region, ventral view, seen if albumen gland (ag) was transparent, spermoviduct (eo) transversely sectioned in its posterior region; (B) anterior end, ventral view, entirely opened longitudinally, portions of adjacent structures also shown. (C) penis, ventral view, entirely opened longitudinally, portions of adjacent structures also shown. Scales= 2 mm.

**Fig 20. *Catracca uhlei*, shells and jaw**. (A-C) Holotype MZSP 151900 (L 44.2 mm). (A) Frontal view. (B) right view. (C) dorsal view. (D) Paratype MZSP 151901#1 (L 42.8 mm), frontal view; (E) holotype protoconch in profile, scale= 3 mm. (F) paratype 151893, shell of one of dissected specimens, frontal view (L 47.1 mm). (G) Paratype MZSP 151901#2 (L 43.0 mm).

(H) Paratype MZSP 151871, young specimen, detail of apex, profile-slightly apical view, scale= 2 mm. (I) Same, apical view, scale= 2 mm. (J) Jaw plate, ventral view (slightly broken in left end, right in Fig.), scale= 1 mm.

**Fig 21. *Catracca uhlei*, anatomy**. (A) Pallial cavity roof, ventral view, pneumostome ventral lip sectioned and deflected upwards. (B) Head-foot, head, digestive and genital structures removed, anterior integument sectioned and deflected to left (right in Fig.), main concern to main head-foot musculature. (C) Fore- and midgut, mostly ventral view, shown as in situ, topology of some adjacent structures also shown. (D) Stomach, ventral view, mostly sectioned longitudinally to show inner surface. (E) Buccal mass, left view. Scales= 5 mm.

**Fig 22. *Catracca uhlei*, radula in SEM**, holotype MZSP 151893. (A) wide view, scale= 300 µm; (B) detail of central region, scale= 100 µm; (C) detail of lateral region, scale= 100 µm; (D) detail of central region, scale= 50 µm; (E) same, higher magnification, scale= 30 µm; (F) same, higher magnification, scale= 10 µm. White arrows indicating rachidian column.

**Fig 23. *Catracca uhlei*, anatomy**. (A) Odontophore, dorsal view, superficial layer of muscles and structures removed, left muscles deflected. (B) Buccal mass, ventral view, sectioned longitudinally, odontophore deflected to left. (C) Genital system, mostly ventral view. (D) Middle region of genital system isolated. (E) Spermoviduct, transverse section in its middle level. (F) Anterior region of genital system, ventral view, female portion sectioned longitudinally to show inner surface. Scales= 2 mm.

**Fig 24. *Catracca uhlei*, anatomy**. (A) Penis, dorsal view. (B) Same, sectioned longitudinally to show inner surface. (C) Nerve ring, dorsal view. Scales= 2 mm.

**Fig. 25. Gallery of shells of all *Anthinus* species**, frontal and dorsal views. (A). *A. albolabiatus*, MZSP 7958 (L 50 mm). (B) *A. miersi*, MZSP 7963 (L 53 mm). (C) *A. multicolor*, MZSP 29620 (L 26 mm). (D) *A. turnix*, holotype USNM 712805 (L 50 mm). (E) *A. synchondrus* holotype MZSP 152074 (L 36.9 mm); (F) *A. vailanti* holotype MZSP 152891 (L 29.0 mm). (G) *A. morenus* holotype MZSP 152891 (L 35.8 mm). (H) *A. savanicus* holotype MZSP 154391 (L 32.5 mm). (I) *Catracca uhlei* holotype MZSP 151900 (L 44.2 mm). A to D extracted from Simone (2006).

**Fig. 26. Gallery of shells of all *Anthinus* species in same scale**, frontal views. (A). *A. albolabiatus*, MZSP 7958. (B) *A. miersi*, MZSP 7963. (C) *A. multicolor*, MZSP 29620. (D) *A. turnix*, holotype USNM 712805. (E) *A. synchondrus* holotype MZSP 152074. (F) *A. vailanti* holotype MZSP 152891. (G) *A. morenus* holotype MZSP 152891. (H) *A. savanicus* holotype MZSP 154391. (I) *Catracca uhlei* holotype MZSP. A to D extracted from Simone (2006). Scale= 10 mm. – verificar citação

**Fig. 27.** Brazil map with states outlined and type localities of each *Anthinus* (*A.*) and *Catracca* shown. New localities in dark blue. Previous localities in light blue. States abbreviations: GO: Goiás; MG: Minas Gerais; RJ: Rio de Janeiro; RS: Rio Grande do Sul.

**Fig. 28.** Single cladogram obtained analyzing the matrix (Appendix 2) of the species assembly reported in Material and Methods (written black), plus the species studied herein (written brown). Both branches below are far outgroups, a bulimulid (*Leiostracus*) and a simpulopsid (*Rhinus*). Branch *15* as close outgroups, two African dorcasiids. Each branch with synapomorphies shown. Each symbol as in the box; above number the character, below number the state. Branch numbers for discussion in blue italics. Method: parsimony; L= 283; CI= 68; RI= 89.

**Appendix 1 – list of characters that base the phylogenetic analysis**

1. Shell walls: 0= thin; 1= thick.
2. Shell form: 0= bulimoid; 1= discoid.
3. Shell size: 0= 2-3 cm; 1= 4-5 cm; 2= over 6 cm.
4. Shell color: 0= monochromatic; 1= mosaic of spots.
5. Periostracum: 0= permanent; 1= deciduous.
6. Aperture: 0= smaller than half of shell length; 1= longer than half of shell length.
7. Protoconch sculpture: 0= smooth or reticulate; 1= reticulate with axial predominance; 2= irregular axial threads; 3 regular narrow axial cords.
8. Protoconch form: 0= pointed; 1= blunt.
9. Head peri-oral flange: 0= absent; 1= present.
10. Haemocoel layers of head rectractor muscles: 0= two; 1= single.
11. Inner layer of head retractors: 0= only present in ommatophores; 1= ommatophores and peribuccals separated; 2= ommatophore and peribuccal fused.
12. Mantle edge inner pneumostome fold: 0= simple; 1- angulous.
13. Differentiated vessel close to pneumostome: 0= absent; 1= present.
14. Afferent pallial pulmonary vessels: 0= single; 1= a pair.
15. Pulmonary septum: 0= absent; 1= present.
16. Nephropore: 0= protected in primary ureter; 1= exposed in pallial cavity.
17. Nephropore: 0= simple; 1= protected by anterior renal fold (? in outgroup).
18. Kidney: 0= smooth; 1= covered by anastomosed net of vessels.
19. Urinary pallial way: 0=via duct; 1= via groove.
20. Ureter groove: 0= smooth; 1= transversely folded.
21. Jaw plate: 0= smooth; 1= transversely folded; 2: narrow.
22. Horizontal muscle (m6): 0= present; 1= absent.
23. Insertion of pair m5: 0= on pair m4; 1= partially on cartilages.
24. Pair of muscles inside radular sac (m7): 0= present; 1= absent.
25. Inner edge of cartilages (anterior to fusion): 0= thick; 1= thin.
26. Radular teeth: 0= rachidial similar to lateral; 1= different.
27. Radular teeth: 0= bi- or multicuspid; 1= unicuspid.
28. Radular mesocone: 0= smaller than base; 1= as long as base; 2= longer than base.
29. Radular mesocone: 0= stubby; 1= flattened, spatula-like.
30. Radular mesocone tip: 0= pointed; 1= rounded.
31. Tissue on radula at end of radular sac (to): 0= simple; 1=reinforced.
32. Salivary duct between salivary gland and buccal mass: 0= long; 1= short.
33. Esophageal duct to anterior lobe of digestive gland: 0= far from stomach; 1= close to stomach.
34. Esophageal duct to anterior lobe of digestive gland: 0= single; 1= closely bifid
35. Stomach: 0= non-muscular; 1= weakly muscular; 2= highly muscular; 3= high muscular connected to typhlosole (add).
36. Stomach inner folds in its smaller curve: 0= absent; 1= present; 2= extending to remaining stomach (add).
37. Pre-rectal valve: 0= absent; 1= present.
38. Pre-rectal pallial muscle: 0= absent; 1= present.
39. Anus: 0= turned outside; 1= opening inside pallial cavity.
40. Seminal receptacle: 0= balloon-like; 1= U-shaped.
41. U-shaped seminal receptacle: 0= widely curved; 1- tightly curved.

42. Talon (swollen end of hermaphroditic duct) in carrefour; 0= absent; 1= present.
43. Duct of albumen gland: 0= connected in spermoviduct; 1= connected directly to albumen chamber.
44. Spermoviduct accessory gland: 0= absent; 1= small; 2= large.
45. Prostate: 0= narrow (~1/3 of diameter); 1= wide (~1/2).
46. Vaginal appendix: 0= absent; 1= present.
47. Epiphallus: 0= conic, long; 1= short, rounded; 2= pseudo-epiphallus; 3= conic, short.
48. Flagellum: 0= absent; 1= single and small.
49. Penis shield: 0= present; 1= absent.
50. Penis retractor muscle: 0= simple; 1= multiple.
51. Penis retractor muscle insertion: 0= in penis tip only; 1= branches in epiphallus.
52. Penis with transverse fold ("penial papilla"): 0= absent; 1= present, simple; 2= with sphincter.
53. Pair of parallel inner folds connecting epiphallus and penis: 0= absent; 1= present.
54. Penis inner surface: 0= smooth; 1= papillate; 2= with large longitudinal fold in a side (pilar).
55. Spawn: 0= minute and numerous capsules; 1= few, large capsules.
56. Capsules cover: 0= thin, weakly calcified; 1= thick, well-calcified (egg like).

# Appendix 2 – matrix of characters that base the phylogenetic analysis

| **Species\ character** | **1** | **2** | **3** | **4** | **5** | **5** |
|---|---|---|---|---|---|---|
| | **1234567890** | **1234567890** | **1234567890** | **1234567890** | **1234567890** | **123456** |
| *Mirinaba antoninensis* | 1010011000 | 1100011010 | 0111101100 | 0111110001 | ?100001011 | 111111 |
| *Mirinaba cadeadensis* | 1010011000 | 1100011010 | 0111101100 | 0111110001 | ?100001011 | 111111 |
| *Megalobulimus lopesi* | 1020002111 | 0010110111 | 1101011100 | 1100221110 | ?102003010 | 000211 |
| *Megalobulimus grandis* | 1020012111 | 0010110111 | 1101011100 | 1100221110 | ?102001010 | 100211 |
| *Megalobulimus parafragilior* | 1020012111 | 0010110111 | 1101011100 | 1100221110 | ?102001010 | 100211 |
| *Megalobulimus proclivis* | 1020012111 | 0010110111 | 1101001100 | 1100321110 | ?002011010 | 000211 |
| *Megalobulimus oblongus* | 1020103111 | 0010110111 | 1101001100 | 1100321110 | ?002110110 | 000211 |
| *Megalobulimus conicus* | 1020103111 | 0010110111 | 1101001100 | 1100321110 | ?002110110 | 000211 |
| *Megalobulimus driades* | 1020003111 | 0010110111 | 1101001100 | 1100321110 | ?002110110 | 000211 |
| *Strophocheilus debilis* | 1010002000 | 0100011010 | 0111101100 | 0111110000 | ?101002010 | 010111 |
| *Catracca uhlei* | 1010003100 | 1100011011 | 0101001100 | 0111110000 | ?000002010 | 020111 |
| *Anthinus multicolor* | 1001001000 | 2000011010 | 0111101111 | 0111110001 | 0002001010 | 101111 |
| *Anthinus synchondrus* | 1011001000 | 2100011010 | 2111101211 | 0111110101 | 1012001010 | 101111 |
| *Anthinus vailanti* | 1010001000 | 2100011010 | 0111101211 | 0111110101 | 1002001010 | 101111 |
| *Anthinus morenus* | 1011001000 | 2100011010 | 2111101211 | 0111110101 | 1012001010 | 101111 |
| *Anthinus savanicus* | 1011001000 | 2100011010 | 0111101211 | 0111110101 | 1012001010 | 101111 |
| *Dorcasia akexandri* | 0100000000 | 0001011010 | 000?000000 | 0000010000 | ?002000000 | 000011 |
| *Trigonephrus porphyrostoma* | 0100?00000 | 0001011010 | 0????00000 | 0000010000 | ?0?2000000 | 000011 |
| *Leiostracus carnavalescus* | 0000000000 | 0000000000 | 0000000000 | 0000000000 | ?000000000 | 000000 |
| *Rhinus botocudus* | 0000000000 | 0000000000 | 0000000000 | 0000000000 | ?000000000 | 000000 |

Figure 01

Click here to access/download;Figure;01b Anthinus multicolor 00.jpg

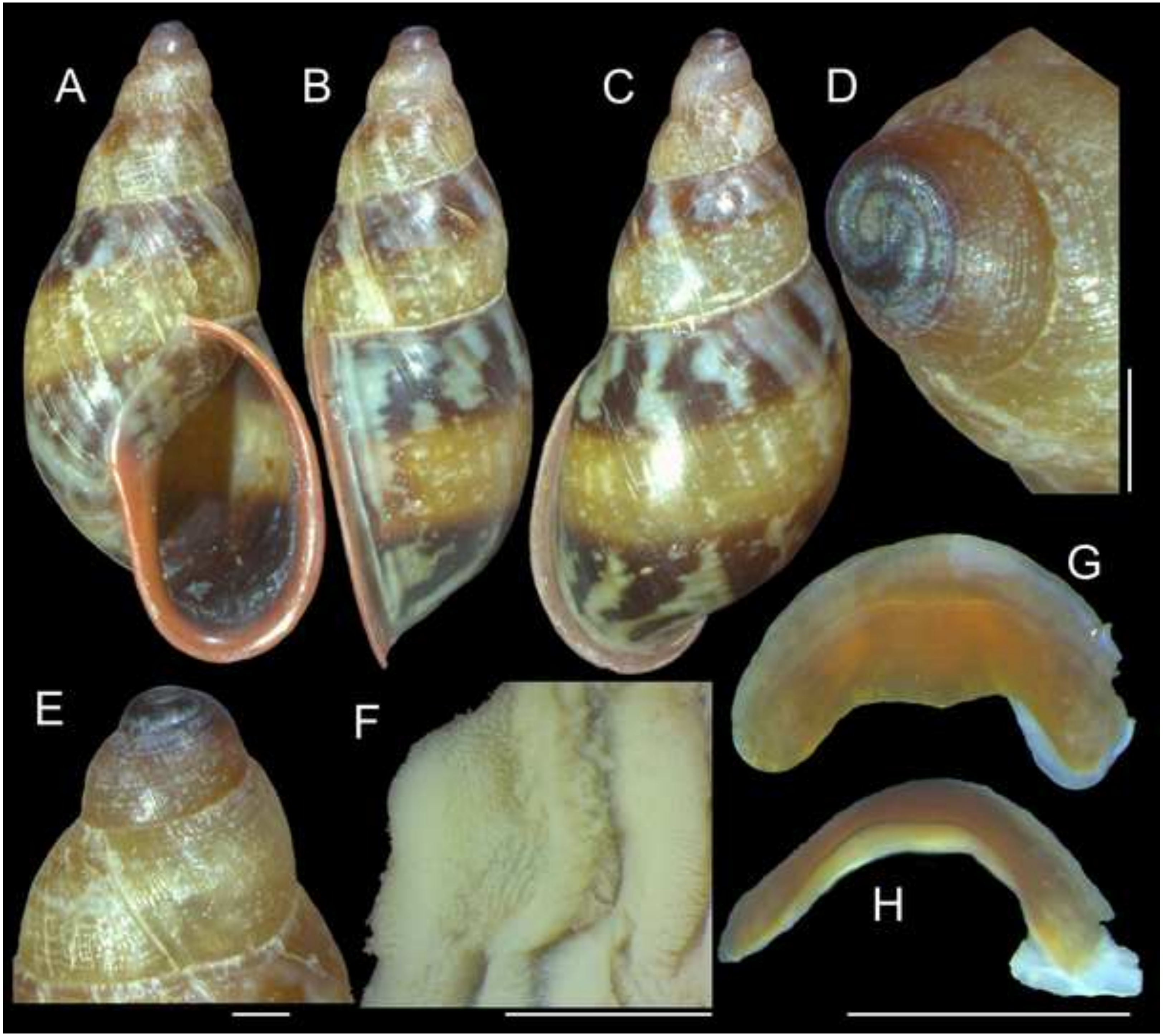

Figure 02

Click here to access/download;Figure;02b Anthinus multicolor 01.jpg

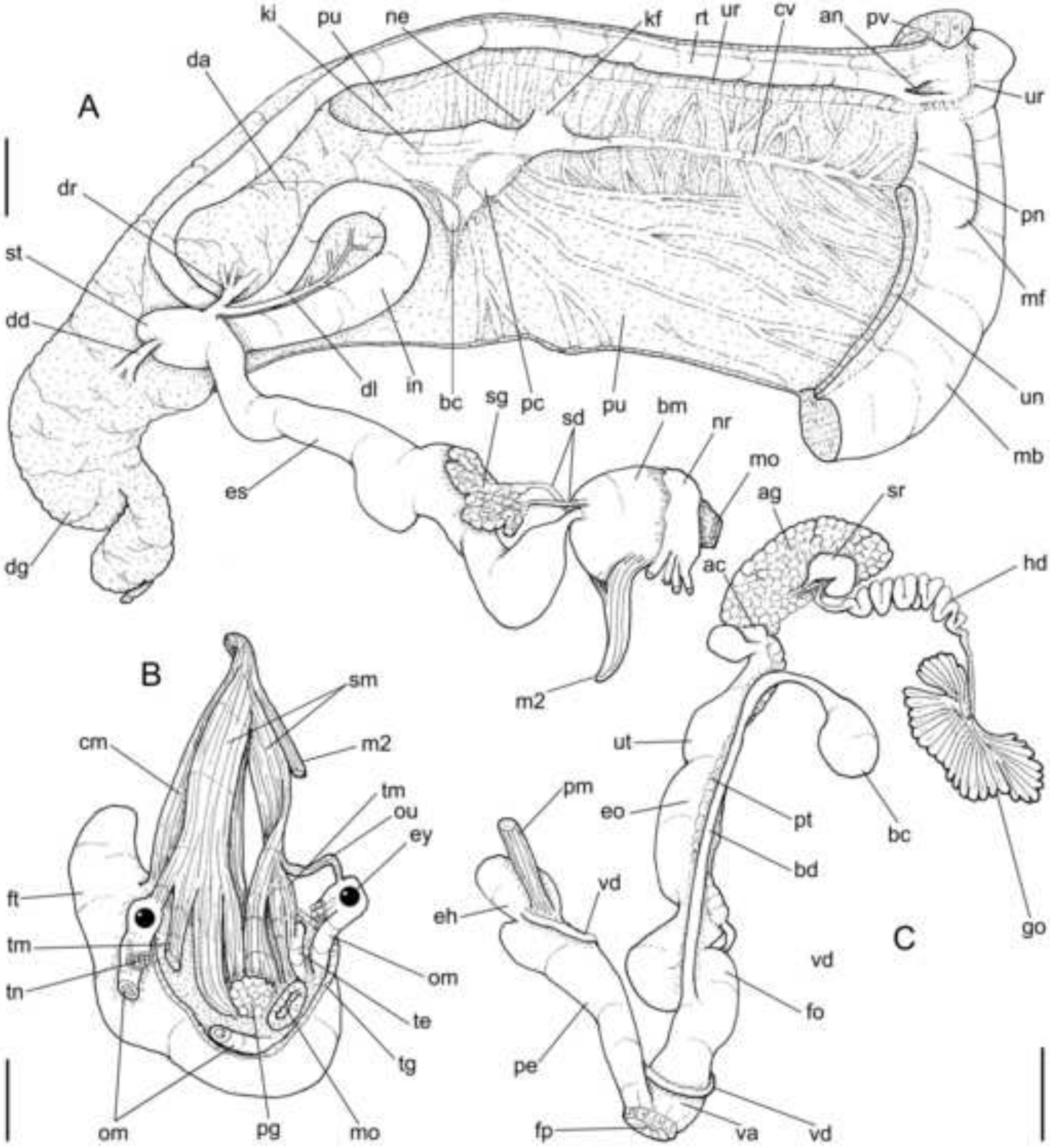

Figure 03

Click here to access/download;Figure;03b Anthinus multicolor Rad.jpg

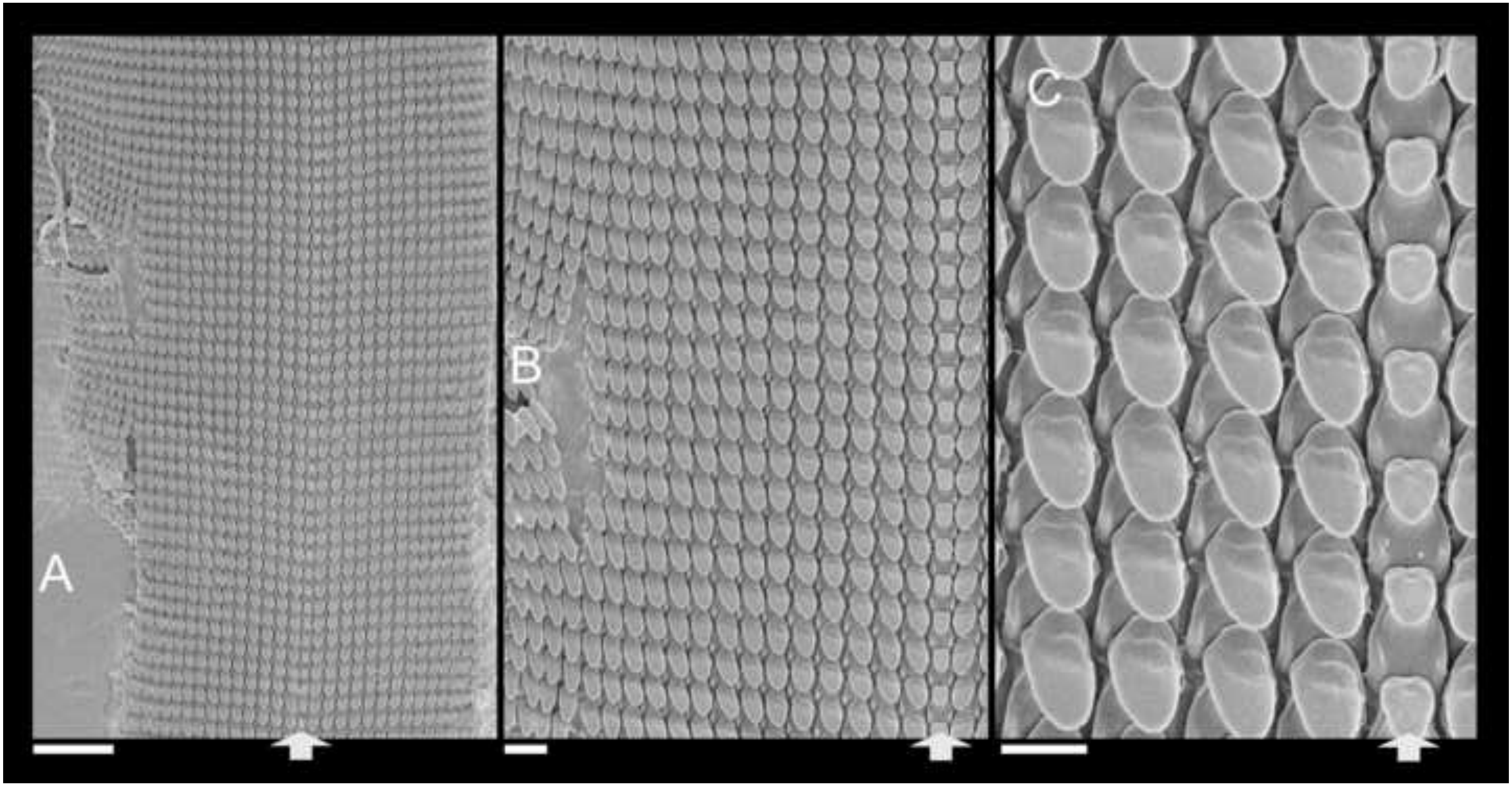

Figure 04

Click here to access/download;Figure;04b Anthinus multicolor 02.jpg

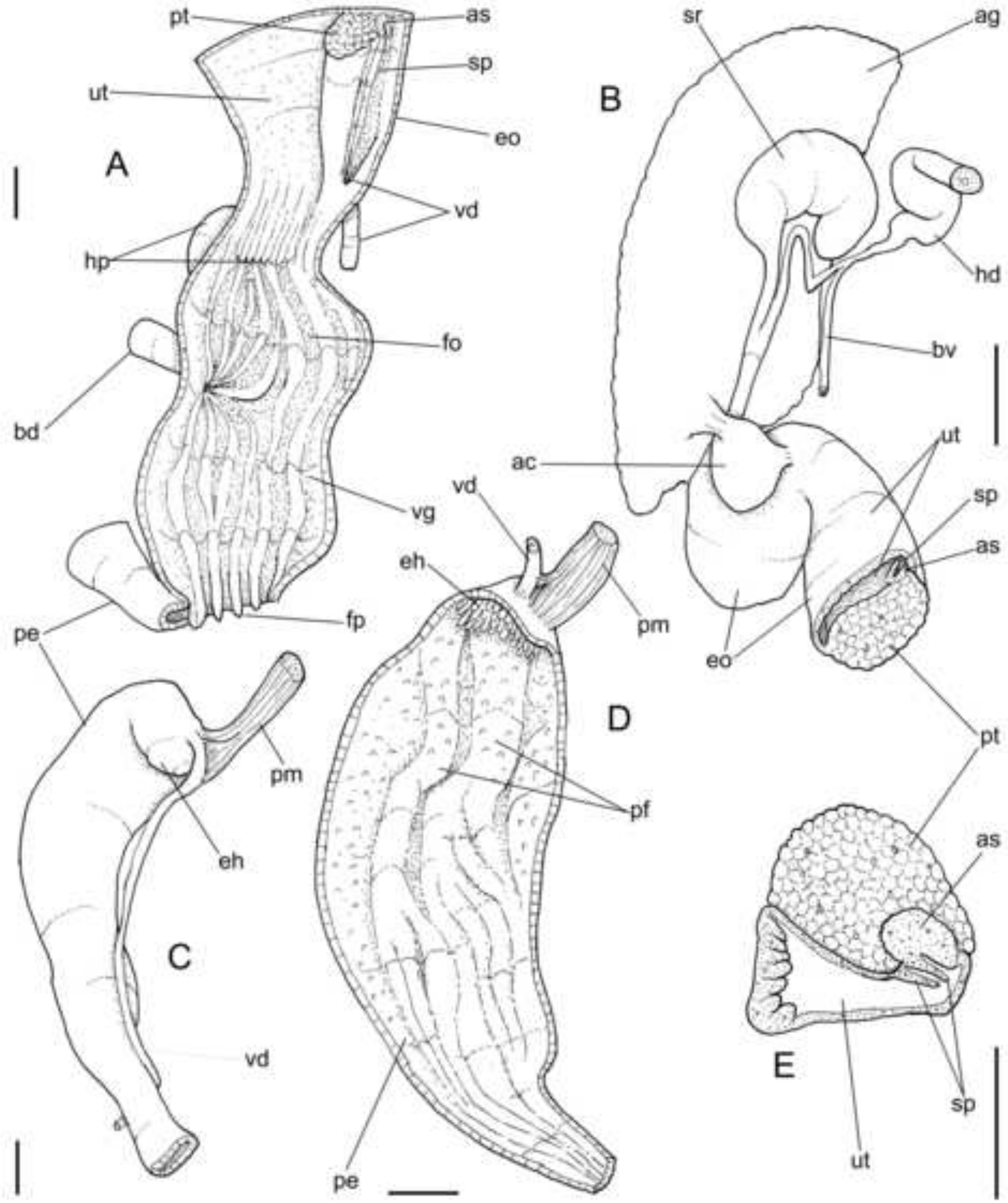

Figure 05

Click here to access/download;Figure;05b Anthinus synchondrus.jpg

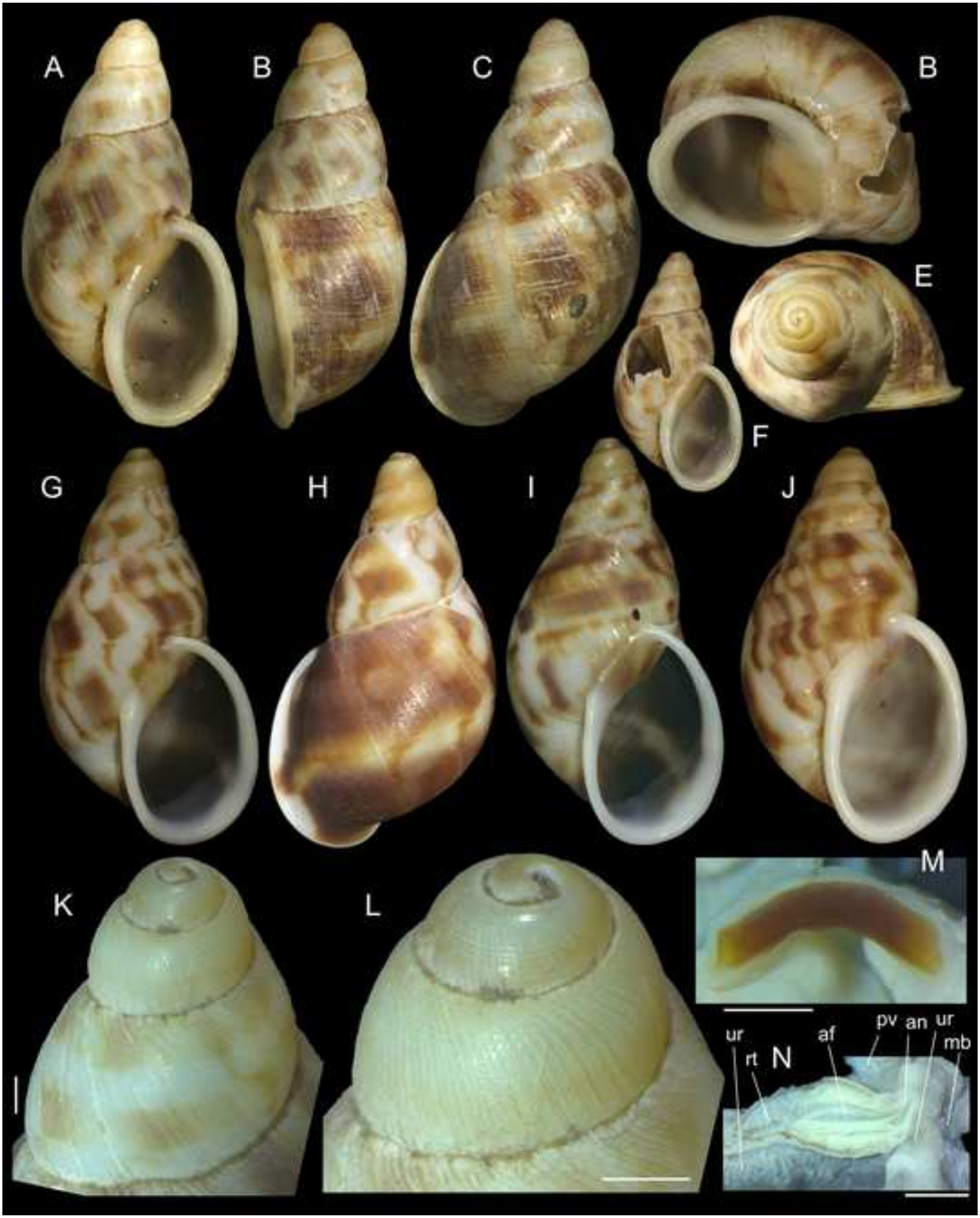

Figure 06

Click here to access/download;Figure;06b Anthinus synchondrus.jpg

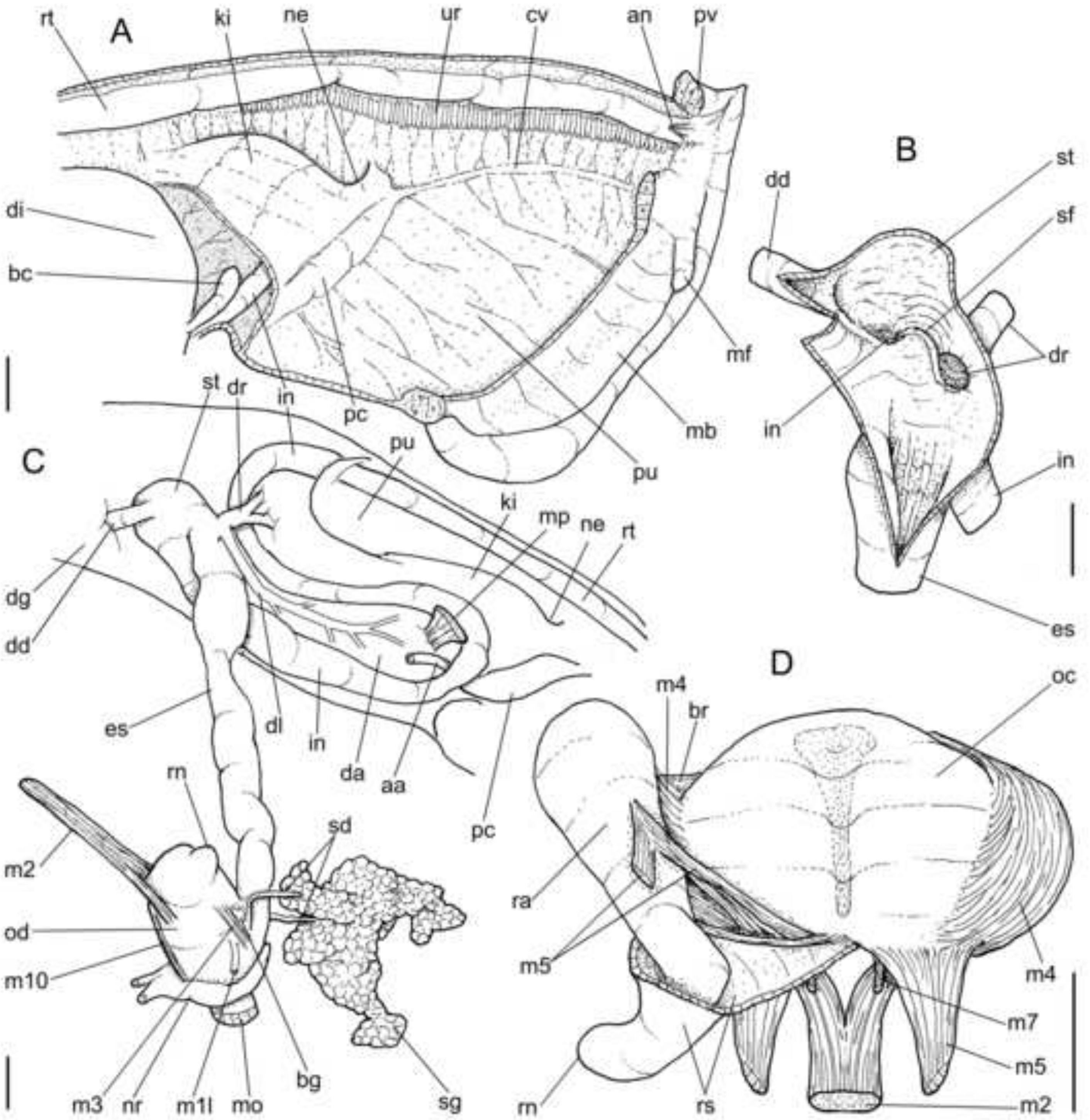

Figure 07

Click here to access/download;Figure;07b Anthinus synchondrus Rad.jpg

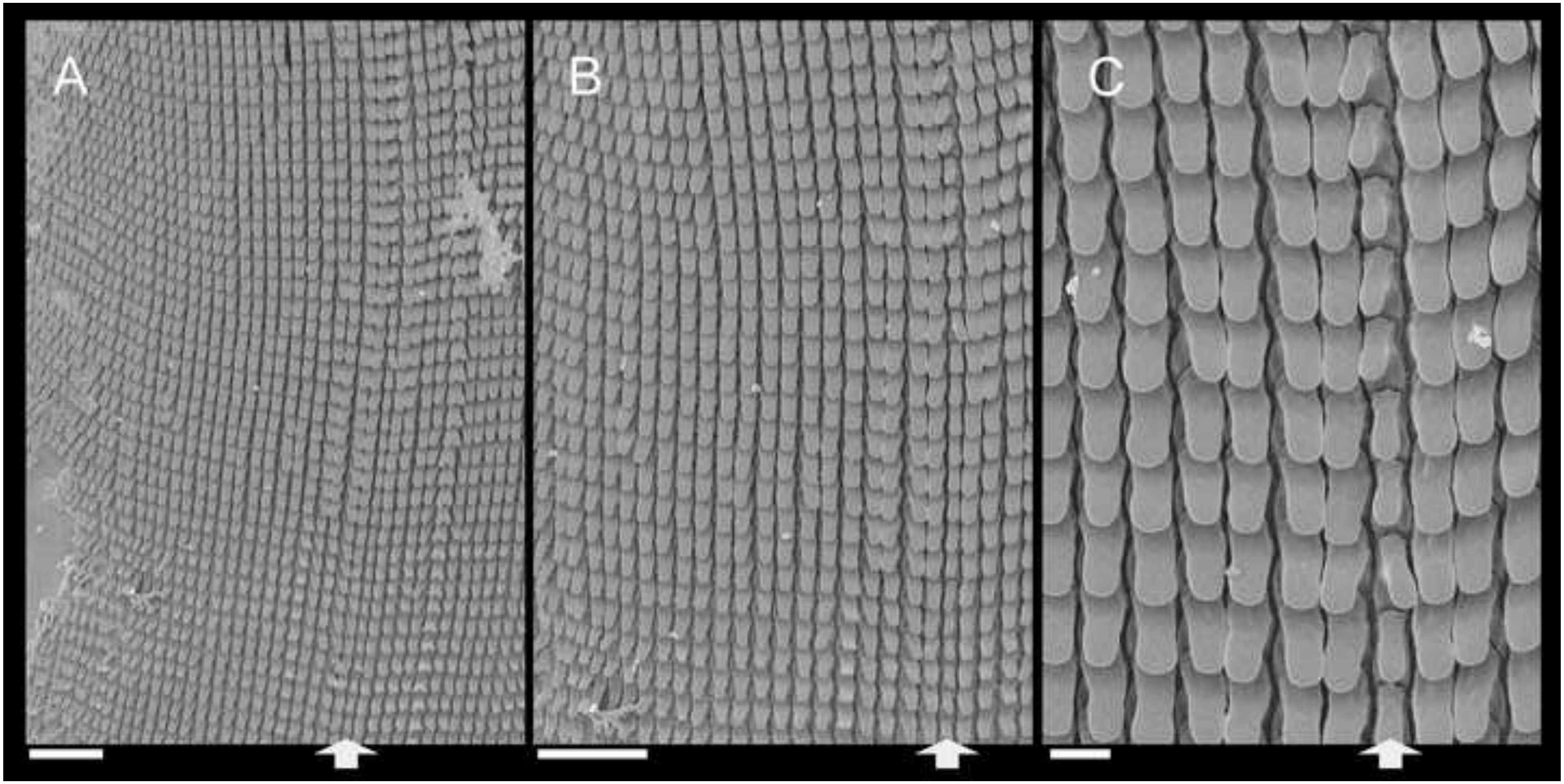

Figure 08

Click here to access/download;Figure;08b Anthinus synchondrus.jpg

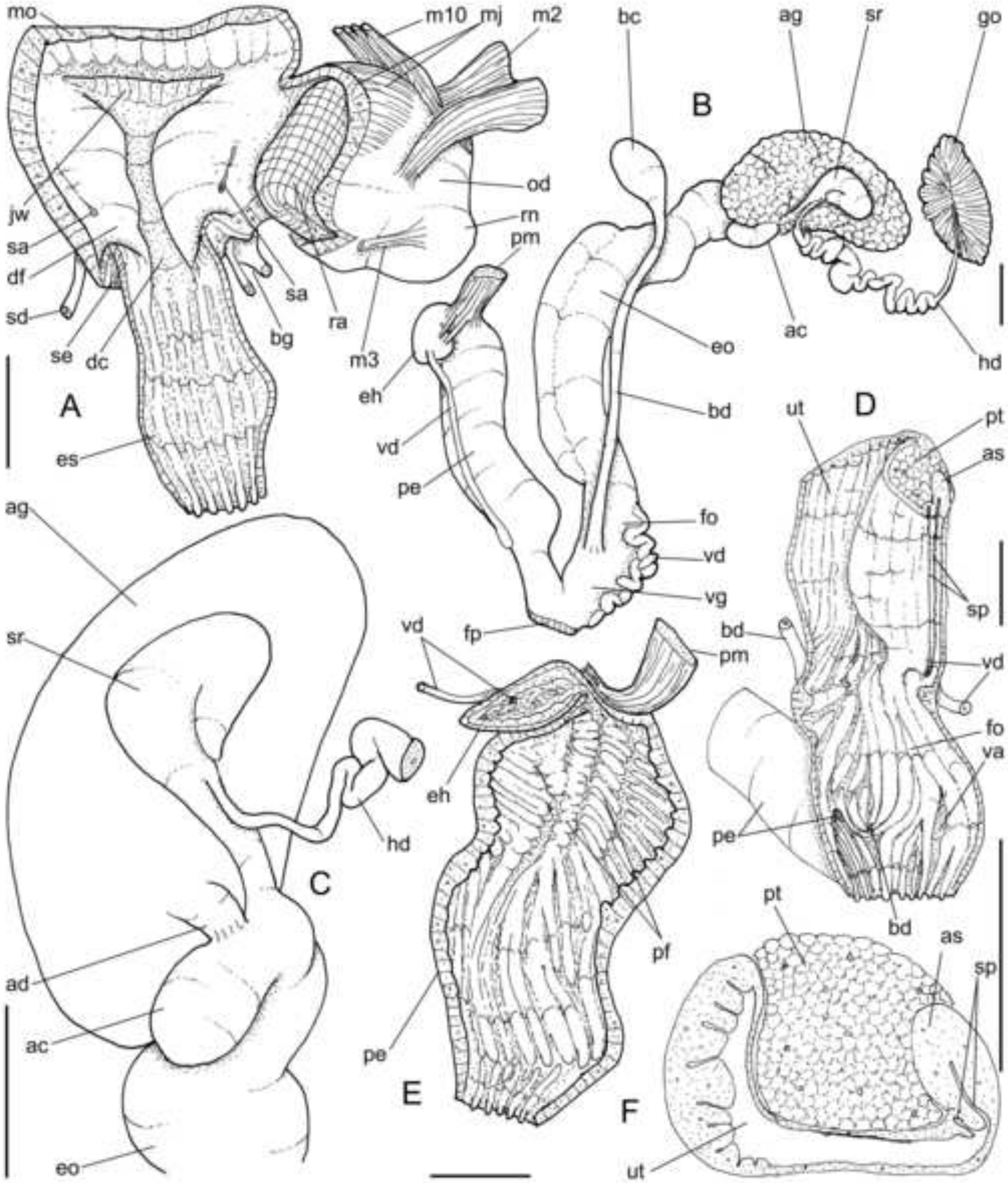

Figure 09

Click here to access/download;Figure;09b Anthinus vailanti.jpg

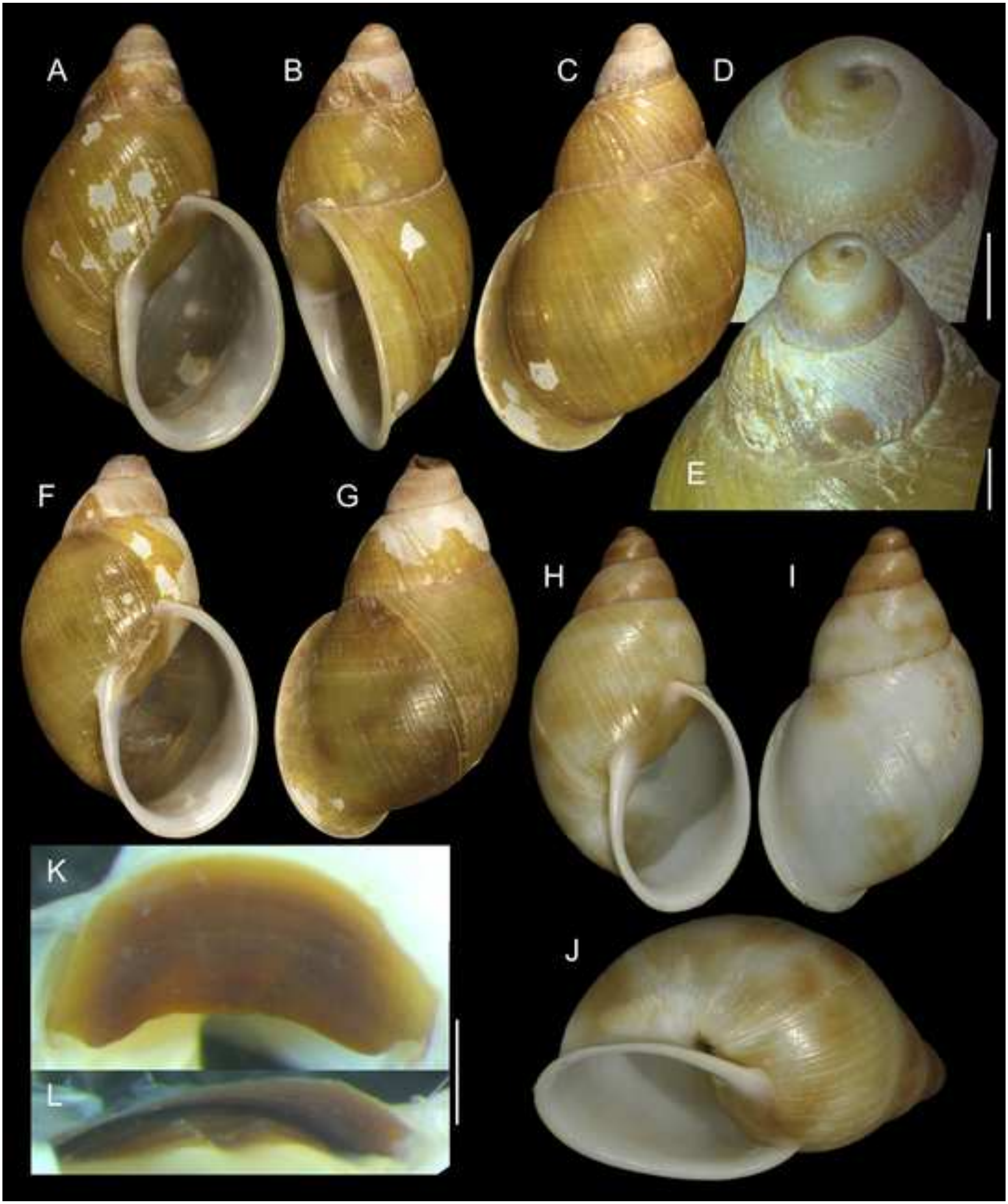

Figure 10

Click here to access/download;Figure;10b Anthinus vailanti.jpg

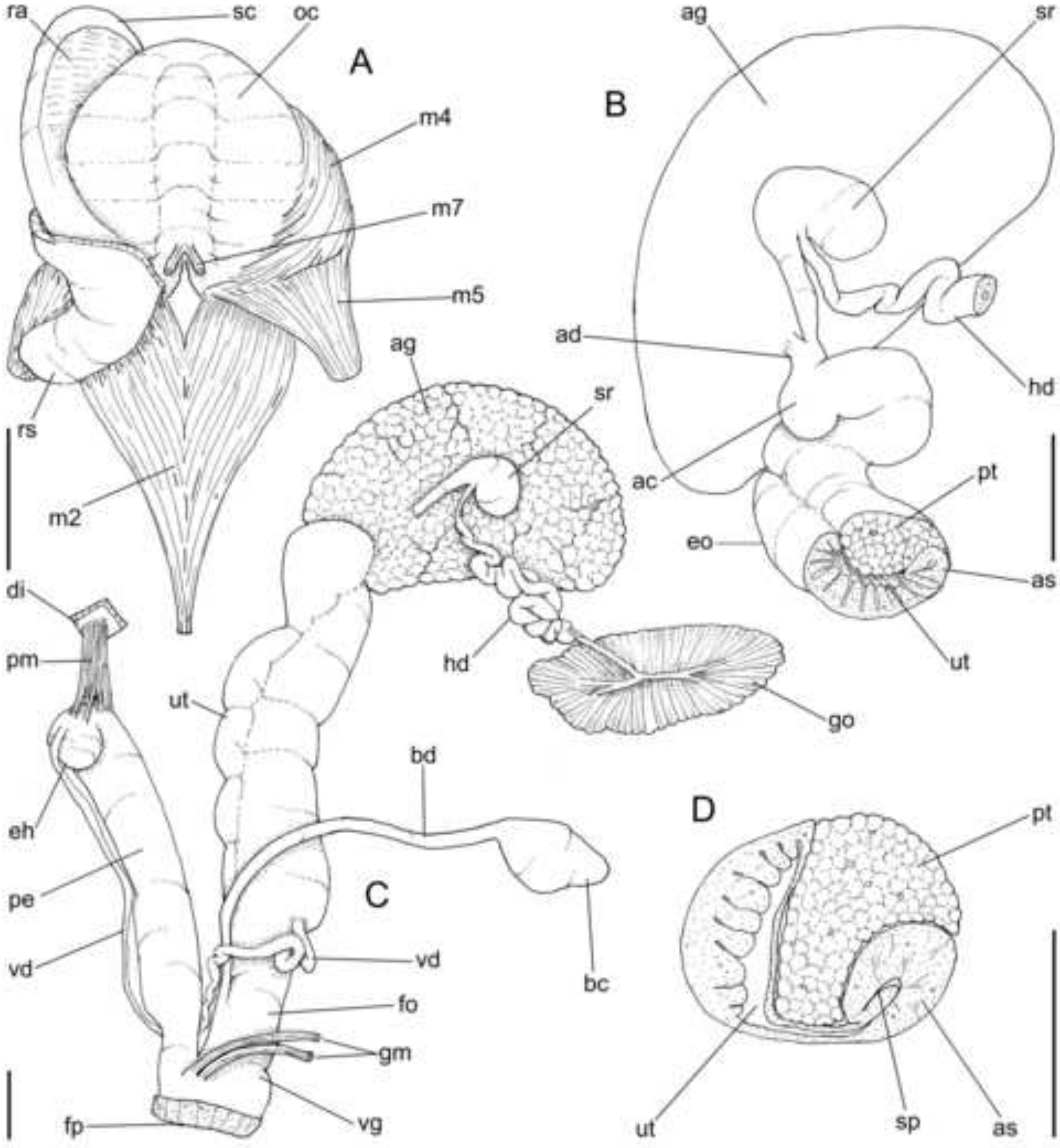

Figure 11

Click here to access/download;Figure;11b Anthinus vailanti.jpg

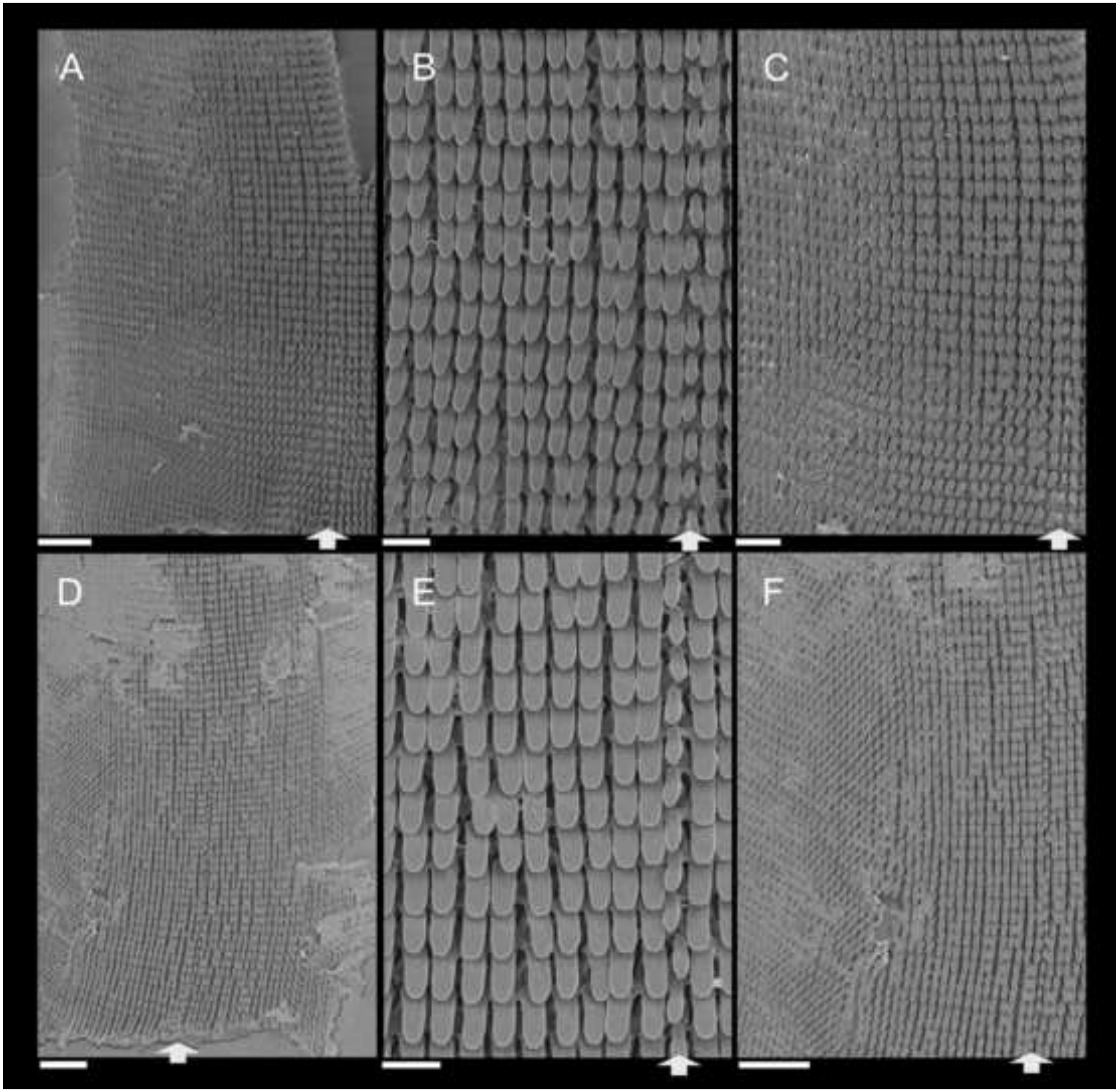

Figure 12

Click here to access/download;Figure;12b Anthinus vailanti.jpg

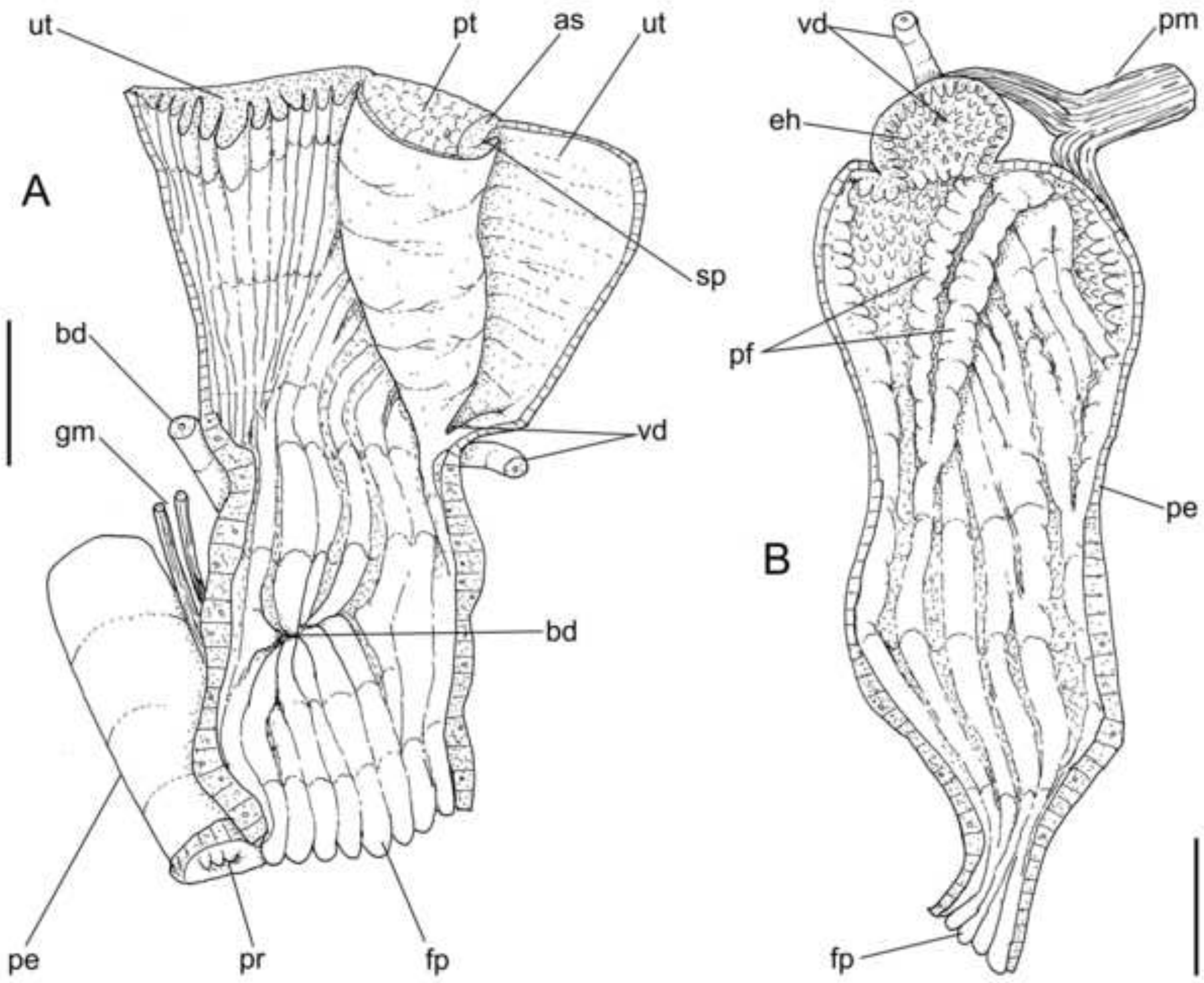

Figure 13

Click here to access/download;Figure;13b Anthinus morenus.jpg

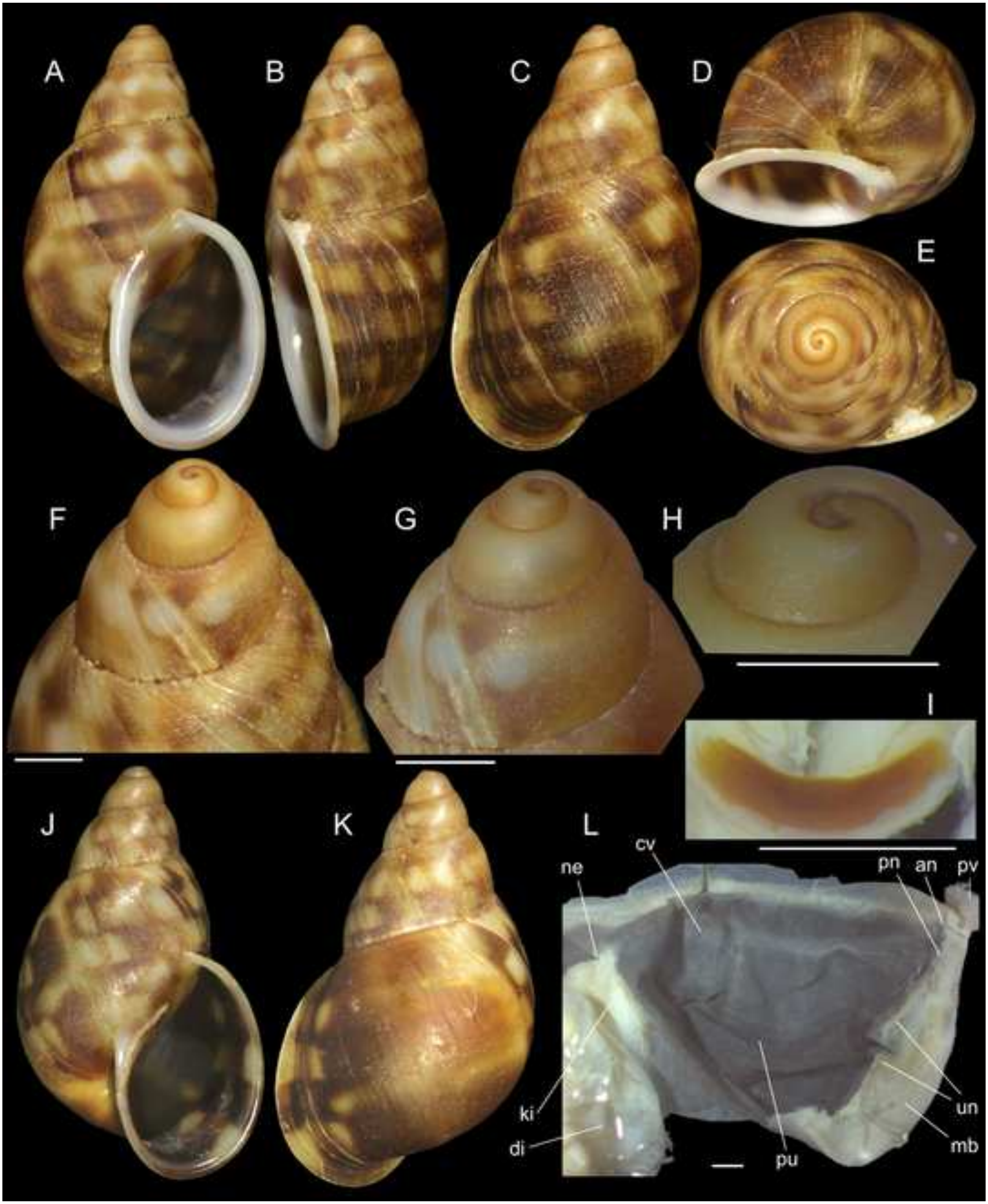

Figure 14 Click here to access/download;Figure;14b Anthinus morenus.jpg

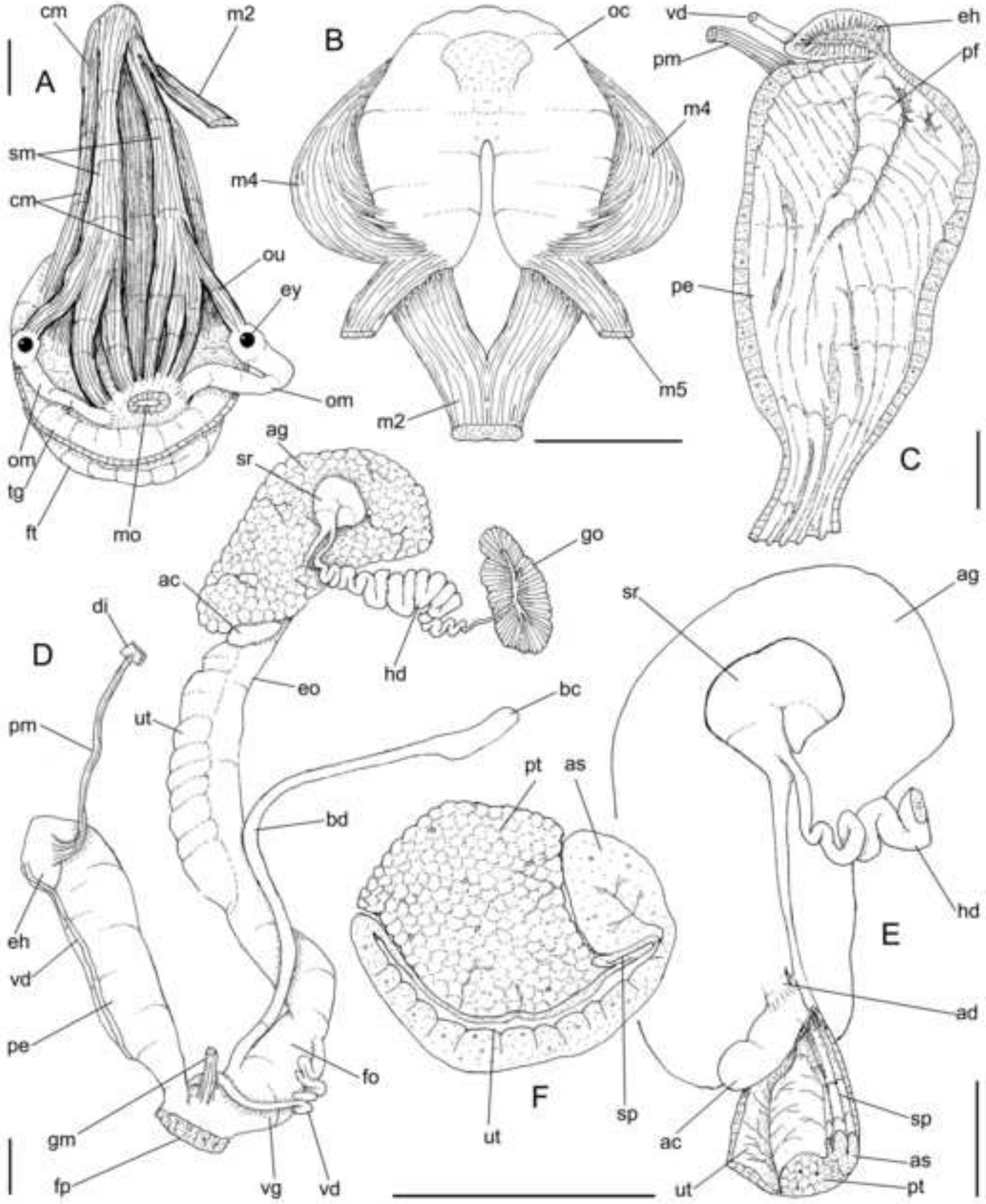

Figure 15 Click here to access/download;Figure;15b Anthinus morenus.jpg

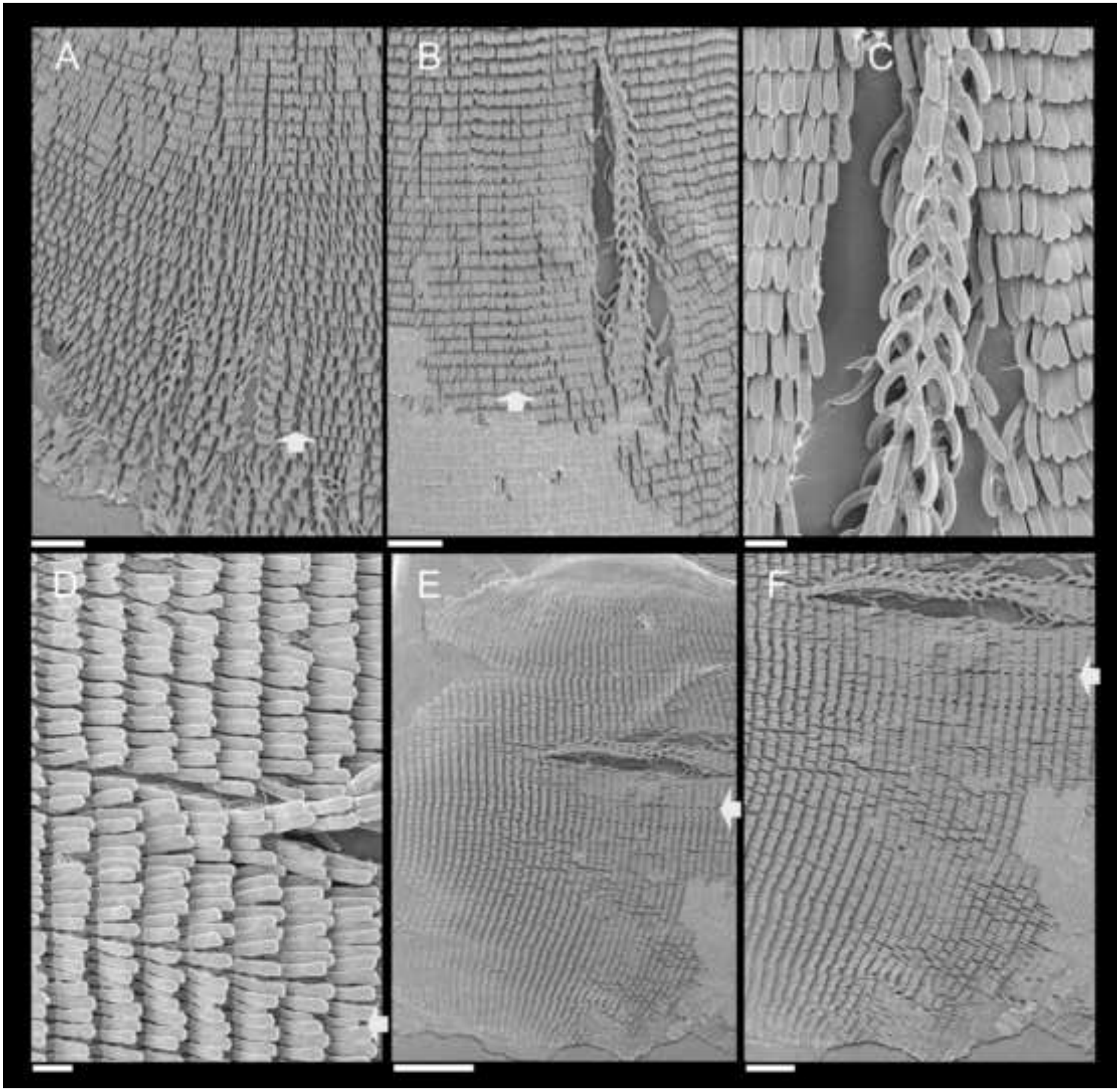

Figure 16

Click here to access/download;Figure;16b Anthinus savanicus.jpg

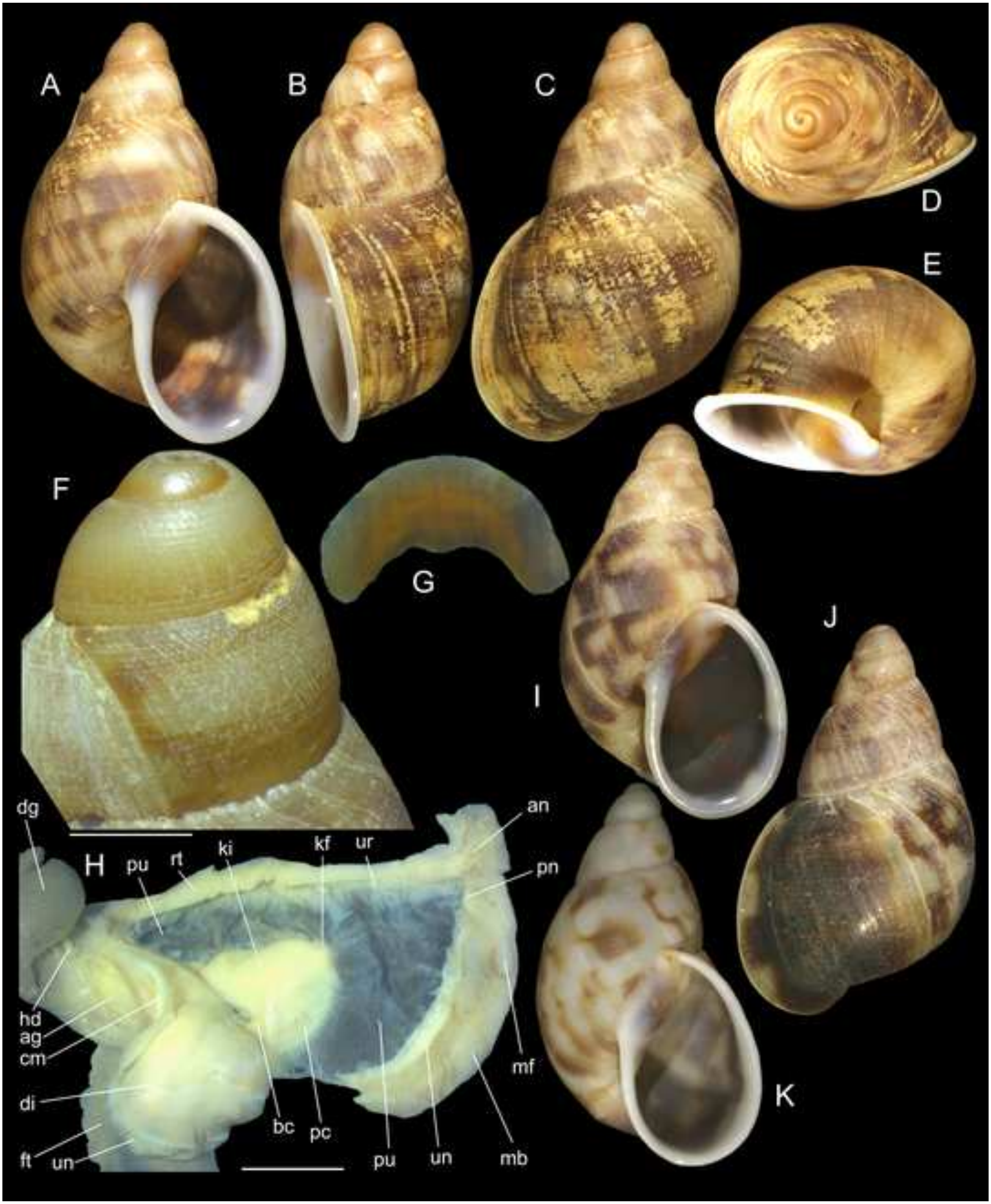

Figure 17

Click here to access/download;Figure;17b Anthinus savanicus.jpg

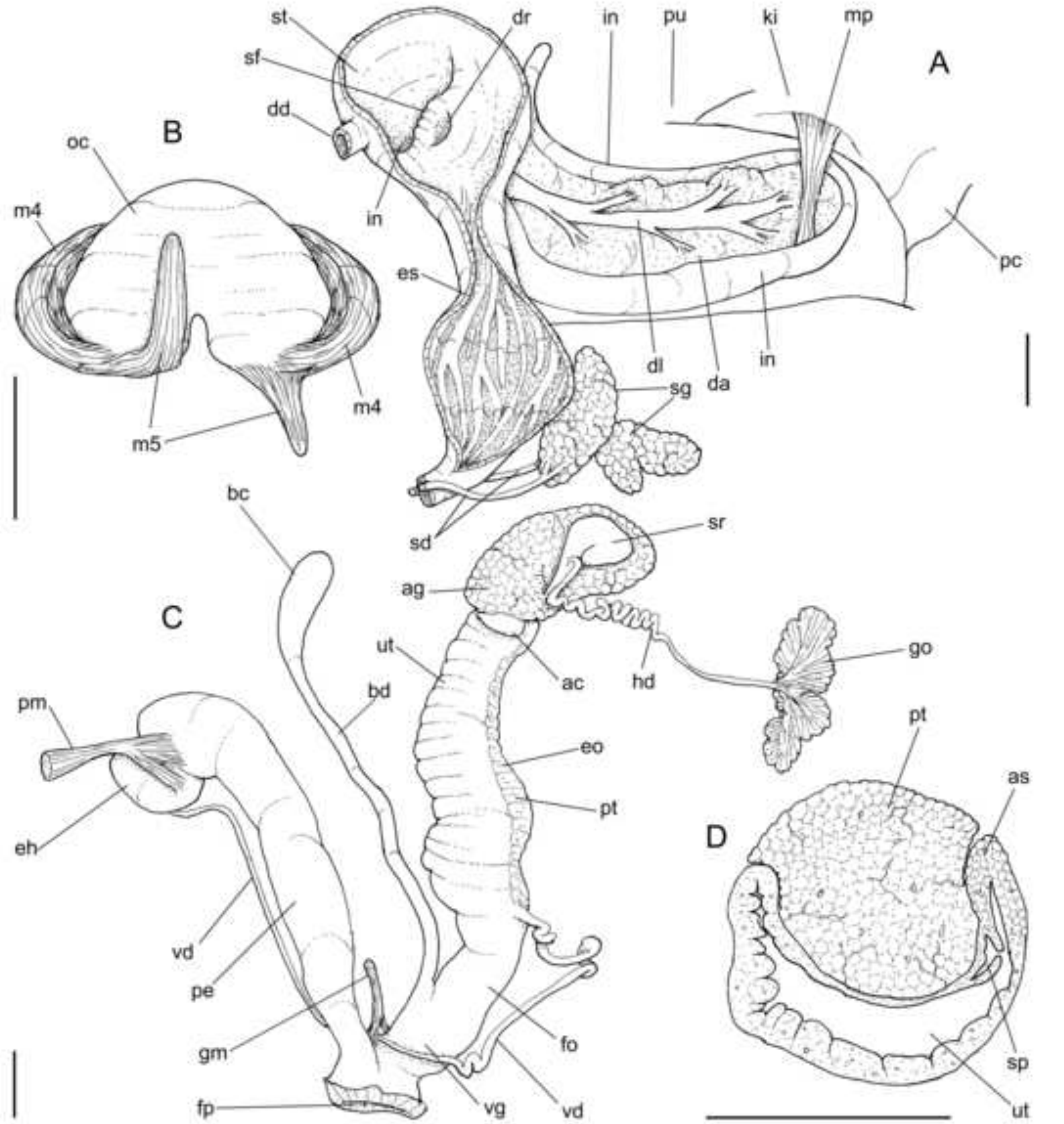

Figure 18

Click here to access/download;Figure;18b Anthinus savanicus.jpg

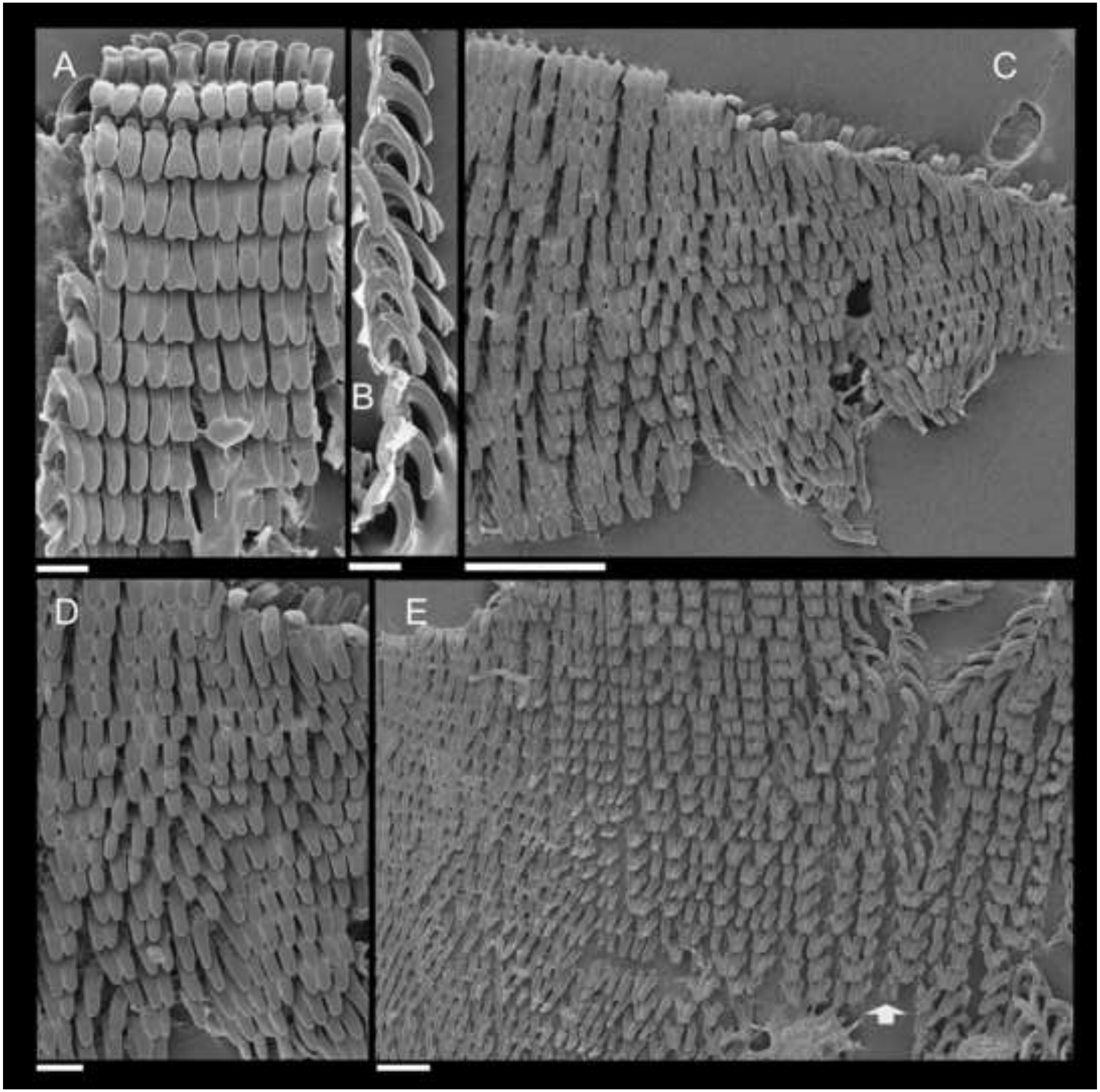

Figure 19

Click here to access/download;Figure;19b Anthinus savanicus.jpg

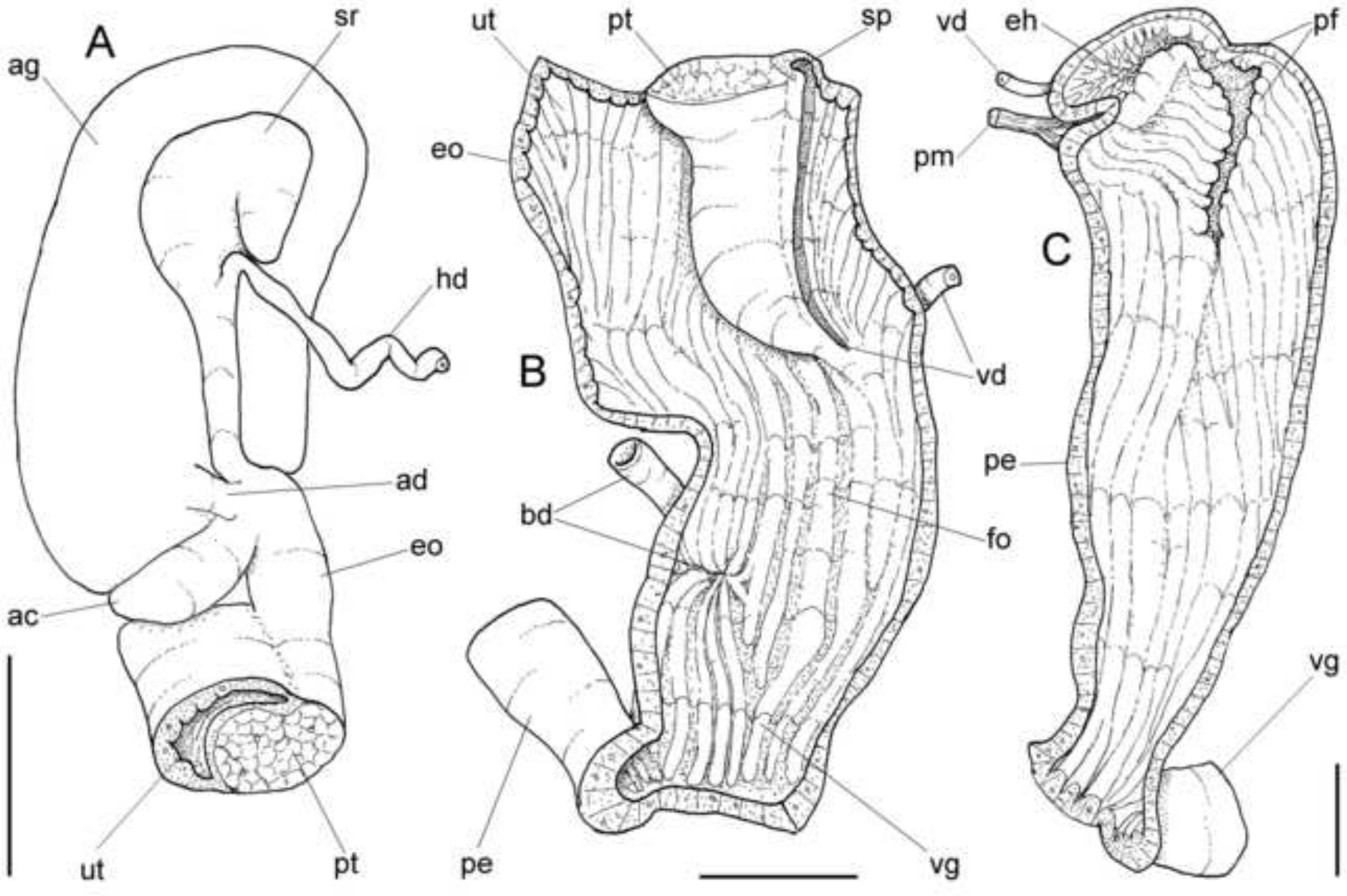

Figure 20

Click here to access/download;Figure;20b Catracca uhlei.jpg

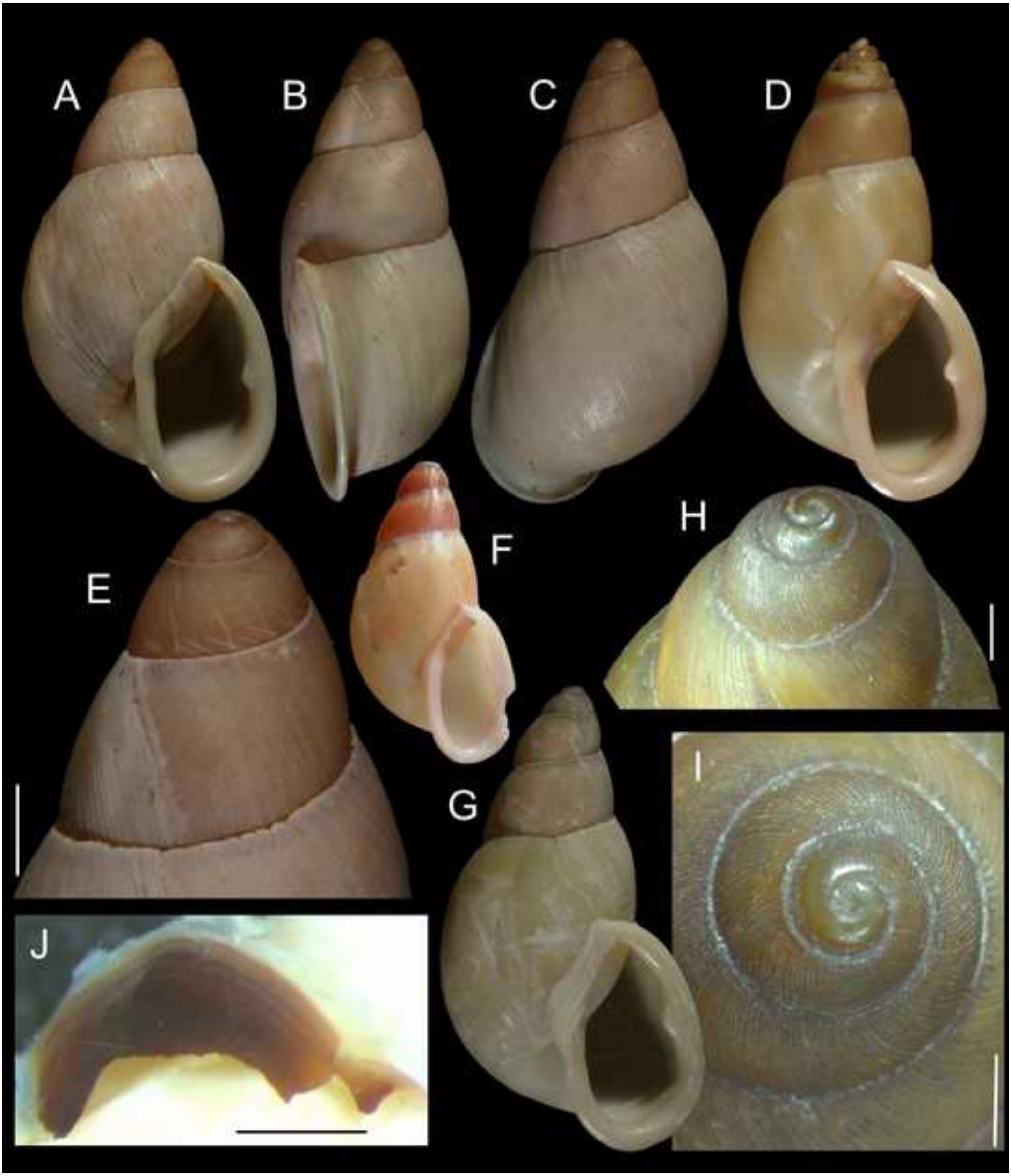

Figure 21 Click here to access/download;Figure;21b Catracca uhlei.jpg

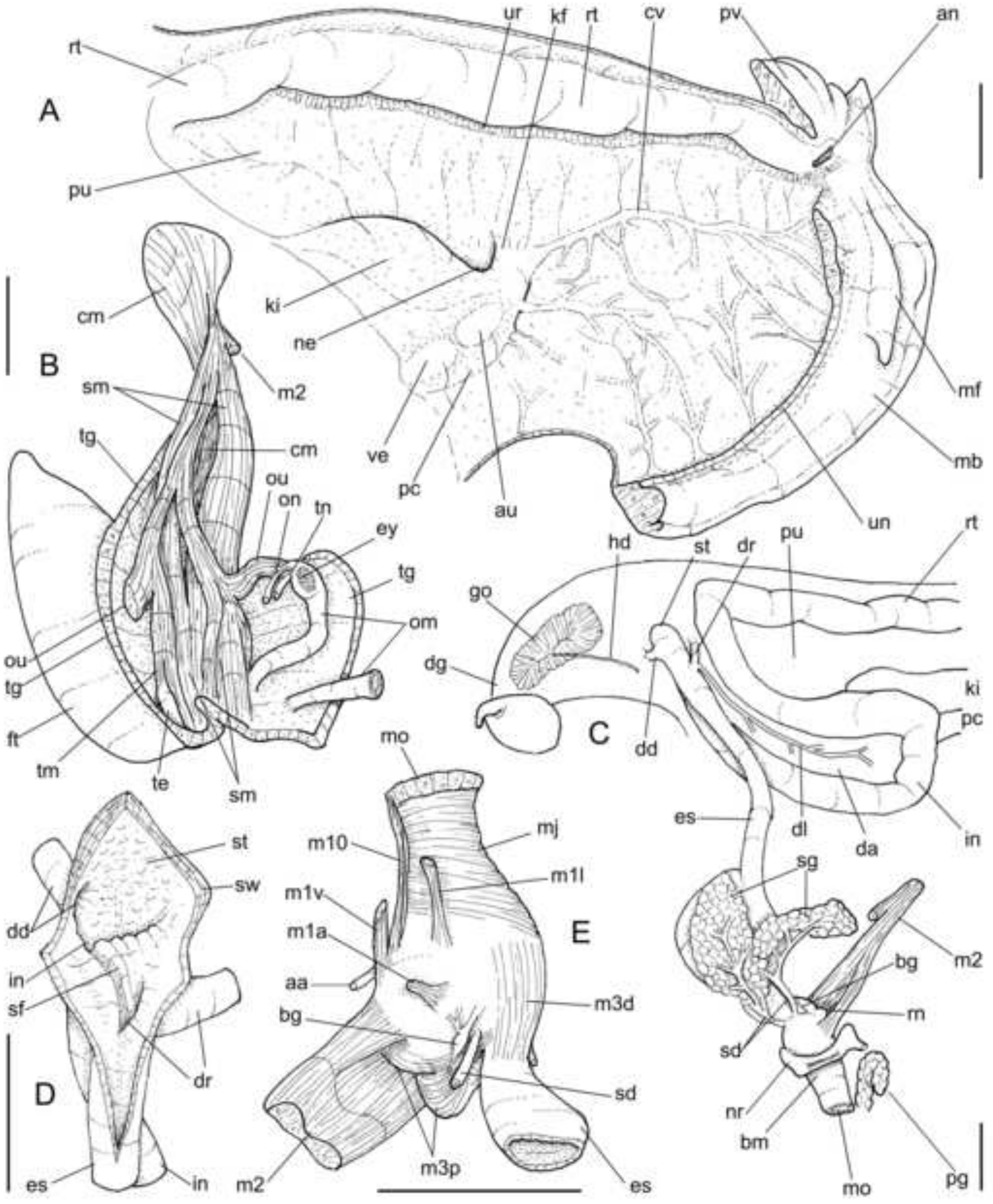

Figure 21 Click here to access/download;Figure;21bb Catracca uhlei.jpg

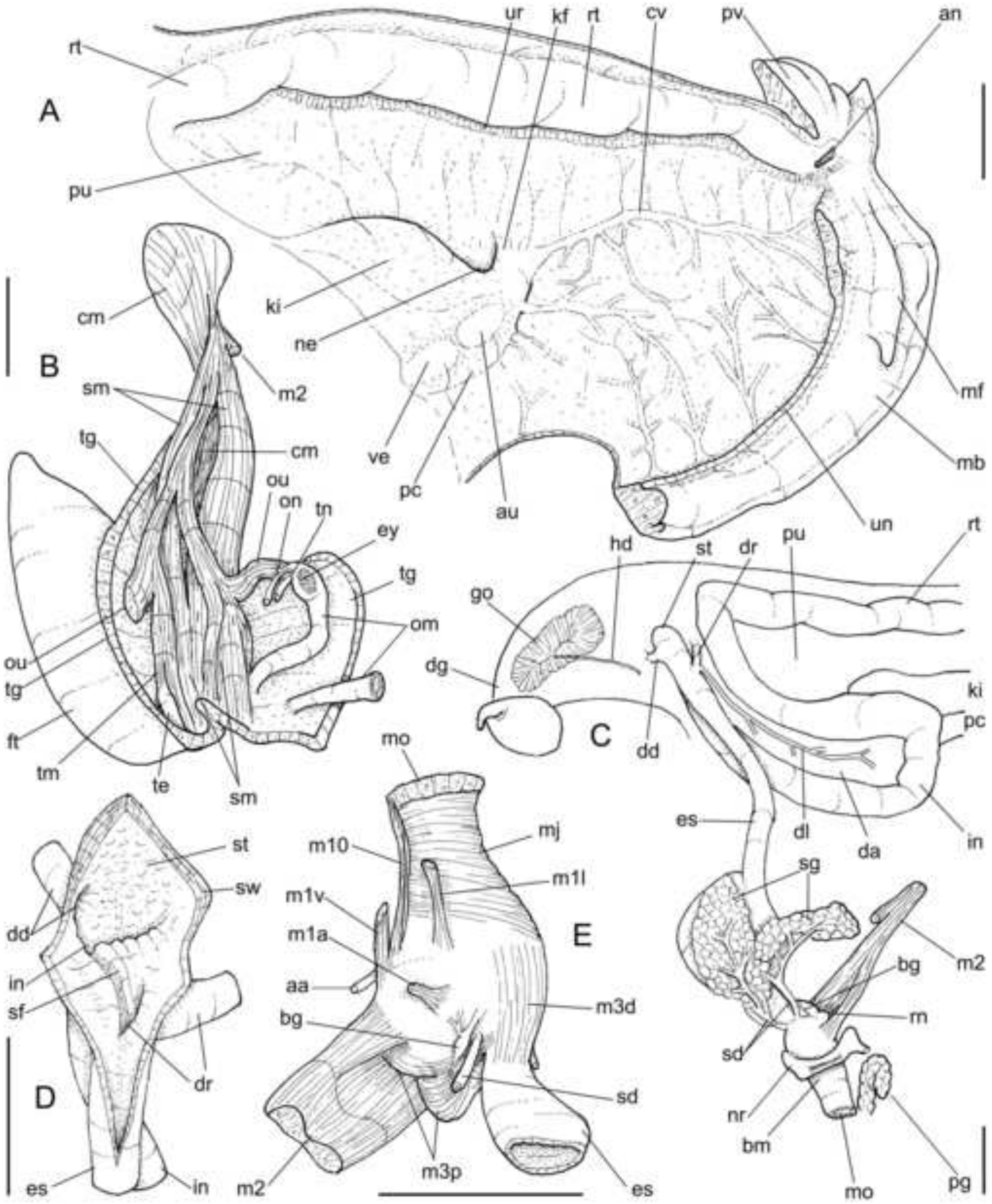

Figure 22

Click here to access/download;Figure;22b Catracca uhlei.jpg

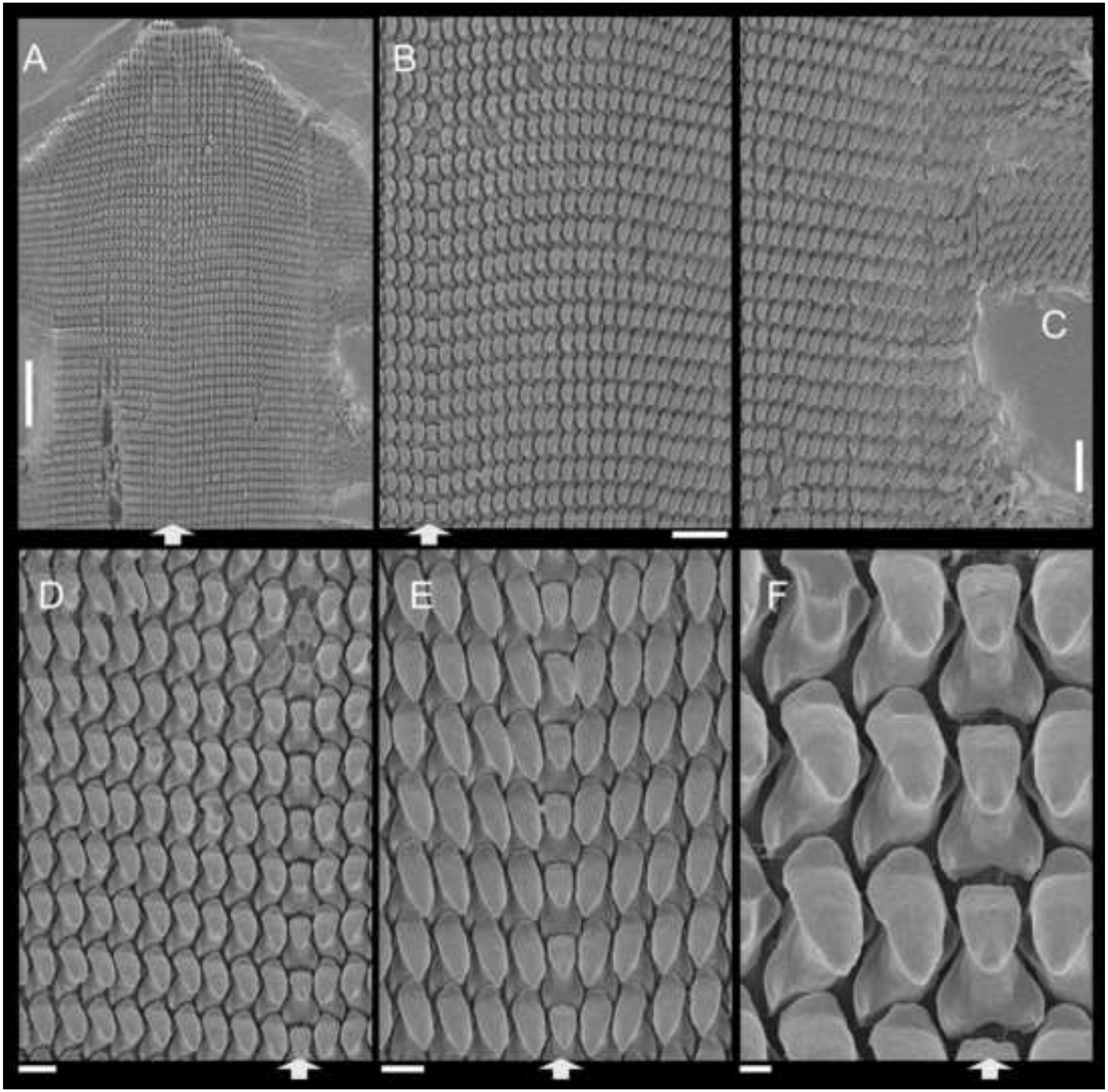

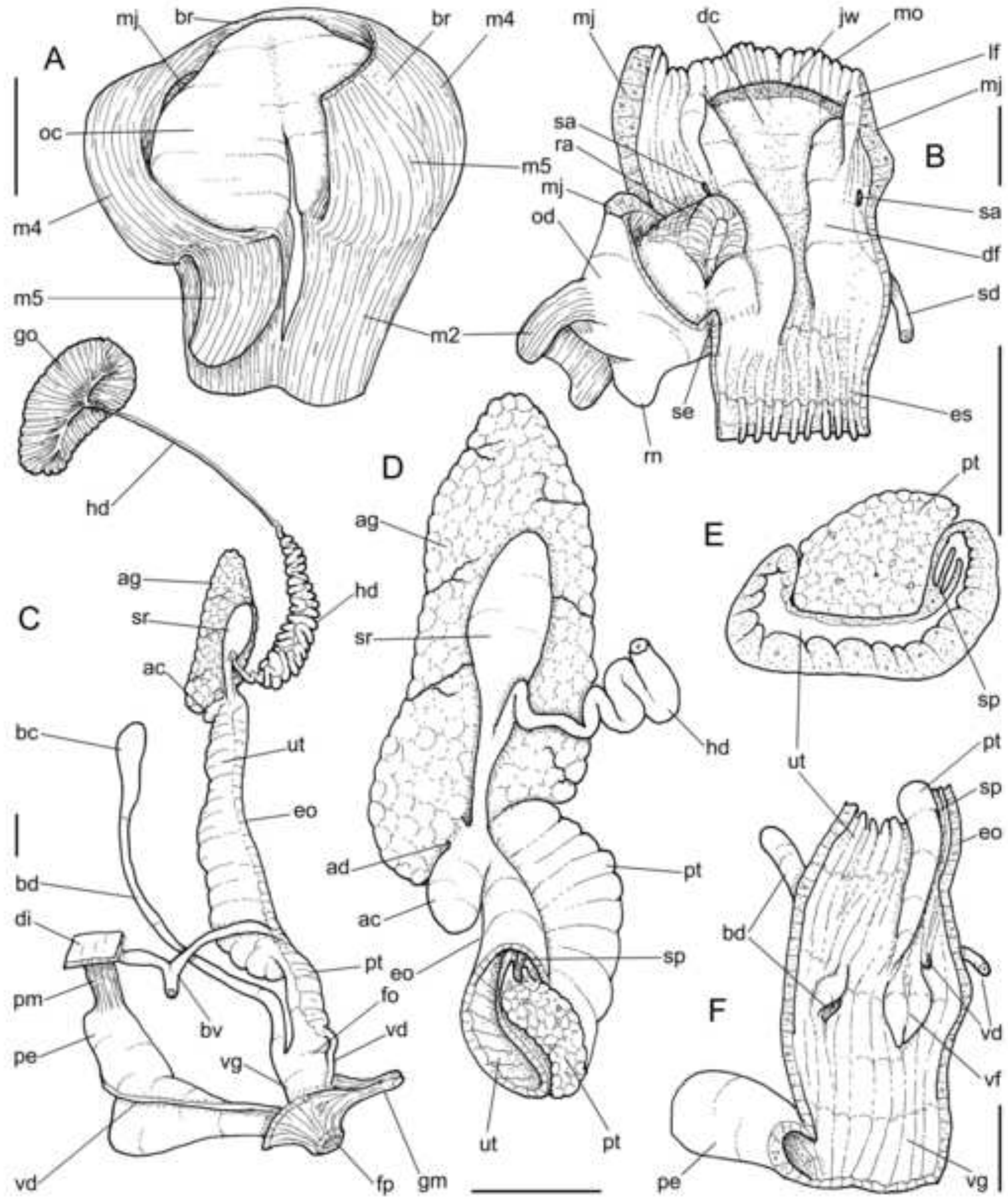
A
mj
br
br
m4
oc
m4
m5
m5
m2
B
mj
dc
jw
mo
lf
mj
sa
ra
mj
od
sa
df
sd
se
rn
es
C
go
hd
hd
ag
sr
ac
bc
ut
eo
bd
di
pm
pe
bv
pt
fo
vd
vg
vd
fp
gm
D
ag
sr
hd
ad
ac
pt
eo
sp
ut
pt
E
pt
ut
sp
F
pt
sp
eo
bd
vd
vf
pe
vg

Figure 24

Click here to access/download;Figure;24b Catracca uhlei.jpg

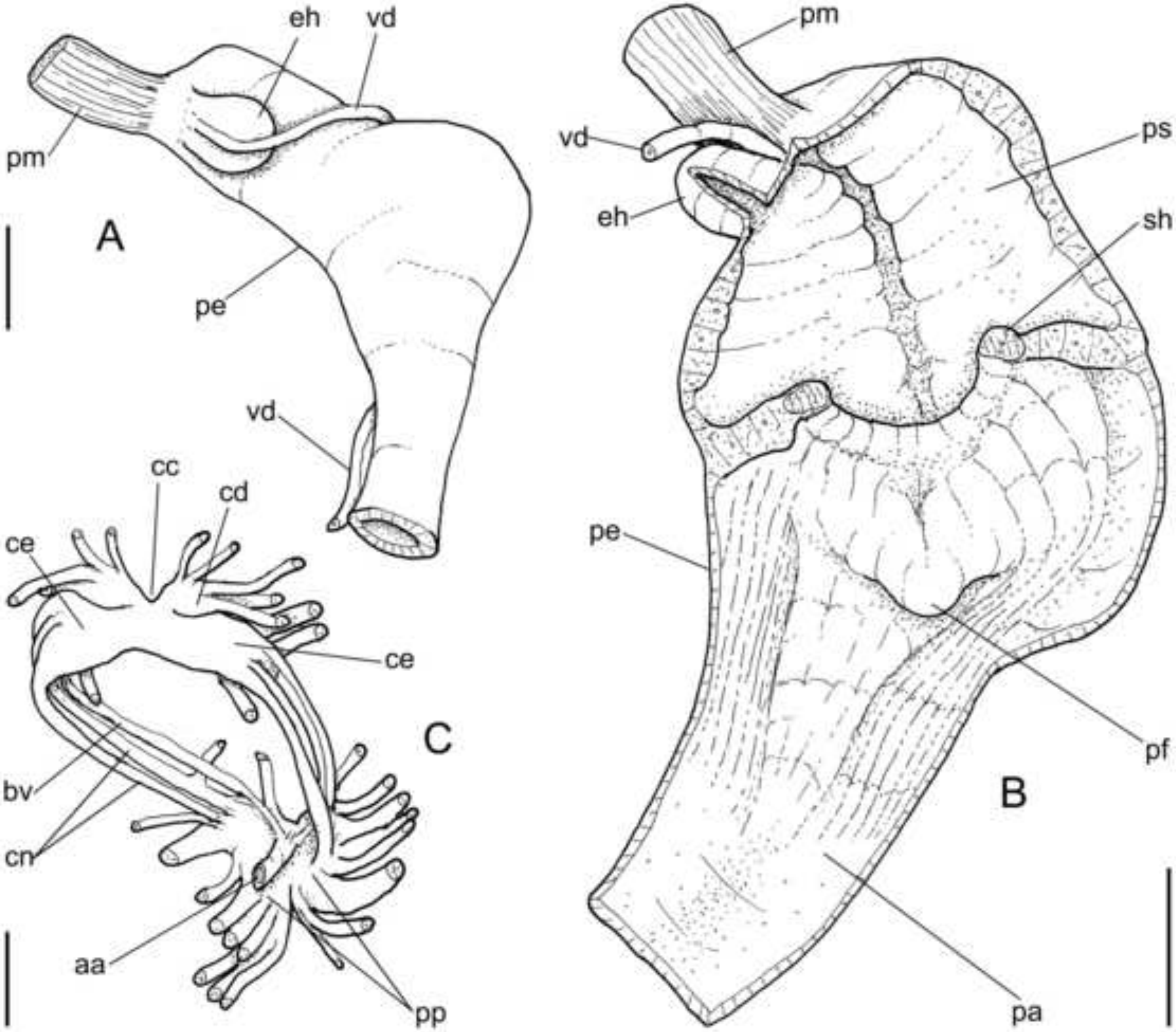

Figure 25

Click here to access/download;Figure;25b Anthinus confrontation.jpg

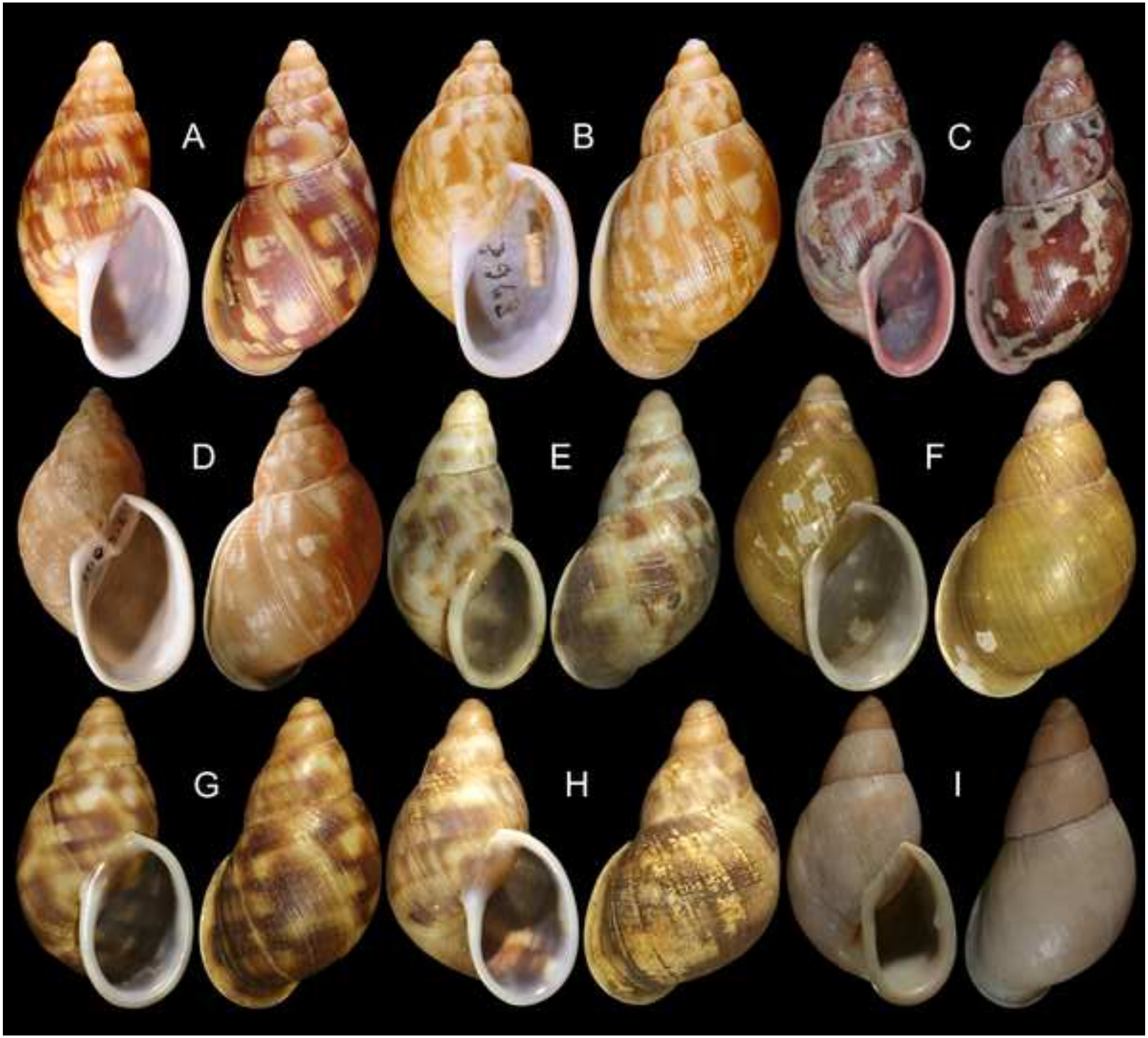

Figure 26

Click here to access/download;Figure;26b Anthinus confrontation2.jpg

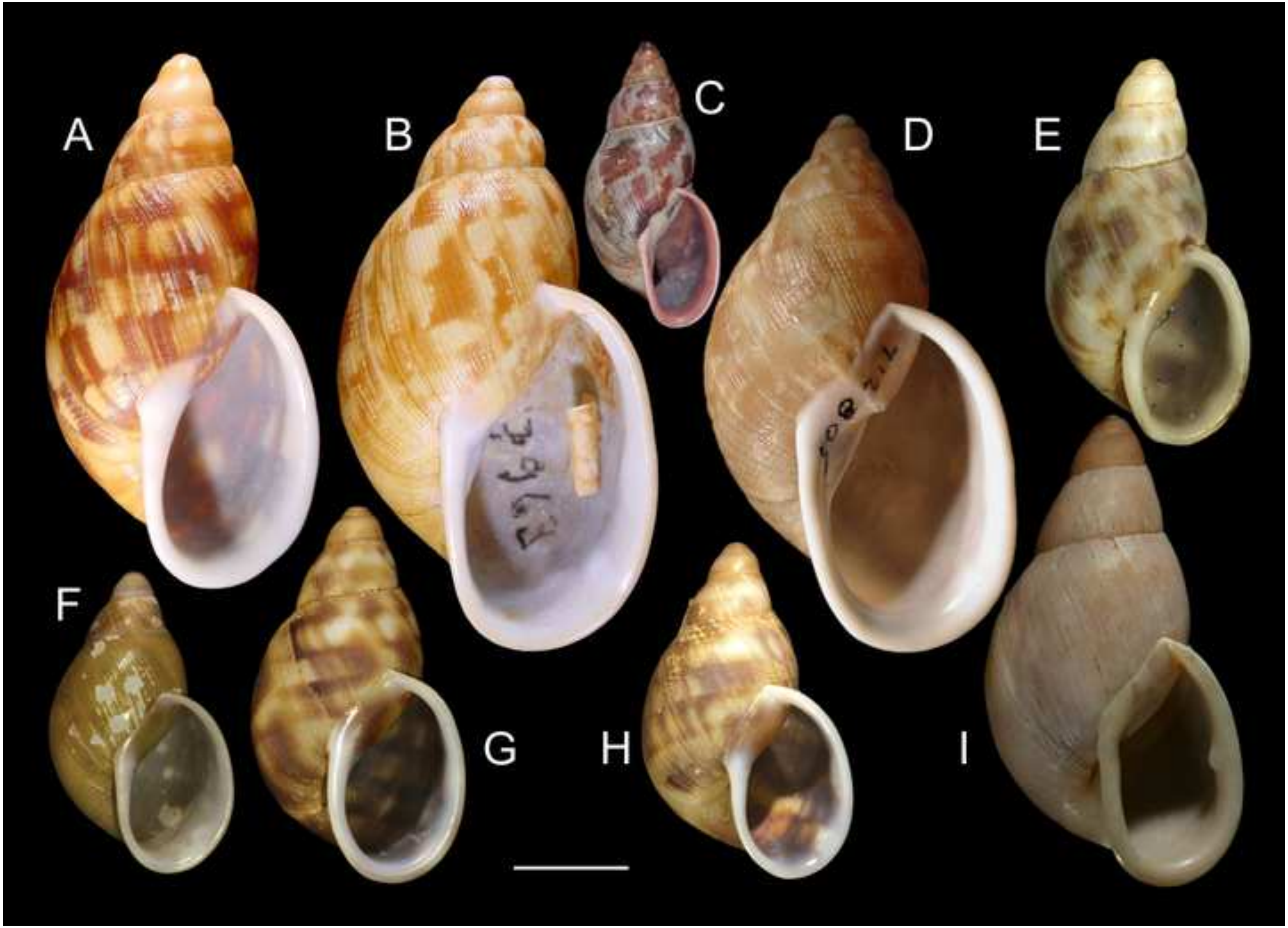

Figure 27

Click here to access/download;Figure;27b Anthinus map.jpg

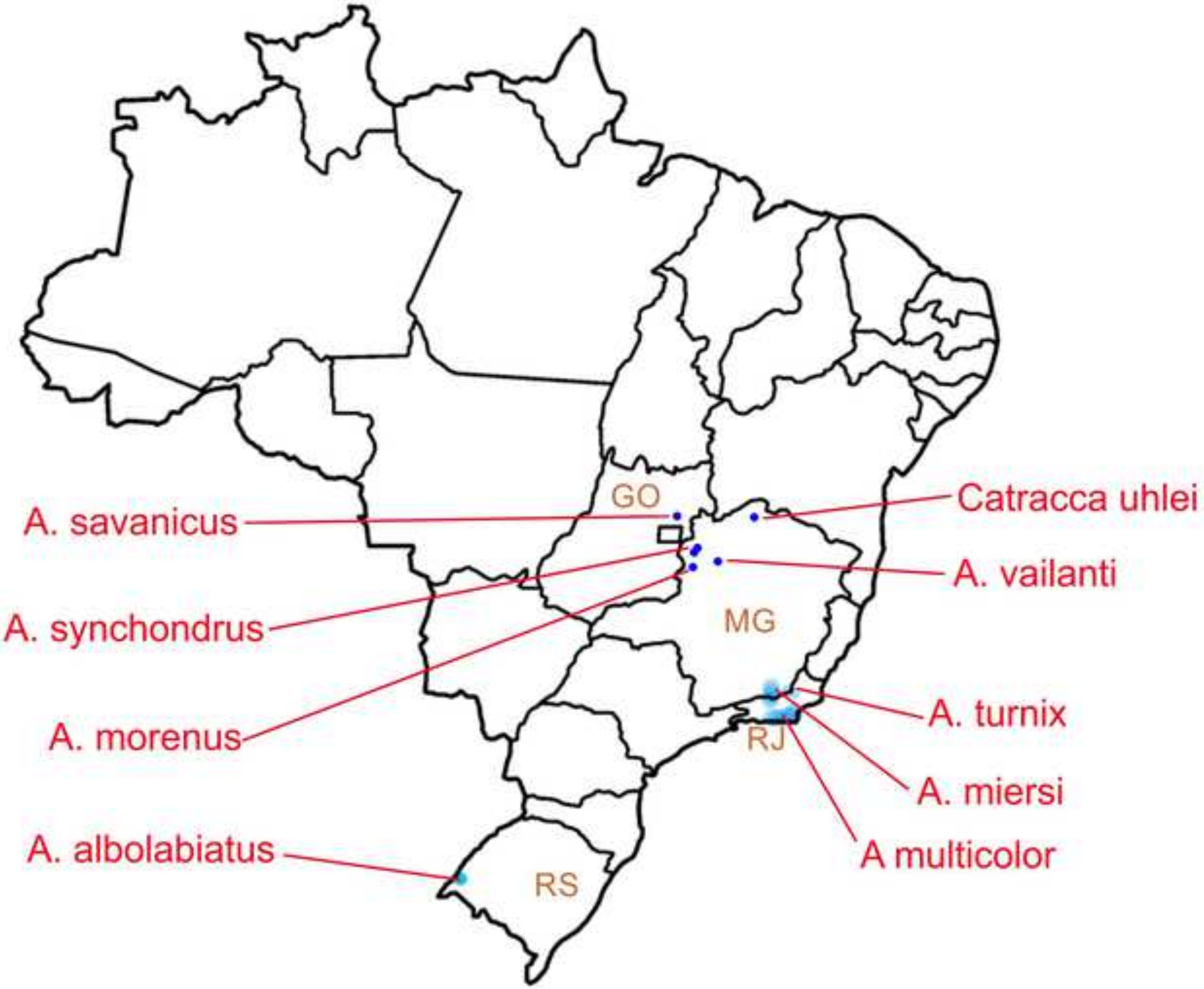

Figure 28

Click here to access/download;Figure;28b Cladogram Anthinus.jpg

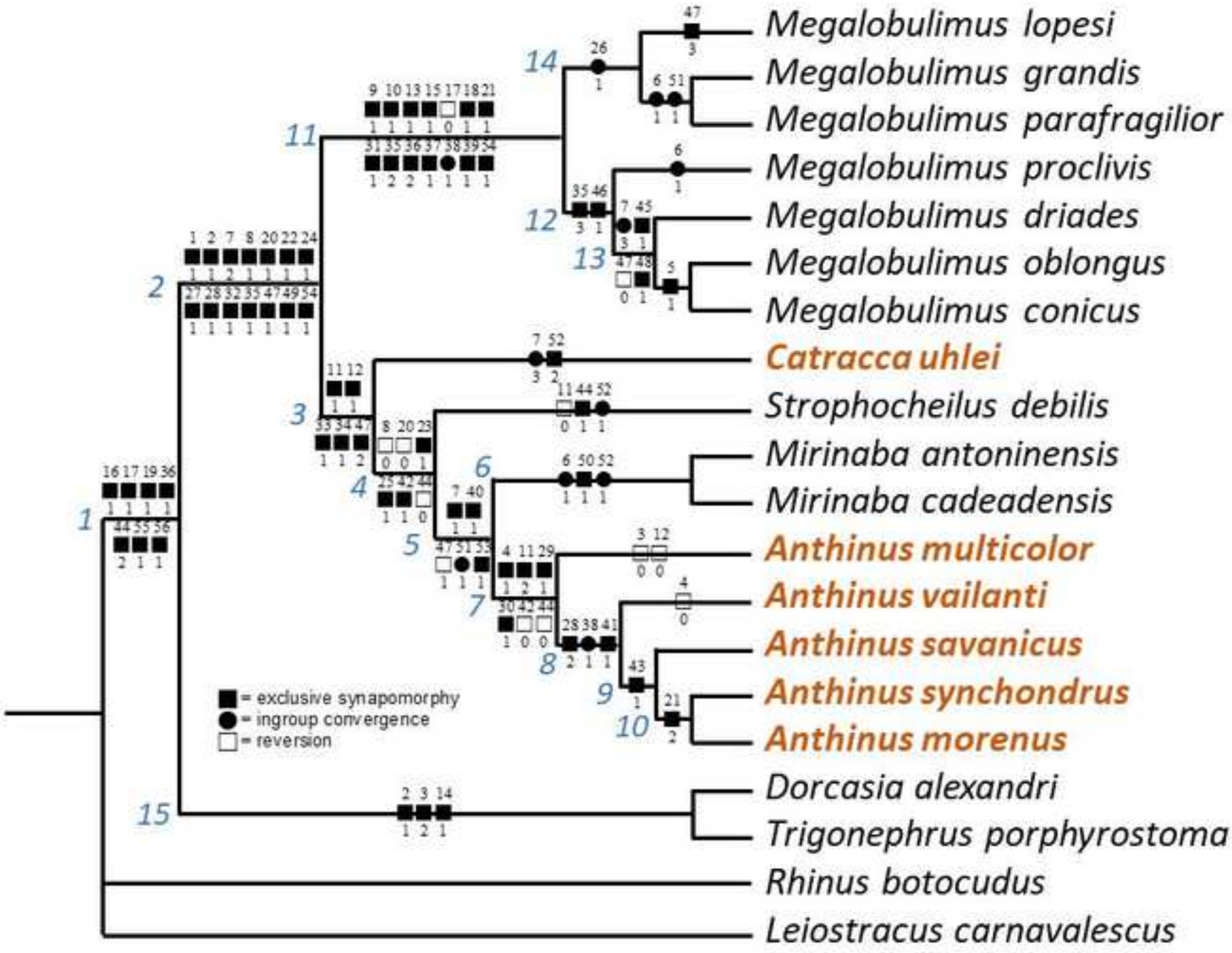

Response to Reviewers

The present document has the answers IN RED to the comments of the three referees, related to the second round of analysis of my paper now titled: **"Additions to the genus *Anthinus* occurring in Minas Gerais and Goiás regions, Brazil, with description of five new species, one of them in the new related genus *Catracca* (Gastropoda, Eupulmonata, Strophocheilidae)."**

The Reviewer #2 was very positive and understanding, approving the paper. The other two Reviewers (#1 and #3), on the other hand, were less friendly, rejecting the paper. However, I think both referees did not completely understand my answers, and that those were requests, and not intransigent denials. Anyway, I answer them below. I did not include the first round of criticisms/answers (PONE-D-21-27031, email 11/25/2021). If it is required, I will add them as soon as I am informed.

It is important to emphasize that the new version practically attended all three reviewers' requirements, looking like worthy for publication.

6. Review Comments to the Author

Reviewer #1: It pains me to see that the author has made only minor cosmetic changes to the Introduction and the Discussion and did not address any of the major problems raised by myself and the other reviewers, like the creation of a pointless genus-level taxon and the complete lack of a phylogenetic structure and molecular data. In almost all cases, the author just stated "that is what I have always done, therefore it is right" or "this is what has always been done in malacology, therefore it is right", without addressing any of the issues. While it is true that some practices in science and in taxonomy in particular date back to the 18th century, they are not necessarily correct or even acceptable in our present 21st century community. For instance, the author commented that a DNA analysis can only be performed after the species has been named, which is preposterous. The DNA analysis is an immediate manner of uncovering information to support or refute the hypothesis of whether the purported OTUs represent new a species or not. The inclusion of a molecular analysis alone could save this manuscript. In addition, the author also did not fully address the ethical and competing interests in naming the species by presenting a necessary disclaimer. The author did not correct any of the figures, keeping his images without scale, which makes proper comparison of the shells very difficult.

The new version, presently submitted, attends all this referee's requirements. The single one that does not meet them is the introduction of molecular analysis. Despite the molecular analysis undoubtedly being an additional data, the already presented conchological, morphological, anatomical, histological and biogeographic approach are convincing enough for basing all the conclusions.

Example: the new version includes a phylogenetic analysis to attend this referee. Although it can be considered provisional, as there are not too many strophocheilid species anatomically known in detail, the analysis clearly additionally supports the

conclusions and the description of the new genus. This can be checked in the submitted new version.

I really sorry that this referee did not consider all my justifications adequate. Anyway, I am afraid he/she did not understand them completely. This is a conclusion I realized by some of the above mentioned assertives, which were not what I stated. An example is "that is what I have always done, therefore it is right" or "this is what has always been done in malacology, therefore it is right". This no way is my point! That I said is, with more than 300 published papers in international journals (Plos included) and ~3,000 anatomic descriptions (check my CV website), I already have a usual, orthodox model which I follow in my papers. This orthodoxy facilitates any interested reader to find what he/she is interested in. At no point did I stated "therefore I am right". This is a dogmatic thought and even an offence, which I securely do not apply as justification. Another example of my answer's misunderstanding is: "DNA analysis can only be performed after the species has been named". I never said that, I only stated that any complementary analysis (molecular included) should be more intelligible if all taxa are already well-established taxonomically. That "only be" are not my words.

The plates of shells usually do not bear scale bars. The direct length (L) is put in the legend. This bakes the plates clean and esthetically more presentable, without losing information. All partial views and anatomical images do have scale bars.

Anyway, and despite these misunderstandings, I think the new version attends the immense majority of this referee's requirements.

Reviewer #2: The revised manuscript is adequate, but still could be improved. That the author has published over 300 papers is acknowledged, but there is always opportunity to improve. Seems the author has not fully taken on the suggestions (that is all they are, suggestions) from the reviewers. I am happy to recommend acceptance, but there are two points that need to be addressed:

This referee was much more understanding. Even though I always try to follow suggestions, they are suggestions as the referee said, consequently non-obligatory. If well-justified, some assertions can be not or only partially accepted.

1. No information is given on the specimens examined for the anatomical work. The 'material examined' listed for each species mentions only shell material. Were the specimen(s) examined anatomically for Catracca uhlei, for example, from the type locality or from elsewhere? – this is an important distinction.

I double-checked this and the new version has this information clear. In Material Examined, "shell" is only shell, thus conchologically analyzed. "Specimen" is an entire individual, shell plus soft parts, thus worthy both for conchology and anatomy. All "specimens" were anatomically investigated.

2. Discussion. 'Specific debate'
The reviewers suggested some alternative formats for presentation of the text relating to shell dimension data – e.g. tabulation or ordination biplots. The author has not accepted

the recommendations and retained the rather laborious text, at great loss of clarity. I think the author should reconsider.

This new version has its Discussion divided into topics. I think this is adequate. However, if the referee or the editors suggest more subdivisions or another "less laborious" model, I will be happy in modifying that part.

There are a number of editorial corrections to be made through out the text - spelling, etc. In Acknowledgements, Barker not Narker

Okay, everything was double-checked. The text was corrected by two different language softwares, and additionally was corrected by a native English speaker Mr. Lee Kremer.

Reviewer #3: The author got a lot of constructive suggestions how to improve his manuscript from three reviewers. He rejected almost all of them.
If he considers "proper measurements" "not necessary in the present context" (!), if he is not able to summarize the differences between his putative species in a single sentence or an easy to read table but hides them in his terribly lengthy text and if he makes higher taxa on a merely phenetic basis without even considering to think about phylogeny, the paper has to be rejected. This is the worst "revision" I have ever seen.

The new version, presently submitted, attends all this referee's requirements. I am sorry this referee did not like my previous revision. Possibly he/she did not read my explanations completely (most were requests, not intransigent rejections) and did not check that I did not "rejected almost all of them", on the contrary, a majority was accepted. It is important to emphasize that "a merely phenetic basis" is ~99% of Mollusca taxonomy, and the base of my career. I wonder if no dataset is more trustable than others in Biology. This does not sound like a good argumentation. Anyway, to attend to this criticism, the new submitted version has a phylogenetic analysis. It is an additional support for the conclusions.

The distinctions amongst the studied species (new or not) are exposed in a taxon viewpoint – the Diagnoses; in a comparative viewpoint – the Distinctive Descriptions; and in a debatable context – the Discussion. What else must I do to "summarize the differences between his putative species"? A table? It is now present, along with the Phylogenetic analysis.
