# Supplementary material for: Additions to the genus Anthinus occurring in Minas Gerais and Goiás regions, Brazil, with description of five new species, one of them in the new related genus Catracca (Gastropoda, Eupulmonata, Strophocheilidae)
Source: PLoS One. 2022 Aug 24;17(8):e0273067. doi: 10.1371/journal.pone.0273067 (PMC9401170; doi:10.1371/journal.pone.0273067)
Supplement: Attachment — Submitted filename: 2022 Anthinus criticisms PLOS ONE.pdf [file pone.0273067.s004.pdf]

PLOS ONE <em@editorialmanageAnswer:com>

7 de maAnswer: de 2022 13:02 (há 7 dias)

para mim

PONE-D-22-00958
Additions to the genus Anthinus occurring in Minas Gerais and Goiás regions, Brazil, with description of five new species, one of them in the new related genus Catracca (Gastropoda, Eupulmonata, Strophocheilidae).
PLOS ONE

Dear D: Simone,

Thank you for submitting your manuscript to PLOS ONE. After careful consideration, we feel that it has merit but does not fully meet PLOS ONE’s publication criteria as it currently stands. Therefore, we invite you to submit a revised version of the manuscript that addresses the points raised during the review process.

Please submit your revised manuscript by Apr 21 2022 11:59PM. If you will need more time than this to complete your revisions, please reply to this message or contact the journal office at plosone@plos.org. When you're ready to submit your revision, log on to https://www.editorialmanageAnswer:com/pone/ and select the 'Submissions Needing Revision' folder to locate your manuscript file.

Please include the following items when submitting your revised manuscript:

- A rebuttal letter that responds to each point raised by the academic editor and reviewer(s). You should upload this letter as a separate file labeled 'Response to Reviewers'.
- A marked-up copy of your manuscript that highlights changes made to the original version. You should upload this as a separate file labeled 'Revised Manuscript with Track Changes'.
- An unmarked version of your revised paper without tracked changes. You should upload this as a separate file labeled 'Manuscript'.

If you would like to make changes to your financial disclosure, please include your updated statement in your cover letter. Guidelines for resubmitting your figure files are available below the reviewer comments at the end of this letter.

If applicable, we recommend that you deposit your laboratory protocols in protocols.io to enhance the reproducibility of your results. Protocols.io assigns your protocol its own identifier (DOI) so that it can be cited independently in the future. For instructions see: https://journals.plos.org/plosone/s/submission-guidelines#loc-laboratory-protocols. Additionally, PLOS ONE offers an option for publishing peer-reviewed Lab Protocol articles, which describe protocols hosted on protocols.io. Read more information on sharing protocols at https://plos.org/protocols?utm_medium=editorial-email&utm_source=authorletters&utm_campaign=protocols.

We look forward to receiving your revised manuscript.

Kind regards,

Michael Schubert
Academic Editor
PLOS ONE

Journal Requirements

1. When submitting your revision, we need you to address these additional requirements.

Please ensure that your manuscript meets PLOS ONE's style requirements, including those for file naming. The PLOS ONE style templates can be found at https://journals.plos.org/plosone/s/file?id=wjVg/PLOSOne_formatting_sample_main_body.pdf and https://journals.plos.org/plosone/s/file?id=ba62/PLOSOne_formatting_sample_title_authors_affiliations.pdf

2. We noted in your submission details that a portion of your manuscript may have been presented or published elsewhere.
(previous submission (PONE-D-21-27031) edited by Michael Schubert)

Please clarify whether this [conference proceeding or publication] was peer-reviewed and formally published. If this work was previously peer-reviewed and published, in the cover letter please provide the reason that this work does not constitute dual publication and should be included in the current manuscript.

ANSWER: I have not seen any part of the MS published elsewhere. I only used some already published data of some papers to help the phylogenetic analysis only. But this is a usual procedure.

3.Thank you for stating the following financial disclosure:
(No)

At this time, please address the following queries:

a) Please clarify the sources of funding (financial or material support) for your study. List the grants or organizations that supported your study, including funding received from your institution.

ANSWER: the study was only supported by my institution; in such I am employee. No additional funding exists. We do not use to put that in Acknowledgements, as looks dispensable.

b) State what role the funders took in the study. If the funders had no role in your study, please state: “The funders had no role in study design, data collection and analysis, decision to publish, or preparation of the manuscript.”

ANSWER: okay. Stated in M&M (paragraph 5).

c) If any authors received a salary from any of your funders, please state which authors and which funders.

ANSWER: not applicable.

d) If you did not receive any funding for this study, please state: “The authors received no specific funding for this work.”

ANSWER: okay. Stated in M&M (paragraph 5).

Please include your amended statements within your cover letter; we will change the online submission form on your behalf.

3. Thank you for stating the following in your Competing Interests section:
(NO authors have competing interests)

Please complete your Competing Interests on the online submission form to state any Competing Interests. If you have no competing interests, please state "The authors have declared that no competing interests exist.", as detailed online in our guide for authors at http://journals.plos.org/plosone/s/submit-now
This information should be included in your cover letter; we will change the online submission form on your behalf.

4.Please take this opportunity to be sure you have met all of our guidelines for new species. When publishing papers that describe a new botanical taxon, PLOS aims to comply with the requirements of the International Code of Nomenclature for algae, fungi, and plants (ICN). In association with the International Plant Names Index (IPNI), the following guidelines for publication in an online-only journal have been agreed such that any scientific botanical name published by us is considered effectively published under the rules of the Code. Please note that these guidelines differ from those for zoological nomenclature, and apply only to seed plants, ferns, and lycophytes.
Effective January 2012, "the description or diagnosis required for valid publication of the name of a new taxon" can be in either Latin or English. This does not affect the requirements for scientific names, which are still to be Latin.
Also effective January 2012, the electronic PDF represents a published work according to the ICN for algae, fungi, and plants. Therefore the new names contained in the electronic publication of a PLOS ONE article are effectively published under that Code from the electronic edition alone, so there is no longer any need to provide printed copies.
For proper registration of the new taxon, we require two specific statements to be included in your manuscript.
a) In the Methods section, include a sub-section called "Nomenclature" using the following wording:
The electronic version of this article in Portable Document Format (PDF) in a work with an ISSN or ISBN will represent a published work according to the International Code of Nomenclature for algae, fungi, and plants, and hence the new names contained

in the electronic publication of a PLOS ONE article are effectively published under that Code from the electronic edition alone, so there is no longer any need to provide printed copies.
In addition, new names contained in this work have been submitted to IPNI, from where they will be made available to the Global Names Index. The IPNI LSIDs can be resolved and the associated information viewed through any standard web browser by appending the LSID contained in this publication to the prefix http://ipni.org/. The online version of this work is archived and available from the following digital repositories: [INSERT NAMES OF DIGITAL REPOSITORIES WHERE ACCEPTED MANUSCRIPT WILL BE SUBMITTED (PubMed Central, LOCKSS etc)].
All PLOS ONE articles are deposited in PubMed Central and LOCKSS. If your institute, or those of your co-authors, has its own repository, we recommend that you also deposit the published online article there and include the name in your article.

b) In the Results section, the globally unique identifier (GUID), currently in the form of a Life Science Identifier (LSID), should be listed under the new species name, for example:
Solanum aspersum S.Knapp, sp. nov. [urn:lsid:ipni.org:names:77103633-1] Type: Colombia. Putumayo: vertiente oriental de la Cordillera, entre Sachamates y San Francisco de Sibundoy, 1600-1750 m, 30 Dec 1940, J. Cuatrecasas 11471 (holotype, COL; isotypes, F [F-1335119], US [US-1799731]).
PLOS ONE staff will contact IPNI to obtain the GUID (LSID) after your manuscript is accepted for publication, and this information will then be added to the manuscript at that time. You may indicate where the number or numbers will be added using XXXXX or an equivalent placeholder:
A complete explanation of our guidelines for publishing new species can be found on our website: http://www.plosone.org/static/guidelines#botanical

ANSWER: The present paper only deals with animal taxa, not botanic. For the ICZN, only the register in Zoobank http://www.zoobank.org/ is necessary for new taxa even in electronic (PDF) publications. This procedure was done in the present paper, and correspondent links are in Abstract and in each new taxon described.

5. We note that Figure 27 in your submission contain map images which may be copyrighted. All PLOS content is published under the Creative Commons Attribution License (CC BY 4.0), which means that the manuscript, images, and Supporting Information files will be freely available online, and any third party is permitted to access, download, copy, distribute, and use these materials in any way, even commercially, with proper attribution. For these reasons, we cannot publish previously copyrighted maps or satellite images created using proprietary data, such as Google software (Google Maps, Street View, and Earth). For more information, see our copyright guidelines: http://journals.plos.org/plosone/s/licenses-and-copyright.

We require you to either (1) present written permission from the copyright holder to publish these figures specifically under the CC BY 4.0 license, or (2) remove the figures from your submission:

a. You may seek permission from the original copyright holder of Figure 27 to publish the content specifically under the CC BY 4.0 license.

We recommend that you contact the original copyright holder with the Content Permission Form (http://journals.plos.org/plosone/s/file?id=7c09/content-permission-form.pdf) and the following text:
“I request permission for the open-access journal PLOS ONE to publish XXX under the Creative Commons Attribution License (CCAL) CC BY 4.0 (http://creativecommons.org/licenses/by/4.0/). Please be aware that this license allows unrestricted use and distribution, even commercially, by third parties. Please reply and provide explicit written permission to publish XXX under a CC BY license and complete the attached form.”

Please upload the completed Content Permission Form or other proof of granted permissions as an ""Other"" file with your submission.

In the figure caption of the copyrighted figure, please include the following text: “Reprinted from [ref] under a CC BY license, with permission from [name of publisher], original copyright [original copyright year].”

b. If you are unable to obtain permission from the original copyright holder to publish these figures under the CC BY 4.0 license or if the copyright holder’s requirements are incompatible with the CC BY 4.0 license, please either i) remove the figure or ii) supply a replacement figure that complies with the CC BY 4.0 license. Please check copyright information on all replacement figures and update the figure caption with source information. If applicable, please specify in the figure caption text when a figure is similar but not identical to the original image and is therefore for illustrative purposes only.
The following resources for replacing copyrighted map figures may be helpful:

USGS National Map Viewer (public domain):
http://vieweAnswer:nationalmap.gov/viewer/
The Gateway to Astronaut Photography of Earth (public domain):
http://eol.jsc.nasa.gov/sseop/clickmap/
Maps at the CIA (public domain): https://www.cia.gov/library/publications/the-world-factbook/index.html and https://www.cia.gov/library/publications/cia-maps-publications/index.html
NASA Earth Observatory (public domain): http://earthobservatory.nasa.gov/
Landsat: http://landsat.visibleearth.nasa.gov/
USGS EROS (Earth Resources Observatory and Science (EROS) Center) (public domain): http://eros.usgs.gov/#
Natural Earth (public domain): http://www.naturalearthdata.com/

ANSWER: this Figure 27 is not copyrighted. I drew it by myself.

[Note: HTML markup is below. Please do not edit.]

Reviewers' comments:

Reviewer's Responses to Questions

Comments to the Author

1. Is the manuscript technically sound, and do the data support the conclusions?

The manuscript must describe a technically sound piece of scientific research with data that supports the conclusions. Experiments must have been conducted rigorously, with appropriate controls, replication, and sample sizes. The conclusions must be drawn appropriately based on the data presented.

Reviewer #1: Partly

Reviewer #2: Yes

Reviewer #3: Yes

---

2. Has the statistical analysis been performed appropriately and rigorously?

Reviewer #1: N/A

Reviewer #2: I Don't Know

Reviewer #3: Yes

---

3. Have the authors made all data underlying the findings in their manuscript fully available?

The PLOS Data policy requires authors to make all data underlying the findings described in their manuscript fully available without restriction, with rare exception (please refer to the Data Availability Statement in the manuscript PDF file). The data should be provided as part of the manuscript or its supporting information, or deposited to a public repository. For example, in addition to summary statistics, the data points behind means, medians and variance measures should be available. If there are restrictions on publicly sharing data—e.g. participant privacy or use of data from a third party—those must be specified.

Reviewer #1: Yes

Reviewer #2: Yes

Reviewer #3: Yes

---

4. Is the manuscript presented in an intelligible fashion and written in standard English?

PLOS ONE does not copyedit accepted manuscripts, so the language in submitted

articles must be clear, correct, and unambiguous. Any typographical or grammatical errors should be corrected at revision, so please note any specific errors here.

Reviewer #1: Yes

Reviewer #2: Yes

Reviewer #3: Yes

---

5. Review Comments to the Author

Please use the space provided to explain your answers to the questions above. You may also include additional comments for the author, including concerns about dual publication, research ethics, or publication ethics. (Please upload your review as an attachment if it exceeds 20,000 characters)

Reviewer #1: I have read the manuscript and the reviews with the Author's comments. The manuscript arouse a lively discussion between the Author and the Reviewers, the tension between them was clearly visible. Therefore, I hope my opinion will be helpful. After I read the manuscript, I had an impression it should be sent (after improvements mentioned below) rather to some purely taxonomic journal more than to PLOSone. I also think, the paper has rather poor chances to be cited. The Author has 165 papers indexed by SCOPUS, which is a huge number, of course. However, the h-index is 15. It is not much for such a numerous bibliography (sorry to rise this issue, but it was mentioned in the comments that the Author published 300 papers therefore I decided to check the indexing).

ANSWER: I am very grateful for the worries about my low Scopus H index. I am worried too, as much that I decided to publish in higher impact journals, like Plos-One in recent years. I have managed to do this and have recently published, for example, in Plos One itself, Cladistics, Zoological Journal, etc. (check in link below).

In respect to the previous submission, I think we must argue in scientific and polite ways in every criticism. If some clear tension was visible, I am sorry. That was not my intention.

The Scopus database is not the best one to be used in Zoology, it underestimates the zoologist's production. As demonstrated by the present referee, Taxonomy does not enjoy the highest prestige, thus, malaco-taxonomists use to publish in journals that are weakly covered by Scopus. This diminishes the Scopus' H index, but that does not necessarily indicate a bad or low-quality production. As it can be checked in http://lattes.cnpq.br/5020466945403853, I am the author of 274 papers published in peer-reviewed journals, 4 books, 38 book chapters, 50 popular papers, etc. Counting only these categories, I am the author of 366 published items. My H-index, for example, in Google is 22, and i10 index is 72. I recognize these are low if compared to applied areas like Medicine, Engineering, etc. But, for a fairer trial, they must be compared to other zoologists. In this scenario, I am far above average.

I think an explanation on the importance of the Taxonomy in biological areas, as it is at the base of any wildlife studies, is not necessary here. But it is important to emphasize that the present paper is not purely taxonomic. It also has lots of information and discussion on morphology, anatomy, biogeography, phylogeny, environmental issues, etc. All of them increase the chance of citations, as are almost inedit. The papers that go beyond the taxonomy difficultly are accepted in taxonomic journals, and are more indicated to journals on broader subjects, like Plos'.

In addition, to underestimate someone else's scientific field, his production, and to check his H-index in a report on his paper is not usual. This is hard to interpret except as a personal problem.

Graphs and photos are nicely done. I think the Author put a lot of effort to prepare the documentary. However, in my opinion, the "scale" problem was not solved properly. I do agree with the Reviewer that the scale is needed and helpful for a potential reader, and does not disturb the plates' aesthetics (which is a secondary issue, anyway). However, drawing just a line in on the graph and providing in the figure caption that e.g. "Scales= 2 mm" is vague. It would be better to provide a number next to the line in the graph, with additional information about the unit in the caption e.g. "Scale bars in mm". This is of course just a small point, which is easy to be solved.

ANSWER: I understand the present suggestion and I can attend it if the Editors also agree with it. However, as only this referee suggested that, and as do not put scale bars in images of entire shell is traditional in Malacology goo papers (they are put only in details, and in anatomical stuff, as done in present paper), I ask permission to maintain the plates as they are. I understand that scales in all images with a number on them have their advantages, but they are terrible for the esthetic of the plate. And esthetic and beauty of the illustrations are equally important, mainly in a wide journal like Plos. I emphasize that nothing is missing in present way of presentation, as all pertinent information are in the legends.

I also think, the Author should provide specification of the optical equipment used for all measurements and photographic documentation.

ANSWER: Done (beginning of paragraph of "Methods and scope").

About the molecular analyses – I also agree with the Reviewer that such the study should be done and presented. The Author mentioned in the Introduction that molecular analyses are scheduled for the future. Such the statement indicate that the study are not finished. Molecular analyses may bring some interesting results changing the currently presented results. I would rather recommend to perform the analyses and presented them with conchological and anatomical approach in a single manuscript.

ANSWER: The present referee agrees that additional molecular approach is worthy, but the other two referees do not, the Reviewer 2, for example, clearly stated that "I do not feel molecular work is necessary for new species descriptions (at least in molluscs)", and the Reviewer 3 neither comment on that. Thus, is there an antagonism between the two opinions, with 2x1 score.

I agree that the inclusion of molecular results would give more data for discussions, as well as any other myriad of additional approaches that could be added (e.g., histological, physiological, ecological, etc.). However, we, I think, need to define the scope of every paper and show the best scenario and result based on that. As the Science progresses, other approaches and scenarios will be added, and the conclusions can or cannot change.

Thus, I ask for permission to maintain the paper based "only" on conchology, anatomy, morphology, biogeography and phylogeny of these taxa, expressing the highest respect to the Reviewer 1 opinion. I repeat that most (more than 90%) of the molluscan taxa were defined only by their shells, and the present paper goes further beyond that.

I also feel that providing an information that "Shell about 40 mm" is too general.

ANSWER: Malacologists usually apply a still more general way, calling the shell "large", "medium" or "small", which is further subjective. This way, putting an approximate average size, I think is more scientific. Additional explanations are in M&M.

The Author should prepare a summarizing table (or two tables – one for conchological traits, the second – for anatomical traits) containing the number of shells and specimens analyzed, with the measurements range for each of the characteristics, mean value, standard deviation, as well as measurements for a holotype. Such the table would made easier to find the data about the number of shell measured and specimens dissected, as the information is, indeed, lost in the text.

ANSWER: Done.

The Author did not provide a number of dissected snails for all the species. In the main text I found the information on 9 specimens of A. multicolor and 2 specimens of C. uhlei, while figures containing anatomical characteristics are given for A. multicolor, A. synchondrus, A. vailanti, A. morenus, A. savanticus, and C. uhlei (In the caption of the Fig. 11 the Author mentioned that he used 2 specimens, but no data is available within the main document).

ANSWER: This is not totally accurate. A complete list of "Material examined" follow all descriptions. It has all examined "specimens" (i.e., shell + dissected) or "shells" of every species. In the new species, the lists include the type material, as indicated. The list of material examined are the examined assembly of specimens.

Overall recommendation: Although the Author put lot of effort to prepare the manuscript, I cannot recommend the paper to be published in PLOSone. In my opinion, the material presented is more adequate for some purely taxonomic journal. Apart of this suggestion, I think the data are too scanty - molecular analyses pending. The data are presented vaguely. I also feel that some summarization of all measurements, containing data on all material analyzed is extremely needed.

ANSWER: Everything attended.

Reviewer #2: Dear author and editor - I would suggest this paper is accepted for review with some very minor revisions.

The paper is a well written description of the new species and an important addition to our knowledge of Molluscs from this group/region. I have made a few suggestions to the text to make the article clearer to the readers and there are a few points I have asked for clarification where the meaning is not clear to me. Unlike previous reviewers I do not feel molecular work is necessary for new species descriptions (at least in molluscs) where there is a history and ongoing precedent of species descriptions based on shell only let alone anatomical characters. I thought the phylogenetic work was a nice addition to the paper.

The species descriptions are done to a high standard and are easily understandable to the malacological community. May I suggest a short (2-3 sentences) 'differential diagnosis' added to the end of each description to succinctly allow this species differences to be highlighted.

I feel that the discussion is very thorough but my feeling is it is overly long and complex and by adding some 'differential diagnosis' to the species and generic descriptions this could be condensed and simplified.
ANSWER: All species already have the initial item "diagnoses", which brings the set of character that effectively differentiates the taxon from the others. All worthy characters are also reported in the Discussion, presented in a comparative scenario. I ask if would another item "differential diagnosis" put at end of the description be redundant? This is not a negative. If my argumentation is not convincing, I can easily obey this suggestion.

In the 'specific debate' there is a lot of shell information which whilst useful is difficult to follow. I wonder if a table showing data such as aperture size, W/L index and spire angle etc. could be used to shorten and simplify this?
Possibly more difficult but again could a table be used to highlight anatomical differences to shorten/simply or even add to the data presented.
ANSWER: done

I found the 'anatomical and broader taxonomic' section very informative and well structured.
ANSWER: Thanks.
I am not a phylogenetic specialist but the addition of this data was interesting and understandable to me.
ANSWER: Thanks.
The references are well structured and wide spread and the figures are well produced and clearly understandable. I do prefer scale bars in figures but I understand that when so many images are made available it would not be applicable in such a layout without it appearing messy.
ANSWER: Thanks.
If the author or editor have any further questions I am happy to contacted.

Reviewer #3: The revised ms is much improved, and has greatly benefited from inclusion of the anatomical data for the type species of Anthinus.
ANSWER: Thanks.

No page numbering on ms. Main comments refer to sections of text identified by numbers pencilled in the left margin of the annotated ms.
ANSWER: Sorry. Page numbers inserted.
Abstract
1. "A bulimulid, a simpulopsid and two dorcasiids are outgroups."
2. "Mirinaba and Anthinus also resulted monophyletic (3 and 6 synapomorphies respectively."
Suggest changing to "Mirinaba and Anthinus were also supported as monophyletic (3 and 6 synapomorphies respectively).
ANSWER: Done.
Introduction
3. "The description of the type species is, the remaining descriptions and illustrations, to minimize redundancy, are mostly presented compared to it."
Sentence ambiguous. Re-work.
ANSWER: Done.
4. "The anatomical base of all of them will certainly be useful for future taxonomic revisions and for phylogenetic inferences."
Suggest modifying as
The anatomical information presented on these new taxa will certainly be useful for future taxonomic revisions and for phylogenetic inferences.
ANSWER: Done.
5. "Despite these aspects are part of a large revisional project in such this study is inserted, an initial phylogenetic treatment is provided in this paper, based on the species with anatomy more detailly known in the literature and in the herein studied species. The main intention is not to present a "phylogeny of the Strophocheilidae", as many more species would be necessary, but to provide a better scenario for a discussion and the base for the new taxa.
Suggest the following changes
Despite these aspects are part of a large revisional project in such this study is inserted, An initial phylogenetic treatment is provided in this paper, based on species for which anatomy is reasonably well known in the literature, coupled with new information on the species herein erected. The main intention is not to present a "phylogeny of the Strophocheilidae", as anatomical investigation many more species would be necessary, but as the basis for erection of the new taxa, and to provide a better scenario for a comparative discussion.
ANSWER: Done.

Materials and methods

6. "The anatomical drawings are an average of all examined specimens. Color photos were obtained by digital cameras, either hand-held or attached to the dissecting microscope. Shell measurements were obtained with a digital caliper, in a minimum of 10 adult shells. The specimens were dissected by standard techniques [13] under dissecting stereomicroscopes, with the specimen immersed under the fixative. All drawings were obtained with the aid of a camera lucida; initially pencilled, afterwards inked; usually drawings produced for each species include data derived from several specimens. The type and voucher…."
Suggest a slight restructure of paragraph
Color photos were obtained by digital cameras, either hand-held or attached to the

dissecting microscope. Shell measurements were obtained with a digital caliper, for a minimum of 10 adult shells. The specimens were dissected by standard techniques [13] under dissecting stereomicroscopes, with the specimen immersed under the fixative. All drawings were obtained with the aid of a camera lucida; initially pencilled, afterwards inked; usually drawings produced for each species include data derived from several specimens. Thus, the anatomical drawings are an average (composite) of all examined specimens. The type and voucher ….
ANSWER: Done.
There is a potential issue arising with presentation composite illustrations rather than illustrating data gathered from individual specimens. Potentially information is lost on variation (if any) among the examined specimens. As a consequence, if there was variation between the examined type specimen [the name bearing specimen sensu Code of Zoological Nomenclature] and other examined specimens [non-name-bearing paratypes, topotypes, etc] then it may be ambiguous as to whether the composite encompasses anatomical information drawn from more than one species, such as can occur if one or more specimens were misidentified as belonging to the species in question. Is ambiguity can only be reduced if there is illustration of anatomical features for all examined specimens, or if the author explicitly states there was insignificant or no variation among these specimens.
Where only non-name-bearing specimens are examined (e.g. paratypes, topotypes, etc) then there will always be uncertainty as to whether this information also pertains to the species as defined by the name-bearing holotype.

ANSWER: This is a very interesting argumentation, which made me think. However, the anatomical illustrations, as well as the description/diagnosis, etc., are related to the SPECIES, and not of a specific specimen. Of course, some authors use to state "description of the holotype" as a formal species' description. I do not apply that way as it is in disuse. I prefer to show an average character referent to the species, based on all specimens, in descriptions and illustrations, rather than simply show the exact features of the holotype. For sure, any important variation must also be reported, as well as, in that case, which variation is the holotype's one. I do report these cases in my papers, but in the present paper the anatomical variation among the specimens can be classified as "insignificant". I introduced this information in M&M (line 12).

7. "… The collector team works for a company called Femorale [www.femorale.com; http://www.femorale.com/femorale/index.asp]. The places of collection are not protected areas, which do not require special permits. The collections, anyway, are supported by the general/permanent license IBAMA-Sisbio 10560-2, which permits extraction of wildlife samples for scientific purposes. …."
Suggest the following:
The material studied and described in this work was collected by a team working for Femorale, a private company [www.femorale.com; http://www.femorale.com/femorale/index.asp]. The places of collection are not within protected areas, and as such collection activity did not require special permits. Nonetheless, the collections were made under general/permanent license IBAMA-Sisbio 10560-2, which permits extraction of wildlife samples for scientific purposes.
ANSWER: Done.

8. "Thus, details on vegetation, climate, soil, rainfall, etc., were not available, but, when relevant, these data were extracted from the literature or official websites…"

Suggest
Thus, details on vegetation, climate, soil, rainfall, etc., were not available, but, when possible and relevant, these data were extracted from the literature, digital online resources, or official websites.
ANSWER: Done.

9. “For comparison of the presently studied species with those already known, the large MZSP collection was consulted, as a previous curator, DAnswer: José L.M. Leme, was a strophocheilid specialist.”
Suggest
For comparison of the presently studied species with those already known, the large MZSP collection was consulted, including the material assembled by DAnswer: José L.M. Leme, a previous curator and strophocheilid specialist.
ANSWER: Done.

10. “Although additional approaches can be added to any taxonomical paper, such as multivariate statistical analyses of shell measurements, molecular sequencing, etc., the presently presented anatomical investigation was so informative and convincing that it already bases very well the taxonomic conclusions. Of course, other approaches, molecular in particular, will be explored in future papers, considering an already well-based taxa provided by the present papeAnswer: It is important to emphasize that most of the land snails taxonomy is based only on their shells alone; the present paper goes further and presents convincing anatomical descriptions.”

This text has been added following adverse comments from previous reviewers, and is the author’s attempt to justify the methodology and pre-empt any future commentary. This text added by the author is unnecessary, redundant, as the published work should stand on its own merits.
ANSWER: Deleted.

11. “The chosen model to do the taxonomic remarks on each described species is reuniting all of them in a single Discussion, which ends this papeAnswer: This is interpreted as a more intelligible, fluent, and economic manner to explore the distinctions and similarities of the addressed taxa. Rather than individual remarks after each description.”
This text too was added by the author as a response to comments from previous reviewers. The criticism was not directed as the author’s model to aggregate the comparative remarks in the Discussion, but in the way that aggregation was done – too verbose, clumsy presentation of comparative morphometric information, lack of clarity about diagnostic features useful in species discrimination and identification, ….
I think this text in the main is redundant and should be replaced by a single sentence stating the fact that the taxonomic remarks are aggregated into the Discussion.
ANSWER: Ok. I redid that phrase for a simpler one. It is important to emphasize that all species bear a “Diagnosis” item, which already informs “about diagnostic features useful in species discrimination and identification”. The remaining parts of the paper are more concerned to discussion in taxonomic, comparative morphologic, and phylogenetic levels. These parts look verbose in a first view, but, as any scientific text, they must be complete, informative and exhaustive involving the literature.

12. “Phylogenetic analysis. In a family such as Strophocheilidae, …..
Elements of this text belongs in Results and Discussion. All that is needed here is a description of the methodology used.
“The phylogenetic methodology is the same as reported by Simone [13, 23], that basically consists of the matrix mounted in Nexus, analyzed by programs TNT and PAUP.” Is insufficient. Information needs to be added on phylogenetic analyses settings/search criteria etc. It is important that, given the data matrix, the results are reproducible [in as much as phylogenetic tree as estimates of a ‘true phylogeny’ are reproducible] based on the description given in Materials and Methods.
The Simone [13, 23] publications were consulted to determine what phylogenetic methodologies were used in those earlier publications. The description of the methods in Simone [13] are brief to the extreme, do not meet modern standards of reporting, and certainly are not informative about what analyses were actually undertaken for the present study, other than imply a maximum parsimony approach.
The link provided to Simone [23, Bosquejos de filogenia] only takes the reader to a small selection of pages that provide no relevant information. Evidently the full publication is not available for direct access on online, but can be requested.
ANSWER: Ok, fully corrected. Those initial phrases were transferred to the Discussion. The methodology was reported in a more direct manner as demanded.
In respect to the methodology of the phylogenetic analysis, it is really incredibly simple actually. No statistical juggle or maneuver were necessary. The given matrix is simply opened in the informed programs and read with minimum interference. The result is that presently shown, only that. I cannot see anything else to inform beyond what is presently informed or are present in the given references. Both are interesting to be reported: 13 has the technical and practical information (despite in being simple), 23 has the philosophical framework that I follow in phylogenetic analyses. All this given scenario permits the full reproducibility of the presented result, nothing is missing. Of course, if something else needs to be added, please, let me know, I put it quickly.

13. “Nomenclatural acts. The electronic edition of this article conforms …..”
This paragraph has been added by the author, apparent in response to some commentary by earlier reviewers about the merits or validity of erecting taxa in online/digital-only journals. Personally I think this text is unnecessary, but do share the concerns about taxa being erected in the digital-only format. In any event, the Commission has been strongly challenged about this aspect of the Code.
ANSWER: I totally agree, but this paragraph is a journal’s requirement.

14. “Material examined. Types (reported above).”
That is
“Types. Holotype MZSP 152074. Paratypes: MZSP 152252, 2 shells, MZSP 152075, 1 shell from type locality. BRAZIL. Minas Gerais; Unaí (W.V. Matos col, iv/2020), Gueda, 16°16’04”S 46°39’43”W, 647-650 m altitude, MZSP 152236, 1 shell.
Type locality. BRAZIL. Minas Gerais; Unaí, Pedra da Fartura, 16°31’37”S 46°49’18”W, 628- 750 m altitude [Weslley Vailant de Matos col, iv.2020].”
So which specimen(s) were used for the anatomical investigation?
ANSWER: All reported examined material was examined. Those all reported as “specimens” were dissected and studied both, in their anatomy and conchology. Those reported as “shells” were only examined in their conchology. Is necessary to report this in M&M?

Discussion

15. “The taxon descriptions presented above include comparative remarks, which shows the detailed differences amongst the taxa herein studied. This is particularly evidenced in given diagnoses. The present section is, thus, more focused on the most interesting features and those more relevant to taxonomy and present/future phylogenetic approaches to resolving relationships among South American strophocheilids.” Contrary to the author’s statement, the taxon descriptions do not include comparative remarks. Apparently this is a legacy of an earlier version of the ms.

ANSWER: I am sorry. It was rephrased.

16. “…The anatomical features were fundamental to determine both as separate genera.”

‘both as separate genera’ is grammatically lazy. Better would be ‘… to determine these are distinct genera’.

ANSWER: Done.

17. “… Both, A. multicolor and C. uhlei, share short, blunt mesocones, particularly those of the rachidian teeth …”

‘both’ redundant. Delete.

ANSWER: Done.

18. “… The present discussion focuses in the characters, every numerical parameter is based on a N from 10 to 20 specimens and is an integer value. Related to the shell elongation, A. multicolor is the slenderer (width/length W/L index = 46%) (Figs. 1A–C, 25C), with a group orbiting W/L ~50%, such as A. synchondrus (Fig. 25E – 54%), A. albolabiatus (Fig. 25A – 49%), A. miersi (Fig. 25B – 51%) and A turnix (Fig. 25D – 55%); while another group has shells broader, encompassing species with W/L = 60%, such as A. vailanti, A. morenus (Fig. 25G) and A. savanicus (Fig. 25H). In respect to the spire angulation, A. synchondrus has the sharpest pointed spire, ~35°, followed by A. multicolor ~40°, and A. albolabiatus ~42°; A, turnix has the broader spire, with ~58°, followed by A. miersi ~55°; while the remaining species (A. morenus, A. vailanti and A. savanicus) have ~50° …..”

In this paragraph, and to a least extent in subsequent paragraphs, the author has persisted with the same presentation style criticised by reviewers of the earlier version of the ms. Thus this section of the discussion continues to suffer in being too verbose, clumsy presentation of comparative morphometric information, lack of clarity about diagnostic features useful in species discrimination and identification, etc. Suggestions on some alternative formats for presentation were suggested earlier, but have not been taken up. This is the author’s call, but at some detriment to clarity.

ANSWER: It is important to emphasize that the set of characters useful in species discrimination and identification are already in the Diagnoses. Diagnoses has just this function, and are one of the ICZN requirements. I do not see the necessity to repeat those in the Discussion. This item is, I wonder, more concerned to comparations and discussions. They are usually read by a colleague that deals with these or related taxa. There is no way to make the text nicer in a technical report like this. Trying to attend this criticism, I introduced the Tables 1 and 2, that show the more important conchological (Table 1) and anatomical (Table 2) characters in synthetic way. However, the insertion of the table does not eliminate the need of that text.

6. PLOS authors have the option to publish the peer review history of their article (what does this mean?). If published, this will include your full peer review and any attached files.

If you choose "no", your identity will remain anonymous but your review may still be made public.

Do you want your identity to be public for this peer review? For information about this choice, including consent withdrawal, please see our Privacy Policy.

Reviewer #1: No

Reviewer #2: **Yes:** Jonathan Ablett

Reviewer #3: **Yes:** Gary M. Barker

[NOTE: If reviewer comments were submitted as an attachment file, they will be attached to this email and accessible via the submission site. Please log into your account, locate the manuscript record, and check for the action link "View Attachments". If this link does not appear, there are no attachment files.]

While revising your submission, please upload your figure files to the Preflight Analysis and Conversion Engine (PACE) digital diagnostic tool, https://pacev2.apexcovantage.com/. PACE helps ensure that figures meet PLOS requirements. To use PACE, you must first register as a useAnswer: Registration is free. Then, login and navigate to the UPLOAD tab, where you will find detailed instructions on how to use the tool. If you encounter any issues or have any questions when using PACE, please email PLOS at figures@plos.org. Please note that Supporting Information files do not need this step.
