# Supplementary material for: Additions to the genus Anthinus occurring in Minas Gerais and Goiás regions, Brazil, with description of five new species, one of them in the new related genus Catracca (Gastropoda, Eupulmonata, Strophocheilidae)
Source: PLoS One. 2022 Aug 24;17(8):e0273067. doi: 10.1371/journal.pone.0273067 (PMC9401170; doi:10.1371/journal.pone.0273067)
Supplement: Attachment — Submitted filename: PONE-D-22-00958_R1_reviewer_JDA.pdf [file pone.0273067.s005.pdf]

PLOS ONE

Additions to the genus Anthinus occurring in Minas Gerais and Goiás regions, Brazil, with description of five new species, one of them in the new related genus Catracca (Gastropoda, Eupulmonata, Strophocheilidae).

--Manuscript Draft--

Manuscript Number:	PONE-D-22-00958R1
Article Type:	Research Article
Full Title:	Additions to the genus Anthinus occurring in Minas Gerais and Goiás regions, Brazil, with description of five new species, one of them in the new related genus Catracca (Gastropoda, Eupulmonata, Strophocheilidae).
Short Title:	Additions to Anthinus and new Catracca (Strophocheilidae) in Brazil
Corresponding Author:	Luiz Ricardo L. Simone, Dr.<br>Museu de Zoologia da Universidade de Sao Paulo<br>São Paulo, São Paulo BRAZIL
Keywords:	Mollusca; Pulmonata; morphology; anatomy; taxonomy; systematics; Brazil; Biogeography; phylogeny; evolution
Abstract:	Four new species of the strophocheilid genus Anthinus are described from Brazil, being A. synchondrus from region of Unaí, Minas Gerais; A. vailanti from the region of Brasilândia de Minas, Minas Gerais; A. morenus from Paracatu region, Minas Gerais; and A. savanicus from Formosa region, Goiás. Another similar snail from Itacarambi, Minas Gerais, is described as a new genus and species: Catracca uhlei . All species are described conchologically and anatomically, with distinctions explored in almost all structures. For comparative purposes, a similar anatomical investigation on the Anthinus type species, A. multicolor , from Rio de Janeiro, is also performed. A detailed comparative analysis and discussion is provided, including taxonomic and initial phylogenetic inferences. The preliminary phylogenetic analysis is based on anatomically known species in the literature and in the presently study. A bulimulid, a simpulopsid and two dorcasiids are outgroups. The preliminary cladogram already shows a monophyletic Strophocheilidae (14 synapomorphies), divided into two also monophyletic subfamilies: Megalobuliminae (14 synapomorphies) and Strophocheilinae (5 synapomorphies). The new Catracca resulted as the most basal strophocheilid branch. Mirinaba and Anthinus were also supported as monophyletic (3 and 6 synapomorphies respectively. Register ZooBank: urn:lsid:zoobank.org:pub:FC4DD323-EF6A-404B-9755-F124F9DBB6D4
Order of Authors:	Luiz Ricardo L. Simone, Dr.
Response to Reviewers:	I thank you and the three referees for the important comments that improved the present paper. This is the fourth round of submission, and this new reformulated paper is very different from the first version, result of the comments of all 6 reviewers. I think this new version is finally worthy for publication. However, if something is still missing, or some of the few disagreement points do not meet the Editorial requirement or judgement, I am available for changing. There is no need to refuse the publication because of them.<br>Additionally, I have no special funding for this study in particular. It was developed only supported by my own laboratory. This fact we do not use to report, instead if required.<br>Additionally, I have no competing interest in any point.<br>Answers to the last email (April 1, 2022, by Rose Puetes) are in red in the cover-letter. But, note that most of them were already answered in "answers to reviewers" of the previous submission.
Additional Information:	
Question	Response
**Financial Disclosure**<br><br>Enter a financial disclosure statement that describes the sources of funding for the	There is no special fund supporting totally or partially the study that generated the present paper. I performed it in my job in the University of São Paulo. The institution contributed with my salary and the usual infrastructure. Is it necessary to inform that? I think that is obvious for any researcher. If so, please, tell me where I inform that

Powered by Editorial Manager® and ProduXion Manager® from Aries Systems Corporation

work included in this submission. Review the submission guidelines for detailed requirements. View published research articles from *PLOS ONE* for specific examples.<br><br>This statement is required for submission and **will appear in the published article** if the submission is accepted. Please make sure it is accurate.<br><br>**Unfunded studies**<br>Enter: *The author(s) received no specific funding for this work.*<br><br>**Funded studies**<br>Enter a statement with the following details:<br>• Initials of the authors who received each award<br>• Grant numbers awarded to each author<br>• The full name of each funder<br>• URL of each funder website<br>• Did the sponsors or funders play any role in the study design, data collection and analysis, decision to publish, or preparation of the manuscript?<br>• **NO** - Include this sentence at the end of your statement: *The funders had no role in study design, data collection and analysis, decision to publish, or preparation of the manuscript.*<br>• **YES** - Specify the role(s) played.<br><br>* typeset	I have not received any salary of funders, only from my job
**Competing Interests**<br><br>Use the instructions below to enter a competing interest statement for this submission. On behalf of all authors, disclose any competing interests that could be perceived to bias this work—acknowledging all financial support and any other relevant financial or non-financial competing interests.<br><br>This statement is required for submission and **will appear in the published article** if the submission is accepted. Please make sure it is accurate and that any funding sources listed in your Funding Information	NO authors have competing interests

later in the submission form are also declared in your Financial Disclosure statement.<br><br>View published research articles from *PLOS ONE* for specific examples.<br><br>**NO authors have competing interests**<br><br>Enter: *The authors have declared that no competing interests exist.*<br><br>**Authors with competing interests**<br><br>Enter competing interest details beginning with this statement:<br><br>*I have read the journal's policy and the authors of this manuscript have the following competing interests: [insert competing interests here]*<br><br>* typeset	
**Ethics Statement**<br><br>Enter an ethics statement for this submission. This statement is required if the study involved:<br><br>• Human participants<br>• Human specimens or tissue<br>• Vertebrate animals or cephalopods<br>• Vertebrate embryos or tissues<br>• Field research<br><br>Write "N/A" if the submission does not require an ethics statement.<br><br>General guidance is provided below. Consult the submission guidelines for detailed instructions. **Make sure that all information entered here is included in the Methods section of the manuscript.**	Not applicable

**Format for specific study types**<br><br>**Human Subject Research (involving human participants and/or tissue)**<br>• Give the name of the institutional review board or ethics committee that approved the study<br>• Include the approval number and/or a statement indicating approval of this research<br>• Indicate the form of consent obtained (written/oral) or the reason that consent was not obtained (e.g. the data were analyzed anonymously)<br><br>**Animal Research (involving vertebrate animals, embryos or tissues)**<br>• Provide the name of the Institutional Animal Care and Use Committee (IACUC) or other relevant ethics board that reviewed the study protocol, and indicate whether they approved this research or granted a formal waiver of ethical approval<br>• Include an approval number if one was obtained<br>• If the study involved *non-human primates*, add *additional details* about animal welfare and steps taken to ameliorate suffering<br>• If anesthesia, euthanasia, or any kind of animal sacrifice is part of the study, include briefly which substances and/or methods were applied<br><br>**Field Research**<br><br>Include the following details if this study involves the collection of plant, animal, or other materials from a natural setting:<br>• Field permit number<br>• Name of the institution or relevant body that granted permission	
**Data Availability**<br><br>Authors are required to make all data underlying the findings described fully available, without restriction, and from the time of publication. PLOS allows rare exceptions to address legal and ethical concerns. See the PLOS Data Policy and FAQ for detailed information.	Yes - all data are fully available without restriction

A Data Availability Statement describing where the data can be found is required at submission. Your answers to this question constitute the Data Availability Statement and **will be published in the article**, if accepted.<br><br>**Important:** Stating 'data available on request from the author' is not sufficient. If your data are only available upon request, select 'No' for the first question and explain your exceptional situation in the text box.<br><br>Do the authors confirm that all data underlying the findings described in their manuscript are fully available without restriction?	
**Describe where the data may be found in full sentences. If you are copying our sample text, replace any instances of XXX with the appropriate details.**<br><br>• If the data are **held or will be held in a public repository**, include URLs, accession numbers or DOIs. If this information will only be available after acceptance, indicate this by ticking the box below. For example: *All XXX files are available from the XXX database (accession number(s) XXX, XXX.*).<br>• If the data are all contained **within the manuscript and/or Supporting Information files**, enter the following: *All relevant data are within the manuscript and its Supporting Information files.*<br>• If neither of these applies but you are able to provide **details of access elsewhere**, with or without limitations, please do so. For example:<br><br>*Data cannot be shared publicly because of [XXX]. Data are available from the XXX Institutional Data Access / Ethics Committee (contact via XXX) for researchers who meet the criteria for access to confidential data.*<br><br>*The data underlying the results presented in the study are available from (include the name of the third party*	All relevant data are within the manuscript only

and contact information or URL).<br>• This text is appropriate if the data are owned by a third party and authors do not have permission to share the data.<br><br>* typeset	
Additional data availability information:	

Powered by Editorial Manager® and ProduXion Manager® from Aries Systems Corporation

Corrected manuscript

Click here to access/download;Manuscript;2022 Anthinus Coltro Plos clean.docx

Additions to the genus *Anthinus* occurring in Minas Gerais and Goiás regions, Brazil, with description of five new species, one of them in the new related genus *Catracca* (Gastropoda, Eupulmonata, Strophocheilidae).

Luiz Ricardo L. Simone

Museu de Zoologia da Universidade de São Paulo, Cx. Postal 42391; 04218-970 São Paulo, SP, Brazil; lrsimone@usp.br; lrlsimone@gmail.com; OrcID: 0000-0002-1397-9823

Abstract

Four new species of the strophocheilid genus *Anthinus* are described from Brazil, being *A. synchondrus* from region of Unaí, Minas Gerais; *A. vailanti* from the region of Brasilândia de Minas, Minas Gerais; *A. morenus* from Paracatu region, Minas Gerais; and *A. savanicus* from Formosa region, Goiás. Another similar snail from Itacarambi, Minas Gerais, is described as a new genus and species: *Catracca uhlei*. All species are described conchologically and anatomically, with distinctions explored in almost all structures. For comparative purposes, a similar anatomical investigation on the *Anthinus* type species, *A. multicolor*, from Rio de Janeiro, is also performed. A detailed comparative analysis and discussion is provided, including taxonomic and initial phylogenetic inferences. The preliminary phylogenetic analysis is based on anatomically known species in the literature and in the presently study. A bulimulid, a simpulopsid and two dorcasiids are outgroups. The preliminary cladogram already shows a monophyletic Strophocheilidae (14 synapomorphies), divided into two also monophyletic subfamilies: Megalobuliminae (14 synapomorphies) and Strophocheilinae (5 synapomorphies). The new *Catracca* resulted as the most basal strophocheilid branch. *Mirinaba* and *Anthinus* were also supported as monophyletic (3 and 6 synapomorphies respectively. Register ZooBank: urn:lsid:zoobank.org:pub:FC4DD323-EF6A-404B-9755-F124F9DBB6D4

Introduction

The Strophocheilidae genus *Anthinus* Albers, 1850 ([1]:148) has as the type species *Helix multicolor* Rang, 1831 [2] (Subsequent Designation by Martens [3]: 189-190). In an early monographic treatment of Strophocheilidae, Bequaert ([4]: 196-202), provided detailed taxonomic history of the genus. *Anthinus* characteristically includes the smallest strophocheilids, with adult specimens with shells around 20-30 mm, usually having a tall, bluntly pointed spire, relatively small aperture in a “bulimoid-shape”, and a mosaic of brown-beige spots [4, 5]. All four valid *Anthinus* species were previously reviewed and illustrated by Simone ([6]: 201-202). Nonetheless, the published anatomical information on *Anthinus* has remained poor, restricted to sparse information and few illustrations [5, 7]. The geographic occurrence of *Anthinus* in the southeastern Brazilian Atlantic Rainforest environment, endorsed by Simone ([6]) was from the south region of Minas Gerais-Mato Grosso do Sul in Brazil (latitude ~15°S) to northern Argentina and Uruguay (latitude ~32°S). A doubtful occurrence in Tobago has been reported ([6] and references therein; [8]).

In recent years, some samples identified as *Anthinus* have been collected in expeditions by the naturalist José Coltro Jr. and colleagues, in regions from north of Minas Gerais, to Goiás (Brazilian midwest) and in several points in northeast Brazil (latitudes ~15°S to ~17°S). This material is used in the present paper. These regions have a dryer environment, including a dryer version of the Atlantic Rainforest, Cerrado and Caatinga semi-arid biomes.

Examination of this material revealed five undescribed species, more than doubling the number of presently known *Anthinus* species. Anatomical information on the type species, *A. multicolor*, collected in Rio de Janeiro, is also included for improving the comparative scenario. The shell of one of the new species, initially appeared to belong to a unicolored *Anthinus*. It has, however, a greatly different set of anatomical features, making necessary the description of a new genus. The description of the type species is presented first, the remaining descriptions and illustrations, to minimize redundancy, are mostly presented compared to it.

The family Strophocheilidae has had an unstable taxonomic history [4, 5], but usually it has been considered endemic from South America, with relatives in Africa – the Dorcasiidae; and in Asia – the Acavidae; all grouped in Acavoidea (e.g., [9]), or sometimes in Strophocheiloidea (e.g., [10]). South American members have traditionally been assigned to two families: Strophocheilidae and Megalobulimidae [5]. More recently these two families have been treated as subfamilies in Strophocheilidae, and, together with Acavidae and Dorcasiidae, placed in Rhytidoidea, which has five other families [11, 12].

This paper has as main objective the formal introduction of the new strophocheilids taxa. The anatomical information presented on these new taxa will certainly be useful for future taxonomic revisions and for phylogenetic inferences. Despite these aspects are part of a large revisional project in such this study is inserted, an initial phylogenetic treatment is provided in this paper, based on species for which anatomy is reasonably well known in the literature, coupled with new information on the species herein erected. The main intention is not to present a “phylogeny of the Strophocheilidae”, as anatomical investigation many more species would be necessary, but as the basis for erection of the new taxa, and to provide a better scenario for a comparative discussion.

Material and methods

Material. A complete list of material examined follows each species’ description. Material comprised of dry shells or shells and specimens preserved in 70% EtOH. All listed specimens were used to compose the descriptions; those that are only shells (sh) were examined at a conchological level; all complete specimens (shell and soft parts) (spm) were extracted and dissected.

Methods and scope. Color photos were obtained by digital cameras, either hand-held (Canon Digital Rebel XTi with macro-lenz) or attached to the dissecting microscope (Zeiss AxioCam ERc 5s). Shell measurements were obtained with a digital caliper, for a minimum of 10 adult shells. The specimens were dissected by standard techniques [13] under dissecting stereomicroscopes, with the specimen immersed under the fixative. All drawings were obtained with the aid of a camera lucida; initially penciled, afterwards inked; usually drawings produced for each species include data derived from several specimens, as the intraspecific variation has shown insignificant. Thus, the anatomical drawings are an average (composite) of all examined specimens. The scales were obtained putting a ruler by side of the specimen. The type and voucher material are deposited in Museu de Zoologia da Universidade de São Paulo (MZSP) malacological collection. The specimens usually are relatively easily extracted from their shells,

except for the holotype of *Anthinus synchondrus*, in this case a 'cesarean' (a small window) needed to be done in the last whorl. SEM work on the material is standard, with pieces metalized by gold, and examined in the Laboratory of Electron Microscopy of the MZSP. Anatomical terminology, particularly of the odontophore muscles, follows Simone [13]. The material studied and described in this work was collected by a team working for Femorale, a private company [www.femorale.com; http://www.femorale.com/femorale/index.asp]. The places of collection are not within protected areas, and as such collection activity did not require special permits. Nonetheless, the collections were made under general/permanent license IBAMA-Sisbio 10560-2, which permits extraction of wildlife samples for scientific purposes. As most of the studied material was collected by non-scientific expeditions, no further data beyond coordinates and place names were available. Thus, details on vegetation, climate, soil, rainfall, etc., were not available, but, when possible and relevant, these data were extracted from the literature, digital online resources, or official websites. For comparison of the presently studied species with those already known, the large MZSP collection was consulted, including the material assembled by Dr. José L.M. Leme, a previous curator and strophocheilid specialist. Collections of other European and American museums were also consulted in my trips seeking type specimens, some of them illustrated in a catalogue [6]. However, despite high effort, types of not all *Anthinus* species were found. In these cases, voucher material of previous revisions was preferentially studied. Despite having material for a total review of the genus *Anthinus* available, the previous Bequaert's review [4] is so well done that nothing actually new could be informed at shell level. Despite being relatively antique, Bequaert's review [4] is still valid in species' level, and few additional information was published on *Anthinus* after that. Thus, the present paper focuses on the description of the new taxa and the anatomical unpublished features. The type species, however, is included, extending to anatomical features and improving the comparative taxonomic scenario. The taxonomical remarks on each species are all reunited in the Discussion item.

Phylogenetic analysis. The phylogenetic methodology is the same as reported by Simone [13, 23], that basically consists of the matrix mounted in Nexus, analyzed by programs TNT and PAUP. All of them resulted in a single cladogram shown below. Thus, beyond the species herein studied, the present preliminary phylogeny is based upon already published data of 14 species listed below, as well as additional examination of their voucher material deposited in MZSP in some unstudied structures, such as, e.g., the odontophore. The list of characters is in Appendix 1; and respective matrix in Appendix 2. It is important to emphasize that the shown phylogeny is not to be interpreted as "the phylogeny of the Strophocheilid". It has only the intention of demonstrating that the description of the new genus – *Catracca* – is necessary. In the literature, a small set of strophocheilids has their anatomy known in sufficient details for an initial phylogenetic inference. They are (1) *Mirinaba antoninensis* (Morretes, 1952) [14, 15]; (2) *Mi. cadeadensis* (Morretes, 1952) [15]; (3) *Strophocheilus debilis* Bequaert, 1948 [5]; (4) *Megalobulimus lopesi* Leme, 1984 [16]; (5) *Me. grandis* (Martens, 1885) [16]; (6) *Me. parafragilior* Leme & Indrusiak, 1990 [17]; (7) *Me. proclivis* (Martens, 1888) [18]; (8) *Me. dryades* Fontenelle, Simone & Cavallari, 2021 [19]; (9) *Me. oblongus* (Müller, 1774) and (10) *Me. conicus* (Bequaert, 1948) [20]. As remote outgroups, a bulimulid (*Leiostracus carnavalescus* Simone & Salvador, 2016 [21]) and a simpulopsid (*Rhinus botocudus* Simone & Salvador, 2016 [21]), and close outgroups a pair of African dorcasiids (*Dorcasia alexandri* Gray, 1838 and *Trigonephrus porphyrostoma* (Melvill & Ponsonby, 1891) [22]) were chosen. All six species studied herein were also included.

Nomenclatural acts. The electronic edition of this article conforms to the requirements of the amended International Code of Zoological Nomenclature, and hence the new names contained herein are available under that Code from the electronic edition of this article. This published work and the nomenclatural acts it contains have been registered in ZooBank, the

online registration system for the ICZN. The ZooBank LSIDs (Life Science Identifiers) can be resolved and the associated information viewed through any standard web browser by appending the LSID to the prefix "http://zoobank.org/". The LSID for this publication is: urn:lsid:zoobank.org:pub:FC4DD323-EF6A-404B-9755-F124F9DBB6D4. The electronic edition of this work was published in a journal with an ISSN, and has been archived and is available from the following digital repositories: PubMed Central, LOCKSS, ResearchGate.

It is important to state that funders had no role in study design, data collection and analysis, decision to publish, or preparation of the manuscript. Addicionally, the author received no specific funding for this work.

Abbreviations in the Figures: **aa**, anterior aorta; **ac**, albumen chamber; **ad**, albumen gland duct; **af**, anal folded subterminal portion; **ag**, albumen gland; **an**, anus; **as**, accessory genital gland; **au**, auricle; **bc**, bursa copulatrix; **bd**, bursa copulatrix duct; **bg**, buccal ganglion; **bm**, buccal mass; **br**, subradular membrane; **bv**, blood vessel; **cc,** cerebral commissure; **ce**, cerebral ganglion; **cd**, cerebral node; **cn**, cerebro-pedal and cerebro-pleural connectives; **cv**, pulmonary (efferent) vein; **da**, digestive gland anterior lobe; **dc**, dorsal chamber of buccal mass; **dd**, gastric duct to digestive gland; **df**, dorsal folds of buccal mass; **dg**, digestive gland posterior lobe; **di**, diaphragm or pallial floor; **dl**, left esophageal duct to digestive gland; **dr**, right esophageal duct to digestive gland; **eh**, epiphallus; **eo**, spermoviduct; **es**, esophagus; **ey**, eye; **fo**, free oviduct; **fp**, genital pore; **ft**, foot; **gm**, genital muscle; **go**, gonad; **hd**, hermaphrodite duct; **hp**, hump of free oviduct; **in**, intestine; **jw**, jaw; **kf**, kidney anterior fold; **ki**, kidney; **lf**, lateral fold of buccal mass; **m1–m10**, extrinsic and intrinsic odontophore muscles; **mb**, mantle border (edge); **mf**, mantle fold; **mj**, jaw and peribuccal muscles; **mo**, mouth; **mp**, prerectal muscle; **ne**, nephrostome; **nr**, nerve ring; **oc**, odontophore cartilage; **od**, odontophore; **om**, ommatophore; **on**, optical nerve; **ou**, ommatophore muscle; **pa**, penis anterior chamber; **pc**, pericardium; **pe**, penis; **pf**, penis fold; **pg** pedal gland; **pm**, penis muscle; **pn**, pneumostome; **pp**, pedal ganglion; **pr**, penis aperture; **ps**, penis posterior chamber; **pt**, prostate; **pu**, pulmonary cavity; **pv**, pneumostome inner flap; **ra**, radula; **rn**, radular nucleus; **rs**, radular sac; **rt**, rectum; **sa**, salivary gland aperture; **sc**, subradular cartilage; **sd**, salivary gland duct; **se**, septum between esophagus and odontophore; **sf**, stomach inner fold; **sg**, salivary gland; **sh**, penis central sphincter; **sm**, secondary cephalic muscles; **sp**, sperm inner longitudinal fold; **sr**, seminal receptacle; **st**, stomach; **sw**, stomach muscular walls; **te**, ventral cephalic tentacle; **tg**, integument; **te**, tentacle; **tm**, tentacle muscle; **tn**, tentacular nerve; **un**, union of mantle border with nuchal surface; **ur**, urinary gutter; **ut**, uterus; **vd**, vas deferens; **ve**, ventricle; **vf**, vaginal fold; **vg**, vagina.

Additionally in the text, the following abbreviations are used: **L**, length; **sh**, empty dry shell; **spm**, complete specimen (shell and soft parts); **W**, width. Institutions: **MNRJ**: Museu Nacional da Universidade Federal do Rio de Janeiro; **MZSP**: Museu de Zoologia da Universidade de São Paulo; **USNM**: National Museum of Natural History, Smithsonian Institution.

Results

Comparative conchology and anatomy

Systematics

Genus *Anthinus* Albers, 1850

Bulimus (*Anthinus*) Albers, 1850[1]: 148; Martens, 1860[3]: 189.

Auris (*Anthinus*): Pilsbry, 1895[24]: 95.

Gonyostomus (*Anthinus*): Bequaert, 1948[4]: 196; Oliveira et al., 1968[8]: 10.

Anthinus: Morretes, 1949[25]: 145; Leme, 1973[5]: 332; Simone, 2006[6]: 201.

Diagnosis. Shell of ~30 mm, apex blunt, aperture ~half of shell length. Protoconch of 3-4 whorls, sculptured by delicate reticulation of spiral and axial lines. Color mosaic of brown, beige and cream wide spots, with tendency of spiral organization in squared irregular spots. Head-foot lacking labial flange. Lung with not-protruded vessels, dark pigmented. Odontophore pair m5 ~half originated from cartilages and ~half from m4, free from m2. Pair of anterior (esophageal) ducts to digestive gland, located posteriorly, practically from stomach; both components (dr, dl) with about same caliber, left duct much longer. Prerectal pallial muscle (mp) usually present. Anus turned externally in pneumostome right corner. Seminal receptacle strongly curved. Spermoviduct accessory gland (as) present. Penis with single inner chamber; epiphallus short, rounded, vas deferens insertion subterminal, inner surface papillate; inner main chamber with main pair of strong longitudinal folds, sulcus between these folds continuous to epiphallus. Other details in [4, 5].

List of included species. *Anthinus multicolor* (Rang, 1831) (type species); *A. albolabiatus* (Jaeckel, 1927); *A. miersi* (Sowerby, 1838); *A. turnix* (Gould, 1846); *A. synchondrus* new species; *A. vailanti* new species; *A. morenus* new species; *A. savanicus* new species.

Taxonomic discussion. see Discussion item.

Anthinus multicolor **(Rang, 1831)** Figs. 1-4

Previous synonymy see Bequaert (1948[4]: 196-197). Complement:

Helix (*Cochlogena*) *multicolor* Rang, 1831[2]: 55 (pl. 3, fig. 1).

Gonyostomus (*Anthinus*) *multicolor:* Bequaert, 1948[4]: 196-198 (pl. 4, fig. 5; pl. 9, figs 3, 7; pl. 13, fig. 6; Pl. 17, fig. 4).

Anthinus multicolor: Morretes, 1949[25]: 145; Leme, 1973[5]: 332; Abbott, 1989[26]: 76 (fig.); Salgado & Coelho, 2003[27]: 156; Simone, 2006[6]: 202 (fig. 760).

Gonyostomus multicolor: Oliveira et al., 1968[8]: 10; 1981[28]: 341.

Types. whereabouts unknown [4].

Type locality. São Paulo State and near Corcovado, Rio de Janeiro.

Diagnosis. Shell about 35 mm; color usual mosaic of brown and beige spots, lacking spiral alignment; spire angle ~40°, protoconch of 2.5 whorls; peristome ~52% of shell length and ~55% of width; width ~46% of length. Mantle edge with simple, rounded fold. Reno-pericardial area small-sized. Pre-rectal pallial muscle absent. Odontophore with cartilages ~90% fused with each other; pair m4 ~50% originated from cartilage, remaining on m4. Jaw plate broad, thick. Radula with short, hook-like mesocone, rachidian different from neighboring teeth. Seminal receptacle widely curved, hermaphrodite duct inserting in its base, curved. Accessory genital gland in spermoviduct small; with pair of similar-shaped sperm-grooves. Free oviduct with distal hump. Penis length ~50% of spermoviduct; penis muscle mostly inserted to penis' tip, with small branch to epiphallus and another small branch to adjacent vas deferens; epiphallus papillae tall, uneven,

extending along entire penis chamber; large pair of penial inner folds wide and low, covered by papillae.

Redescription

Shell. (Figs. 1A-E) proper description in [4]. Complement: Adult shell around 35 mm, conical-oval; apex blunt; wider on last whorl; width ~46% shell length. Walls relatively thin. Color sometimes entirely brown ([6]: 202), but usually with reddish beige spiral band along inferior half of each spire whorl, or central region of body whorl (Figs. 1A-C); white spots randomly splayed, overlapping remaining colors, usually coalescent near superior suture, and also inferior third of last whorl (Figs. 1B-C), barely forming irregular axial bands; small white punctiform spots splayed in inferior half of spire whorls and middle region of body whorl. Spire angle ~40°. Protoconch of 2.5 whorls (Figs. 1D-E), ~12% of length, width of ~6.1 mm, slightly taller than wide; first whorl smooth, gradually spiral narrow aligned punctuations appearing (Figs. 1D-E), lacking axial cords except for relatively regular axial undulations, ~3 per mm, last protoconch whorl with spiral sculpture more spaced, ~8 in last protoconch whorl. Teleoconch of ~2 whorls, profile slightly convex; sculpture similar to that of protoconch last whorl, spiral punctuations (10 in penultimate whorl) and axial undulations; last 1.5 whorls lacking spiral punctuations, only having smooth growth lines (Fig. 1C). Whorls profile weakly convex (Figs. 1A-C). Suture well-marked, not channeled (Fig. 1C). Aperture slightly prosocline (~10° from longitudinal general axis) (Fig. 1B), elliptic; ~52% of shell length, ~55% of shell width. Peristome continuous, reflected, thick, color red (Fig. 1A); outer lip arched, lacking middle tooth; inner lip with inferior half almost straight, superior half as broad, weak callus with ~2/5 of peristome length (Fig. 1A), weakly convex. Inferior half of inner lip covering umbilicus. Umbilicus relatively wide, ~10% of inferior area. Body whorl ~70% shell length; uniform with spire.

Head-foot. (Fig. 2B) of normal shape. Color uniformly pale. Columellar muscle thick, 1.5 whorls in length. Inner arrangement of columellar annexed muscles relatively complex. Main columellar bundle (cm) occupying ventral floor of haemocoel, relatively flat, wide slightly broader than half of foot width. Pair of secondary cephalic muscles (sm) with middle and anterior regions approximately double thickness and half width those of columellar main bundle (left muscle slightly broader than right one); originating gradually along dorsal surface of main columellar bundle, running anteriorly on main bundle; anterior third of each muscle with four parallel branches, two medial branches broad (~2/3 of its basal width) inserting in ventral region of perioral region. Branches 3 and 4 respectively tentacular and ommatophore muscles; ommatophore muscles (ou) narrower, mode lateral, inserting in tip of ommatophore; tentacular muscle (tm) longer, inserted in tip of tentacles. Pedal gland (pg) protruding in posterior region of buccal area.

Mantle organs. (Fig. 2A) Mantle border thick, lacking pigments. Pneumostome (pn) protected by simple right ventral flap (pv), width ~1/5 of aperture length. Dorsal fold well developed (mf), occupying ~1/3 of dorsal mantle edge length; rounded. Pneumostome (pn) ~1/10 of shell aperture length, bearing exclusively air entrance and urinary gutter. Anus (an) separate aperture located at right, adjacent to pneumostome. Lung of 1.2 whorls in length, wide and elongated; right side ~1.5 times longer than left side. Pulmonary venation well-developed; posterior region of pulmonary vein (cv) protruded; left half only having longitudinal vessels, right half mostly having perpendicular vessels, except for oblique vessels in region preceding pneumostome. Pulmonary vein (cv) running longitudinally across pallial cavity roof medially or towards right, anterior end trifurcated. Reno-pericardial area beige, slightly triangular, located posteriorly within pallial cavity, its posterior abutting wall of visceral cavity, occupying ~10% of cavity length and ~40% of its width (details below). Rectum (rt) wide. Urinary gutter (ur) broad, smooth, lacking clear transverse folds; running along rectum; anterior urinary gutter surrounding left anal aperture.

Visceral mass. (Fig. 2A) ~3 whorls in length. Both digestive gland lobes brown in color; anterior lobe (da) flattened, occupying ~1/5 of visceral volume, located just posteriorly to pallial cavity, continuous to kidney. Posterior lobe (dg) larger, extending 2 spiral whorls, occupying ~60% of visceral volume. Stomach ~1/15 of visceral volume, located between both digestive gland lobes, about 3/4 whorl posterior to pallial cavity (st). Digestive tubes (described below) surrounding anterior lobe of digestive gland. Gonad multi-lobed, cream color, encased between posterior lobe of digestive gland and columella, occupying ~1/3 whorl, ~1/10 of visceral volume.

Circulatory and excretory systems. (Fig. A) Pericardium (pc) ~twice as long as wide, located obliquely between middle and left thirds of posterior end of pallial roof, appressed against right lateral side of kidney; occupying ~5% of lung area. Auricle located anteriorly, as continuation from pulmonary vein (cv); ventricle located posteriorly, larger. Kidney (ki) simple, dorso-ventrally flattened; size reported above; somewhat triangular, width ~2/3 of length; internally organized as two distinct regions – a longitudinal hollow cavity on left; filled by sponge-like renal tissue, clearly U-shaped (convexity left) in section. Nephropore (ne) small, longitudinal slit at anterior-left corner of kidney, directed towards right; protected by curved anterior projection of kidney (kf). Urinary gutter (ur) reported above.

Digestive system. (Figs. 2A) Mouth (mo) and oral tube wide, muscular. Jaw plate (Figs. 1G-H) thick; cutting edge blunt, sigmoid; lacking inner reinforcement, immersed in jaw thickness. Buccal mass spherical, occupying~1/5 of haemocoel volume. Dorsal surface of oral cavity with well-developed pair of dorsal folds, width of each ~1/3 of dorsal wall width; separated from each other by dorsal chamber (dc) as wide as folds, but broader anteriorly. Odontophore (od) with ~60% of buccal mass volume. Odontophore muscles: **mj**, jaw and peribuccal muscles originating in outer-ventral surface of odontophore cartilages, running towards dorsal, splaying in dorsal wall of oral tube; **m1**, jugal muscles covering entirely haemocoelic structures, more concentrated close to mouth; **m1v**, small pair of ventral protractors jugal muscles, originating in ventral surface of haemocoel close to mouth, running towards posterior, inserting in ventral-posterior region of odontophore close to m2 insertion; **m2**, strong pair of retractor muscles of buccal mass, or radular muscles, originating as single bundle in columellar muscle posterior end, running anteriorly close to median line along ~60% of haemocoel length, inserting as two different bundles in ventro-posterior edge of odontophore, surrounding at some distance radular nucleus; **m3d**, thin layer of longitudinal fibers immersed in dorso-posterior wall of odontophore, preceding esophageal origin; **m4**, main pair of dorsal tensor muscles of radula, very thick, originating in postero-medial region of odontophore cartilages, surrounding outside and medially cartilages, inserting in subradular membrane in its region correspondent to buccal cavity; **m5**, pair of thick auxiliary dorsal tensor muscles of radula, originating half on posterior-medial surface of m4, and half on portion in postero-ventral region of odontophore cartilages, running towards median line covering m4, inserting in subradular membrane by side of m4 insertion; **m6**, horizontal muscle absent; **m7**, absent; **m10**, pair of narrow ventral odontophore protractor muscles, originating in ventro-anterior region of haemocoel, just ventral to mouth, running towards posterior along buccal mass length, inserting in latero-posterior surface of odontophore close to m2 insertions; **m11**, pair of narrow ventral tensor muscles of radula absent. Odontophore non-muscular structures: oc, pair of odontophore cartilages flattened, rather elliptical, anterior region slightly projected anteriorly close to median line, ~1.2 times longer than wide, fused with each other along ~90% in their anterior-medial edge, posterior end roughly rounded; **sc**, subradular cartilage, with expanding region in buccal cavity protecting subradular membrane. Radular sac short, not extending beyond odontophore.

Radula. (Fig. 3) Slightly longer than odontophore; with rachidian teeth, and ~30 pairs of lateral/marginal teeth; no clear distinction between lateral and marginal teeth (Fig. 3A), marginal teeth only slightly narrower than lateral teeth, and more inclined medially (Figs. 3A, B: left side);

all teeth with base as long as mesocone, articulating neighboring rows (Figs. 3C); mesocone arched, curved inwards and posterior, ~1.5 longer than wide, apex rounded, base reinforcement slightly more developed, subterminal; no secondary cusps. **Rachidian** tooth (Figs 3B, C: arrow) as large as lateral teeth; base ~twice longer than wide, slightly flattened, barely triangular, with arched posterior edge articulating with neighbor tooth and pair of distal reinforcements; mesocone reduced, ~half size of those of lateral teeth, rounded, covering ~1/3 of base; width and length similar to those of lateral teeth. **Lateral teeth** similar to rachidian, except in being slightly longer, longer asymmetrical mesocone, arched towards medial region; base trapezoid, with medial concavity articulating with neighbor medial tooth and single distal reinforcement (Fig. 3C). **Marginal teeth** starting with no clear boundary with lateral teeth (Figs. 3A, B), occupying ~1/3 of each side; shaped similarly to lateral teeth, except for being slightly narrower and more inclined medially, row practically straight (Fig. 3A: left side).

Salivary glands small, covering ~1/5 of esophagus length, in its anterior quarter region (Fig. 2A: sg), forming two rounded, white, thin masses. Each salivary duct differentiable in middle and anterior side of glands, with ~1/12 of esophageal width (sd). Salivary duct running in both sides of esophageal origin, penetrating buccal mass wall in region close to buccal ganglia, running immersed in buccal dorsal wall along ~1/3 its length. Salivary ducts opening as small slits in located in posterior region of lateral edges of wide dorsal folds.

Esophagus ~1-whorl long, with thin, flaccid walls (Fig. 2A: es); anterior 1/2 weakly broader, crop-like; inner surface simple, smooth. Stomach (st) narrow, curved, weakly bulging; position and size described above (visceral mass); gastric walls thin, weakly muscular; inner surface mostly smooth, but with chitinous inner cover well-developed. Esophageal insertion on right side, intestinal origin on left side, both close to columella. Duct to anterior lobe of digestive gland at short distance from esophagus and intestine intersection (dr) narrow, running shortly towards right, bifurcating; additional left duct (dl) originated in main duct base with ~1/3 of its width, running narrowly towards left and anterior along digestive gland lobe compressed by first intestinal loop (da), periodically possessing short ramifications along its length. Duct to posterior lobe of digestive gland located short distance from intestinal origin, posterior to above-described duct, directed towards opposite side (dd) as wide as anterior duct. Intestine (in) initially as wide as esophageal insertion, gradually narrowing up to ~half that width along its wide sigmoid loop in anterior lobe of digestive gland (in). Rectum and anus position described above (pallial cavity) (rt, an). Anus sessile, as slit in right end of mantle edge directly turned outside; inner surface with 8–10 simple longitudinal, relatively tall folds.

Reproductive system. (Fig. 2C, 4) Gonad position described above (visceral mass), composed of 8–10 lobes with minute digitiform acini. Hermaphroditic duct (Fig. 2C: hd) broad; coiled portions occupying anterior 2/3, with very strong coils; insertion strongly curved (Figs. 2C, 4B: hd). Seminal receptacle (Figs. 2C, 4B: sr) relatively large, softly and amply curved (convexity left, wide), insertion of hermaphroditic duct more anterior, at middle level of seminal receptacle duct. Fertilization complex simple, located at narrow and elongated base of seminal receptacle as duct of seminal receptacle; slightly shorter than length of receptacle. Fertilization complex totally immersed in albumen gland, inserting in posterior end of spermoviduct, by side of albumen gland duct, relatively wide, twice wider than receptacle’s duct. Albumen gland (ag) solid, white, elliptical, more-or-less same size as gonad (~1/3 whorl). Albumen gland duct subterminal, connected to distal end of spermoviduct (Fig. 4B), in lateral, small albumen chamber (Fig. 4D: ac); narrowly connected to distal end of spermoviduct (Fig. 4B: eo). Spermoviduct (Figs. 2C, 24A-B: eo) of ~1.5 whorl in length, slightly narrower than albumen gland, ~10 times longer than wide; prostate wide (pt); glandular walls in uterus only along lateral side (Figs. 4B, E: ut), remaining ~2/3 of uterine inner surface relatively thin-glandular. Presence of accessory genital gland (Figs. 2B, E: as) lying along spermoviduct, flanking masculine furrows (sp), bulging along

prostate (pt) side. Pair of masculine furrows lying along accessory genital gland (Figs. 4B, E: sp). Vas deferens originating in simple aperture slightly posterior to end of uterine level (Fig. 4A: vd). Vas deferens not coiled (Figs. 2C, 4C: vd). Genital muscles not detectable. Penis slightly shorter than half of spermoviduct length (Fig. 2C: pe); penis muscle inserted only between penis and epiphallus (Fig. 2C: pm), with small branch connected to vas deferens in its region preceding its insertion (Fig. 4C: pm). Epiphallus (eh) ~1/6 penis' length; vas deferens also inserted in middle level of outer epiphallus wall (Fig. 4C: vd). Epiphallus inner surface with high layer of papillae (Fig. 4D: eh). Internal penial arrangement of folds similar, but with fewer and wider folds, pair of more developed (main) folds (Fig. 4D: pf) not so developed, being wider and lower; papillae slightly smaller than those inside epiphallus also lying along entire distal 2/3 of penis (Figs. 2F, 4D).

Central nervous system. (Fig. 2A: nr) More detailed in following studied species, being noteworthy cerebral commissure wide; cerebral node (cd) not so developed; all connectives slightly shorter and all ganglia slightly larger.

Distribution. Apparently restricted to Rio de Janeiro region. Remaining records to other states possibly are misidentifications (personal observation, upcoming study).

Habitat. Atlantic Rainforest.

Measurements. (in mm) MZSP 152925#2 (Figs. 1A-C): 30.2 by 13.3.

Material examined. BRAZIL. **Rio de Janeiro**; São Fidélis, Serra da Sapateira, 21°39'S 41°46'W, MZSP 152925, 9 specimens (2 with only shells) (Femorale leg., 2010).

Taxonomic remarks. See Discussion item.

Anthinus synchondrus new species (Figs. 5–8)

ZooBank: urn:lsid:zoobank.org:act:64ABD2D2-71C4-49D7-AA69-4321AA8595EB

Types. Holotype MZSP 152074. Paratypes: MZSP 152252, 2 shells, MZSP 152075, 1 shell from type locality. BRAZIL. **Minas Gerais**; Unaí (W.V. Matos col, iv/2020), Gueda, 16°16'04"S 46°39'43"W, 647-650 m altitude, MZSP 152236, 1 shell.

Type locality. BRAZIL. **Minas Gerais**; Unaí, Pedra da Fartura, 16°31'37"S 46°49'18"W, 628-750 m altitude [Weslley Vailant de Matos col, iv.2020].

Etymology. The specific epithet is derived from Greek words *syn*, a prefix meaning "together", and *chondros*, meaning "gristle, cartilage". This is an allusion to the total fusion of the odontophore cartilages (Fig. 6D).

Diagnosis. Shell about 40 mm; color mosaic of brown-beige spots; spire angle ~35°, protoconch of 3.2 whorls; peristome ~48% of shell length and ~50% of width; width ~54 of length. Mantle edge with projected fold. Reno-pericardial area large. Pre-rectal pallial muscle present. Odontophore with cartilages totally fused; pair m4 ~50% originated from cartilage, remaining on m4. Jaw plate narrow. Radula with elongated, spatula-like mesocone, rachidial slightly different from neighboring teeth. Seminal receptacle tightly curved, hermaphrodite duct inserting in its base perpendicularly. Accessory genital gland in spermoviduct large; with two similar sperm-grooves. Penis ~60% of spermoviduct; penis muscle with pair of small branches in epiphallus; epiphallus papillae low, uniform, restricted to it; pair of larger folds composed of transverse subfolds.

Distinctive description

Shell. (Figs. 5A–L) Adult shell around 40 mm, conical-oval; apex blunt; wider at middle of last whorl; width ~54% of shell length. Walls weakly thick. Dorso-ventrally weakly flattened (Fig. 5E). Color light beige and brown squared spots, both predominating (Fig. 5C) or slightly brown predominating (Figs. 5H, J); brown spots slightly randomly distributed along 4-5 spiral, wide, rather isometric bands in last whorl, 2-3 spiral bands in penultimate whorl; usual concentration of brown pigment preceding peristome. Spire angle ~35°. Protoconch of 3.2 whorls (Figs. 5B, J, K), ~23% of length, width of ~7 mm, ~twice taller than wide; first whorl smooth, gradually axial and spiral narrow cords appearing (Figs. 5K, L), initially both predominating, gradually axial cords slightly stronger, from suture to suture, interspaced slightly broader than each cord, ~10 per mm; transition to teleoconch clear, slightly prosocline (Figs. 5B, H, K). Teleoconch sculpture similar to that of protoconch last whorl, as axial, uniform, complete cords and growth lines, gradually fading in last whorl (Figs. 5C, H, J); of ~2.3 whorls. Whorls profile weakly convex (Figs. 5G-J) (Figs. 5A-C, F, I, J). Suture well-marked, weakly channeled (Figs. 5J, K, L). Aperture slightly prosocline (~5° from longitudinal general axis) (Fig. 5B), oval; ~48% of shell length, ~50% of shell width. Peristome well-reflected, thick, color variating from pale beige (Figs. 5A, G) to white (Fig. 5I); outer lip arched, lacking middle tooth; inner lip with inferior half widely concave, superior half as narrow, weak callus with ~1/3 of peristome length (Figs. 5A, I), usually concave, rarely convex (Fig. 5G). Inferior half of inner lip intensely covering umbilicus (Figs. 5A, D, G, I). Umbilicus narrow (Fig. 5B). Body whorl ~55% of shell length; usually uniform with spire (Figs. 5A, G, I).

Head-foot. Similar features as *A. multicolor*, including columellar muscle having only two layers; i.e., secondary head muscles (ou, tm) originating direct from pair of secondary muscles (tm).

Mantle organs. (Fig. 6A) most characters similar to *A. multicolor*. Distinctions and remarks following. Mantle edge dorsal fold well developed (mf), with elongated edge pointed to left. Anus directly opening externally (ventrally), preceded by right longitudinal folds (Fig. 5N: af). Urinary gutter (ur) slightly broader, with slightly taller perpendicular folds.

Visceral mass. Similar features as preceding species.

Circulatory and excretory systems. (Fig. 6A) Most features similar to *A. multicolor*; remarks following. Pericardium (pc) lightly more elongated, ~3-times as long as wide. Kidney (ki) tissue clearly U-shaped (convexity left). Nephropore (ne) also protected by curved anterior projection of kidney. Urinary gutter (ur) slightly broader, mainly along its middle portion. Narrow urinary gutter surrounding left edge of anus (an) also present.

Digestive system. (Figs. 6C–8A) General features similar to those of *A. multicolor*, distinctions and interesting data following. Jaw slender, with uniform width along its length (Figs. 5M, 8A: jw), weak central beak; transverse ribs very weak, almost imperceptible; commarginal sculpture wanting. Buccal mass and odontophore (Fig. 6D) lacking m1a, m1v; **m3**, only detected in latero-dorsal region (Figs. 6C, 8A: m3); **m2**, slightly thinner, insertion narrow on m4 base (Fig. 6D); **m4**, very thick, relatively short; **m5**, thin and elongated, origin about half on m4 and half on posterior region of cartilages; **m7**, small narrow pair, origin in posterior end of cartilages, running inside radular sac, splaying along its inner surface. Pair of odontophore cartilages entirely fused with each other along their inner-ventral edge (Fig. 6D: oc). **Radula** (Fig. 7) with same general features of preceding species; with rachidian teeth, and ~40 pairs of lateral/marginal teeth; no clear distinction between lateral and marginal teeth (Fig. 7C); differing in having longer, rather rectangular mesocone, ~3 times longer than wide, covering ~1/3 of posterior tooth (Figs. 7B-C), mesocone apex rounded; long mesocone reinforced by longitudinal fold in inner concavity, continuous with base; base with similar characters as preceding species. **Rachidian** tooth (Figs 7A-C: arrow) slightly narrower than lateral teeth, with mesocone with ~half size of neighboring mesocones (Fig. 7C). **Lateral teeth** with weak medial inclination, rarely with bifid cutting edge

(Fig. 7A: right side). **Marginal teeth** starting with no clear boundary with lateral teeth; shaped similarly to lateral teeth, except for being weakly smaller and narrower, row slightly more inclined (Fig. 7A: left side) along ~1/3 of each side. Salivary glands apertures as long longitudinal slits (Fig. 8A: sa), located in posterior region of lateral edges of wide dorsal folds (df). Esophagus broader all along its length (Fig. 6C: es). Stomach (st) ~50% larger, walls with considerable muscular walls; stomach inner surface (Fig. 6B) with fold (sf) running between anterior duct to digestive gland (dr) and intestine (in) more developed. Esophageal-anterior duct to digestive gland also bifid (Fig. 6C), left duct (dl) almost as wide as right duct (dr), much longer. Anus turned externally (Fig. 6A: an); inner surface also with 8–10 simple, longitudinal, subterminal, tall folds (Fig. 5N: af).

Reproductive system. (Figs. 8B-F) General characters similar to those described for *A. multicolor*, distinctions and remarks following. Hermaphroditic duct (Fig. 8B: hd) with broader coiled portions along its middle and distal thirds, with fewer but wider coils; insertion lacking curve (Fig. 8C). Seminal receptacle (Figs. 8B-C: sr) large, strongly and tightly curved (convexity left), forming distal blunt beak disposed pointing to hermaphroditic duct insertion. Spermoviduct (Figs. 8B-C: eo) with glandular walls in uterus only in ventral side (Fig. 8F: ut), remaining walls relatively thin-glandular. Presence of accessory genital gland (Figs. 8D, F: as) lying along spermoviduct, flanking masculine furrows (sp), bulging along prostate (pt) side. Pair of masculine furrows narrow, one lying along accessory genital gland, and other along its medial edge, ~half deep (Figs. 8D, F: sp). Vas deferens (vd) originating in simple junction of masculine furrows (Fig. 8D: vd), no vaginal fold. Vas deferens relatively broad, intensely coiled in its portion on free oviduct and vagina (Fig. 8B: vd). Penis slightly longer than half of spermoviduct length (Fig. 8B: pe); penis muscle inserting terminally, short, broad (Figs. 8B, E: pm), with pair of narrow branches connected to epiphallus. Epiphallus (eh) ~1/5 penis' length; vas deferens inserted subterminally on middle level of epiphallus wall (Fig. 8E: vd). Epiphallus inner surface as thick glandular, uniform layer of papillae. Internal penial surface with single chamber (Fig. 8E); arrangement of inner folds as in Fig. 8E, with 8-10 longitudinal wide folds, almost no interspace, in basal half; these folds gradually coalescent in middle penial region, additionally appearing narrower transverse folds in distal region; these folds forming two aligned bulged portions in penis side, producing pair of longitudinal cancelled wide folds (Fig. 8E: pf), separated by narrow furrow continuous with epiphallus aperture.

Central nervous system. Nerve ring located across buccal mass (Fig. 6C: nr). Pair of cerebral ganglia (ce) almost fused; cerebral commissure slightly narrower than ganglia; each ganglion about as wide as adjacent esophageal section; several wide nerves originating in cerebral antero-lateral region. Cerebral node or gland (cd) located in postero-medial quadrant, with ~1/8 each ganglion's size. On each side, parallel, rather narrow connectives (cn) running between cerebral ganglion and ventrally located fused pedal and pleural ganglia (pp), accompanied by blood vessels (bv) issuing from anteriorly-directed aorta. At least six pairs of nerves originating from anterior side of pedal-visceral ganglion complex. Pair of statocysts not seen.

Distribution. So far known in region of Unaí, Minas Gerais, Brazil.

Habitat. Cerrado biome, semi-dry forest, 628-750 m altitude.

Measurements. (length and width in mm) Holotype MZSP 152074 (Figs. 5A-F): 36.9 by 19.7; paratype MZSP 152252 (Figs. 5I-J): 36.4 by 19.6.

Material examined. Types (reported above).

Taxonomic remarks. See Discussion item.

***Anthinus vailanti* new species** (Figs. 9-12)

ZooBank: urn:lsid:zoobank.org:act:5CF7693D-4C22-4E66-9FCA-B0B821981E0B

Types. Holotype MZSP 154382. Paratypes: MZSP 152118, 3 spm, MZSP 152244, 2 shells, MZSP 152119, all from type locality.

Type locality. BRAZIL. **Minas Gerais**; North of Brasilândia de Minas, 16°59'29"S 46°00'29"W, 630-635 m altitude [Weslley Vailant de Matos col, iv.2020].

Etymology. The specific epithet is in honor of Weslley Vailant de Mattos, from Alcobaça, Bahia, the field collector responsible for most of the new discoveries.

Diagnosis. Shell about 30 mm; color mostly monochromatic; spire angle ~50°, protoconch of 3 whorls; peristome ~55% of shell length and ~60% of width; width ~60 of length. Mantle edge with pointed fold. Reno-pericardial area medium-sized. Pre-rectal pallial muscle present. Odontophore with cartilages ~90% fused with each other; pair m4 ~80% originated from cartilage, remaining on m4. Jaw plate broad, with central fold. Radula with elongated, spatula-like mesocone, rachidial slightly different from neighboring teeth. Seminal receptacle widely curved, hermaphrodite duct inserting in its middle region, perpendicularly. Accessory genital gland in spermoviduct large; with single, coiled sperm-groove. Penis ~60% of spermoviduct; penis muscle with pair of small branches in epiphallus; epiphallus papillae of medium size, not-uniform, extending to penial chamber; pair of larger folds simple, fused with each other proximally.

Distinctive description

Shell. (Figs. 9A–J) Adult shell around 30 mm, conical-oval; apex blunt; wider at middle of last whorl; width ~60% of shell length. Walls thin. Dorso-ventrally weakly flattened (Fig. 9J). Color uniform reddish brown in ~half of specimens (Figs. 9A-G); to sparse squared pale brown spots on uniform cream base in other half of specimens (Figs. 9H-J), spots less dense than preceding species, but with similar remaining features, with areas denser (Fig. 9J) and areas almost lacking spots (Fig. 9I). Periostracum heavy, deciduous in some areas, mainly in apex (Figs. 9A-G). First spire whorls white in superior half, gradually becoming pale brown in inferior half (Figs. 9D-E). Spire angle ~50°. Protoconch of 3.0 whorls (Figs. 9B, E), ~20% of length, width of ~8.8 mm, slightly taller than wide; first whorl smooth, gradually axial and spiral narrow cords appearing (Figs. 9D-E), initially both predominating, gradually axial cords slightly stronger, from suture to suture, interspaced slightly broader than each cord, ~6 per mm, last protoconch whorl with spiral sculpture more spaced, producing rectangular spaced with axial lines; transition to teleoconch clear, slightly prosocline (Fig. 9E). Teleoconch sculpture similar to that of protoconch last whorl, as axial, uniform, complete cords and growth lines, gradually small punctuations appearing on axial lines before penultimate whorl, aligned in order to produce spiral punctuated lines, ~20 in penultimate whorl (Fig. 9E), lying next to peristome (Figs. 9C, F, J); of ~2.2 whorls. Whorls profile weakly convex (Figs. 9A-J). Suture well-marked, weakly channeled (Figs. 9I). Aperture slightly prosocline (~20° from longitudinal general axis) (Fig. 9B), elliptic; ~55% of shell length, ~60% of shell width. Peristome reflected, thick, color white (Figs. 9A, F, H); outer lip arched, lacking middle tooth; inner lip with inferior half weakly concave, superior half as narrow, weak callus with ~2/3 of peristome length (Figs. 9A, F, H), almost straight. Inferior half of inner lip covering umbilicus. Umbilicus relatively wide (Fig. 9J). Body whorl ~80% of shell length; usually uniform with spire (Figs. 9F-J) to slightly broader (Figs. 9A-C).

Head-foot. Similar features as *A. multicolor*, except for more developed pedal glands, bulging in haemocoel floor.

Mantle organs. Most characters similar to *A. multicolor*. Except for urinary gutter ~5% broader,

Visceral mass. Similar features as preceding species.

Circulatory and excretory systems. Most features similar to *A. multicolor*; except for renal anterior fold proportionally smaller, ~half size. Renal glandular lobe also U-shaped, but with ventral branch with double thickness of dorsal branch.

Digestive system. General features similar to those of *A. multicolor*. Remarks following. Jaw plate (Fig. 9K-L) much broader and thicker; cutting edge blunt, with median transverse reinforcement (Fig. 9L) in its ventral side. Buccal mass possessing m1v pair. Pair of salivary ducts slightly broader. Odontophore (Fig. 10A) differing in having **m5** pair only ~20% originating from cartilages, remaining originating on m4; **m7** pair originated in dorsal-inner surface of posterior region of cartilages; odontophore cartilages (oc) only ~90% fused, with small posterior remaining notch. **Radula** (Fig. 11) similar to that of *A. synchondrus*, with ~50 pairs of lateral/marginal teeth (Figs. 11A, D); distinctions following. **Rachidian** tooth (Figs. 11B, E: arrow) further smaller and narrower; mesocone slightly claviform. **Lateral teeth** not inclined, pointing posteriorly only; mesocone slightly more elongated, covering ~1/3 of neighboring posterior tooth. **Marginal teeth** inclined outside, located in more arched row (Figs. 11C, D: left side). Anus subterminal folds taller and stronger, 4-5 in number only; occupying ~10% of rectum length.

Reproductive system. (Figs. 10B-D, 12A–B) General characters similar to those described for *A. multicolor*, distinctions and remarks following. Hermaphroditic duct (Fig. 10C: hd) also broad; coiled portions occupying almost its entire length, except for short stretch at both ends; insertion lacking curve (Fig. 10B). Seminal receptacle (Figs. 10B–C: sr) relatively small, strongly and widely curved (convexity left), insertion of hermaphroditic duct in middle of concavity. Spermoviduct (Figs. 10B, C: eo) with narrower prostate (pt); glandular walls in uterus only along latero-ventral side (Figs. 10D, 12A: ut), remaining ~1/3 of uterine inner surface relatively thin-glandular. Accessory genital gland (Figs. 10B, D, 12A: as) also present and large. Only single detectable masculine furrow lying along accessory genital gland (Figs. 10D, 12A: sp). Vas deferens originating in simple aperture in end of uterine level (Fig. 12A: vd), also lacking vaginal fold. Vas deferens also coiled in its portion on feminine tubes, but less intensely (Fig. 10C: vd). Presence of pair of genital muscles (Fig. 10C: gm) in ventral side of penis base. Penis slightly longer than half of spermoviduct length (Fig. 10C: pe); penis muscle also with pair of narrow branches connected to epiphallus (Figs. 10C, 12B: pm). Epiphallus (eh) ~1/6 penis' length; vas deferens also inserted subterminally on middle level of epiphallus wall (Fig. 12B: vd). Epiphallus inner surface with high layer of papillae (Fig. 12B: eh). Internal penial arrangement of folds similar, but with pair of more developed (main) folds (Fig. 12B: pf) narrower and slightly taller; papillae similar to those inside epiphallus also lying at side of these main folds (Fig. 12B).

Central nervous system. With similar attributes as preceding species.

Distribution. So far known from type locality.

Habitat. Cerrado biome, semi-dry forest, 630-635 m altitude.

Measurements. (in mm) Holotype MZSP 154382 (Figs. 9A-E): 29.0 by 17.7; paratypes MZSP 152118 (Figs. 9F-G): 27.9 by 17.7; MZSP 152244 (Figs. 9H-J): 29.5 by 16.5.

Material examined. Types (reported above).

Taxonomic remarks. See Discussion item.

Anthinus morenus **new species** (Figs. 13–15)

ZooBank: urn:lsid:zoobank.org:act:A34D18E0-47B6-40C8-A3C8-7FF3367E3AE5

Types. Holotype MZSP 151891. Paratypes: MZSP 152047, 12 spm from type locality (3 with only shells), MZSP 152048, 2 shells. BRAZIL. **Minas Gerais**; Paracatu, Gruta Sapezal, 16°48'03"S 46°53'47"W, 700-710 m altitude, MZSP 152195, 3 shells (WV Matos col., iv.2020).

Type locality. BRAZIL. **Minas Gerais**; Paracatu, São José do Sapezal, 16°48'03"S 46°53'47"W, 700-710 m altitude [Weslley Vailant de Matos col, iv.2020].

Etymology. The specific epithet is in apposition, a Latinization based on the Portuguese word "moreno", meaning dark-brown colored, an allusion to the dark shell color.

Diagnosis. Shell about 35 mm; color usual mosaic of brown and beige spots; spire angle ~50°, protoconch of 3 whorls; peristome ~51% of shell length and ~51% of width; width ~60 of length. Mantle edge with simple, rounded fold. Reno-pericardial area small-sized. Pre-rectal pallial muscle present. Odontophore with cartilages ~50% fused with each other; pair m4 ~40% originated from cartilage, remaining on m4. Jaw plate narrow, lacking central beak. Radula with elongated, spatula-like mesocone, rachidial rather similar to neighboring teeth. Seminal receptacle tightly curved, hermaphrodite duct inserting in its base, perpendicularly. Accessory genital gland in spermoviduct large; with pair of thick-walled sperm-grooves. Penis ~50% of spermoviduct; penis muscle mostly inserted to epiphallus; epiphallus papillae tall, restricted to it; single, large inner fold.

Description

Shell. (Figs. 13A-H, J-K) Adult shell around 35 mm, conical-oval; apex blunt; greatest width on last whorl; width ~60% shell length. Walls relatively thin. Color beige, with three spiral, equidistant brown bands up to penultimate whorl, five in last whorl; mosaic of sparse, apparently random transverse (axial) spots, irregular in size, normally limited to two neighboring spiral bands, but sometimes extending further (Fig. 13C, K). Spire angle ~50°. Slightly flattened dorso-ventrally, dorso-ventral axis ~80% of latero-lateral axis (Fig. 13E). Protoconch of 3 whorls (Figs. 13B, F, G, K), ~20% of length, width of ~7 mm; first whorl smooth, gradually axial narrow cords appearing (Figs. 13G-H), from suture to suture, interspaced similar to each cord, ~10 per mm; transition to teleoconch clear, slightly prosocline (Figs. 13B, F, G, K). Teleoconch of ~2.5 whorls; sculpture similar to that of protoconch last whorl in its first whorl, as axial, uniform, complete cords and growth lines (Fig. 13F); after first teleoconch whorl axial cords gradually disappearing, being substituted by rather irregular axial undulations and minute spiral aligned punctuations (Figs. 13C, F, J), ~25 spiral lines in penultimate whorl, interval between lines equivalent to their width up to double of it; spiral punctuated lines relatively uniformly covering surface up to peristome (Figs. 13B, C, K). Whorls profile slightly convex. Suture well-marked, slightly channeled (Figs. 13B, F, J). Aperture slightly prosocline (~7° from longitudinal general axis) (Fig. 13B), elliptic; ~51% of shell length, ~51% of shell width. Peristome reflected, thick, pure white (Figs. 13A, B) to pale beige (Fig. 13J); outer lip arched, lacking tooth (Figs. 13A, J); inner lip uniformly arched, concave; callus narrow, in superior third (Figs. 13A, J). Inferior 2/3 of inner lip partially covering umbilicus. Umbilicus relatively narrow (Fig. 13D). Body whorl ~84 shell length; usually looking wider than if spire had uniform growth (Figs. 13C, K).

Head-foot. (Fig. 14A) of normal shape, with characters similar to *A. multicolor*. Differing in pair of secondary cephalic muscles thicker, broader, and separated from each other almost up to their posterior end. Pedal gland not protruding in haemocoel.

Mantle organs. (Fig. 13L) most features similar to *A. multicolor*, differences following. Pallial roof uniformly pigmented dark (pu). Except for pulmonary vein (cv), all remaining pulmonary vessel not protruded, only visible with light passing through roof. Secondary mantle edge fold

(mf) smaller, not pointed at left. Lung longer, almost 2 whorls. Reno-pericardial structures smaller, occupying ~10% of roof area. Urinary gutter smooth, lacking transverse folds.

Visceral mass. of similar attributes of preceding species.

Circulatory and excretory systems. (Fig. 13L) with same features as preceding species, except in being ~40% smaller.

Digestive system. General features similar to those of preceding species. Remarks following. Jaw plate (Fig. 13I) narrow, relatively thinner, including its cutting edge. Pair of salivary glands ~30% less developed. Odontophore (Fig. 14B) differing in having **m5** pair with narrower origin, ~40% originating from cartilages, remaining originating on m4; **m7** absent; odontophore cartilages (oc) only ~50% fused, with long posterior notch separating both. **Radula** (Fig. 15) mostly similar to *A. synchondrus,* with ~60 pairs of lateral/marginal teeth; distinctions and remarks following. Mesocone inner reinforcement clearly shown in Fig. 15C (central column, with teeth in profile), running along mesocone, becoming thicker and attaching to base. All teeth narrower and more elongated, ~4-5 times longer than wide (Figs. 15B, F); some rare column bifid (Fig. 15C: right side). **Rachidian** tooth of difficult visualization (Figs. 15A, B, E), ~1/3 narrower and shorter than lateral tooth, and slightly dislocated anteriorly in relation to its row (Fig. 15D: arrow). **Lateral teeth** with very elongated mesocone, widely curved posteriorly (Fig. 15D). **Marginal teeth** with no clear boundary with lateral teeth, as gradual diminishment in lateral regions (Figs. 15E-F, inferior region).

Reproductive system. (Figs. 14C-F) General characters similar to those described for preceding species, distinctions and remarks following. Hermaphroditic duct (Fig. 14D: hd) also broad; coiled portions occupying almost its entire length, except for short stretch at both ends (Fig. 14E). Seminal receptacle (Figs. 14D-E: sr) relatively small, strongly and tightly curved (convexity left), insertion of hermaphroditic duct in receptacle base, lacking curve (Fig. 14E). Spermoviduct (Figs. 14D, E: eo) with glandular walls in uterus along its entire outer side (Figs. 14E, F: ut). Accessory genital gland (Figs. 14E, F: as) also present and large. Pair of masculine furrows narrow, one lying along accessory genital gland, and other along its medial edge, ~half deep (Figs. 14E, F: sp). Vas deferens originating in simple aperture in end of uterine level, also lacking vaginal fold. Vas deferens also coiled in its portion on feminine tubes, but less intensely (Fig. 14D: vd). Presence of single genital muscle (Fig. 14D: gm) in ventral side of vaginal base. Penis ~half of spermoviduct length (Fig. 14D: pe); penis muscle mostly inserted in epiphallus (Figs. 14D: pm) with additional small portion connected to penis distal end. Epiphallus (eh) ~1/4 penis' length; vas deferens also inserted subterminally in middle level of epiphallus wall (Figs. 14C, D: vd). Epiphallus inner surface with high layer of papillae (Fig. 14C: eh). Internal penial arrangement of folds similar, but with single more developed (main) fold (Fig. 14C: pf) broad and slightly taller; penial wall substantially thick muscular, inner set of folds (Fig. 14C) softer and simpler.

Central nervous system. With equivalent attributes as preceding species; except having longer cerebral commissure

Distribution. Paracatu region, Minas Gerais.

Habitat. Cerrado biome, semi-dry forest, 700-710 m altitude.

Measurements. (length and width in mm) Holotype MZSP 152891 (Figs. 13A-H): 35.8 by 21.0; paratype MZSP 152047#1 (Figs. 13I-L): 34.8 by 20.8.

Material examined. Types (reported above).

Taxonomic remarks. See Discussion item.

***Anthinus savanicus* new species** (Figs. 16-19)

ZooBank: urn:lsid:zoobank.org:act:7816D208-22FE-4C19-BEE5-ED2DD68B3FAD.

Types. Holotype MZSP 154391. Paratypes MZSP 153897, 6 spm, MZSP 152171, 4 shells, all from type locality.

Type locality. BRAZIL. **Goiás**; north of Formosa, 15°21'50"S 47°27'08"W [Weslley Vailant de Matos col, ix.2012].

Etymology. The specific epithet is derived from the regional biome, called *Savana Acidentada* (rugged savanna).

Diagnosis. Shell about 32 mm; color usual mosaic of brown and beige spots; spire angle ~50°, protoconch of 2.8 whorls; peristome ~48% of shell length and ~51% of width; width ~60% of length. Mantle edge with projected fold. Reno-pericardial area large-sized. Pre-rectal pallial muscle present. Odontophore with cartilages ~85% fused with each other; pair m4 ~50% originated from cartilage, remaining on m4. Jaw plate broad, thick. Radula with elongated, spatula-like mesocone, rachidial rather similar to neighboring teeth. Seminal receptacle tightly curved, hermaphrodite duct inserting in its base, curved. Accessory genital gland in spermoviduct small; with pair of sperm-grooves, one being T-shaped. Penis length ~80% of spermoviduct; penis muscle mostly inserted in penis distal end, with thick branch to epiphallus; epiphallus papillae tall, restricted to it; large pair of penial inner folds at end of secondary folds.

Distinctive description

Shell. (Figs. 16A–F, I–K) Adult shell around 32 mm, conical-oval; apex blunt; wider at middle of last whorl; width ~60% of shell length. Walls slightly thick. Dorso-ventrally slightly flattened (Figs. 16D, E). Color light beige and brown squared spots, both predominating; brown spots randomly distributed along 4-5 spiral, wide, rather isometric bands in last whorl, 2-3 spiral bands in penultimate whorl; usual concentration of brown pigment preceding peristome (Figs. 16C, J). Spire angle ~50°. Protoconch of 2.8 whorls (Figs. 16B, D, F, J), ~19% of length, width of ~7.7 mm, slightly taller than wide; 5-6 narrow, spiral lines present since first whorl, equally spaced, gradually more spiral and axial narrow lines appearing (Fig. 16F), initially spiral lines predominating, gradually in last whorl both predominating, gradually axial cords slightly stronger, from suture to suture, interspaced by similar width as lines', ~7 per mm; transition to teleoconch clear, slightly prosocline (Figs. 16B, F, J). Teleoconch sculpture similar to that of protoconch's last whorl, as axial, uniform, complete cords and growth lines; cords containing punctuations, aligned with their neighboring cords producing spiral punctuated cords (Figs. 16A, B, I, J); ~18 lines in penultimate whorl. Suture well-marked, weakly channeled (Figs. 16J, K). Aperture slightly prosocline (~10° from longitudinal general axis) (Fig. 16B), elliptical; ~48% of shell length, ~51% of shell width. Peristome reflected, thick, color white (Figs. 16A, I, K); outer lip arched, lacking middle tooth; inner lip with inferior half shallowly concave, superior half as narrow, weak callus with ~2/5 of peristome length (Figs. 16A, I, K), usually convex. Inferior half of inner lip intensely covering umbilicus. Umbilicus relatively wide (Fig. 16E). Body whorl ~80% of shell length; uniform with spire (Figs. 16C, J, K).

Head-foot. (Fig. 16H) Similar features as preceding species.

Mantle organs. (Fig. 16H) most characters similar to preceding species. Distinctions and remarks following. Pallial cavity ~0.75 of whorl. Mantle edge dorsal fold (mf) with very elongated edge pointed to left. Urinary gutter (ur) slightly narrow, transverse folds low.

Visceral mass. Similar features as preceding species.

Circulatory and excretory systems. (Fig. 16H) Most features similar to preceding species; except in being proportionally larger, occupying ~40% of pallial cavity (pc, ki).

Digestive system. (Figs. 17A-B) General features similar to those of preceding species, distinctions and interesting data following. Jaw strong, well-arched, with central inner reinforcement (Fig. 16G), weak central beak; transverse ribs very weak, almost imperceptible; commarginal sculpture wanting. Buccal mass and odontophore (Fig. 17B) lacking m1a, m1v and m3d; **m3p**, well-developed, extending along base of esophageal origin; **m4**, very thick, relatively short; **m5**, thin and short, origin about half on m4 and half on posterior region of cartilages; **m7**, absent. Pair of odontophore cartilages ~85% fused, with small posterior medial notch separating both cartilages (Fig. 17B: oc). **Radula** (Fig. 18) virtually similar to preceding *A. morenus*, with ~50 pairs of lateral teeth; but with all teeth slightly shorter and wider (Figs. 18A, C, D), mesocone reinforcement (Fig. 18B) and bifid column (Fig. 18A: center) also detected. **Rachidian** tooth (Fig 18E: arrow) similar to lateral teeth, except in being slightly smaller. **Marginal teeth** also turned outside, but proportionally larger, more similar to lateral teeth (Fig. 18E: left side). Salivary ducts narrow, elongated (Fig. 17A: sd). Esophagus broader all along its length, inner folds longitudinal, rather irregular (Fig. 17A: es). Stomach (st) large, walls considerably muscular; stomach inner surface (Fig. 17A) lacking developed fold, only having small transverse folds (sf) close to duct to digestive gland (dl). Esophageal-anterior left duct (dl) also broad and long.

Reproductive system. (Figs. 17C, D, 19A–C) General characters similar to those described for preceding species, distinctions and remarks following. Hermaphroditic duct (Fig. 17C: hd) with coiled portions only in distal half, with fewer and simpler coils; insertion with strong curve (Figs. 17C, 15A: hd). Seminal receptacle (Figs. 17C, 19A: sr) large, strongly and tightly curved (convexity left), forming distal blunt beak pointing to hermaphroditic duct insertion. Spermoviduct (Figs. 17C, 19A, B: eo) with glandular walls in uterus relatively low and splayed (Fig. 17D: ut). Accessory genital gland relatively smaller (Figs. 17D, 19B: as), only present in basal 2/3 of spermoviduct, distal third lacking this gland (Fig. 19A); masculine furrows composed of pair of separated folds flanking wide groove (Figs. 17D, 19B: sp). Vas deferens (vd) originating in simple junction of masculine furrows (Fig. 19B: vd), no vaginal fold. Vas deferens relatively broad, coiled only in some sparce portions (Fig. 17C: vd); becoming very narrow along penial region. Penis almost as long as spermoviduct length (Fig. 17C: pe); penis muscle inserting as pair of relatively same-sized wide branches attached both, to epiphallus and to distal penial end (Fig. 17C: pm). Epiphallus (eh) ~1/6 penis' length; vas deferens inserted subterminally on middle level of epiphallus wall. Epiphallus inner surface as thick glandular, uniform layer of papillae (Fog. 10C: eh. Internal penial surface with single chamber (Fig. 19C); arrangement of inner folds as in Fig. 19C, with 8-10 longitudinal wide, low folds, almost no interspace, along entire penis length; these folds distally as pair of longitudinal multilobed folds (Fig 19C: pf) restricted to single side, with wide, smooth groove separating them; this groove continuous to epiphallus aperture (Fig. 19C).

Central nervous system. with equivalent attributes as preceding species, except for cerebral pair of ganglia more closely located, with wider commissure; and for slightly more antero-posteriorly elongated cerebral and pedal pair of ganglia.

Distribution. So far known the type locality.

Habitat. Cerrado biome.

Measurements. (length and width in mm) Holotype MZSP 154391 (Figs. 16A–C): 32.5 by 20.3; Paratype MZSP 153897 (Figs. 16I–L) 31.5 by 18.7; Paratype MZSP 152171 (Fig. 16K): 35.3 by 20.1.

Material examined. Types (reported above).

Taxonomic remarks. See Discussion item.

Genus *Catracca* new genus

ZooBank. urn:lsid:zoobank.org:act:C9AEAD6E-EA30-4584-A884-AB967AE8CE6A

Diagnosis. shell of relatively thick walls; spire tall, with blunt tip; narrowly umbilicate. Protoconch of three whorls; first whorl smooth; narrow close-placed axial cords uniformly disposed in remaining whorls, from suture to suture. Peristome deflected, callus narrow. Color uniform, lacking spots. Pallial cavity lacking septum, weak vascular network. Kidney with anterior projection protecting nephrostome. Odontophore with pair m5 originated part from pair m4 and part from pair m2. Stomach with walls relatively muscular. Seminal receptacle balloon-like, non-curved. Spermoviduct lacking accessory genital gland. Epiphallus short, rounded, amply connected to penis. Penis internally comprising two chambers separated by strong and complex folded, transverse, ring-like, its distal edge comprising tall fold with well-developed sphincter.

Type species. ***Catracca uhlei*** new species.

List of included taxa. ***Catracca uhlei***.

Etymology. The genus' name is in apposition and is derived from the Portuguese word for ticket gate, *catraca*, as a make-up word; anyhow, its central region usually has a reinforced core sustaining the bars, this piece resembles the described shell. Gender: feminine.

Taxonomic discussion. see Discussion item.

Catracca uhlei **new species** (Figs. 20–24)

ZooBank: urn:lsid:zoobank.org:act:F8EEDDB8-F57F-40C2-831E-5F667C529BCF

Type specimens. Holotype MZSP 151900. Paratypes: MZSP 151901, 17 shells, MNRJ 23647, 1 shell, USNM 1661730, 1 shell, all from type locality. BRAZIL. **Minas Gerais**; Itacarambi (W.V. Matos col), entrance of Parque Nacional do Peruaçu (ii.2019), 15°10'30"S 44°13'24"W, 530 m altitude, MZSP 152161, 21 shells, MZSP 152222, 15 shells, MZSP 152242, 11 shells, Vargem Grande, 15°00'50"S 44°04'34"W, 466 m altitude, MZSP 151871, 3 specimens (7.ii.2020); Januária, border of Parque Nacional do Peruaçu, 14°57'55"S 44°04'21"W, 485 m altitude, MZSP 151893, 1 specimen, MZSP 151892, 10 shells (i.2020).

Type locality. BRAZIL. **Minas Gerais**; West of Itacarambi, 15°00'38"S 44°07'10"W, 540-550 m altitude [Weslley Vailant de Matos col, ii.2019].

Etymology. The specific epithet is in honor of Mauricio Sergio Uhle, São Paulo, a shell collector and sponsor of expeditions.

Description

Shell. (Figs 20A–I) Adult shell around 45 mm, conical-oval; apex blunt; wider on last whorl; width ~1/2 shell length. Walls relatively thick. Color uniform light beige or yellowish pale beige; some few specimens with reddish-brown tone in inferior third of spire whorls (Fig. 20F). Spire angle ~40°. Protoconch of 3 whorls (Figs. 20B, E), ~23% of length, width of ~9 mm; first whorl smooth, gradually axial narrow cords appearing (Figs. 20H, I), running from suture to suture,

interspaces similar to each cord's width, ~10 per mm; transition to teleoconch distinct, slightly prosocline (Figs. 20E, H). Teleoconch of ~2 whorls, profile slightly convex; sculpture similar to that of protoconch last whorl, as axial, uniform, complete cords and growth lines, slightly more irregular on last whorl (Figs. 20A, C, D, G). Suture well-marked, slightly channeled (Figs. 20B, C, E, G). Aperture slightly prosocline (~10° from longitudinal general axis) (Fig. 20B), oval; ~45% of shell length, ~50% of shell width. Peristome continuous, reflected, thick, same color as remaining shell; outer lip arched, usually with small, blunt tooth medially (Figs. 20A, D, G) absent in younger adults (Fig. 20F); inner lip with inferior half almost straight, superior, parietal half appressed to body whorl, with thick callus, sometimes planar in last whorl (Figs. 20D, F) sometimes flanked by shallow outer furrow (Figs. 20A, F). Inferior half of inner lip partially covering umbilicus. Umbilicus relatively wide (Figs. 20A, D, G), ~10% of inferior area. Body whorl ~2/3 shell length; usually regularly increasing in size (Figs. 20A, D, F), more rarely wider (Fig. 20G).

Head-foot. (Fig. 21B) of normal shape. Color uniformly pale. Columellar muscle thick, 1.5 whorls in length. Inner arrangement of columellar annexed muscles relatively complex. Main columellar bundle (cm) occupying ventral floor of haemocoel, relatively flat, wide slightly broader than half of foot width. Pair of secondary cephalic muscles (sm) with middle and anterior regions approximately double thickness and half width those of columellar main bundle (left muscle slightly broader than right one); originating gradually along dorsal surface of main columellar bundle, running anteriorly on main bundle, inserting in ventral region of perioral region. Pair of tentacular/ommatophore muscles originating between posterior and middle thirds of main columellar muscle bundle, between both secondary cephalic muscles; gradually thickening and bifurcating towards anterior; strong bifurcation halfway between their detected origin and anterior end, dorso-lateral branches becoming short and strong ommatophore muscles (ou), inserting near tip of ommatophore (om); ventro-medial branches running towards anterior, dorsally to secondary cephalic muscles, halfway newly bifurcating, lateral branch becoming short tentacular muscles (tm), inserting in tip of tentacles (te); medial branch running further towards anterior, inserting dorsally to insertion of secondary cephalic muscles. Pedal gland large, curved, protruding in ventral region of buccal area (Fig. 21C: pg).

Mantle organs. (Fig. 21A) Mantle border thick, lacking pigments. Pneumostome (pn) protected by simple right ventral flap (pv), width ~1/5 of aperture length. Dorsal fold well developed (mf), occupying ~1/3 of dorsal mantle edge length; left end pointed, projected beyond its base. Pneumostome (pn) ~1/10 of shell aperture length, bearing exclusively air entrance and urinary gutter. Anus (an) separate aperture located at right, adjacent to pneumostome. Lung of 1.5 whorls in length, wide and elongated; right side ~1.5 times longer than left side. Pulmonary vessels conspicuous, but low; 6-7 stronger vessels perpendicularly disposed at right; 6-7 main vessels at left radially disposed; all pulmonary vessels visible, but low, i.e., weakly protruded. Pulmonary vein (cv) running longitudinally across pallial cavity roof medially or towards right, anterior end trifurcated. Reno-pericardial area beige, slightly triangular, located posteriorly within pallial cavity, its posterior abutting wall of visceral cavity, occupying ~30% of cavity length and ~40% of its width (details below). Rectum (rt) wide. Urinary gutter (ur) narrow, running along rectum; anterior urinary gutter surrounding left anal aperture.

Visceral mass. (Fig. 21C) ~3 whorls in length. Both digestive gland lobes brown in color; anterior lobe (da) flattened, occupying ~1/5 of visceral volume, located just posteriorly to pallial cavity, continuous to kidney. Posterior lobe (dg) larger, extending 2 spiral whorls, occupying ~60% of visceral volume. Stomach ~1/15 of visceral volume, located between both digestive gland lobes, about 3/4 whorl posterior to pallial cavity (st). Digestive tubes (described below) surrounding anterior lobe of digestive gland. Gonad multi-lobed, cream color, encased between posterior lobe of digestive gland and columella, occupying ~1/3 whorl, ~1/10 of visceral volume.

Circulatory and excretory systems. (Fig. 21A) Pericardium (pc) ~twice as long as wide, located obliquely between middle and left thirds of posterior end of pallial roof, appressed against right lateral side of kidney; occupying ~5% of lung area. Auricle (au) located anteriorly, as continuation from pulmonary vein (cv); ventricle (ve) located posteriorly, larger. Kidney (ki) simple, dorso-ventrally flattened; size reported above; somewhat triangular, width ~2/3 of length; internally organized as two distinct regions – a longitudinal hollow cavity on left; filled by sponge-like renal tissue on right ~2/3 from posterior to anterior ends, being gradually broader towards anterior. Nephropore (ne) small, longitudinal slit at anterior-left corner of kidney, directed towards right; protected by curved anterior projection of kidney (kf). Urinary gutter (ur) composed of small folds perpendicular to rectum axis, flanking almost entire rectum left edge, starting at short distance from pulmonary postero-right end, ending in pneumostome as narrow urinary groove flanking left edge of anus (an).

Digestive system. (Figs. 21C–23B) Oral tube wide, muscular (Figs. 21E: mj). Radula features (Fig. 22) below. Jaw simple, thick, dark brown, exposed portion horseshoe-shaped, thicker at middle (Fig. 20J); surface having only coaxial low undulations and growth lines; located ai anterior end of buccal dorsal chamber (Fig. 23B: jw). Buccal mass spherical, occupying~1/5 of haemocoel volume (Fig. 21C: bm). Dorsal surface of oral cavity with well-developed pair of dorsal folds (Fig. 23B: df), width of each ~1/3 of dorsal wall width; separated from each other by dorsal chamber (dc) as wide as folds, but broader anteriorly. Odontophore with ~60% of buccal mass volume (Figs 21E, 23B: od). Odontophore muscles (Figs 21E–23B): **mj**, jaw and peribuccal muscles originating in outer-ventral surface of odontophore cartilages (Fig. 23A: mj), running towards dorsal, splaying in dorsal wall of oral tube (Fig. 21E: mj); **m1**, jugal muscles covering entirely haemocoelic structures, more concentrated close to mouth; **m1a**, small pair of lateral muscles, origin in latero-ventral region of haemocoel, running dorsally short distance, inserting in lateral surface of odontophore; **m1l**, small pair of lateral protractor muscles, origin in lateral region of buccal area, running towards posterior along 2/3 buccal mass length, inserting in lateral region of odontophore, anterior to m1a; **m1v**, small pair of ventral protractors jugal muscles, originating in ventral surface of haemocoel close to mouth, running towards posterior, inserting in ventral-posterior region of odontophore close to m2 insertion (Fig. 21E: m1v); **m2**, strong pair of retractor muscles of buccal mass, or radular muscles, originating as single bundle in columellar muscle posterior end (Fig. 21B: m2), running anteriorly close to median line along ~60% of haemocoel length, inserting as two different bundles in ventro-posterior edge of odontophore, surrounding at some distance radular nucleus (Fig. 23A: m2); **m3d**, thin layer of longitudinal fibers immersed in dorso-posterior wall of odontophore, preceding esophageal origin (Fig. 21E); **m3p**, thin layer of dorso-ventral fibers covering posterior odontophore wall, ventral to esophagus (Fig. 21E); **m4**, main pair of dorsal tensor muscles of radula, thick, originating in postero-medial region of odontophore cartilages (Fig. 23A: m4), surrounding outside and medially cartilages, inserting in subradular membrane in its region correspondent to buccal cavity; **m5**, pair of thick auxiliary dorsal tensor muscles of radula, originating on posterior-medial surface of m4 and short portion in postero-ventral region of odontophore cartilages (Fig. 23A: m5), , running towards median line covering m4, inserting in subradular membrane by side of m4 insertion; **m6**, horizontal muscle absent (Fig. 23A); **m7**, absent; **m10**, pair of narrow ventral odontophore protractor muscles, originating in ventro-anterior region of haemocoel, just ventral to mouth, running towards posterior along buccal mass length (Fig. 21E: m10), inserting in latero-posterior surface of odontophore close to m2 insertions; **m11**, pair of narrow ventral tensor muscles of radula absent. Odontophore non-muscular structures (Figs. 23A-B): **oc**, pair of odontophore cartilages flattened, rather elliptical, anterior region slightly projected anteriorly close to median line, ~1.2 times longer than wide, fused with each other along ~half in their anterior-medial edge (Fig. 23A: oc), posterior end roughly rounded; **sc**, subradular cartilage, with expanding region in

buccal cavity protecting subradular membrane. Radular sac short, not extending beyond odontophore.

Radula. (Fig. 22) Slightly longer than odontophore; with rachidian teeth, and ~45 pairs of lateral/marginal teeth; no clear distinction between lateral and marginal teeth (Fig. 22A), marginal teeth only slightly narrower than lateral teeth, and more inclined medially (Fig. 22C: right side); all teeth with base as long as mesocone, articulating neighboring rows (Figs. 22D, F); mesocone arched, curved inwards and posterior, ~twice longer than wide, apex bluntly pointed; no secondary cusps. **Rachidian** tooth (Figs. 22E-F: arrow) as large as lateral teeth; base ~twice longer than wide, slightly flattened, barely triangular, with arched posterior edge articulating with neighbor tooth and pair of distal reinforcements; mesocone ~half of base, symmetrical, located slightly dislocated posteriorly from adjacent lateral teeth row (Figs. 22E-F). **Lateral teeth** similar to rachidian (Figs. 22B, D), except in being slightly longer, asymmetrical, arched towards medial region; base trapezoid, with medial concavity articulating with neighbor medial tooth and single distal reinforcement (Fig. 22F). **Marginal teeth** starting with no clear boundary with lateral teeth (Figs. 22A, C) occupying ~1/3 of each side; shaped similarly to lateral teeth, except for being slightly narrower and more inclined medially (Fig. 22C).

Salivary glands covering esophagus in its region preceding its anterior quarter (Fig. 21C: sg), forming two elongated, white, thin masses. Each salivary duct differentiable in middle and anterior side of glands, with ~1/12 of esophageal width (Figs. 21C, E, 4B: sd). Salivary duct running in both sides of esophageal origin, penetrating buccal mass wall in region close to buccal ganglia (Figs. 21E, 23B: sd), running immersed in buccal dorsal wall along ~1/3 its length (Fig. 23B: sd). Salivary ducts opening as small slits in middle level of dorsal folds, on their lateral side (Fig. 23B: sa).

Esophagus ~1-whorl long, with thin, flaccid walls (Fig. 21C: es); anterior 1/3 clearly broader, crop-like; posterior 2/3 narrower; inner surface simple, with 5–10 simple longitudinal folds. Stomach (Fig. 21C: st) narrow, curved, almost not bulging; position and size described above (visceral mass); gastric walls relatively thick muscular (Fig. 21D: sw); inner surface mostly smooth (Fig. 21D), except for narrow longitudinal fold along smaller curvature (sf), connecting esophageal duct to digestive gland with intestinal origin; series of low, right folds flanking anterior side of longitudinal fold. Esophageal insertion on right side, intestinal origin on left side, both close to columella. Duct to anterior lobe of digestive gland at short distance from esophagus and intestine intersection (Figs. 21C, D: dr) wide, running shortly towards right, bifurcating; additional left duct (Fig. 21C: dl) originated in main duct base with ~1/3 of its width, running narrowly towards left and anterior along digestive gland lobe compressed by first intestinal loop (da), periodically possessing short ramifications along its length. Duct to posterior lobe of digestive gland located short distance from intestinal origin, posterior to above-described duct, directed towards opposite side (Figs. 21C, D: dd) slightly wider than anterior duct. Intestine (in) initially as wide as esophageal insertion, gradually broadening up to ~double that width along its wide sigmoid loop in anterior lobe of digestive gland (Fig. 21C: in). Rectum and anus position described above (pallial cavity) (Fig. 21A: rt, an). Anus sessile, as slit in right end of mantle edge directly turned outside; inner surface with 8–10 simple longitudinal, relatively tall folds.

Reproductive system. (Fig. 23C-24B) Gonad position described above (visceral mass), composed of 8–10 lobes with minute digitiform acini (Fig. 21C: go). Hermaphroditic duct (Fig. 23C: hd) narrow and weakly coiled in posterior half, abruptly becoming wider (up to 3-times wider), more intensely coiled in anterior half, coiling diminishing only close to insertion (Fig. 24D: hd). Seminal receptacle (Figs. 23C, D: sr) large, oval, balloon-like sac, ~twice longer than wide, ~three times wider than hermaphroditic duct. Fertilization complex simple, located at narrow and elongated base of seminal receptacle (Fig. 23D) as duct of seminal receptacle; slightly shorter than length of receptacle. Fertilization complex totally immersed in albumen gland,

inserting in posterior end of spermoviduct, by side of albumen gland duct, relatively wide, twice wider than receptacle's duct. Albumen gland (Fig. 23C, D: ag) solid, white, elliptical, more-or-less same size as gonad (~1/3 whorl). Albumen gland duct subterminal, connected to distal end of spermoviduct (Fig. 23D: ad), in lateral, large albumen chamber (Fig. 23D: ac); widely connected to distal end of spermoviduct (Fig. 23D: eo). Spermoviduct (eo) of ~1.5 whorl in length, slightly narrower than albumen gland, ~10 times longer than wide. Prostate gland occupying ~3/4 of spermoviduct surface and ~1/2 its volume (Figs. 23C, E: pt). Uterus occupying ~3/4 of spermoviduct space, external walls thick-glandular (Figs. 23C, E: ut), inner surface mostly covered by wide oblique folds (Fig. 23D: ut). Sperm groove entirely composed of two tall folds (Figs. 23D, E: sp), becoming vas deferens some distance posterior to genital pore (~1/15 of remaining spermoviduct length) (Figs. 23C, F: vd), with ~1/10 of anterior spermoviduct width, running outside of penis shield (Fig. 23C). Vagina ~1/20 spermoviduct length (Figs. 23C, F: vg); inner surface simple, with 8-10 longitudinal, low, wide folds (Fig. 23F: vg). Bursa copulatrix almost as long as spermoviduct length; bursa duct ~half of adjacent spermoviduct width in its origin, gradually narrowing towards posterior end (Fig. 23C: bd); bursa oval, ~1/3 of albumen gland size (Fig. 23C: bc), located encased between pericardium and adjacent intestinal loop. Penis ~3/4 of spermoviduct length, ~2/3 its anterior width (Fig. 23C: pe); penis muscle inserting terminally, short, broad (Figs. 23C, 24A: pm), with no branch on epiphallus. Epiphallus ~1/7 penis' length, tip rounded, located laterally (Fig. 24A: eh) inner surface with smooth, widely opening to penis posterior chamber (Fig. 24B: eh). Vas deferens inserted subterminally in penis tip, in intersection with epiphallus (Figs. 24A, B: vd). Internal penial surface with two clear chambers (Fig. 24B), strong transverse septum dividing penis in about similar-sized posterior (ps) and anterior (pa) chambers; transverse septum having strong sphincter in edge (sh), and proximal blunt projection bulging in a side of anterior chamber (pf); this transverse septum swelling middle portion of penis externally (Figs. 23C, 24A); posterior penis chamber with wide furrow running from opposite side of epiphallus aperture, up to region close to transverse septum (Fig. 24B: ps); anterior penis chamber with strong, wide longitudinal fold located at base of blunt projection (pf), tapering towards anterior; low, wide longitudinal folds covering entire surface of anterior penis chamber (pa). Genital pore with radial arrangement of muscles, with pair of more developed muscles (Fig. 23C: gm) running dorsally and posteriorly, connecting with middle region of columellar muscle.

Central nervous system. (Fig. 24C) Nerve ring located across buccal mass (Fig. 21C: nr). Pair of cerebral ganglia (ce) almost fused; cerebral commissure slightly narrower than ganglia; each ganglion about as wide as adjacent esophageal section; several wide nerves originating in cerebral antero-lateral region. Cerebral node or gland (cd) located in postero-medial quadrant, with ~1/8 each ganglion's size. On each side, parallel, rather narrow connectives (cn) running between cerebral ganglion and ventrally located fused pedal and pleural ganglia (pp), accompanied by blood vessels (bv) issuing from anteriorly-directed aorta. At least six pairs of nerves originating from anterior side of pedal-visceral ganglion complex. Pair of statocysts not seen.

Distribution. Minas Gerais region around Parque Nacional Cavernas do Peruaçu, so far from Itacarambi (north) to São João da Ponte (south).

Habitat. Limestone outcrops, Cerrado forest, 466-759 m altitude.

Measurements. (length and width in mm) holotype MZSP 151900 (Figs. 20A-C): 44.2 by 23.5 (Figs. 20A-C); paratypes MZSP 151901#1 (Fig. 20D): 42.8 by 20.7; #2: 43.0 by 24.4 (Fig. 20G); MZSP 151893: 47.1 by 25.3 (Fig. 20F).

Material examined. Types (reported above). BRAZIL. **Minas Gerais** (W.V. Matos col); Itacarambi, Vargem Grande, 15°00'50"S 44°04'34"W, 466 m altitude, MZSP 151869, 3 shells

(7.ii.2020); São João da Ponte, near Olímpio Campos, 15°50’44”S 44°00’03”W, 759 m altitude, MZSP 151811, 1 shell (i.2020).

Taxonomic remarks. See Discussion item.

Discussion

The taxon descriptions presented above do not include comparative remarks, which are reported in this item. However, the descriptions are presented in a comparative scenario with the preceding ones, evidencing the main differences amongst the taxa herein studied. Besides, there are the diagnoses, which bring the set of character necessary to identify each taxon. The present section is, thus, more focused on the most interesting features and those more relevant to taxonomy and present/future phylogenetic approaches to resolving relationships among South American strophocheilids.

Generic debate

As reported above, *Anthinus* appears to encompass the smallest strophocheilids [6], and those with a slightly “Bulimulus”-like, bulimoid shell, i.e., a fusiform shell with a relatively small aperture. These features easily differentiate the genus from the remaining cofamiliar genera that are bigger and with proportionally larger aperture. *Catracca*, introduced here, actually breaks this rule, as it is similar to *Anthinus* in these features, so much so that samples were initially identified as belong to that genus. Despite the *Catracca* shell lacking the colorful pattern of *Anthinus*, the discovery of *A. vailanti*, which also possesses a rather monochromatic shell, affects the generic separation only based on conchology. The anatomical features were fundamental to determine that these are distinct genera.

The set of characters reported here in diagnoses of both genera are sufficient to individualize them amongst the strophocheilids. However, it is recognized that the remaining genera do not have the same level of details known. Despite Leme [5] having redefined all strophocheilid (and megalobulimid) genera, including their anatomy, the level of details was not the same as those reported herein. Contrary to Leme’s [5] definition of *Anthinus*, some characteristics were not confirmed in the present study. In particular, the pair of colored bands of the antero-dorsal region of the animal were not found. Further, all examined *Anthinus* species possessed the genital accessory gland (e.g., Figs. 4E, 10D, 14F: as) that was considered absent, and being an exclusive feature of the megalobulimines. Other characteristics reported in Leme’s [5] diagnosis are accurate, such as the weakly muscular stomach lacking inner folds (e.g., Fig. 6B), the curvature of the seminal receptacle (e.g., Figs. 8C, 24B: sr) (which is also shared with other strophocheilines), and the shape of the epiphallus (e.g., Fig. 4C: eh). Other interesting characteristics that now can be added to the *Anthinus* diagnosis include the low profile of the pulmonary vessels, usual for species of smaller size, dark pigmented (e.g., Fig. 13L); the double origin of the odontophore muscle pair m5 (e.g., Fig. 6D); the posterior insertion of the anterior (esophageal) duct to the digestive gland, inserted practically into the stomach (e.g., Fig. 6B: dr), its bifurcation, with both components of similar caliber (e.g., Fig. 6C: dr, dl); the presence of the pallial, pre-rectal muscle (another supposed exclusive character of the megalobulimines); and the penis inner arrangement of folds forming a pair of stronger posterior folds, the furrow between which is continuous with the epiphallus aperture.

The new genus *Catracca* shares some characteristics with *Anthinus*, distinguishing both from other strophocheilids, such as the relatively small shell aperture; the small but distinct umbilicus (Figs. 20A, D); the configuration of the robust head musculature (Fig. 21B); the posterior position of the anterior duct of the digestive gland (Fig. 21C, despite the left branch,

dl, is much narrower); and of the nerve ring (Fig. 24C). However, *Catracca* has a set of exclusive characters that justify its introduction, reported in its diagnosis above, some commented below. In some aspects, the *Catracca* protoconch resembles those of *Megalobulimus* Miller, 1878 in being blunt, wide and with uniform axial sculpture (Figs. 20E, H, I). It differs, however, in being much more elongated and of smaller absolute size. The other strophocheilid genera have the protoconch with delicate reticulated sculpture and much more acuminate [4, 6]; *Catracca* actually has the bluntest perspective, with a proportionally widest protoconch amongst the strophocheilines, producing a somewhat mamillated shell. From the anatomical diagnostic characters of *Catracca*, which particularly distinguish it from *Anthinus*, the more interesting are the thick muscular stomach (Fig. 21D: sw), which in muscular development is inferior only to the gizzard-like stomach of *Megalobulimus* [5]; the odontophore pair m5 entirely originated from the pair m4; the sac-like, non-curved seminal receptacle (Fig. 23D: sr); the absence of accessory genital gland in the spermoviduct (Fig. 23E); and by the pair of vaginal folds (Fig. 23F: vf). However, the more outstanding distinctive feature of *Catracca* is its inner penial arrangement (Fig. 24B). The large transverse septum with well-developed sphincter in its edge (sh) is unique amongst the strophocheilids. A structure approaching this was only found only in *Mirinaba* Morretes, 1955, particularly in *M. planidens* (Michelin, 1831) ([5]: fig. 54). However, in the latter, the transverse septum is much lower, thicker, and lacks the sphincter. Besides, the *Mirinaba* copulatory organ has an elongated epiphallus, more similar to those of *Megalobulimus*. The epiphallus in *Catracca* is most similar to that of *Anthinus*, short and rounded (Fig. 23A: eh); it differs, however, in lacking inner papillae cover (e.g., Fig. 14C, 20C: eh). Moreover, in *Catracca* the vas deferens inserts in the border between the epiphallus and penis (Figs. 23A-B), while in *Anthinus* the vas deferens inserts in the middle level of the outer epiphallus wall (e.g., Fig. 8E: vd). The penis muscle insertion is restricted to the penis wall in *Catracca* (Fig. 23A), while some branches of it insert on the epiphallus in *Anthinus* (e.g., Figs. 8B, 17C: pm).

Interestingly, in *Anthinus*, the radula of the type species *A. multicolor* looks more like that of *Catracca uhlei* than those of remaining studied congeners. *A. multicolor* and *C. uhlei*, share short, blunt mesocones, particularly those of the rachidian teeth (Figs. 3C, 22F: arrow), while the mesocone of the remaining *Anthinus* species are much more elongated, flattened, spatula-like, with the rachidian more similar to lateral. The radulae of both genera differ from those of the megalobulimines in lacking broad mesocones, of almost the same size as the bases. The radulae of the other strophocheilid genera are not known.

The presence of supposedly megalobulimine characteristics in *Anthinus*, such as the pre-rectal pallial muscle (e.g., Fig. 17A: mp) and the accessory genital gland (e.g., Fig. 17D: as); as well as the megalobulimine supposed exclusive characters in *Catracca*, such as the protoconch (Fig. 20E) and the sac-like seminal receptacle (Fig. 23D: sr) are of difficult interpretation in respect to generic interrelationships. In the provisional phylogenetic treatment below, the accessory genital gland resulted in a convergence between megalobulimines and *Anthinus*. The same result (convergence) was obtained for the regular narrow axial cords in the protoconch shared between *Catracca* and some megalobulimines. The sac-like seminar receptable, on the other hand, appears to be plesiomorphic in the family.

Specific debate

A gallery of shells of all known *Anthinus* species are assembled in Figs. 25A–H, 26A–H, based on representatives (mostly holotypes – D–H), as well as of *Catracca uhlei* (Fig. 25I, 26I holotype). In that, the most outstanding feature is the monochromia of *A. vailanti* (Fig. 25F, 26F) and of *C. uhlei*, which already sets them apart from the remaining that possess characteristic beige-brown spots or blotches forming weakly-defined, spiral interrupted bands, i.e., pale spots or blotches on the beige-brown background. However, some specimens of *A.*

vailanti possess some evidence of such banding (Figs. 9H-I). After that, the present debate focuses on *Anthinus* species, considering that the diagnosis of each species already brings the set of characters exclusive of each species that supported their description. The present discussion focuses in the characters, every numerical parameter is based on a N from 10 to 20 specimens and is an integer value. Related to the shell elongation, *A. multicolor* is the slenderer (width/length W/L index = 46%) (Figs. 1A–C, 25C), with a group orbiting W/L ~50%, such as *A. synchondrus* (Fig. 25E – 54%), *A. albolabiatus* (Fig. 25A – 49%), *A. miersi* (Fig. 25B – 51%) and *A. turnix* (Fig. 25D – 55%); while another group has shells broader, encompassing species with W/L = 60%, such as *A. vailanti*, *A. morenus* (Fig. 25G) and *A. savanicus* (Fig. 25H). In respect to the spire angulation, *A. synchondrus* has the sharpest pointed spire, ~35°, followed by *A. multicolor* ~40°, and *A. albolabiatus* ~42°; *A. turnix* has the broader spire, with ~58°, followed by *A. miersi* ~55°; while the remaining species (*A. morenus*, *A. vailanti* and *A. savanicus*) have ~50°. The number of protoconch whorls is relatively constant amongst the species, with ~3 whorls; however, that of *A. synchondrus* looks slightly longer, with 3.2 whorls (Figs. 5K, L); while those of *A. multicolor* and *A. turnix* look shorter, with ~2.5 whorls, followed by *A. savanicus* with ~2.8 whorls (Fig. 16F). The protoconch absolute size, in mm, is also an important parameter, as usually is relatively constant within each species; the largest protoconch is that of *C. uhlei*, with 9 mm of diameter; it is followed by that of *A. vailanti*, with 8.8 mm; in the range of ~7 mm there are *A. savanicus* – 7.7 mm, *A. albolabiatus* – 7.5 mm, and *A. synchondrus* and *A. morenus* with 7.0 mm; the range of ~6 mm starts with *A. turnix* – 6.8 mm. *A. miersi* – 6.3 mm, ending with the smallest protoconch of *A. multicolor* – 6.1 mm. In relation to the aperture, the amplest is that of *A. turnix*, with aperture length occupying 60% if shell length, and 67% of shell width; it is followed by *A. miersi* – 58%-60% respectively; the smallest proportional peristome is that of *C. uhlei* – 45%-50%, followed by *A. synchondrus* – 48%-50% and *A. savanicus* – 48%-51%; the remaining species are in the range of peristome size about half of the shell's size, as follows: *A. morenus* – 51%-51%, *A. albolabiatus* – 52%-54%, *A. multicolor* – 52%-55%, and *A. vailanti* – 55%-60%. The shell's absolute size is also an interesting parameter, with *A. miersi* being the larger, with an average of 53 mm, followed by *A. turnix* – 50 mm; the smallest species is *A. vailanti*, with average size of 30 mm, followed by *A. savanicus* – 32 mm, and *A. morenus* – *A. multicolor*, both with 35 mm; *A. synchondrus* – 40 mm, and *A. albolabiatus* – 45 mm, are intermediary. The average size of *C. uhlei* is 45 mm.

The Table 1 summarizes the main conchological differences among the species studied herein.

Table 1. Summary of main conchological differences among the species studied in this paper (all measures are approximations of average measures of several specimens)

species	Shell size (mm)	Shell color	Spire angle	Protoconch whorls	Peristome x length	Width x length	Apex
Anthinus multicolor	35	mosaic	40°	2.5	52%	46%	narrow
Anthinus synchondrus	40	mosaic	35°	3.2	48%	54%	narrow
Anthinus vailanti	30	Monoch-romatic	50°	3	55%	60%	narrow
Anthinus morenus	35	mosaic	50°	3	51%	60%	narrow
Anthinus savanicus	32	mosaic	50°	2.8	48%	60%	narrow
Catracca uhlei	45	Monoch-romatic	40°	3	45°	50%	blunt

Regarded to *Anthinus multicolor*, a more detailed discussion on the loss of the types, geographic distribution, and shell variation, is found elsewhere [4]. In the original description [29], Rang reported that the examined material came from Brazil, particularly Saint-Paul province (the State of São Paulo), and from near Corcovado, which today is a place inside the city of Rio de Janeiro, State of Rio de Janeiro. Bequaert [4] reported the species as occurring in these two states (Rio de Janeiro and São Paulo), and figured a topotype ([4]: pl. 4, figs. 3, 7; MCZ 74441, from Corcovado), which is similar to the original illustration ([29]: pl 3, fig. 1). The specimen has irregular dark spots in its shell, lacking apparent spiral alignment as usually have the other species (Fig. 25). The shell illustrated by Simone [6] (Fig. 25C) was collected in Teresópolis, located close to Serra dos Órgãos, contiguous with the Corcovado rainforest. It also shows the same spot patterns of the previous illustrations [4, 29]. The presently studied sample were collected in São Fidélis, a place ~80km far from Corcovado, but also apparently in the same biome, as it is close to the Desengano State Park. The shells of the presently studied sample (Figs. 1A-C) also have the irregular, not spirally aligned spots, but they possess a wide spiral clear band along the middle level of the last whorl. This band is not present in all specimens of that lot, and, thus, is interpreted as variation. The non-alignment of the dark spots is, thus, an interesting species' distinctive character.

Related to anatomy, beyond the species described herein, there is some information only on *A. miersi* (radula, pallial roof and genitalia [7]) and the generic statement by Leme [5]. The anatomical discussion on the other species is, thus, precluded, and even on *A. miersi* the details are not equivalent. The mantle edge of the studied *Anthinus* has the usual pattern of most pulmonates, which includes a small left fold from the pneumostome (e.g., Fig. 21A: mf, which is pointed to the left); this fold has shown relatively constant in each species and of taxonomical value, in *A. synchondrus* and in *A. savanicus*, it has an elongated projection (Figs. 6A, 16H: mf); it is pointed in *A. vailanti*, and simply rounded in *A. morenus* and *A. multicolor* (Figs. 13L, 22A). The pallial cavity is interestingly darkly pigmented in all species (e.g., Fig. 134H: pu), in which the pulmonary vessels are difficult to discern. The different pattern of lung vascularization is evident in the drawings represented for each species. Furthermore, the relative size of the reno-pericardial area varies: *C. uhlei* and *A. vailanti* (Fig. 21A), considered of medium size (~1/4-1/5 of total cavity area); while *A. synchondrus* (Fig. 6A) and *A. savanicus* (Fig. 16H) can be considered large, occupying ~1/3 of lung area; and *A. morenus* (Fig. 13L), and *A. multicolor* (Fig. 2A), can be considered small (about 1/5 of lung area). The pallial pre-rectal muscle, as referred above, is supposedly a megalobulimine characteristic [5], but found in all here studied *Anthinus* examined here (Figs. 6C, 17A: mp) except for *A. multicolor* (Fig. 2A). In the digestive system, the jaw plate shows interesting comparative data; it is deeper and broad in *C. uhlei* (Fig. 20J), *A. vailanti* (Figs. 9K-L), *A. savanicus* (Fig. 16G), *A. multicolor* (Figs. 1G-H), and possibly in *A. miersi* [7]; however, it is shallow and wide in *A. synchondrus* (Fig. 5M), and *A. morenus* (Fig. 13I); moreover, a ventral additional medial fold reinforces the jaw plate exclusively in *A. vailanti* (Fig. 9L), and the jaw lacks a median anterior projection or beak only in that of *A. morenus* (Fig. 13I). Related to the odontophore intrinsic and extrinsic musculature, they have shown lightly similar; the most variable pairs are the m1, m3 and m7, which can be modified, present or absent according to the species (see descriptions), however, the variation found in pair m5 appears to be more interesting; it is 100% originated from m4 in *C. uhlei*, but it is ~50% originated from m4 and 50% from cartilages in *A. synchondrus, A. savanicus* and *A. multicolor*, it is ~60% originated from m4 in *A. morenus*, and ~20% in *A vailanti* (Fig. 10A). The degree of odontophore cartilages fusion along the ventral-inner edge is also of taxonomic interest, both cartilages are ~50% fused in *C. uhlei* (Fig. 23A) and *A. morenus* (Fig. 14B); ~90% fused in *A. vailanti* (Fig. 10A) and *A. multicolor*; ~85% fused in *A. savanicus* (Fig. 17B); while the cartilages are entirely fused in *A. synchondrus* (Fig. 6D). The radular teeth's mesocone is short, compared to those of the other genera of the family, in *C. uhlei* (Fig. 22), *A. multicolor*

(Fig. 23), and *A. miersi* [7]; however, it is elongated, spatula-like in *A. synchondrus* (Fig. 7), *A. vailanti* (Fig. 11), *A. morenus* (Fig. 15), and *A. savanicus* (Fig. 18). This mesocone elongation looks something exclusive of this subgroup of *Anthinus* amongst the known strophocheilids. An additional character is easily differentiating the rachidian tooth and its neighbors; the species with short mesocone, the differentiation between both is easy, while those with elongated mesocone, the rachidian is very similar to the neighboring teeth, being of difficult differentiation; from those, *A. synchondrus* and *A. vailanti* with certain effort it is possible to point out which is the rachidian column; however, this is very difficult in *A. morenus* and *A. savanicus*. Another interesting feature of the examined species is the posterior position of the anterior duct to the digestive gland, called the esophageal duct, which connects to the anterior lobe of the digestive gland. This esophageal duct originates practically from the stomach, while at least in *Megalobulimus*, it is far from the stomach; this feature is not known in the other strophocheilid genera. Beyond the odd position of the esophageal duct to digestive gland, there is the strong bifurcation of it, i.e., it bifurcates shortly after its origin. This characteristic is found both, in *Catracca* and in *Anthinus*, being only different in size; both branches have relatively similar caliber in *Anthinus* (e.g., Fig. 6C: dr, dl), while the left branch is narrower in *Catracca*; this does not occur at least in *Megalobulimus*, but this is unknown in other related genera.

The genital system usually is very informative in heterobranchs, but it has shown relatively conservative in strophocheilids, differing only in small details reported below. The presence of the supposedly exclusive megalobulimine accessory genital gland in spermoviduct (e.g., Figs. 8D, F: as) of the *Anthinus* is extraordinary. The seminal receptacle offers some distinction of the examined species, being balloon-like in *Catracca*, as well in megalobulimines [5], and strongly curved in *Anthinus*; however, the seminal receptacle is amply, widely curved in *A. vailanti* (Fig. 10B: sr) and *A. multicolor* (Fig. 4B); while it is tightly curved in *A. synchondrus* (Fig. 8C), *A. morenus* (Fig. 14E), and *A. savanicus* (Fig. 19A). The insertion of the hermaphrodite duct (hd) in the seminal receptacle is in its base in all studied species, except for *A. vailanti*, in which it is inserted in the middle of its concave edge; this insertion is strongly curved in *C. uhlei*, *A. savanicus* and *A. multicolor*, while it is straight, rather perpendicular in *A. synchondrus, A. vailanti* and *A. morenus*. The penis relative size is ~80% of the spermoviduct in *A. savanicus* (Fig. 17C: pe), ~60% in *A. synchondrus* (Fig. 8B) and *A. vailanti* (Fig. 10C), and ~50% in *C. uhlei* (Fig. 23C), *A. morenus* (Fig. 14D), *A. multicolor* (Fig. 2C), and *A. miersi* [7]. The accessory genital gland in the spermoviduct is relatively small in *A savanicus* (Fig. 4B: as) and *A. multicolor*, while it is large in the other three species. The number and form of the spermgrooves in the spermoviduct is also variable according to the species; they are double and similar-sized in *C. uhlei* (Fig. 23E: sp) and *A. multicolor* (Fig. 4E), they are also double, but asymmetrical in *A. synchondrus* (Fig. 8F), it is single and with a spiral fold in *A. vailanti* (Fig. 10D), they are double but of different thickness in *A. morenus* (Fig. 14F), they are double, but one of them is T-shaped in *A. savanicus* (Fig. 17D). In the vaginal-free oviduct cavity, *C. uhlei* has a double vaginal fold (Fig. 4F: vf), while *A. multicolor* has a hump (Fig. 4C: hp); the internal pattern of folds is different in each species, and are schematized in their respective drawings (Figs. 4A, 8D, 12A, 19B). The insertion of the penis muscle shows also interesting distinctions, it is only inserted in the penis wall in *C. uhlei* (Fig. 24A: pm), it has two small branches additionally connected to epiphallus in *A. synchondrus* (Fig. 8B), and *A. vailanti* (Fig. 10C), it has a larger component connected to epiphallus in *A. morenus* (Fig. 14D), it has a single and broad branch to epiphallus in *A. savanicus* (Fig. 17C), and it has a small branch connected to epiphallus and another to vas deferens in *A. multicolor* (Figs. 4C-D). As told above, all *Anthinus* have a papillae cover in epiphallus lumen, but the papillae are small and uniform in *A. synchondrus* (Fig. 8E), of medium size in *A. vailanti* (Fig. 12B), and tall in *A. morenus* (Fig. 14C), *A. savanicus* (Fig. 19C) and *A. multicolor* (Fig. 4D); additionally, the papillae extend

further along the penial chamber in *A. multicolor*, and along an adjacent area in *A. vailanti*; besides, the papillae of *A. multicolor* are not uniform. The internal pattern of penial folds is also distinctive, *A. morenus* is the only species in having a single and simple large posterior fold (Fig. 14C: pf); all remaining *Anthinus* have a pair of parallel folds in a side: *A. synchondrus* has these folds composed of a series of transverse threads (Fig. 8E); *A. vailanti* is the only in having this pair fused in the proximal region (Fig. 12B); *A. savanicus* has this pair formed by the end of the secondary folds of the remaining penial inner surface (Fig. 19C); while *A. multicolor* has these folds wide and covered by papillae (Fig. 4D). Interestingly, this pair of folds has its intermediary furrow continuous with the aperture of the epiphallus. All these internal structures are exteriorized through copulation, including the complex structure of *Catracca* (Fig. 24B). To understand how they work during copula is a fascinating issue for future investigation.

Anatomical and broader taxonomical debate

As referred above, details of the some remaining strophocheilid genera are still unknown, and what is known is mostly focused in megalobulimines. This precludes further inferences on the intrinsic and extrinsic interrelationships and phylogeny of the taxa. Leme (e.g., [5, 10, 22]) had that as a main professional goal, but it remains unfinished. The intention is to find a character or a set of characters supporting each taxon, which, based on lack of studies, so far is impossible. Megalobuliminae, firstly introduced as a family [5] really looks like a subdivision of Strophocheilidae, possessing the largest species (regarded as an anti-predatory strategy [30]), and supported at least by the pulmonary septum, buccal flange, pre-rectal septum in intestine, pre-rectal pallial muscle and accessory genital gland in the spermoviduct [5]. However, the presence of these two last characteristics in *Anthinus*, here presented, shows that these concepts must be reevaluated. Within strophocheilids, only *Megalobulimus* (so far the single genus of Megalobuliminae, with almost a hundred species), and *Anthinus* (this study) have samples with anatomical description sufficiently detailed for supporting more detailed comparative analyses like phylogeny. The other six genera [5, 6] are totally unknown or only have some few structures known.

The central nervous system (nerve ring) has shown relatively conservative in both genera studied here, all of them are similar (e.g., Fig. 24C), varying only in some small details and proportions, as reported above. At least in its main features, the nerve ring of the studied species is very similar to that of the megalobulimines [10] and even of the Bulimulidae (e.g., [31]).

A phenotypical base to understand the relationship of the Strophocheilidae with the other related families is also difficult for the same reason – lack of anatomical knowledge. At the time Strophocheilidae was only closely related to Dorcasiidae (African) and Acavidae (Asian) within Acavoidea [9], a group that apparently lays large eggs, produced by a thick walled, glandular spermoviduct, the phenotypic analysis was difficult. The scenario became more complicated with the present conception of these families as part of Rhytidoidea, with five additional families, based on molecular approaches [11, 12, 32]. The plan is to perform studies on other genera of all these families in a similar level of details of the present one, to produce a morphological database enough for a phylogenetic analysis. Only then a confrontation of the present taxonomy will be possible, and, possibly, to provide morphological bases for all subdivisions. The traditional Acavoidea above mentioned looks to be a morphologically well-supported taxon by above mentioned characters. However, the present Rhytidoidea still needs a better phenotypic support, as the fusion of Acavoidea with Rhytidoidea [11] is only based upon two papers with incongruent results, one of them did not have the Acavoidea as a focus [32], and another is an unpublished dissertation [33].

The Table 2 summarizes the main anatomic differences among the species studied herein.

Table 2. Summary of main anatomic differences among the species studied in this paper (all measures are approximations of average measures of several specimens)

species	Mantle edge folds	Reno-pericardial area	Pre-rectal pallial muscle	fusion of odontophore cartilages	Origin of m4 on cartilages	Jaw plate	Radula mesocone	Radular rachidian and neighboring	Seminal receptacle
Anthinus multicolor	rounded	small	absent	90%	50%	broad, thick	hook-like	similar	widely curved
Anthinus synchondrus	projected	large	present	100%	50%	narrow	Spatula-like	different	tightly curved
Anthinus vailanti	with pointed fold	medium	present	90%	80%	broad, central fold	Spatula-like	different	widely curved
Anthinus morenus	rounded	small	present	50%	40%	narrow	Spatula-like	similar	tightly curved
Anthinus savanicus	projected	large	present	85%	50%	broad	Spatula-like	similar	tightly curved
Catracca uhlei	projected	large	absent	50%	100%	Ticker at middle	hook-like	different	Baloon-like

species	Insertion of hermaphrodite duct	Accessory genital gland	Inner masculine folds	Free oviduct	Length penis x spermoviduct	Insertion of penis muscle	epiphallus papillae type	epiphallus papillae extension	penial inner folds
Anthinus multicolor	At base, curved	small	pair of similar-shaped	With distal hump	50%	Mostly in penis tip	tall, uneven	along entire penis chamber	wide and low, with papillae
Anthinus synchondrus	At base, perpendicular	large	pair of similar-shaped	usual	60%	Pair on epiphallus	Low, uniform	Only inside	Larger folds composed of transverse subfolds
Anthinus vailanti	At middle, perpendicular	large	Single, coiled	usual	60%	Pair on epiphallus	Medium, not uniform	Along penial chamber	Pair simple, part fused
Anthinus morenus	At base, perpendicular	large	Pair, thick	Usual	50%	Mostly on epiphallus	tall	Only inside	Single, large
Anthinus savanicus	At base, curved	small	Pair, one T-shaped	Usual	80%	Thick branch to epiphallus	tall	Only inside	Pair, large, secondary folds
Catracca uhlei	At base, curved	absent	Pair, thick	With genital fold	75%	Penis only	absent	—	Transverse septum

Phylogenetic analysis

Once more it is important to emphasize that the present phylogenetic analysis is not concerned with resolving the family Strophocheilidae, nor any other higher taxa. The main intention, using already published data, is to demonstrate that a new genus was necessary to be introduced, and the species does not fit in any of the known genera.

The computational processing of the Matrix (Appendix 2), which was based on the given list of 56 characters/126 states (Appendix 1) (see Material and Methods and [13]), resulted in a single cladogram (Fig. 28) with the following indices: length (L)= 283; consistency index (CI)= 68; retention index (RI)= 89.

In the cladogram (Fig. 28), the position of *Catracca uhlei* (after node 3) clearly shows that it does not belong to any known genera. It is recognized that some strophocheilid genera are not represented in the cladogram, but all of them (i.e., *Gonyostomus* Beck, 1837; *Speironepion* Bequaert, 1948; and the Andean *Austroborus* Parodiz, 1949 and *Chiliborus* Pilsbry, 1926) are beforehand reasonably different. The isolated position of *Catracca* in the base of Strophocheilidae (node 3), with two autapomorphies (character 7: *Megalobulimus oblongus*-like protoconch; character 52: transverse septum in the penis) is compatible with the generic separation.

Additionally, some few provisional conclusions are possible. The genus *Anthinus* resulted monophyletic (node 7), supported by six synapomorphies, such as the mosaic shell color (character 4); special arrangement of head muscles (ch. 11); the spatula-like radular mesocone (ch. 29), with rounded tip (ch. 30); the loss of the talon (ch. 42), and of the spermoviduct accessory gland (ch. 44). *Anthinus multicolor*, the type species, resulted as first branch, with two autapomorphies, and separated from the other congeners by three autapomorphies. The other four *Anthinus* species introduced herein resulted in successive branches, with *A. vailanti* at base, and *A. synchondrus*–*A. morenus* together as terminals.

Other interesting provisional results are the monophyly of the South American Strophocheilidae (node 2 – 14 synapomorphies), separated from the African Dorcasiidae (node 15 – 3 synapomorphies). The dichotomy of the family into Megalobuliminae (node 11 – 15 synapomorphies) and Strophocheilinae (node 3 – 5 synapomorphies) is also corroborated. Also noticeable is the division of the megalobulimines, in which what Leme [5] informally called as "*M. ovatus* group" (node 14 – 1 synapomorphy) and *"M. oblongus* group (node 12 – 2 synapomorphies). These possibly can be an indicative of genetic separation, an issue that is in analysis.

The present provisional cladogram achieved its goals, mainly concerned to *Catracca* allocation, as well as shows that *Anthinus* is monophyletic and has a perceptible internal arrangement that can arouse future subgeneric divisions. The increment of this cladogram with more taxa is an in-progress project.

Ecologic-distribution information

The discovery of several new taxa in central regions of Brazil has indicated how weak the knowledge level on the Brazilian malacofauna is. With human activities, possibly species have been lost even before they were at least known. Several Brazilian land snails have shown to be endemic from small areas (personal observations), which is an important factor for conservation efforts and politics. The taxa described in this paper are examples that lots of Brazilian species and even genera still need to be discovered or better-known. Knowing the fauna is the first step to raise protective rules to them and their environments. Thus, their descriptions are relatively urgent.

The type localities of all *Anthinus* and *Catracca* are shown in the map of Fig. 27. In that it is possible to see that there is a concentration in the northwest region of Minas Gerais (MG) (and adjacent region of Goiás – GO), and another in Rio de Janeiro (RJ). The single type locality far from those is that of *A. albolabiatus*, in east of Rio Grande do Sul (RS). Reports to *Anthinus* in literature and collections are not common, but samples and citations of *Anthinus* have been collected from south of Minas Gerais to North Argentina and Uruguay, sometimes

even identified at a specific level. As the areas in such the *Anthinus* samples were better studied – Rio de Janeiro and NW Minas Gerais (Fig. 27) show that several species with restricted distribution occur in relatively small areas, the question arises: are the *Anthinus* from the other regions belonging to undescribed species? As in the moment there is no possibility of checking this, no answer is possible.

Acknowledgements

A special thanks to José Coltro Jr and his team of collectors for the collect and donation of most studied samples of the present paper. To Lee Kremer for the thoughtful correction of the text. To Gary M. Barker and the other two anonymous referees, and the Editor for the comments, criticisms and improvements. To Lara Guimarães, MZSP, for the assistance in SEM examinations. The collects were performed under the IBAMA-Sisbio license 10560-2.

References

1. Albers JC. Die Heliceen, nach natürlicher Verwandtschaft. Verlag von Th.Chr.Fr. Enslin. Berlin, 262 pp; 1850.
2. Rang S. Descriptions des coquilles terrestres recueillies pedant un voyage à la côte occidentale d'Afrique, et au Brésil. Ann Sci Natur. 1831; 24: 1-63, pls. 1-3.
3. Martens Ev. Albers Die Heliceen, nach natürlicher Verwandtschaft (second edition). Verlag von Wilhelm Engelmann. Leipzig, 359 pp; 1860.
4. Bequaert JC. Monograph o the Strophocheilidae, a Neotropical family of terrestrial mollusks. Bull Mus Comp Zool. 1948, 100(1): 1–210, 32 pls.
5. Leme JLM. Anatomy and systematics of the Neotropical Strophocheiloidea (Gastropoda, Pulmonata) with the description of a new family. Arq Zool. 1973; 23(5): 295–337.
6. Simone LRL. Land and freshwater Molluscs of Brazil. São Paulo, EGB/ FAPESP; 2006.
7. Oliveira MP, Almeida EL, Vieira I, Oliveira MH. Comunicação n° 3: sobre o *Gonyostomus* multicolor miersii (Sowerby). Trab Setor Malac Univ Fed Juiz de Fora. 1968: 7-20.
8. Salvador RB, Colley E, Simone LRL. Terrestrial mollusks from the region of Corumbá and Maciço de Urucum, SW Brazil. J Conch. 2018; 43(1): 71–88.
9. Fontenelle JH, Miranda MS. Aspects of biology of *Megalobulimus paranaguensis* (Gastropoda, Acavoidea) in the coastal plain of the Brazilian southeast. Iheringia sér Zool. 2017; 107: e2017004; DOI: 10.1590/1678-4766e2017004.
10. Simone LRL, Leme JLM. Two new species of Megalobulimidae (Gastropoda, Strophocheiloidea) from north São Paulo, Brazil. Iheringia sér Zool. 1998; 85: 189-203.
11. Bouchet P, Rocroi JP, Hausdorf B, Kaim A, Kano Y, Nützel A, Parkhaev P, Schrödl M, Strong EE. Revised classification, nomenclator and typification of gastropod and monoplacophoran families. Malacologia. 2017; 61(1-2): 1–526.
12. MolluscaBase eds. MolluscaBase. Rhytidoidea Pilsbry, 1893. Website accessed at: http://www.molluscabase.org/aphia.php?p=taxdetails&id=890459 on 2021-02-10; 2021.
13. Simone LRL. Phylogeny of the Caenogastropoda (Mollusca), based on comparative morphology. Arq Zool. 2011; 42: 161–323.
14. Leme JLM, Castro RL, Indrusiak LF. Contribuição anatômica e histológica para o conhecimento de *Mirinaba antoninensis* (Morretes, 1952) (Gastropoda, Strophocheilidae). Pap Avul Zool. 1979; 32(14): 183–191.
15. Indrusiak LF, Leme JLM. Anatomia comparada de três espécies de *Mirinaba* Morretes, 1952 (Gastropoda, Strophocheilidae) do Estado do Paraná, Brasil. Acta Biol Paranaense 1985; 14: 163–180.

16. Leme JLM. *Megalobulimus lopesi* sp.n., uma nova espécie de Pulmonata terrestre da mata atlântica brasileira (Mollusca, Gastropoda, Megalobulimidae). Mem Inst Oswaldo Cruz 1989; 84(suppl. 4): 313–318.
17. Leme JLM, Indrusiak LF. *Megalobulimus parafragilior*, sp.n., uma nova espécie de Pulmonata terrestre da Serra do Mar (Gastropoda, Megalobulimidae). Pap Avul Zool 1990; 37(5): 97–105.
18. Leme JLM, Indrusiak F. Anatomia e considerações sobre *Megalobulimus proclivis* (Martens, 1888) (Gastropoda, Megalobulimidae). Iheringia (Zool) 1995; 78: 19–27.
19. Fontenelle JH, Simone LRL, Cavallari DC. *Megalobulimus dryades*, a new species from the Atlantic Forest in southeastern Brazil, and redescription of *Megalobulimus gummatus* (Gastropoda: Strophocheilidae). Pap Avul Zool 2021; 61: e20216144. Doi 10.11606/1807-0205/2021.61.44.
20. Silva FS, Simone LRL. Anatomy of two species of *Megalobulimus* (Strophocheilidae, Megalobuliminae) from Center-South of Bahia, Brazil. Pap Avul Zool (in press).
21. Simone LRL, Salvador RB. Taxonomical study on a sample of land snails from Nanuque (Minas Gerais, Brazil), with descriptions of three new species. Stutt Beit Naturk A 2016; N Ser 9: 9–30.
22. Leme JLM. Sobre a posição taxonômica de *Dorcasia* e *Trigonephrus* (Gastropoda, Pulmonata). Pap Avul Zool. 1980; 34(2): 11–19.
23. Simone LRL. Bosquejos de filogenia. Clube de Autores. Curitiba, 60 pp. https://www.google.com.br/books/edition/Bosquejos_De_Filogenia/b5TtDwAAQBAJ?hl=pt-BR&gbpv=0
24. Pilsbry HA. American bulimi and bulimuli – *Strophocheilus, Plekocheilus*, Auris, *Bulimulus*. Man Conch.1895-1896; (2)10: 1-213, 51 pls.
25. Morretes FL. Ensaio de catálogo dos moluscos do Brasil. Arq Mus Paran. 1949; 7: 1–216.
26. Abbott RT. Compendium of landshells. American Malacologists. Hong Kong, 240 pp; 1989
27. Salgado NC, Coelho ACS. Moluscos terrestres do Brasil (gastrópodes operculados ou não, exclusive Veronicellidae, Miladicae e Limacidae). IN Barrientos, Z & Monge-Nájera, J. [ed.] Malacología Latinamericana. Rev Biol Trop. 2003; 51(suppl.3): 149–189.
28. Oliveira MP, Rezende GJR, Castro GA. Catálogo dos moluscos da Universidade Federal de Juiz de Fora. Sinonímia de família, gênero e espécie. MEC, UFJF. Juiz de Fora, 520 pp, 1981.
29. Rang S. Description des coquilles terrestres recucillies pendand un voyage à la côte occidentale d'Afrique, et au Brésil. Ann Sci Nat. 1831: 24: 5–63.
30. Simone LRL. Physical defense strategies of South American land snails. Malacopedia. 2018; 1: 3–11. http://www.moluscos.org/trabalhos/Malacopedia/01-02%20Simone%202018%20Physical%20strategies%20of%20defense.pdf
31. Simone LRL, Belz CE, Gernet MV. A new species of *Drymaeus* endemic from Currais Archipelago, Paraná, Brazil (Pulmonata, Bulimulidae). Pap Avul Zool. 2020; 60: e20206057. DOI 10.11606/1807-0205/2020.60.57.
32. Herbert DG, Moussalli A, Griffiths OL. Rhytididae (Eupulmonata) in Madagascar: reality or conjecture? J Moll Stud. 2015; 81(2): 259–268
33. Teasdele LC. Phylogenomics of the pulmonate land snails. PhD Thesis, University of Melbourne. 199 pp. http://hdl.handle.net/11343/128240; 2017.

Legends

Fig 1. *Anthinus multicolor*, shell and some anatomical structures, sample MZSP 152925. (A-C) shell of specimen #2 (L 30.2 mm), frontal, right and dorsal views. (D) same, detail of apex, apical-slightly dorsal views. (E) same, protoconch and part of first teleoconch whorl in

profile. (F) penis, inner surface, detail of middle and distal thirds showing papillae. (G) jaw, ventral view. (H) same, anterior view. Scales= 1 mm.

Fig 2. *Anthinus multicolor*, anatomy. (A) pallial cavity, partially uncoiled visceral mass and digestive system, mostly ventral view, inner lip of pneumostome sectioned and deflected upwards. (B) head-foot, dorsal view, most of dorsal integument and inner organs removed, main concern to retractile/columelar musculature, some right-anterior structures sectioned. (C) genital system, mostly dorsal view. Scales = 3 mm.

Fig 3. *Anthinus multicolor*, radula in SEM, MZSP 152925. (A) wide view, scale= 200 µm; (B) detail of central region, scale= 50 µm; (C) same, higher magnification, scale= 30 µm. White arrows indicating rachidian column.

Fig 4. *Anthinus multicolor*, anatomy. (A) anterior half of genital tubes, opened longitudinally. (B) carrefour region of genitalia and posterior region of spermoviduct, albumen gland seen as transparent. (C) penis, ventral view. (D) same, opened longitudinally showing inner surface. (E) spermoviduct, transverse section in its middle region. Scales= 1 mm.

Fig 5. *Anthinus synchondrus*, shell of types and some anatomical photos. (**A-F)** holotype MZSP 152074 shell (L 36.9 mm). (A) frontal view. (B) right view. (C) dorsal view. (D) anterior-slightly ventral view. (E) apical view (W 19.7 mm). (F) frontal view after extraction of specimen by an artificial hole. (G-H) paratype MZSP 152236, (L 40.8 mm), frontal and dorsal views. (I-J) 2 paratypes MZSP 152252 shell, frontal views (L 36.4, 36.2 mm). (K) holotype, detail of protoconch and part of first teleoconch whorl, profile. (L) same, detail of apical region. (M) jaw of holotype in situ, ventral view. (N) detail of right-anterior corner of pallial roof of holotype, ventral view, distal portion of rectum sectioned longitudinally. Scales= 1 mm.

Fig 6. *Anthinus synchondrus*, anatomy. (A) pallial cavity roof, ventral view, pneumostome ventral lip sectioned and deflected upwards, portion of pallial floor also shown. (B) stomach opened longitudinally, ventral view. (C) digestive tubes mostly as in situ, ventral view, topology of some adjacent structures also shown. (D), odontophore, dorsal view, superficial layer of muscles and structures removed, left muscles deflected, radula deflected to left. Scales= 2 mm.

Fig 7. *Anthinus synchondrus*, radula in SEM, holotype MZSP 152074. (A) wider view, scale= 200 µm; (B) detail of central region, scale= 200 µm; (C) same, higher magnification, scale= 50 µm. White arrows indicating rachidian column.

Fig 8. *Anthinus synchondrus*, anatomy. (A) foregut opened longitudinally, ventral view, odontophore deflected to right. (B) genital system, mostly dorsal view. (C) same, detail of carrefour, ventral view, seen if albumen gland was transparent. (D) genital system, anterior end opened longitudinally, penis only partially shown. (E) penis and epiphallus opened longitudinally. (F) spermoviduct, transverse section in its middle level. Scales = 2 mm.

Fig 9. *Anthinus vailanti*, shells and jaw. (A-E) holotype MZSP 152891 (L 29.0 mm). (A) frontal view. (B) right view. (C) dorsal view. (D) detail of apex, right-slightly apical view. (E) protoconch and adjacent teleoconch whorl, right-slightly apical view. (F-G) paratype MZSP 152188 (L 27.9 mm) frontal and dorsal views. (H-J) paratype MZSP 152244 (L 29.5). (H) frontal view. (I) dorsal view. (J) anterior-slightly left view showing umbilicus. (K) holotype's jaw plate, ventral view. (L) same, anterior view. Scales= 0.5 mm

Fig 10. *Anthinus vailanti*, anatomy. (A) odontophore, dorsal view, superficial layer of muscles and structures removed, most muscles deflected, radular ribbon deflected to left. (B) middle region of genital system isolated, only topology of albumen gland shown. (C) genital system, mostly ventral view. (D) spermoviduct, transverse section in its middle level. Scales= 2 mm.

Fig 11. ***Anthinus vailanti*, radulae in SEM**, Paratypes MZSP 152118, 2 specimens. (A) wider vision, scale= 200 µm; (B) detail of central region, scale= 50 µm; (C) wide left view, scale= 100 µm; (D) wide view of another specimen, scale= 200 µm; (E) detail of central region, scale= 50 µm; (F) wide left view, scale= 200 µm. White arrows indicating rachidian column.

Figs 12. ***Anthinus vailanti*, anatomy**. (A) genital system, detail of anterior region opened longitudinally, penis and other adjacent structures only partially shown. (B) penis and epiphallus opened longitudinally. Scales= 2 mm.

Fig 13. ***Anthinus morenus*, shell of some types and anatomy**. (A-H) Holotype MZSP 152891 (L 35.8 mm). (A) Frontal view. (B) Right view. (C) Dorsal view. (D) Anterior view (W 21.0 mm). (E) Apical view. (F) Detail of protoconch and fist teleoconch whorl, profile. (G) Same, higher magnification. (H) Same, detail of apex. (I-L) Paratype MZSP 152047#1. (I) Jaw, in situ, ventral view. (J) Shell, frontal view (L 34.8 mm). (K) Shell, dorsal view. (L) Pallial cavity, ventral view, dorsal part of mantle edge and deflected to right, part of mantle floor also shown. Scales= 2 mm.

Fig 14. ***Anthinus morenus*, anatomy**. (A) Head-foot, head, digestive and genital structures removed, dorsal integument removed, main concern to main head-foot musculature. (B) Odontophore, dorsal view, superficial layer of muscles and structures removed, most muscles deflected. (C) Penis, sectioned longitudinally to show inner surface. (D) Genital system, mostly ventral view. (E) Middle region of genital system isolated, only topology of albumen gland shown. (F) Spermoviduct, transverse section in its middle level. Scales= 2 mm.

Fig 15. ***Anthinus morenus*, Radula in SEM**, paratype MZSP 152047. (A) wide view, scale= 200 µm; (B) detail of central region, scale= 200 µm; (C) detail of damaged region, with teeth in profile, scale= 50 µm; (D) detail of right region, scale= 50 µm; (E) wide view, scale= 500 µm; (F) detail of left region, scale= 200 µm. White arrows indicating rachidian column.

Fig 16. ***Anthinus savanicus*, shell of types and anatomical details**. (A-H) holotype MZSP 154391 (L 32.5 mm). (A) shell, frontal view. (B) right view. (C) dorsal view. (D), apical view. (E) inferior (umbilical) view. (F) protoconch and part of first teleoconch, profile, scale= 2 mm. (G) jaw, ventral view (L 1.7 mm). (H) almost entire specimen, mostly dorsal view, pallial cavity removed and deflected right (ventral view), head-foot deflected below, visceral mass slightly uncoiled, scale= 5 mm. (I-J) paratype MZSP 153897 (L 31.5 mm), frontal and dorsal views. (K) paratype MZSP 152171 (L 35.3 mm), frontal view.

Fig 17. ***Anthinus savanicus*, anatomical drawings**. (A) midgut as in situ, ventral view, some adjacent structures or their topology also shown, stomach and esophagus opened longitudinally. (B) odontophore, dorsal view, outer layer of structures removed, pair of odontophore cartilages deflected, both m4 and left m5 (right in Fig.) also deflected. (C) genital structures, mostly ventral view, most vas deferens (dv) detached. (D) spermoviduct, transverse section of its middle level. Scales= 2 mm.

Fig 18. ***Anthinus savanicus*, radulae in SEM**. (A) holotype MZSP 154391. Detail of central region, scale= 50 µm; (B) same, detail of column in profile, scale= 50 µm; (C) same, detail of right region, scale= 200 µm; (D) paratype MZSP 153897, detail of right region, scale= 50 µm; (E) same, central region, scale= 100 µm. White arrows indicating rachidian column.

Fig 19. ***Anthinus savanicus*, anatomy, genital system**. (A) carrefour region, ventral view, seen if albumen gland (ag) was transparent, spermoviduct (eo) transversely sectioned in its posterior region; (B) anterior end, ventral view, entirely opened longitudinally, portions of adjacent structures also shown. (C) penis, ventral view, entirely opened longitudinally, portions of adjacent structures also shown. Scales= 2 mm.

Fig 20. *Catracca uhlei*, shells and jaw. (A-C) Holotype MZSP 151900 (L 44.2 mm). (A) Frontal view. (B) right view. (C) dorsal view. (D) Paratype MZSP 151901#1 (L 42.8 mm), frontal view; (E) holotype protoconch in profile, scale= 3 mm. (F) paratype 151893, shell of one of dissected specimens, frontal view (L 47.1 mm). (G) Paratype MZSP 151901#2 (L 43.0 mm). (H) Paratype MZSP 151871, young specimen, detail of apex, profile-slightly apical view, scale= 2 mm. (I) Same, apical view, scale= 2 mm. (J) Jaw plate, ventral view (slightly broken in left end, right in Fig.), scale= 1 mm.

Fig 21. *Catracca uhlei*, anatomy. (A) Pallial cavity roof, ventral view, pneumostome ventral lip sectioned and deflected upwards. (B) Head-foot, head, digestive and genital structures removed, anterior integument sectioned and deflected to left (right in Fig.), main concern to main head-foot musculature. (C) Fore- and midgut, mostly ventral view, shown as in situ, topology of some adjacent structures also shown. (D) Stomach, ventral view, mostly sectioned longitudinally to show inner surface. (E) Buccal mass, left view. Scales= 5 mm.

Fig 22. *Catracca uhlei*, radula in SEM, holotype MZSP 151893. (A) wide view, scale= 300 µm; (B) detail of central region, scale= 100 µm; (C) detail of lateral region, scale= 100 µm; (D) detail of central region, scale= 50 µm; (E) same, higher magnification, scale= 30 µm; (F) same, higher magnification, scale= 10 µm. White arrows indicating rachidian column.

Fig 23. *Catracca uhlei*, anatomy. (A) Odontophore, dorsal view, superficial layer of muscles and structures removed, left muscles deflected. (B) Buccal mass, ventral view, sectioned longitudinally, odontophore deflected to left. (C) Genital system, mostly ventral view. (D) Middle region of genital system isolated. (E) Spermoviduct, transverse section in its middle level. (F) Anterior region of genital system, ventral view, female portion sectioned longitudinally to show inner surface. Scales= 2 mm.

Fig 24. *Catracca uhlei*, anatomy. (A) Penis, dorsal view. (B) Same, sectioned longitudinally to show inner surface. (C) Nerve ring, dorsal view. Scales= 2 mm.

Fig. 25. Gallery of shells of all *Anthinus* species, frontal and dorsal views. (A). *A. albolabiatus*, MZSP 7958 (L 50 mm). (B) *A. miersi*, MZSP 7963 (L 53 mm). (C) *A. multicolor*, MZSP 29620 (L 26 mm). (D) *A. turnix*, holotype USNM 712805 (L 50 mm). (E) *A. synchondrus* holotype MZSP 152074 (L 36.9 mm); (F) *A. vailanti* holotype MZSP 152891 (L 29.0 mm). (G) *A. morenus* holotype MZSP 152891 (L 35.8 mm). (H) *A. savanicus* holotype MZSP 154391 (L 32.5 mm). (I) *Catracca uhlei* holotype MZSP 151900 (L 44.2 mm). A to D extracted from Simone (2006).

Fig. 26. Gallery of shells of all *Anthinus* species in same scale, frontal views. (A). *A. albolabiatus*, MZSP 7958. (B) *A. miersi*, MZSP 7963. (C) *A. multicolor*, MZSP 29620. (D) *A. turnix*, holotype USNM 712805. (E) *A. synchondrus* holotype MZSP 152074. (F) *A. vailanti* holotype MZSP 152891. (G) *A. morenus* holotype MZSP 152891. (H) *A. savanicus* holotype MZSP 154391. (I) *Catracca uhlei* holotype MZSP. A to D extracted from Simone (2006). Scale= 10 mm.

Fig. 27. Brazil map with states outlined and type localities of each *Anthinus* (*A.*) and *Catracca* shown. New localities in dark blue. Previous localities in light blue. States abbreviations: GO: Goiás; MG: Minas Gerais; RJ: Rio de Janeiro; RS: Rio Grande do Sul.

Fig. 28. Single cladogram obtained analyzing the matrix (Appendix 2) of the species assembly reported in Material and Methods (written black), plus the species studied herein (written brown). Both branches below are far outgroups, a bulimulid (*Leiostracus*) and a simpulopsid (*Rhinus*). Branch *15* as close outgroups, two African dorcasiids. Each branch with

synapomorphies shown. Each symbol as in the box; above number the character, below number the state. Branch numbers for discussion in blue italics. Method: parsimony; L= 283; CI= 68; RI= 89.

Appendix 1 – list of characters that base the phylogenetic analysis

1. Shell walls: 0= thin; 1= thick.
2. Shell form: 0= bulimoid; 1= discoid.
3. Shell size: 0= 2-3 cm; 1= 4-5 cm; 2= over 6 cm.
4. Shell color: 0= monochromatic; 1= mosaic of spots.
5. Periostracum: 0= permanent; 1= deciduous.
6. Aperture: 0= smaller than half of shell length; 1= longer than half of shell length.
7. Protoconch sculpture: 0= smooth or reticulate; 1= reticulate with axial predominance; 2= irregular axial threads; 3 regular narrow axial cords.
8. Protoconch form: 0= pointed; 1= blunt.
9. Head peri-oral flange: 0= absent; 1= present.
10. Haemocoel layers of head rectractor muscles: 0= two; 1= single.
11. Inner layer of head retractors: 0= only present in ommatophores; 1= ommatophores and peribuccals separated; 2= ommatophore and peribuccal fused.
12. Mantle edge inner pneumostome fold: 0= simple; 1- angulous.
13. Differentiated vessel close to pneumostome: 0= absent; 1= present.
14. Afferent pallial pulmonary vessels: 0= single; 1= a pair.
15. Pulmonary septum: 0= absent; 1= present.
16. Nephropore: 0= protected in primary ureter; 1= exposed in pallial cavity.
17. Nephropore: 0= simple; 1= protected by anterior renal fold (? in outgroup).
18. Kidney: 0= smooth; 1= covered by anastomosed net of vessels.
19. Urinary pallial way: 0=via duct; 1= via groove.
20. Ureter groove: 0= smooth; 1= transversely folded.
21. Jaw plate: 0= smooth; 1= transversely folded; 2: narrow.
22. Horizontal muscle (m6): 0= present; 1= absent.
23. Insertion of pair m5: 0= on pair m4; 1= partially on cartilages.
24. Pair of muscles inside radular sac (m7): 0= present; 1= absent.
25. Inner edge of cartilages (anterior to fusion): 0= thick; 1= thin.
26. Radular teeth: 0= rachidial similar to lateral; 1= different.
27. Radular teeth: 0= bi- or multicuspid; 1= unicuspid.
28. Radular mesocone: 0= smaller than base; 1= as long as base; 2= longer than base.
29. Radular mesocone: 0= stubby; 1= flattened, spatula-like.
30. Radular mesocone tip: 0= pointed; 1= rounded.
31. Tissue on radula at end of radular sac (to): 0= simple; 1=reinforced.
32. Salivary duct between salivary gland and buccal mass: 0= long; 1= short.
33. Esophageal duct to anterior lobe of digestive gland: 0= far from stomach; 1= close to stomach.
34. Esophageal duct to anterior lobe of digestive gland: 0= single; 1= closely bifid
35. Stomach: 0= non-muscular; 1= weakly muscular; 2= highly muscular; 3= high muscular connected to typhlosole (add).
36. Stomach inner folds in its smaller curve: 0= absent; 1= present; 2= extending to remaining stomach (add).
37. Pre-rectal valve: 0= absent; 1= present.
38. Pre-rectal pallial muscle: 0= absent; 1= present.
39. Anus: 0= turned outside; 1= opening inside pallial cavity.
40. Seminal receptacle: 0= balloon-like; 1= U-shaped.
41. U-shaped seminal receptacle: 0= widely curved; 1- tightly curved.

42. Talon (swollen end of hermaphroditic duct) in carrefour; 0= absent; 1= present.
43. Duct of albumen gland: 0= connected in spermoviduct; 1= connected directly to albumen chamber.
44. Spermoviduct accessory gland: 0= absent; 1= small; 2= large.
45. Prostate: 0= narrow (~1/3 of diameter); 1= wide (~1/2).
46. Vaginal appendix: 0= absent; 1= present.
47. Epiphallus: 0= conic, long; 1= short, rounded; 2= pseudo-epiphallus; 3= conic, short.
48. Flagellum: 0= absent; 1= single and small.
49. Penis shield: 0= present; 1= absent.
50. Penis retractor muscle: 0= simple; 1= multiple.
51. Penis retractor muscle insertion: 0= in penis tip only; 1= branches in epiphallus.
52. Penis with transverse fold (“penial papilla”): 0= absent; 1= present, simple; 2= with sphincter.
53. Pair of parallel inner folds connecting epiphallus and penis: 0= absent; 1= present.
54. Penis inner surface: 0= smooth; 1= papillate; 2= with large longitudinal fold in a side (pilar).
55. Spawn: 0= minute and numerous capsules; 1= few, large capsules.
56. Capsules cover: 0= thin, weakly calcified; 1= thick, well-calcified (egg like).

Appendix 2 – matrix of characters that base the phylogenetic analysis

**Species\ character**	**1**	**2**	**3**	**4**	**5**	**5**
	**1234567890**	**1234567890**	**1234567890**	**1234567890**	**1234567890**	**123456**
Mirinaba antoninensis	1010011000	1100011010	0111101100	0111110001	?100001011	111111
Mirinaba cadeadensis	1010011000	1100011010	0111101100	0111110001	?100001011	111111
Megalobulimus lopesi	1020002111	0010110111	1101011100	1100221110	?102003010	000211
Megalobulimus grandis	1020012111	0010110111	1101011100	1100221110	?102001010	100211
Megalobulimus parafragilior	1020012111	0010110111	1101011100	1100221110	?102001010	100211
Megalobulimus proclivis	1020012111	0010110111	1101001100	1100321110	?002011010	000211
Megalobulimus oblongus	1020103111	0010110111	1101001100	1100321110	?002110110	000211
Megalobulimus conicus	1020103111	0010110111	1101001100	1100321110	?002110110	000211
Megalobulimus driades	1020003111	0010110111	1101001100	1100321110	?002110110	000211
Strophocheilus debilis	1010002000	0100011010	0111101100	0111110000	?101002010	010111
Catracca uhlei	1010003100	1100011011	0101001100	0111110000	?000002010	020111
Anthinus multicolor	1001001000	2000011010	0111101111	0111110001	0002001010	101111
Anthinus synchondrus	1011001000	2100011010	2111101211	0111110101	1012001010	101111
Anthinus vailanti	1010001000	2100011010	0111101211	0111110101	1002001010	101111
Anthinus morenus	1011001000	2100011010	2111101211	0111110101	1012001010	101111
Anthinus savanicus	1011001000	2100011010	0111101211	0111110101	1012001010	101111
Dorcasia akexandri	0100000000	0001011010	000?000000	0000010000	?002000000	000011
Trigonephrus porphyrostoma	0100?00000	0001011010	0????00000	0000010000	?0?2000000	000011
Leiostracus carnavalescus	0000000000	0000000000	0000000000	0000000000	?000000000	000000
Rhinus botocudus	0000000000	0000000000	0000000000	0000000000	?000000000	000000

Figure 01

Click here to access/download;Figure;01b Anthinus multicolor 00.jpg

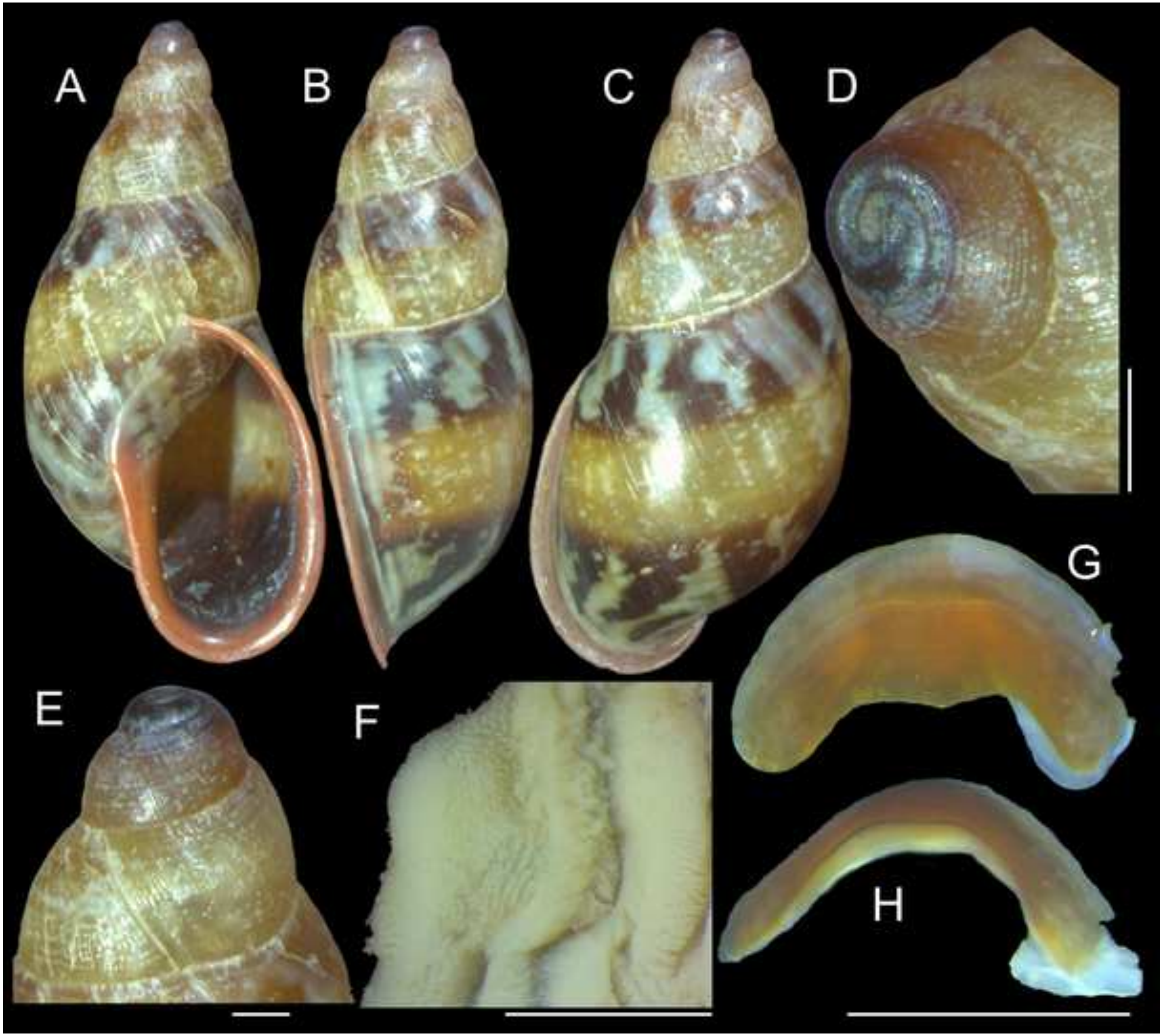

Figure 02

Click here to access/download;Figure;02b Anthinus multicolor 01.jpg

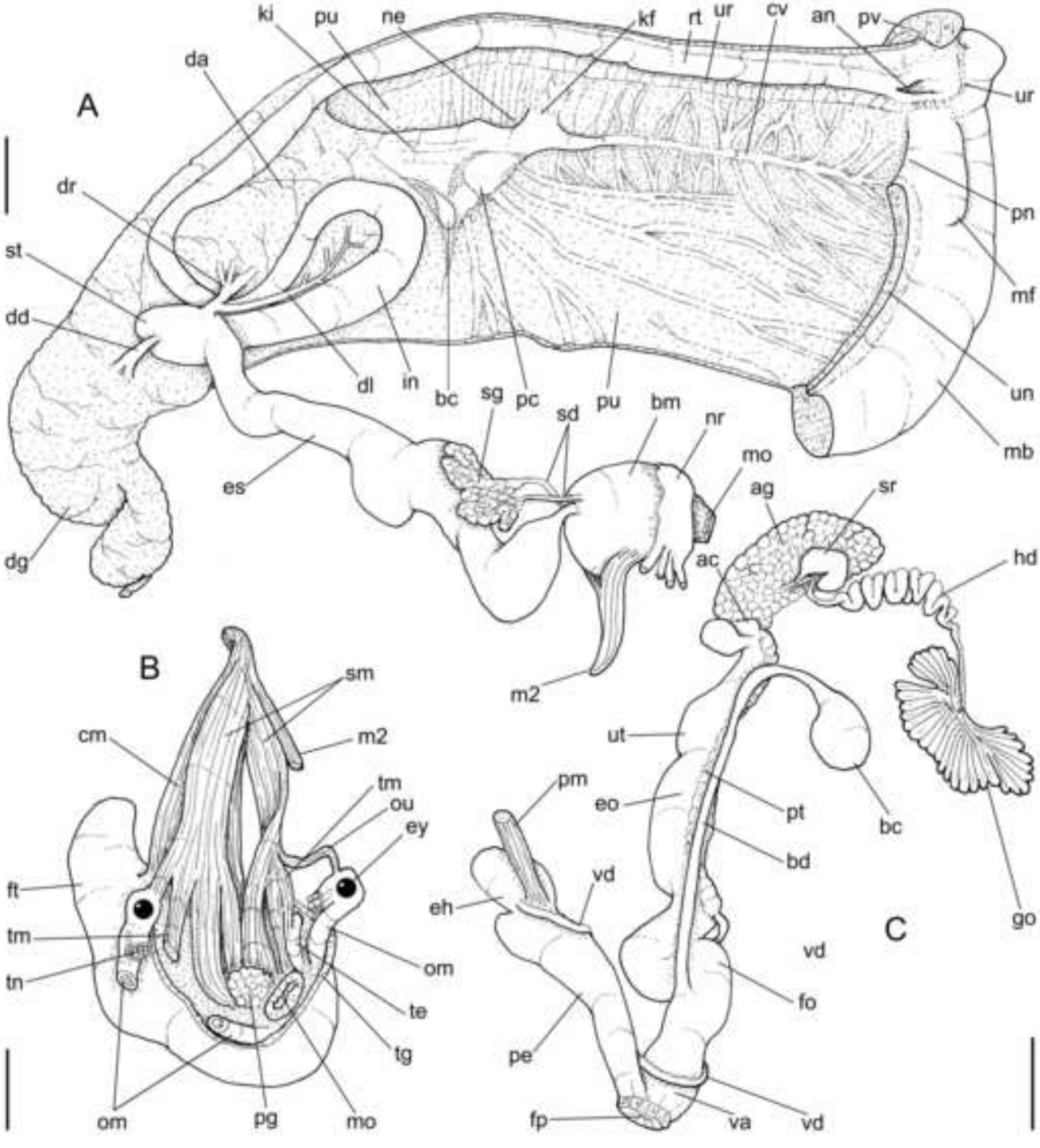

Click here to access/download;Figure;03b Anthinus multicolor Rad.jpg

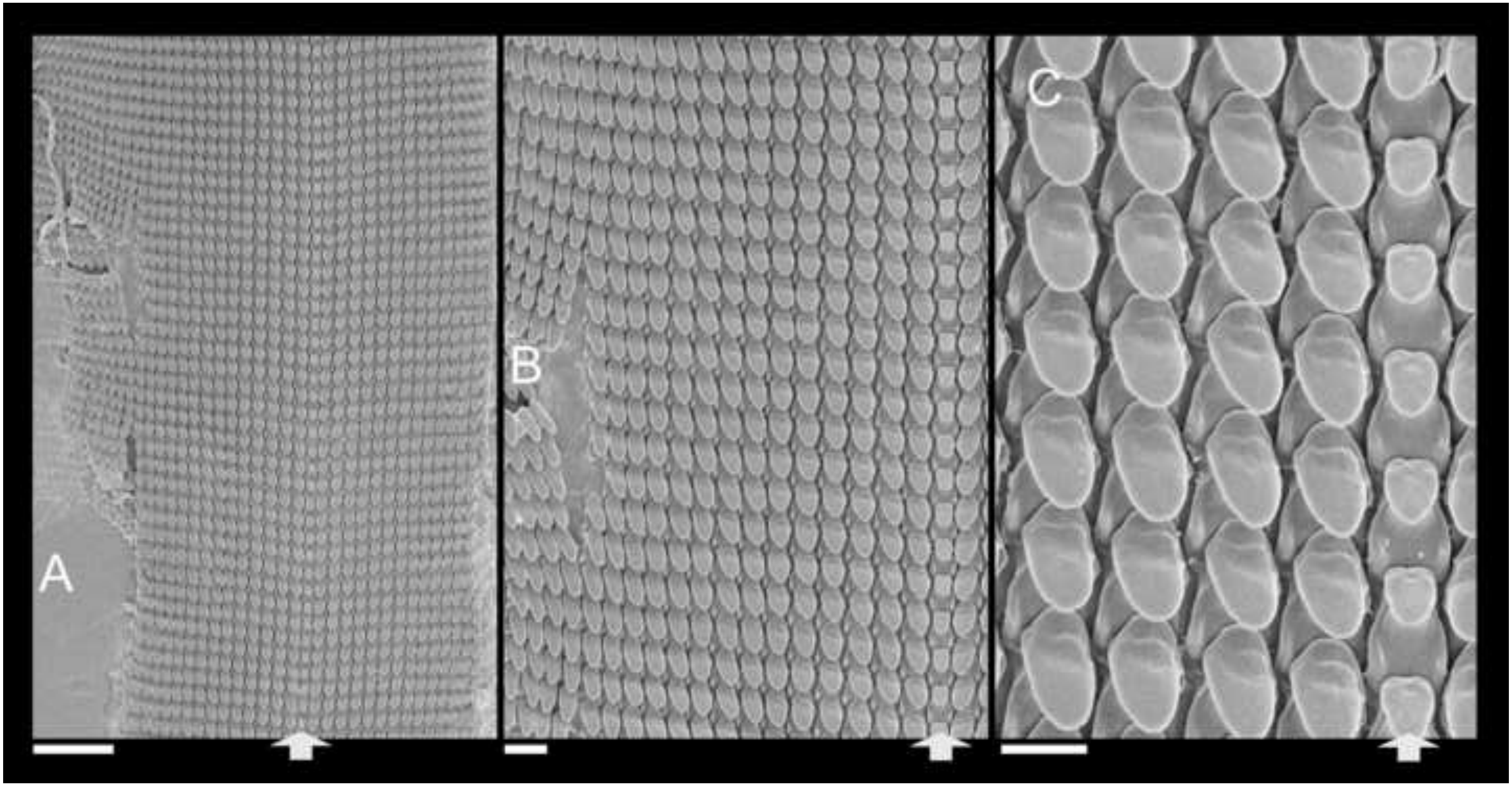

Figure 04

Click here to access/download;Figure;04b Anthinus multicolor 02.jpg

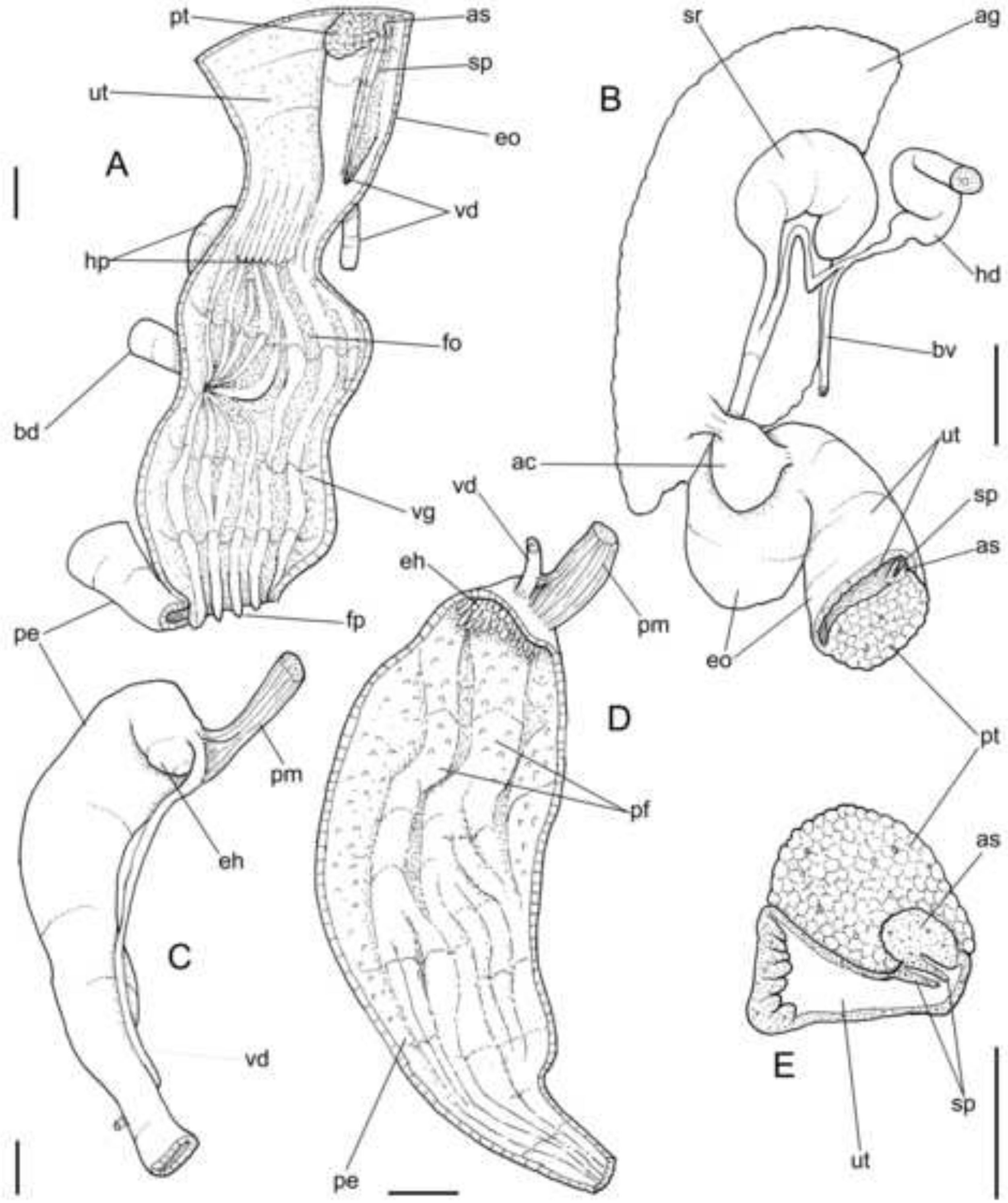

Figure 05

Click here to access/download;Figure;05b Anthinus synchondrus.jpg

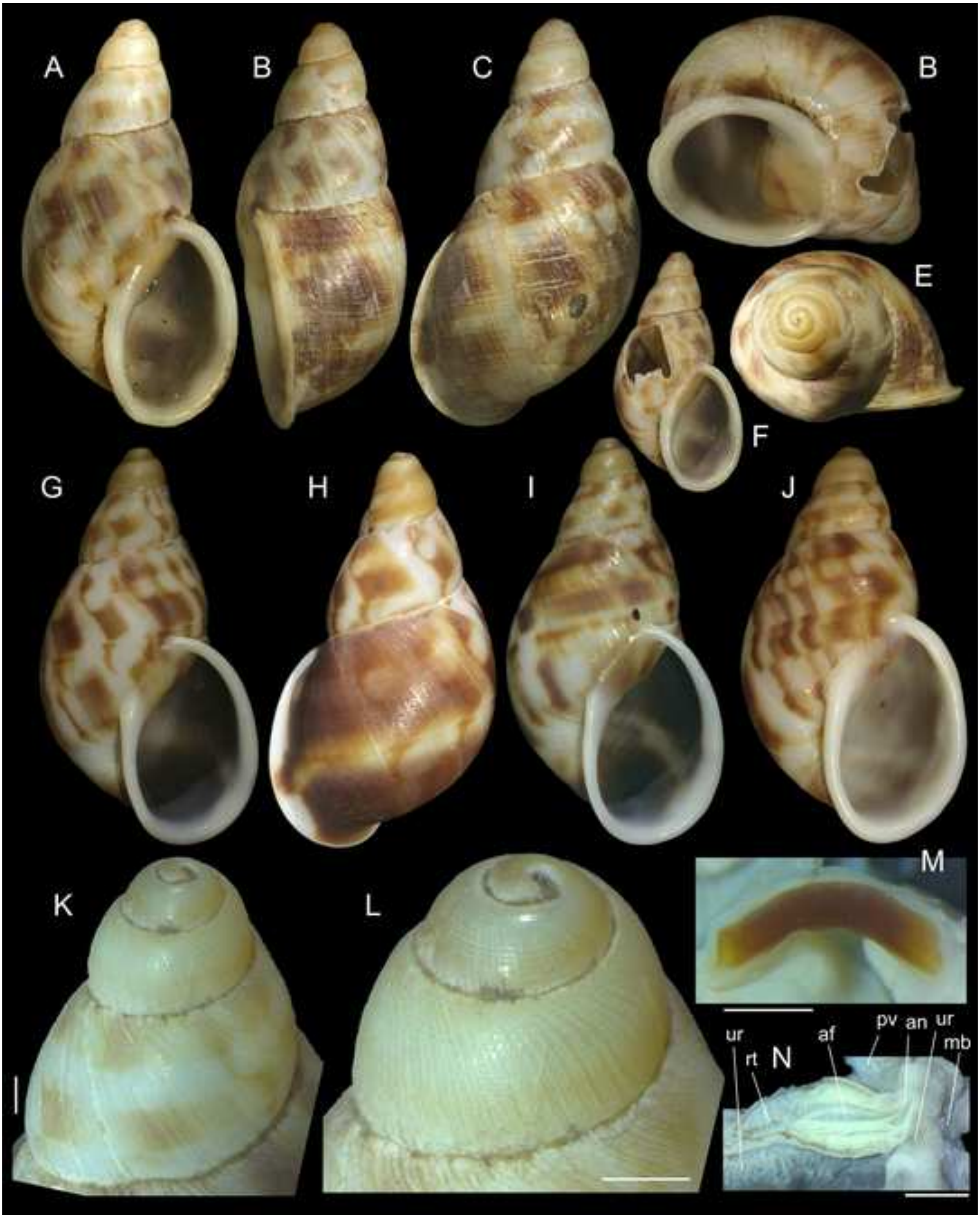

Figure 06

Click here to access/download;Figure;06b Anthinus synchondrus.jpg

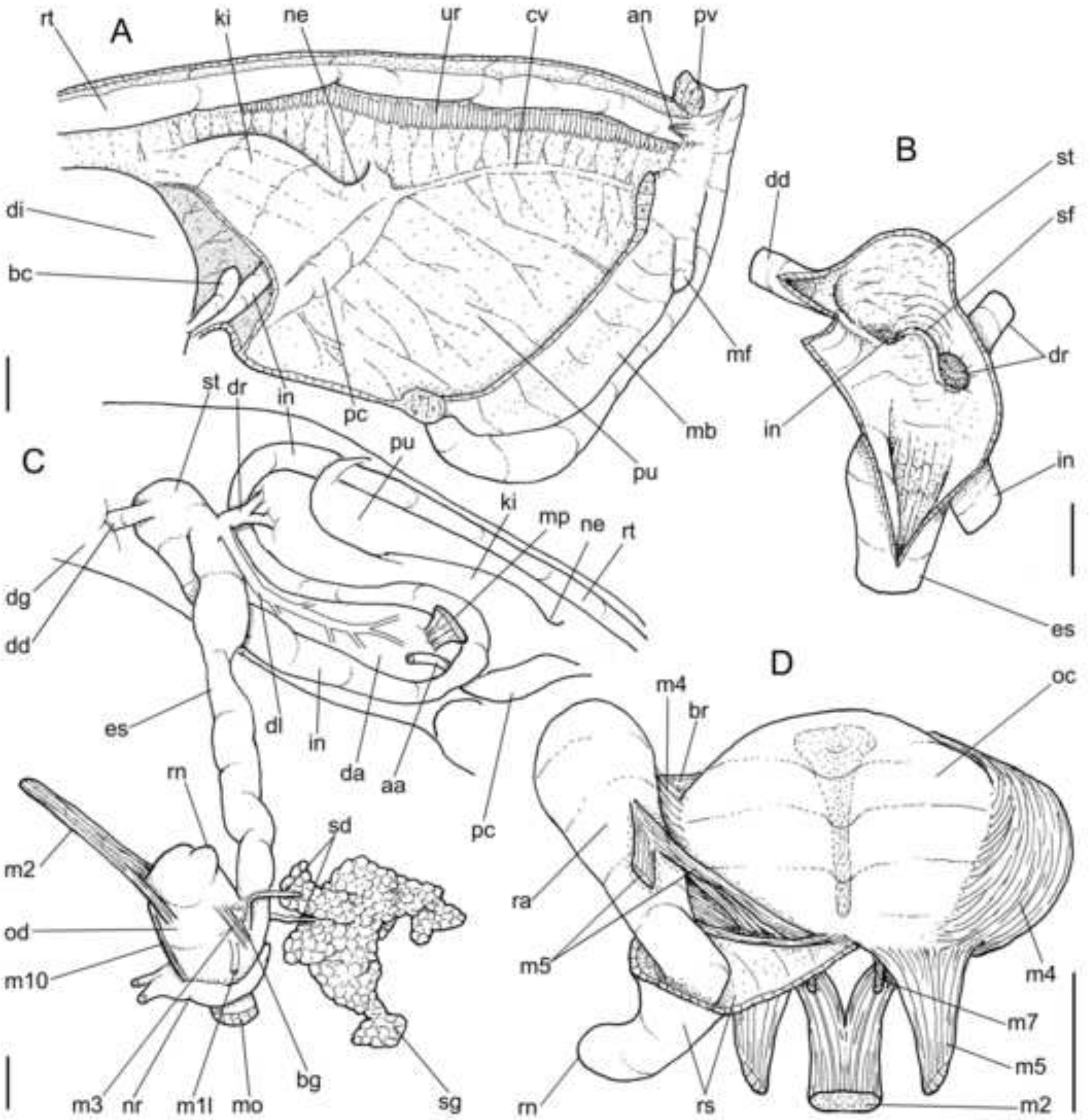

Figure 07

Click here to access/download;Figure;07b Anthinus synchondrus Rad.jpg

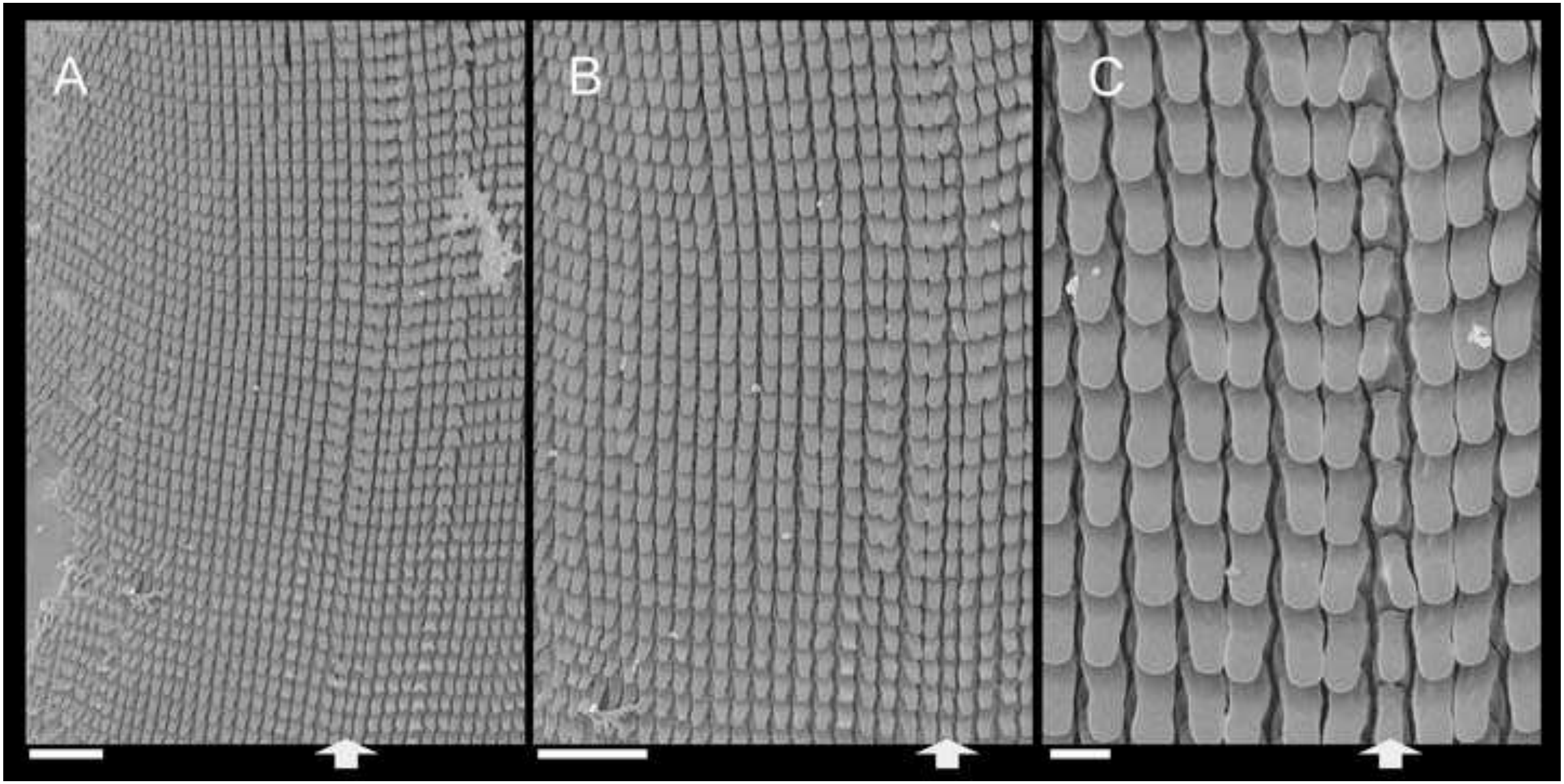

Figure 08

Click here to access/download;Figure;08b Anthinus synchondrus.jpg

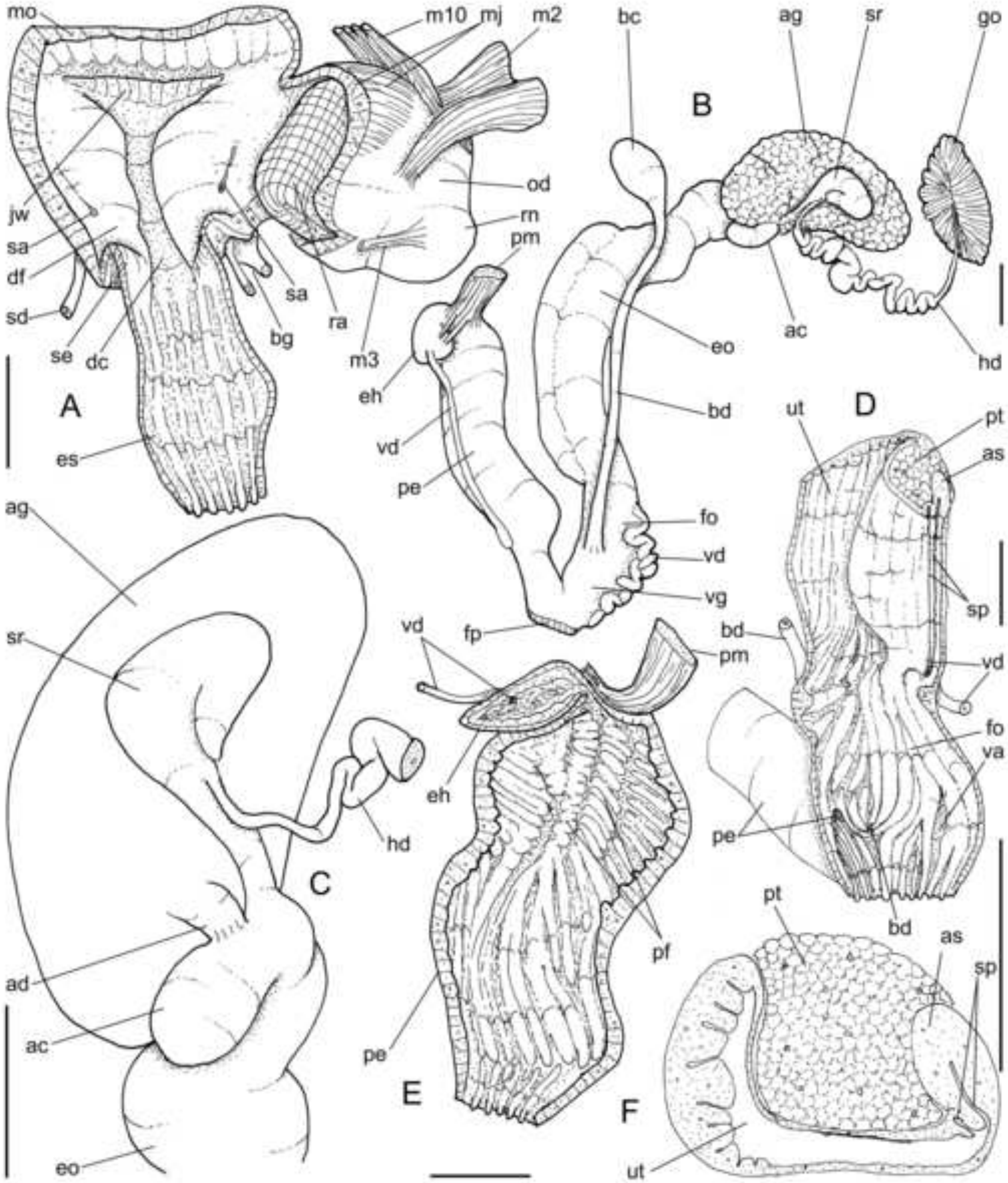

Figure 09

Click here to access/download;Figure;09b Anthinus vailanti.jpg

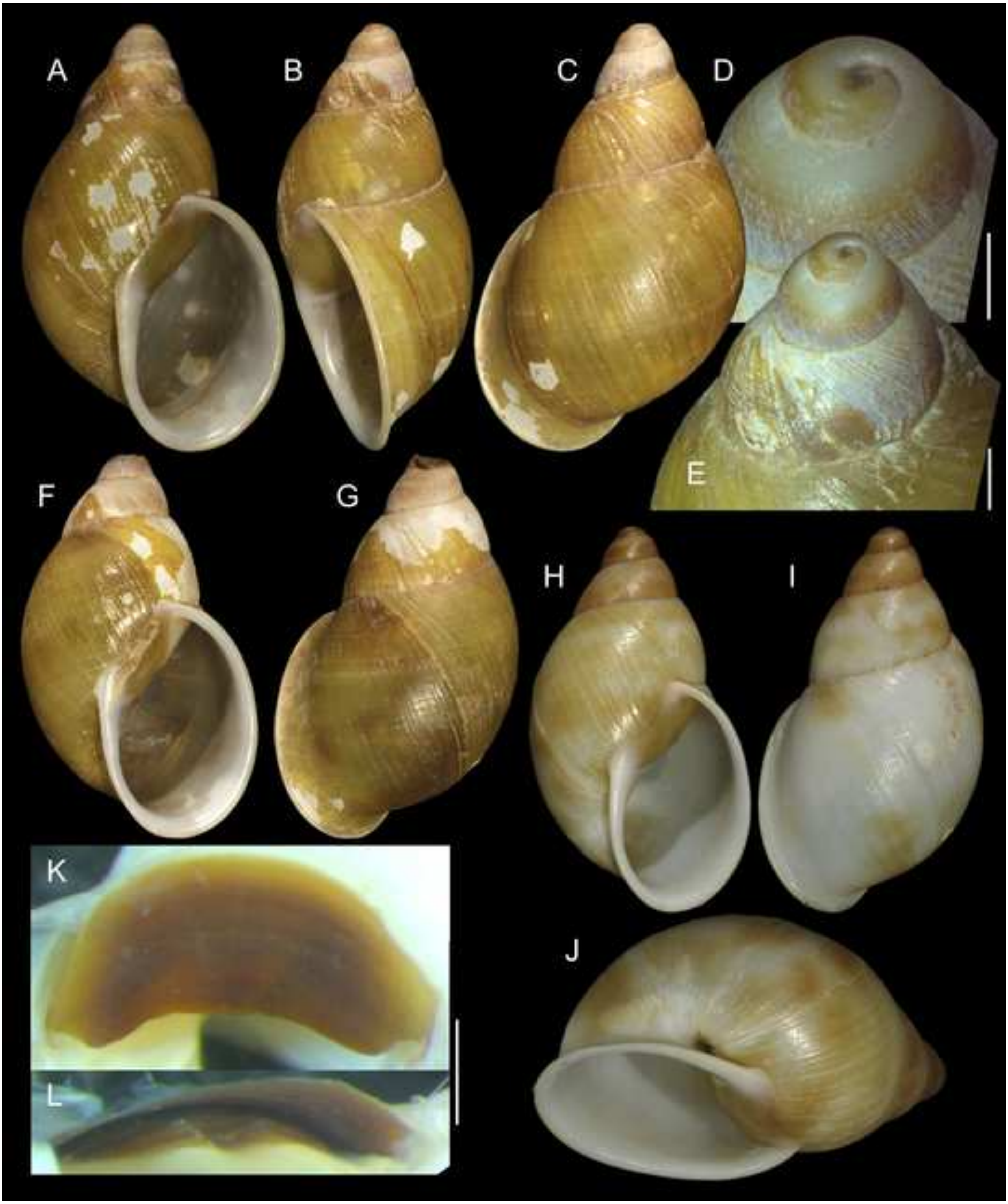

Click here to access/download;Figure;10b Anthinus vailanti.jpg

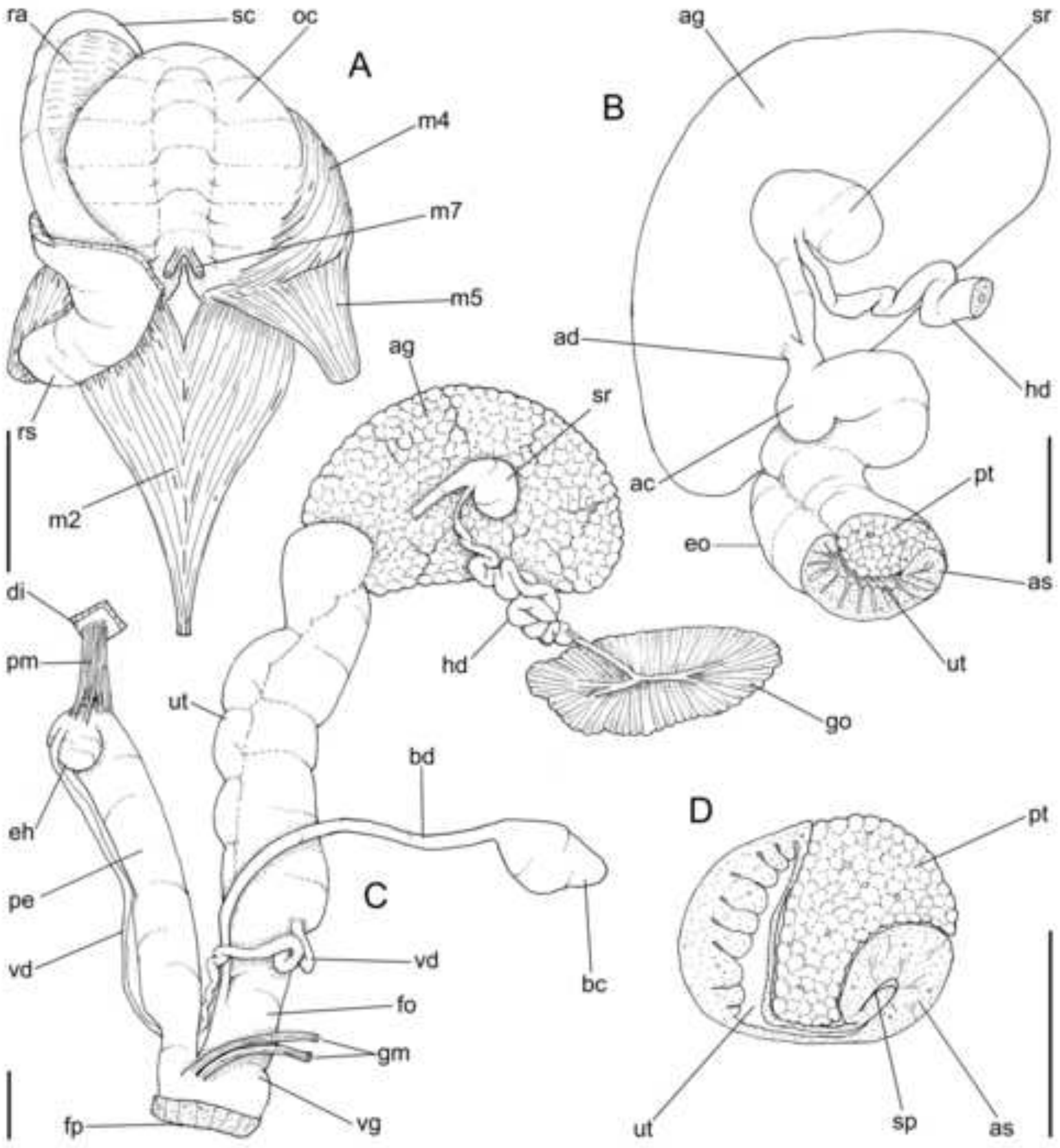

Figure 11

Click here to access/download;Figure;11b Anthinus vailanti.jpg

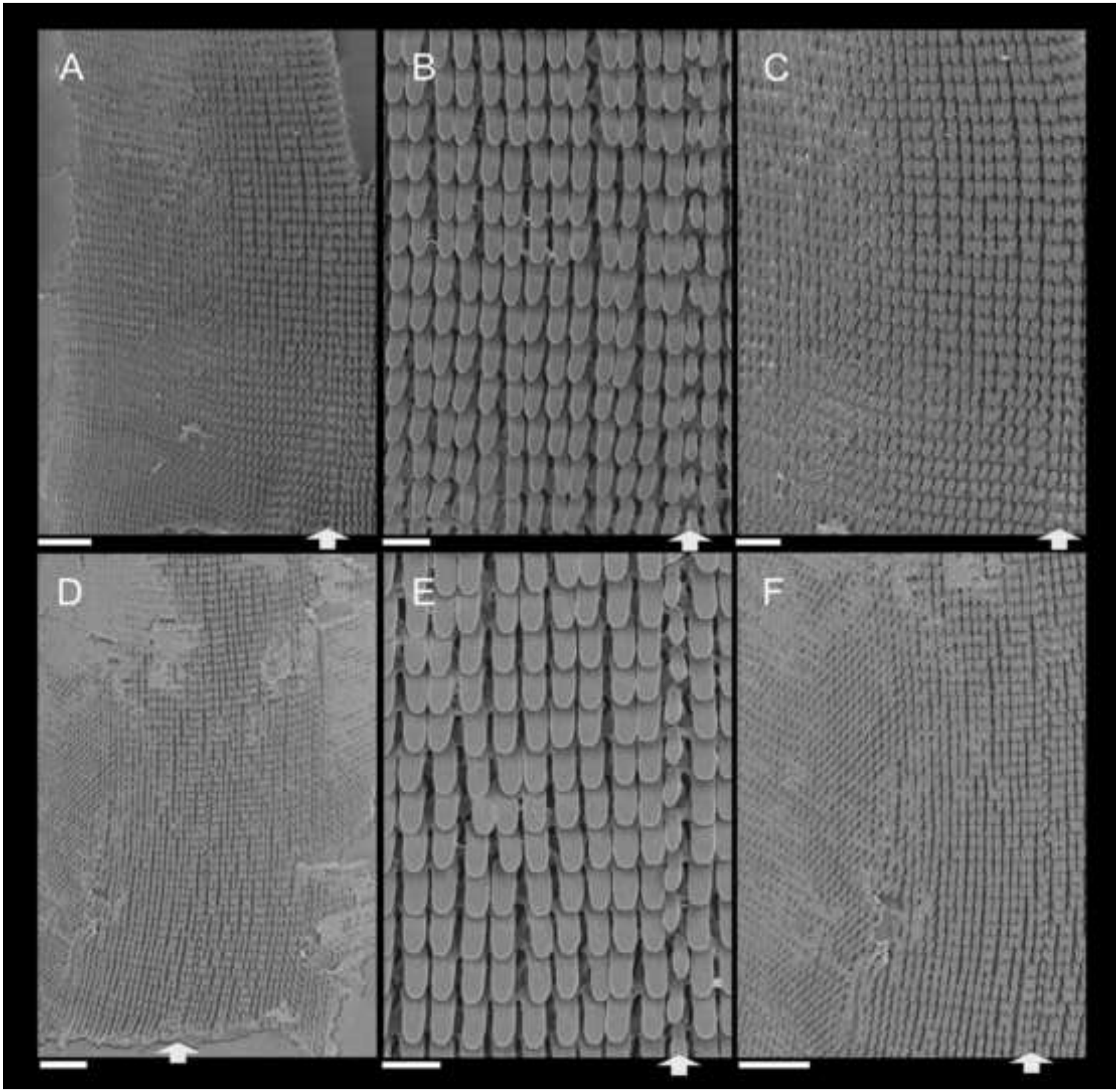

Figure 12

Click here to access/download;Figure;12b Anthinus vailanti.jpg

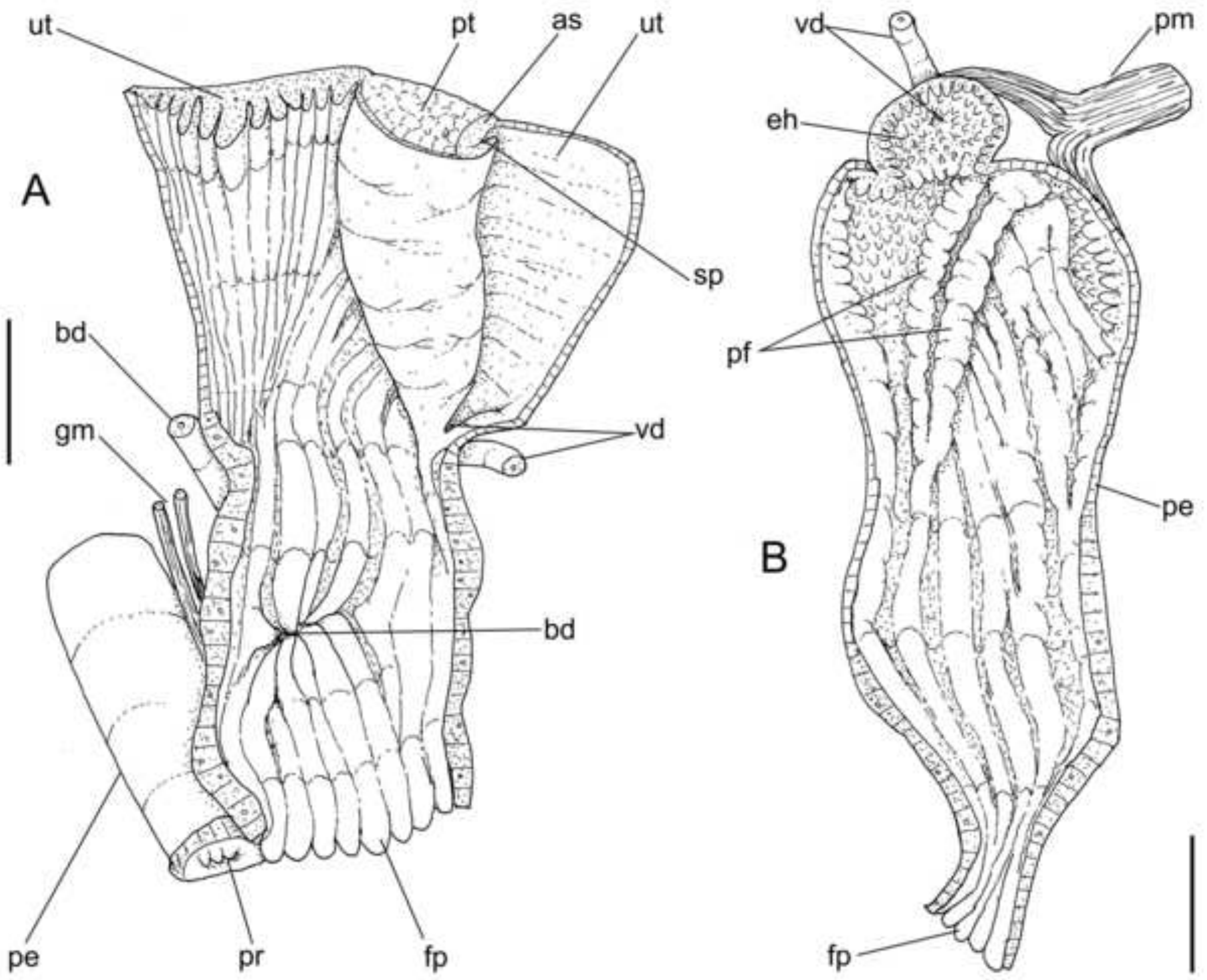

Figure 13

Click here to access/download;Figure;13b Anthinus morenus.jpg

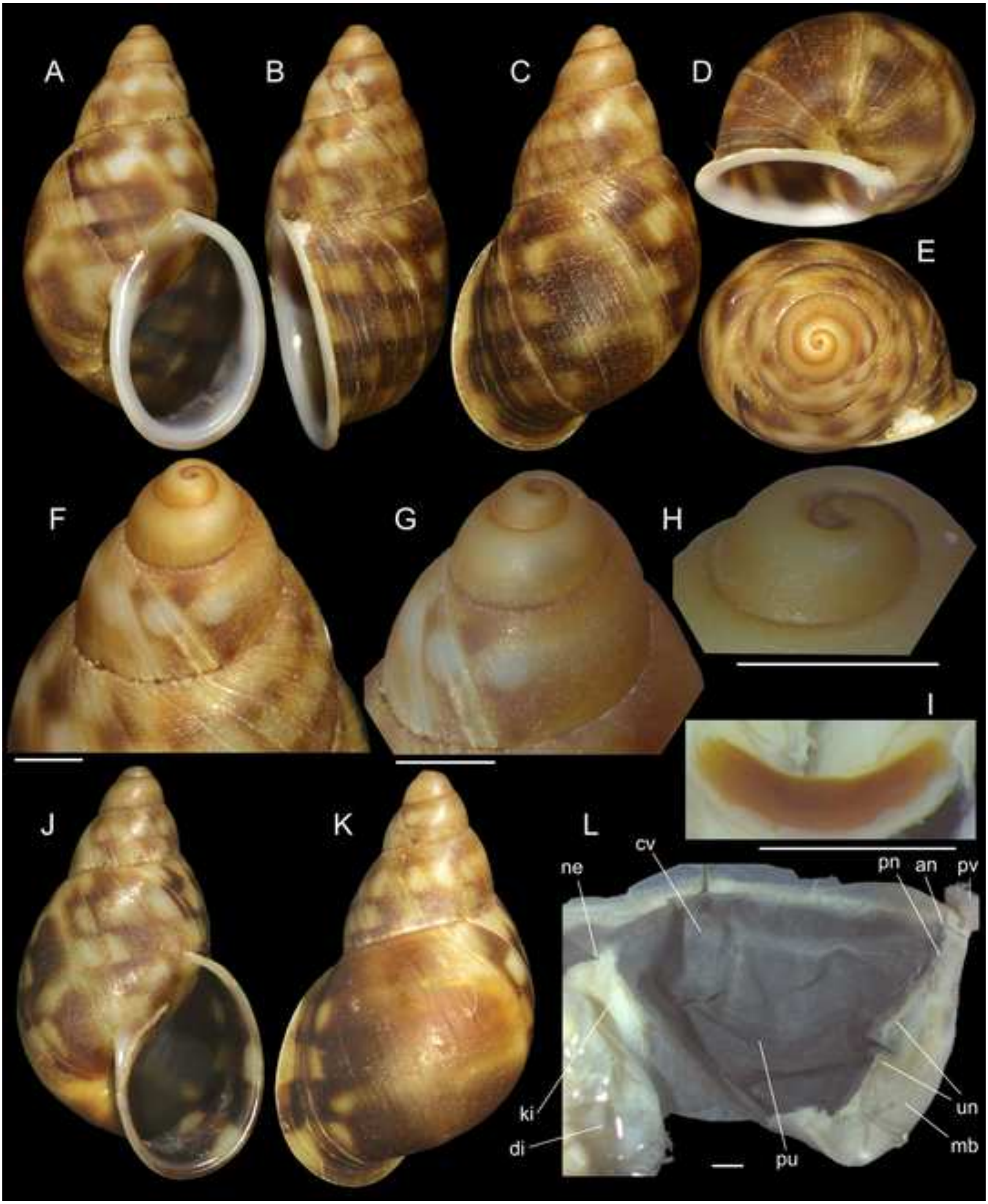

Click here to access/download;Figure;14b Anthinus morenus.jpg

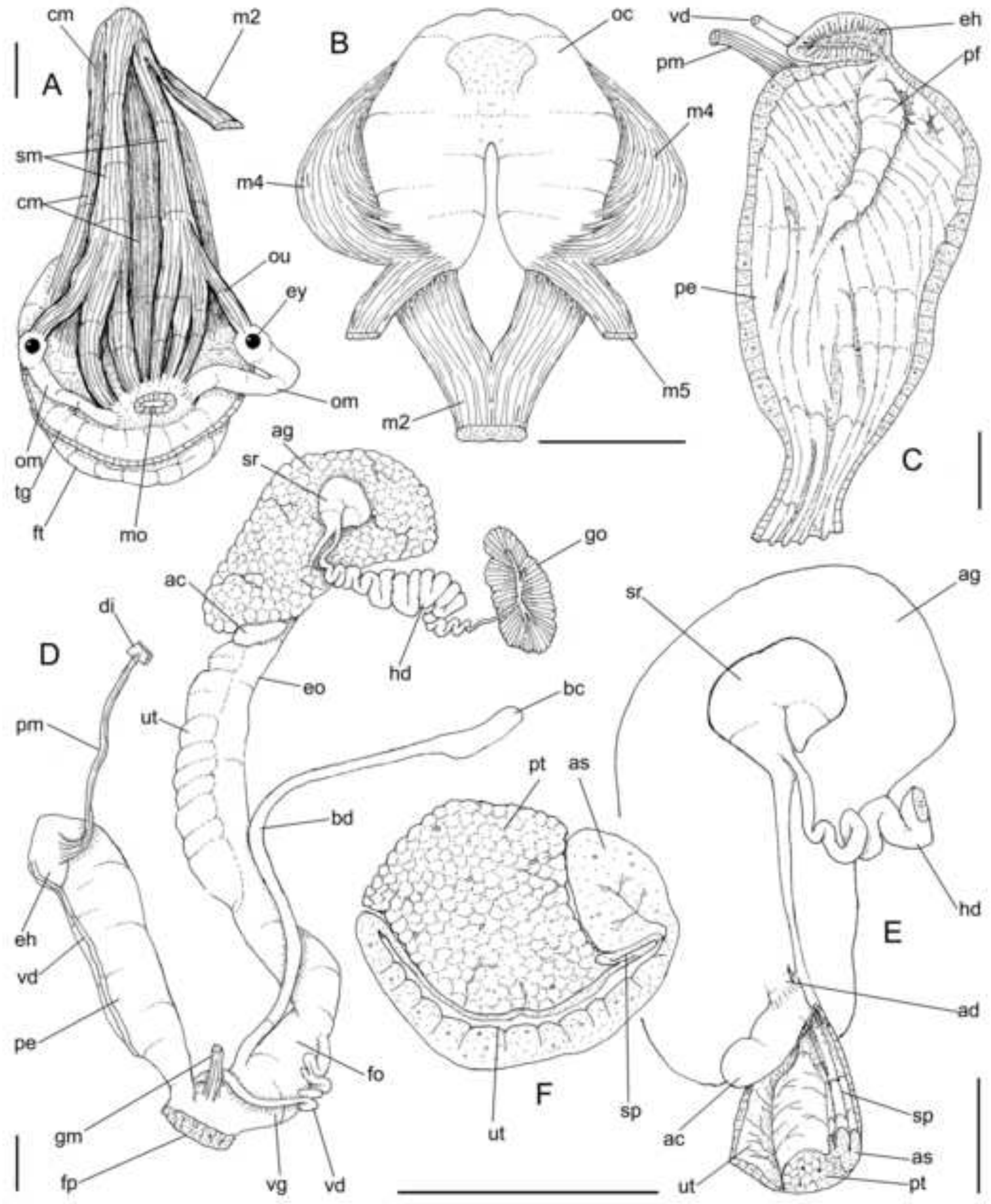

Figure 15

Click here to access/download;Figure;15b Anthinus morenus.jpg

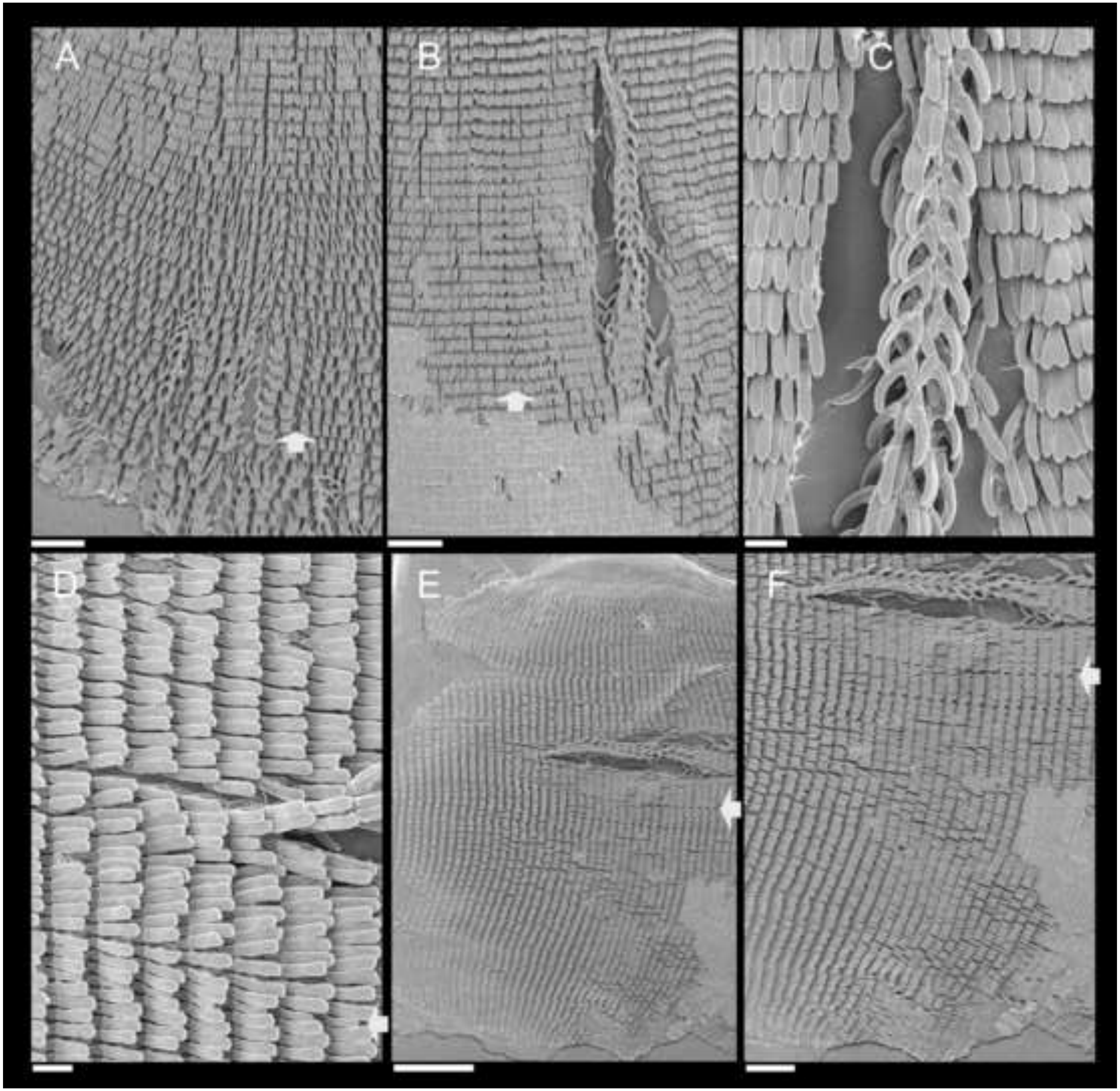

Figure 16

Click here to access/download;Figure;16b Anthinus savanicus.jpg

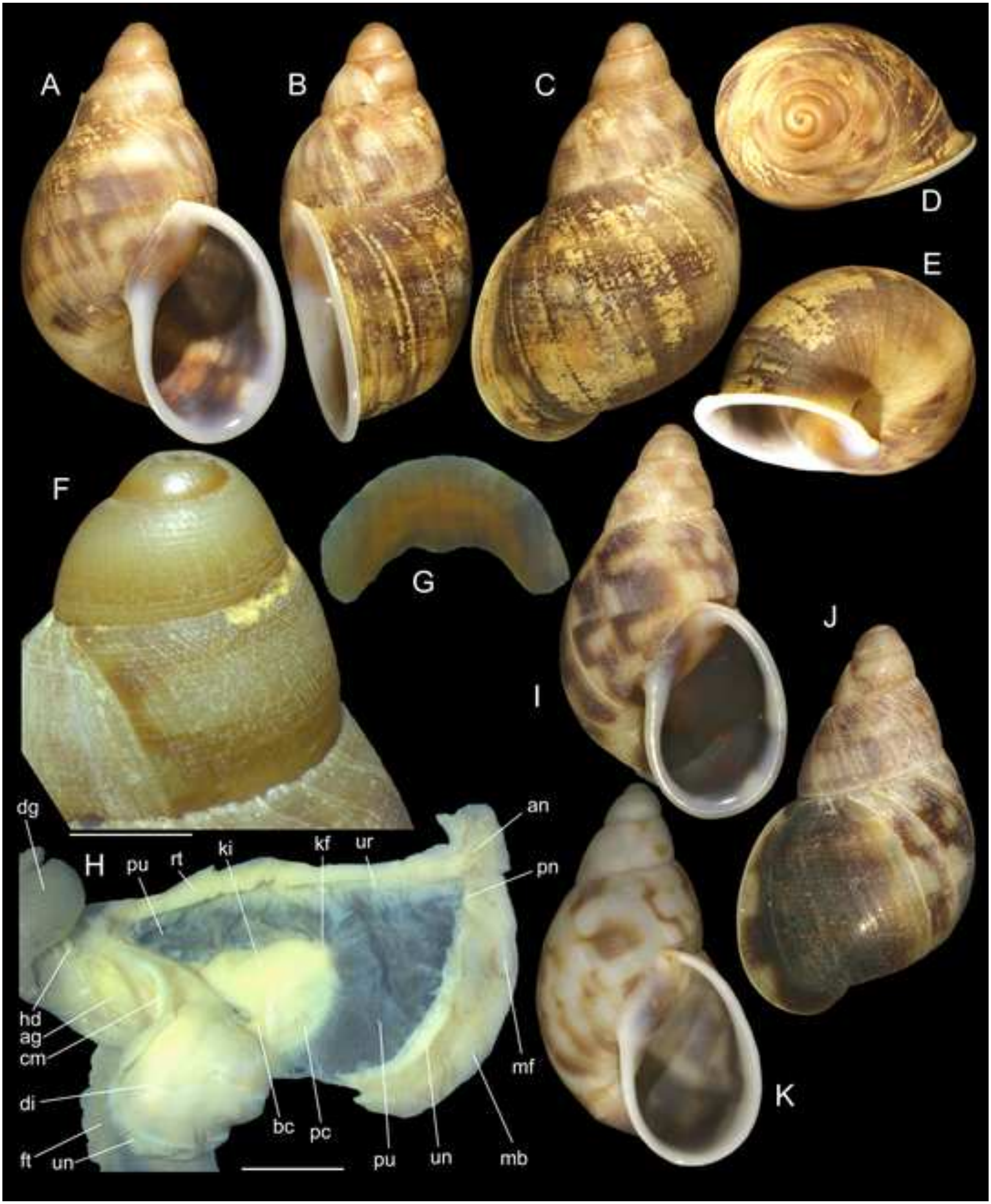

Figure 17

Click here to access/download;Figure;17b Anthinus savanicus.jpg

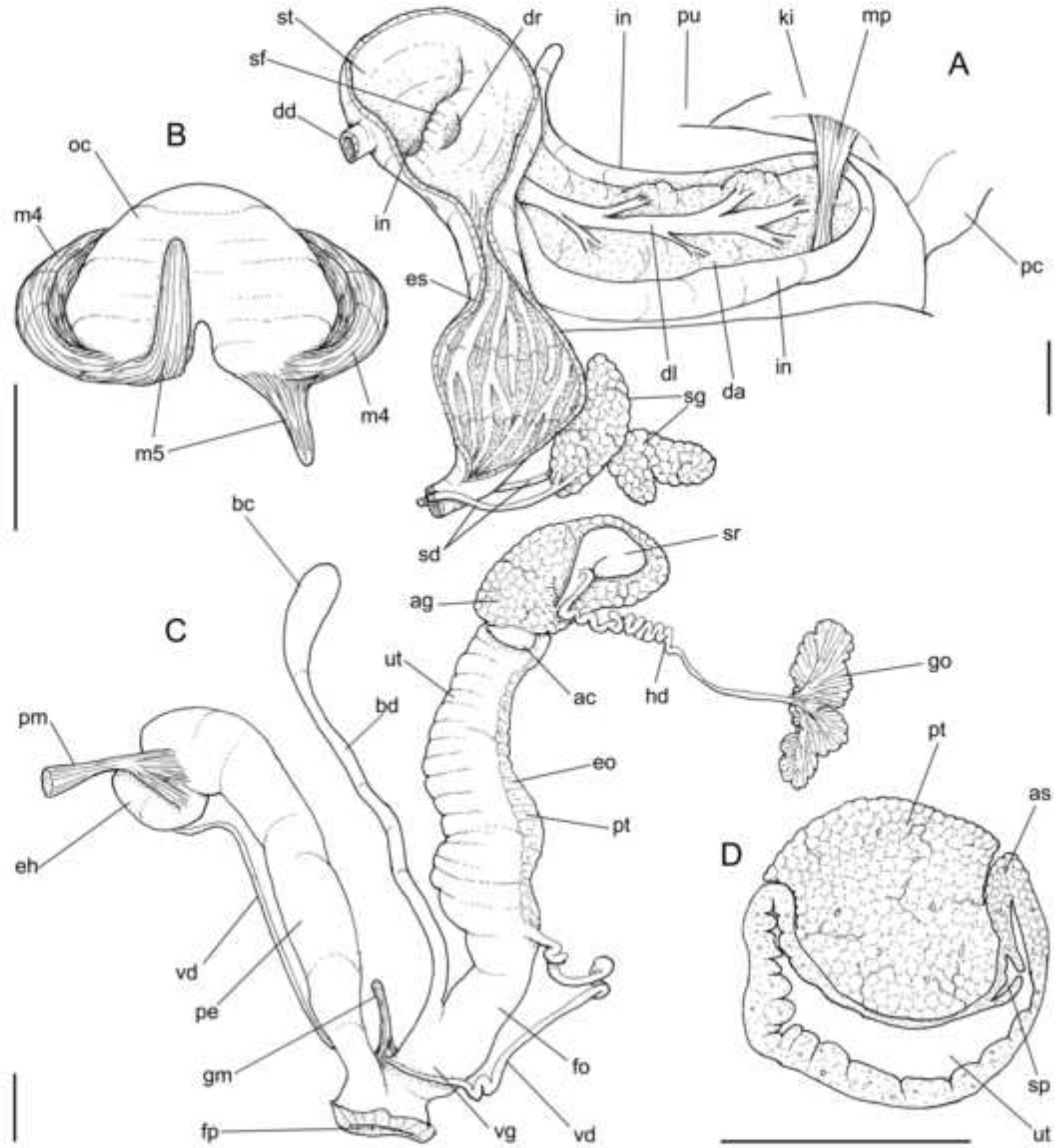

Figure 18

Click here to access/download;Figure;18b Anthinus savanicus.jpg

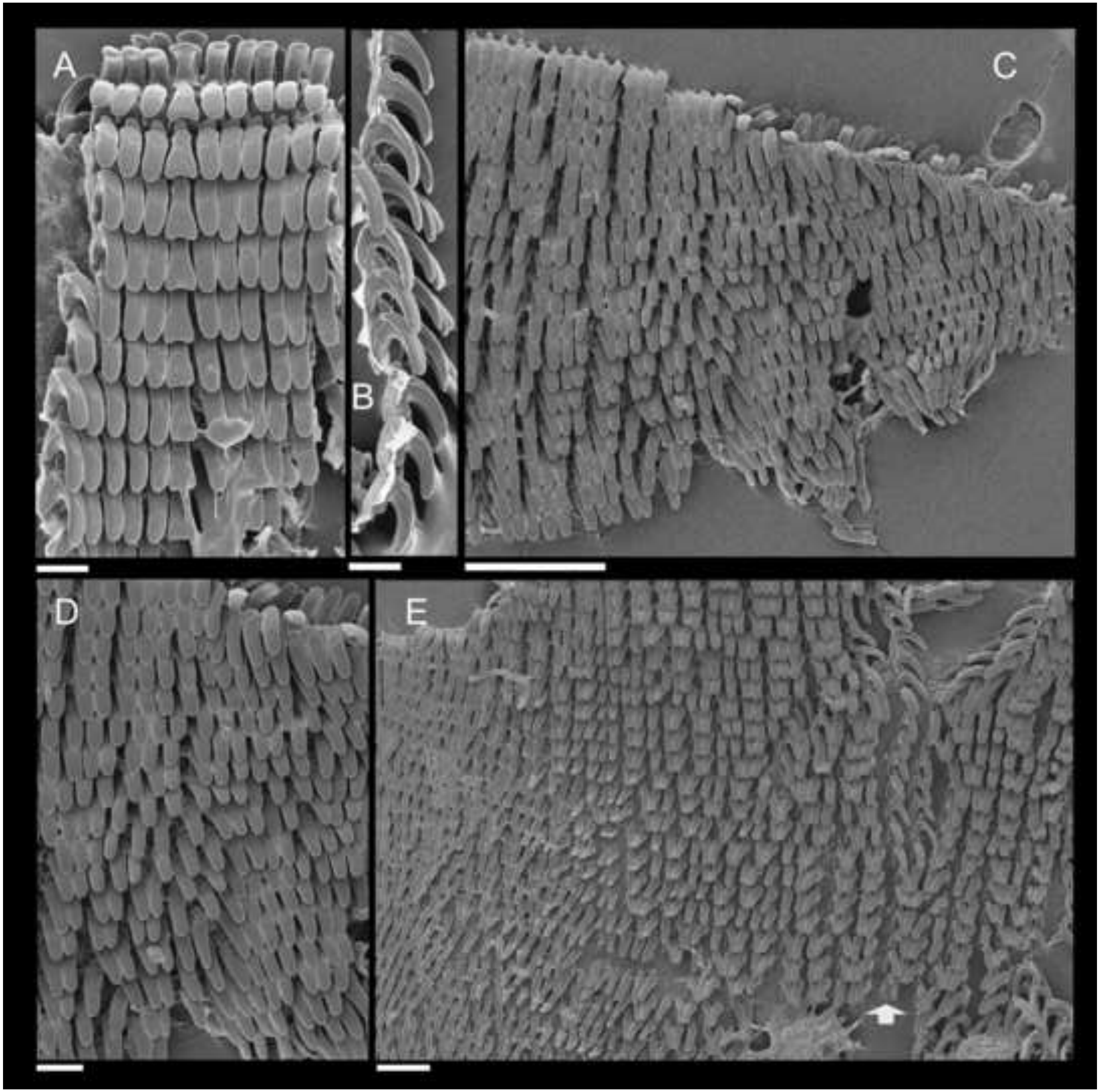

Click here to access/download;Figure;19b Anthinus savanicus.jpg

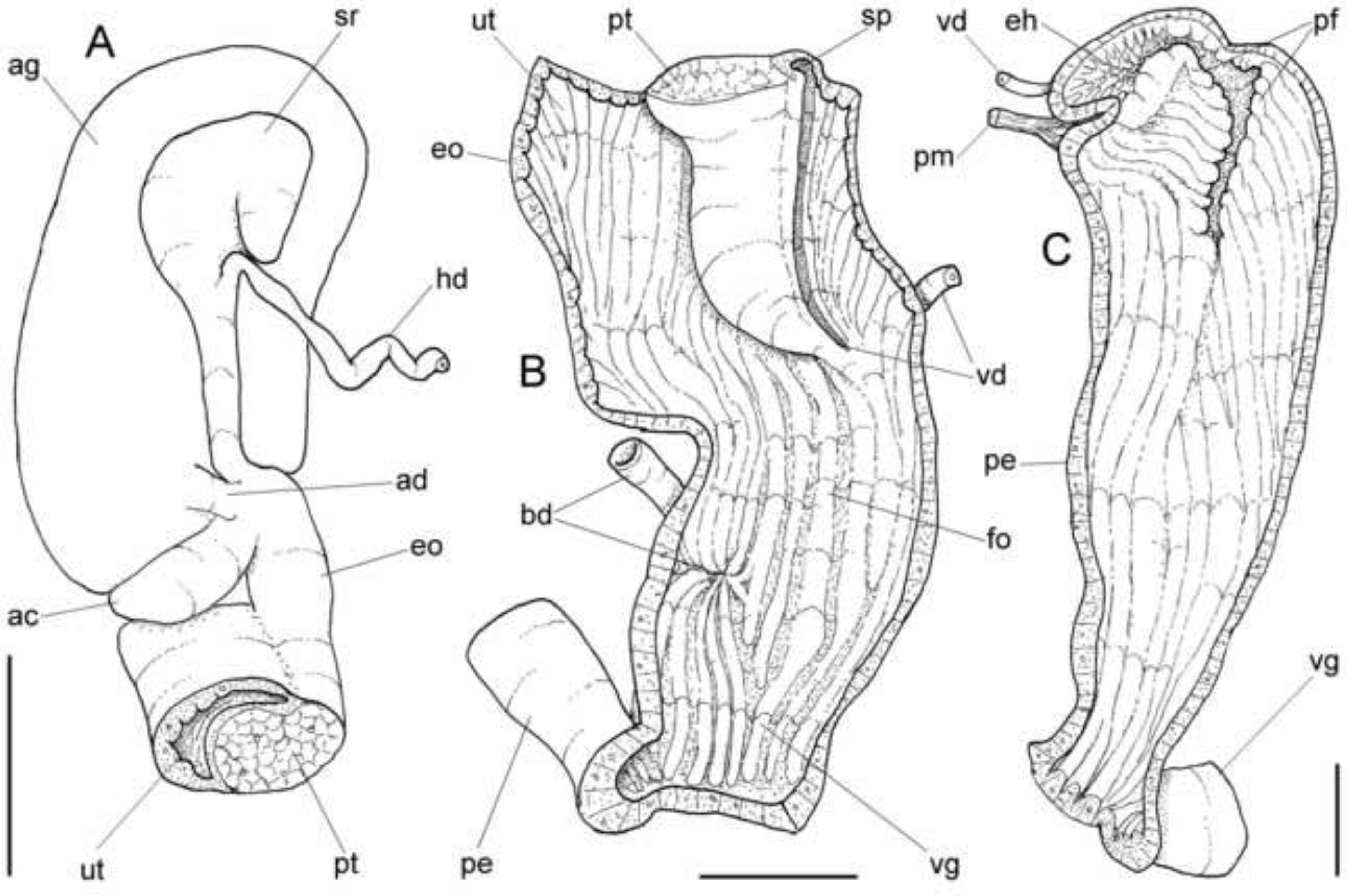

Figure 20

Click here to access/download;Figure;20b Catracca uhlei.jpg

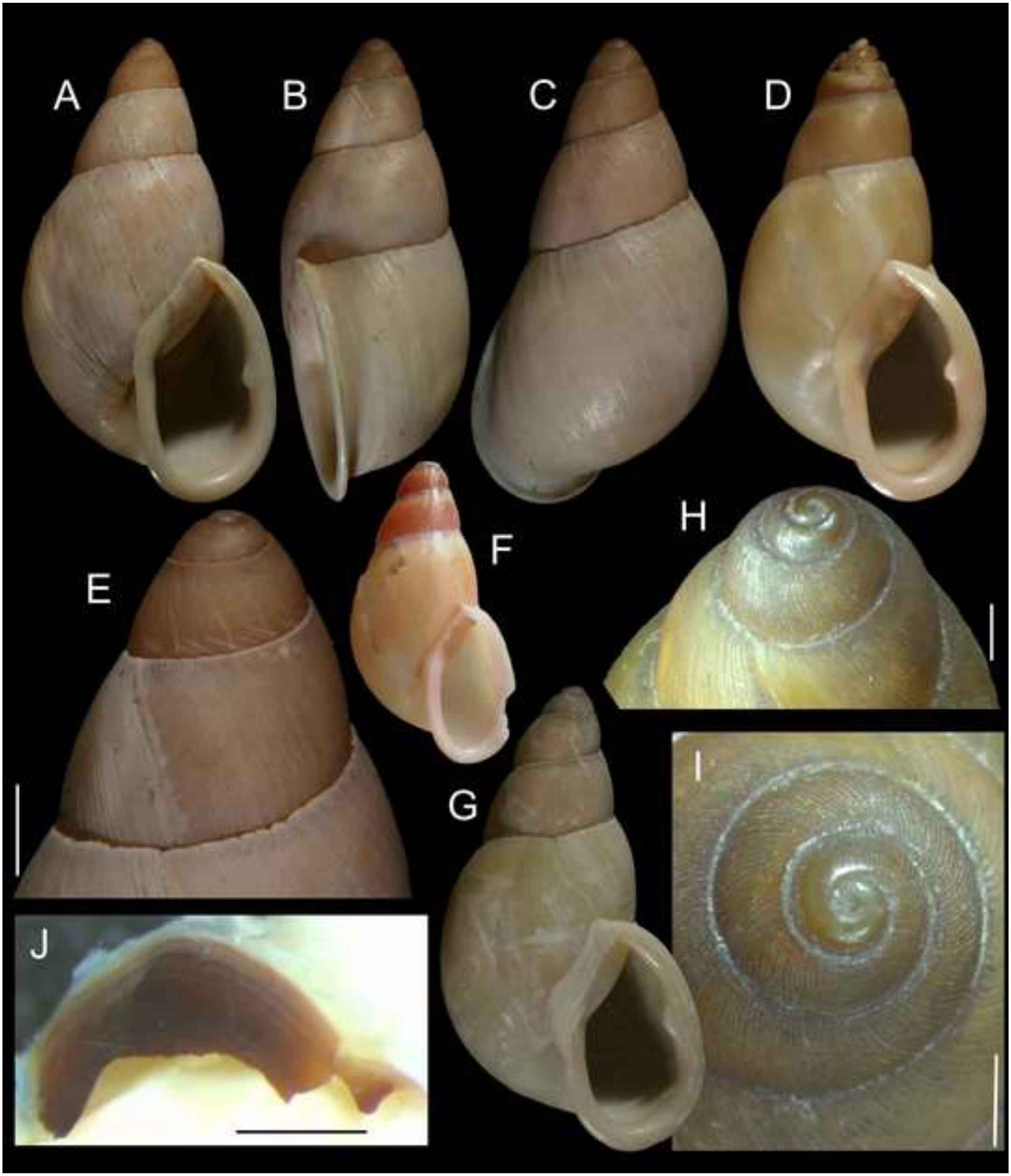

Figure 21 Click here to access/download;Figure;21b Catracca uhlei.jpg

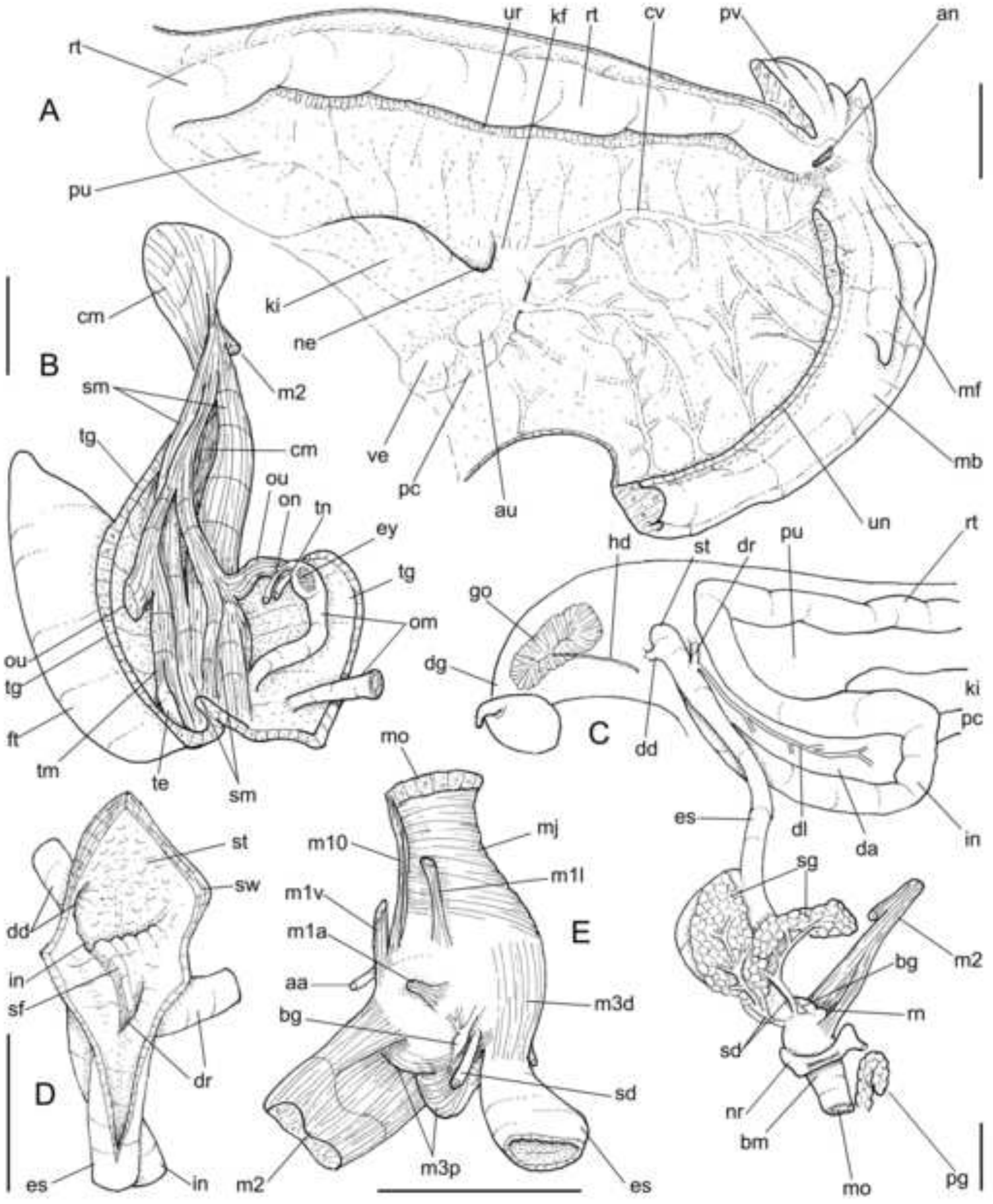

Click here to access/download;Figure;21bb Catracca uhlei.jpg

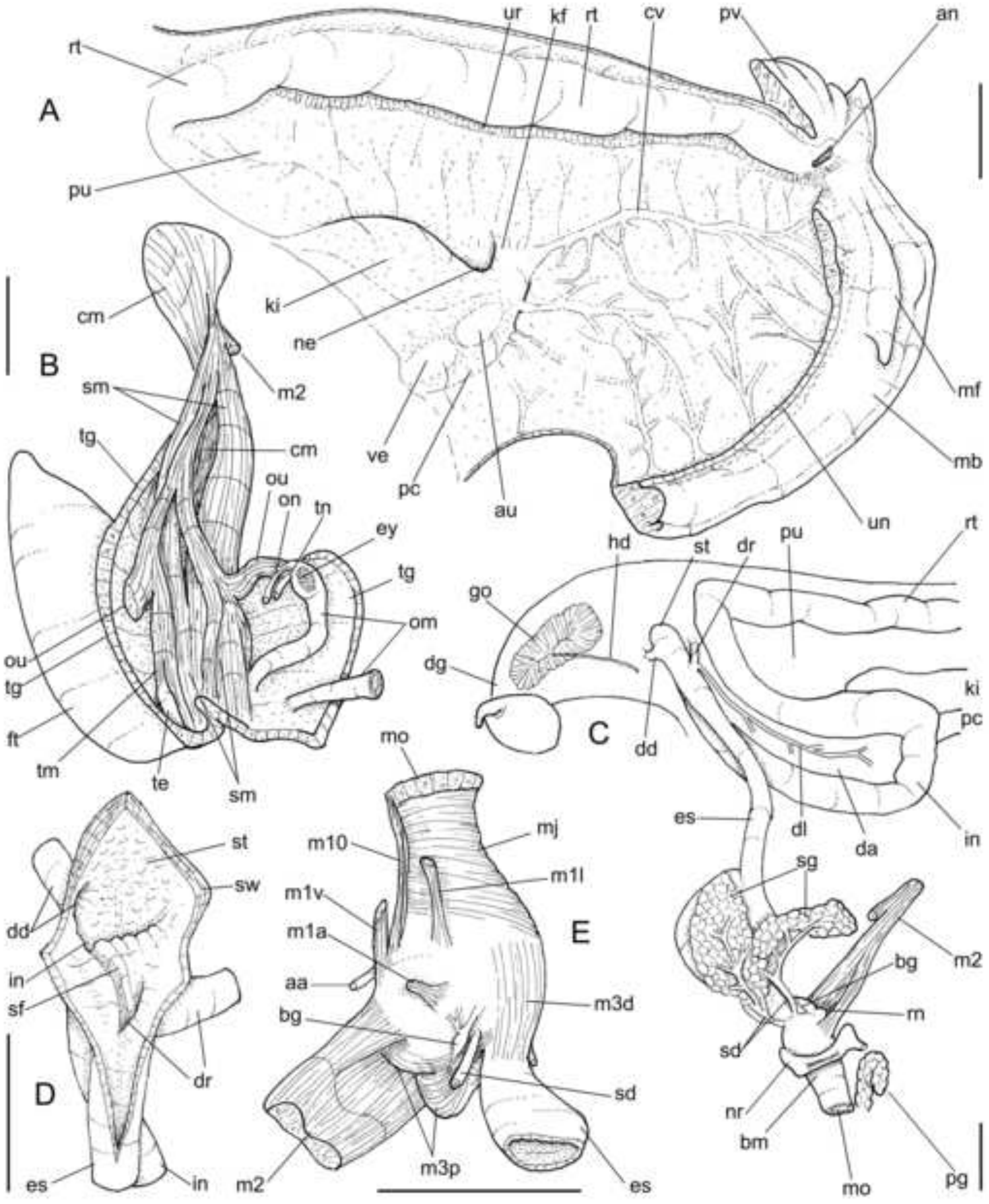

Figure 22 Click here to access/download;Figure;22b Catracca uhlei.jpg

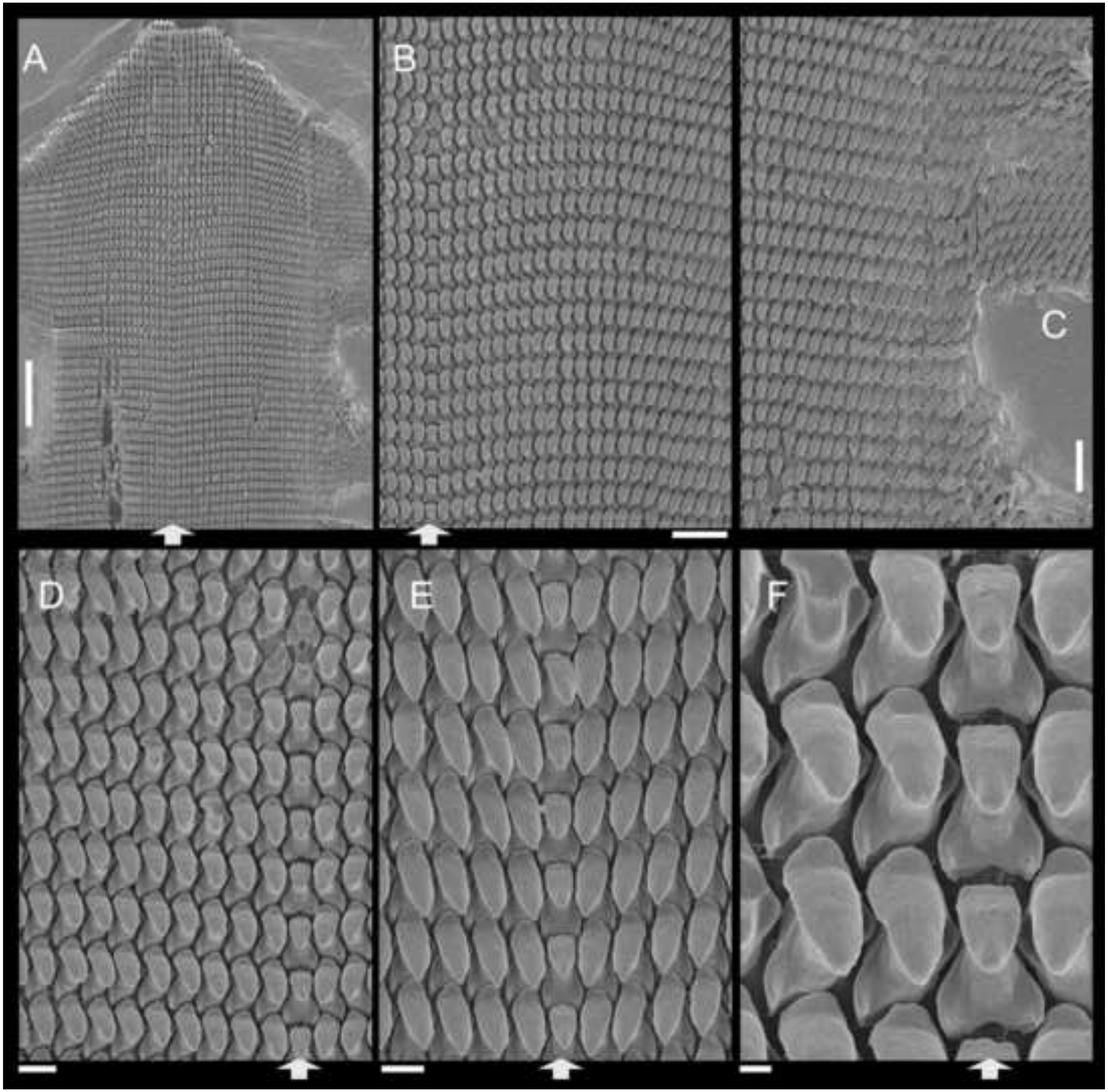

Figure 23

Click here to access/download;Figure;23b Catracca uhlei.jpg

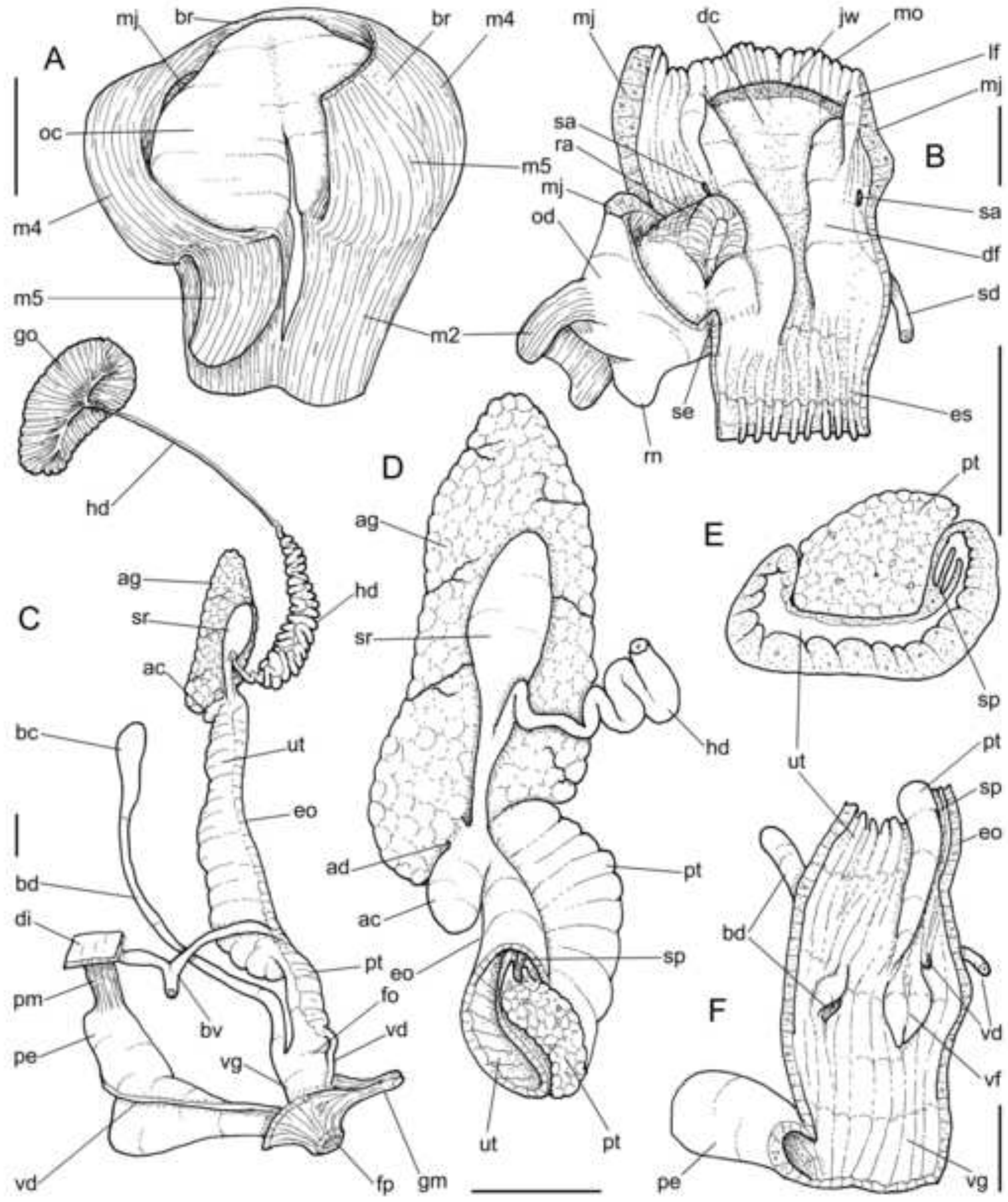

Figure 24

Click here to access/download;Figure;24b Catracca uhlei.jpg

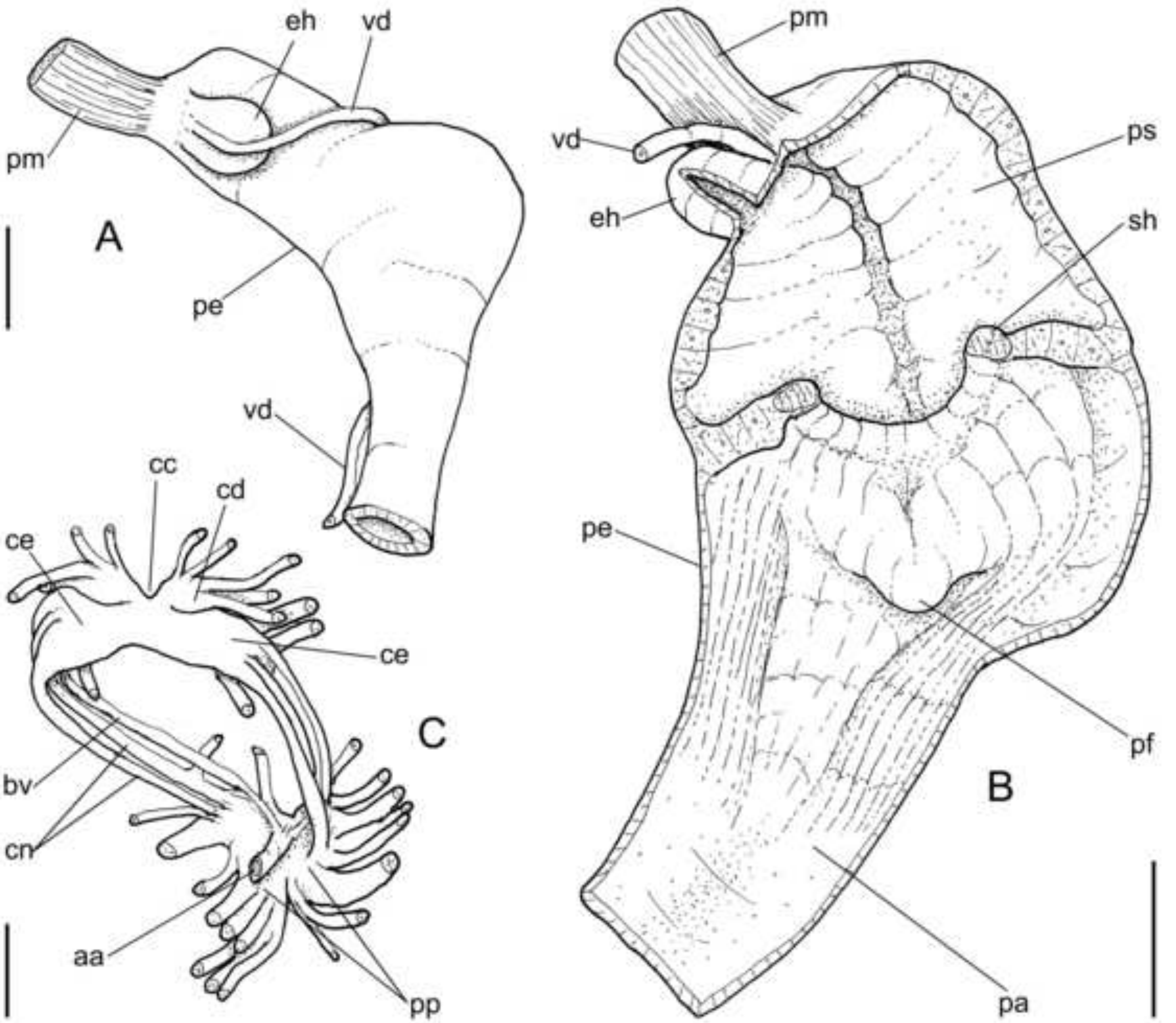

Figure 25

Click here to access/download;Figure;25b Anthinus confrontation.jpg

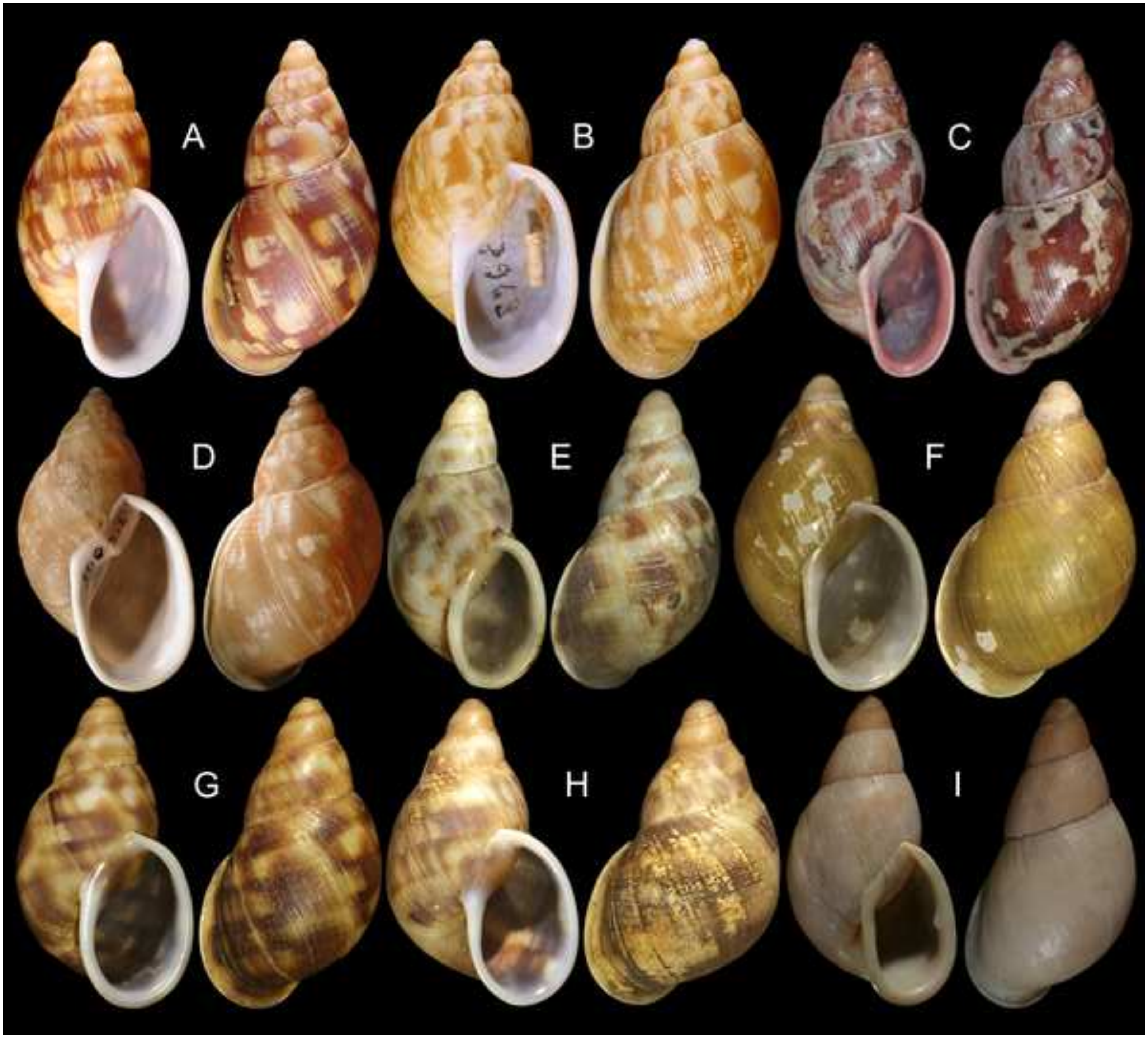

Figure 26 Click here to access/download;Figure;26b Anthinus confrontation2.jpg

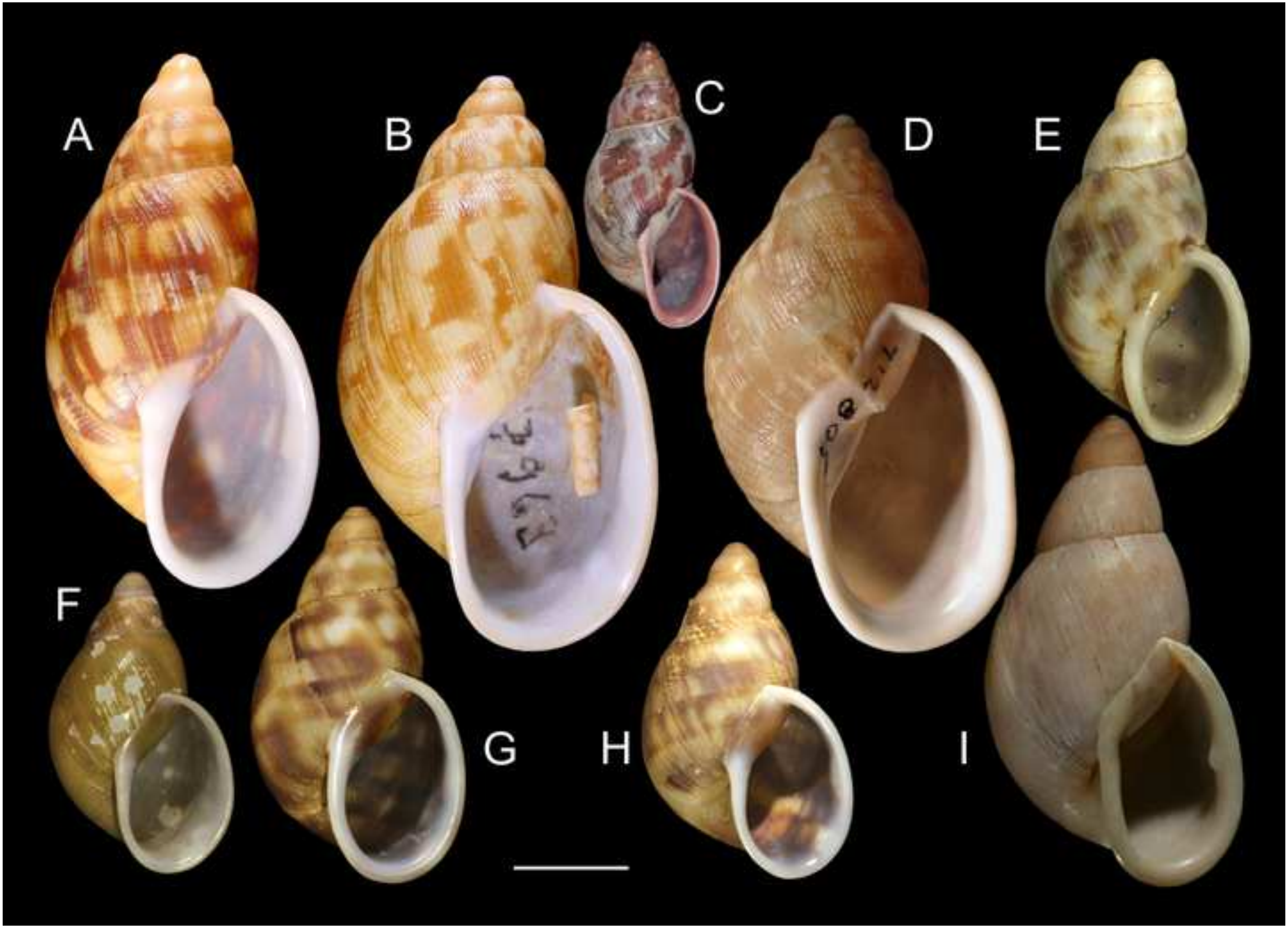

Figure 27

Click here to access/download;Figure;27b Anthinus map.jpg

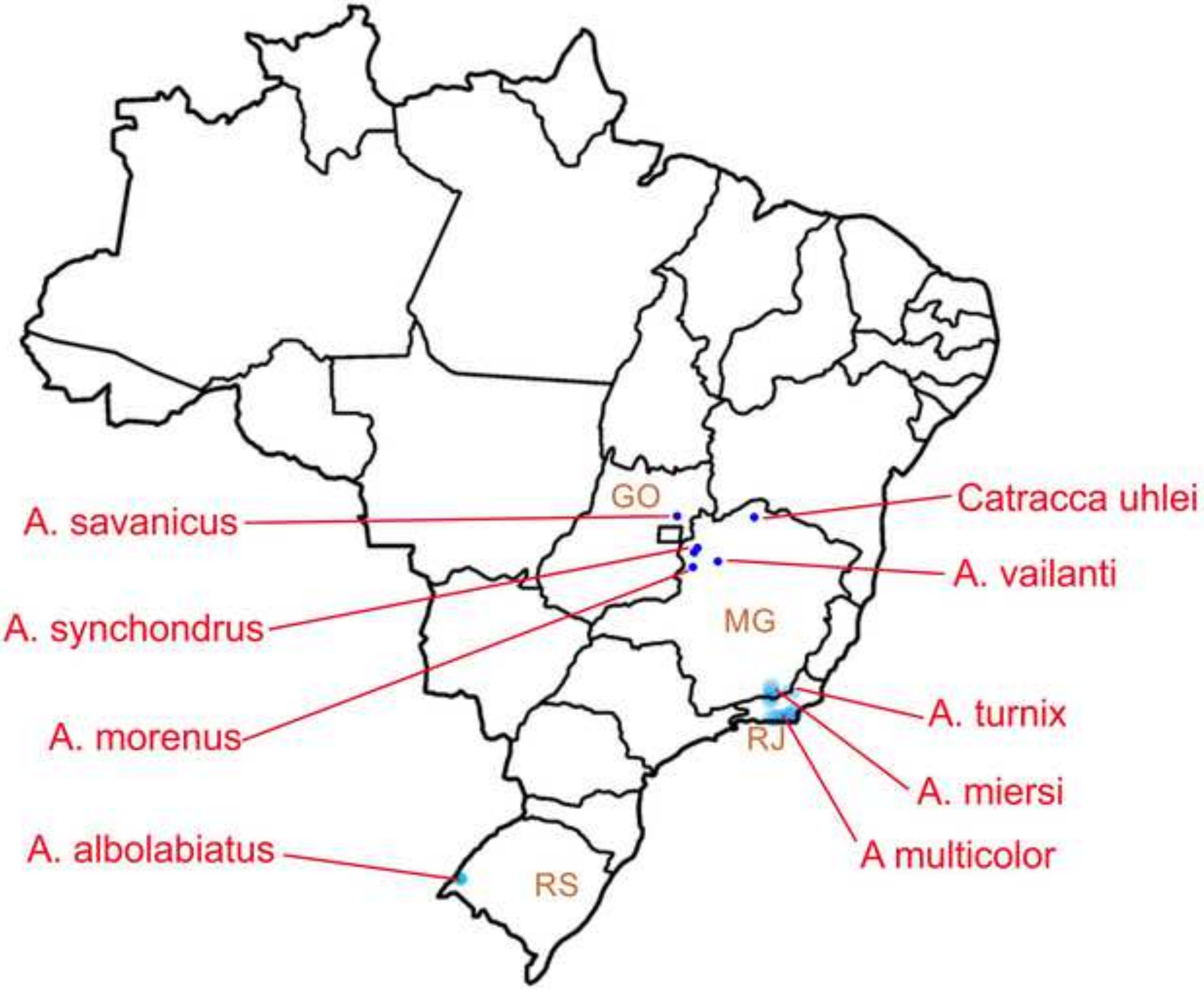

Figure 28

Click here to access/download;Figure;28b Cladogram Anthinus.jpg

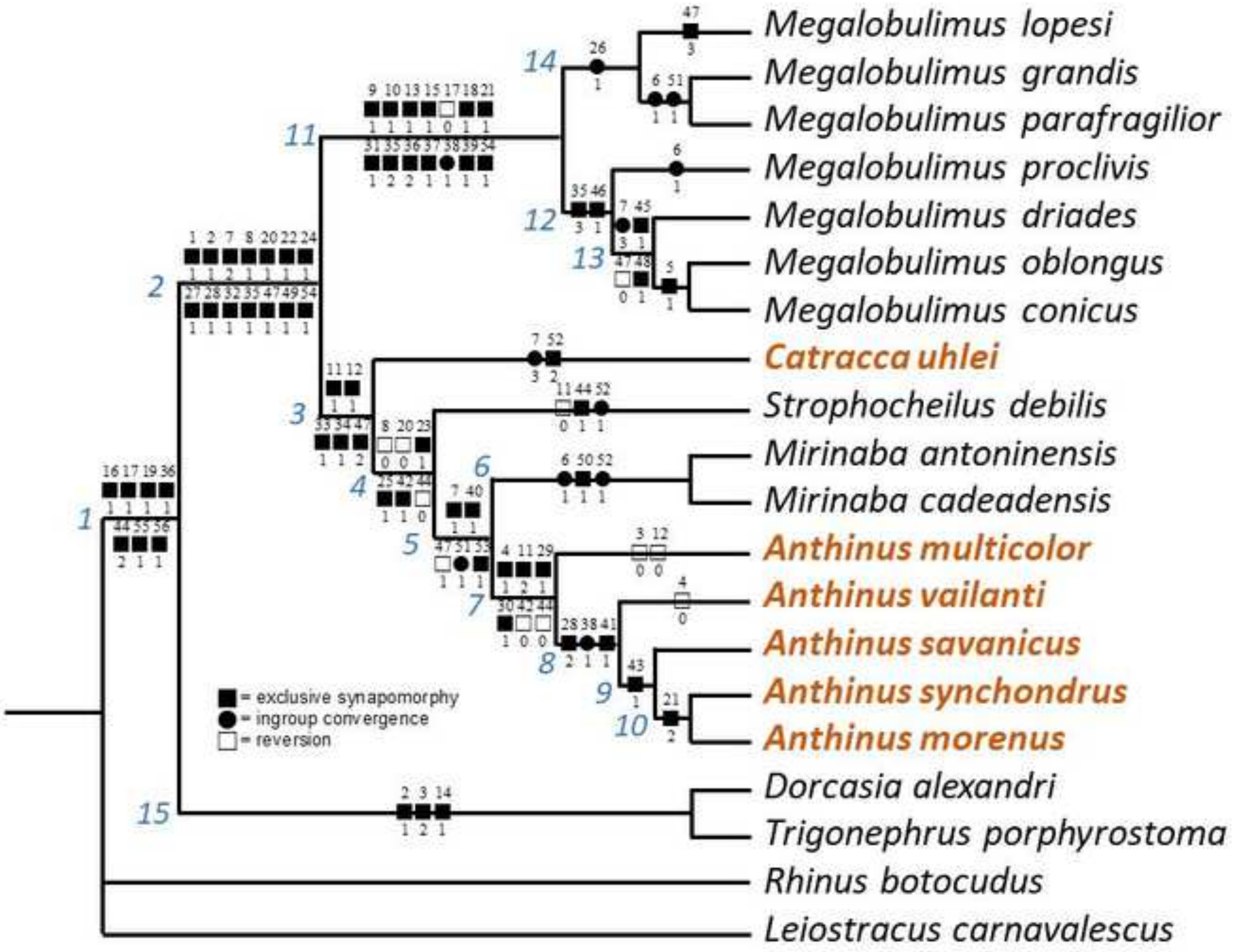

Additions to the genus *Anthinus* occurring in Minas Gerais and Goiás regions, Brazil, with description of five new species, one of them in the new related genus *Catracca* (Gastropoda, Eupulmonata, Strophocheilidae).

Luiz Ricardo L. Simone

Museu de Zoologia da Universidade de São Paulo, Cx. Postal 42391; 04218-970 São Paulo, SP, Brazil; lrsimone@usp.br; lrlsimone@gmail.com; OrcID: 0000-0002-1397-9823

Abstract

Four new species of the strophocheilid genus *Anthinus* are described from Brazil, being *A. synchondrus* from region of Unaí, Minas Gerais; *A. vailanti* from the region of Brasilândia de Minas, Minas Gerais; *A. morenus* from Paracatu region, Minas Gerais; and *A. savanicus* from Formosa region, Goiás. Another similar snail from Itacarambi, Minas Gerais, is described as a new genus and species: *Catracca uhlei*. All species are described conchologically and anatomically, with distinctions explored in almost all structures. For comparative purposes, a similar anatomical investigation on the *Anthinus* type species, *A. multicolor*, from Rio de Janeiro, is also performed. A detailed comparative analysis and discussion is provided, including taxonomicaltaxonomic and initial phylogenetic inferences. The preliminary phylogenetic analysis is based on the more detailly anatomically known species in the literature and in the presently study. A bulimulid, a simpulopsid and two dorcasiids are outgroups. The preliminary cladogram already shows a monophyletic Strophocheilidae (14 synapomorphies), divided into two also monophyletic subfamilies: Megalobuliminae (14 synapomorphies) and Strophocheilinae (5 synapomorphies). The new *Catracca* resulted as the most basal strophocheilid branch. *Mirinaba* and *Anthinus* were also resultedsupported as monophyletic (3 and 6 synapomorphies respectively. Register ZooBank: urn:lsid:zoobank.org:pub:FC4DD323-EF6A-404B-9755-F124F9DBB6D4

Introduction

The Strophocheilidae genus *Anthinus* Albers, 1850 ([1] pp:]:148) has as the type species *Helix multicolor* Rang, 1831 [2] (Subsequent Designation by Martens [3]: 189-190). In an early monographic treatment of Strophocheilidae, Bequaert ([4]: 196-202), provided detailed taxonomic history of the genus. *Anthinus* characteristically includes the smallest strophocheilids, with adult specimens with shells around 20-30 mm, usually having a tall, bluntly pointed spire, relatively small aperture in a "bulimoid-shape", and a mosaic of brown-beige spots [4, 5]. All four valid *Anthinus* species are reportedwere previously, reviewed and illustrated by Simone ([6]: 201-202). Nonetheless, the published anatomical information on *Anthinus* has remained poor, restricted to sparse information and few illustrations [5, 7]. The geographic occurrence of *Anthinus* in the southeastern Brazilian Atlantic Rainforest environment, endorsed by Simone ([6]) was from the south region of Minas Gerais-Mato Grosso do Sul in Brazil (latitude ~15°S) to northern Argentina and Uruguay (latitude ~32°S). A doubtful occurrence in Tobago has been reported ([6] and references therein; [8]).

Formatted: Font: Italic

In recent years, some samples identified as *Anthinus* have been collected in expeditions by the naturalist José Coltro Jr. and colleagues, in regions from north of Minas Gerais, to Goiás (Brazilian midwest) and in several points in northeast Brazil (latitudes ~15°S to ~17°S). This material is used in the present paper. These regions have a dryer environment, including a dryer version of the Atlantic Rainforest, Cerrado and Caatinga semi-arid biomes.

Examination of this material revealed five undescribed species, more than doubling the number of presently known *Anthinus* species. Anatomical information on the type species, *A. multicolor*, collected in Rio de Janeiro, is also included for improving the comparative scenario. The shell of one of the new species, initially appeared to belong to a unicolored *Anthinus*. It has, however, a greatly different set of anatomical features, making necessary the description of a new genus. The description of the type species is presented first, the remaining descriptions and illustrations, to minimize redundancy, are mostly presented compared to it. The published anatomical information on *Anthinus* is scanty, restricted to some sparse information and illustrations [5, 8]; it is explored in the discussion.

The family Strophocheilidae has had an unstable taxonomic history [4, 5], but usually it has been considered endemic from South America, with relatives in Africa – the Dorcasiidae; and in Asia – the Acavidae; all grouped in Acavoidea (e.g., [9]), or sometimes in Strophocheiloidea (e.g., [10]). South American members have traditionally been assigned to two families: Strophocheilidae and Megalobulimidae [5]. More recently these two families have been treated as subfamilies in Strophocheilidae, and, together with Acavidae and Dorcasiidae, placed in Rhytidoidea, which has five other families [11, 12].

This paper has as main objective the formal introduction of the new strophocheilids taxa. The anatomical base of all of theminformation presented on these new taxa will certainly be useful for future taxonomic revisions and for phylogenetic inferences. Despite these aspects are part of a large revisional project in such this study is inserted, an initial phylogenetic treatment is provided in this paper, based on the species withfor which anatomy more detaillyis reasonably well known in the literature and in the, coupled with new information on the species herein studied species.erected. The main intention is not to present a "phylogeny of the Strophocheilidae", as anatomical investigation many more species would be necessary, but as the basis for erection of the new taxa, and to provide a better scenario for a comparative discussion and the base for the new taxa.

Material and methods

Material. A complete list of material examined follows each species' description. Material comprised of dry shells or shells and specimens preserved in 70% EtOH. All listed specimens were used to compose the descriptions; those that are only shells (sh) were examined at a conchological level; all complete specimens (shell and soft parts—) (spm) were extracted and dissected.

Methods and scope. The anatomical drawings are an average of all examined specimens. Color photos were obtained by digital cameras, either hand-held (Canon Digital Rebel XTi with macro-lenz) or attached to the dissecting microscope. (Zeiss AxioCam ERc 5s). Shell measurements were obtained with a digital caliper, infor a minimum of 10 adult shells. The specimens were dissected by standard techniques [13] under dissecting stereomicroscopes, with the specimen immersed under the fixative. All drawings were obtained with the aid of a camera lucida; initially penciled, afterwards inked; usually drawings produced for each species include data derived from several specimens., as the intraspecific variation has shown

insignificant. Thus, the anatomical drawings are an average (composite) of all examined specimens. The scales were obtained putting a ruler by side of the specimen. The type and voucher material are deposited in Museu de Zoologia da Universidade de São Paulo (MZSP) malacological collection. The specimens usually are relatively easily extracted from their shells, except for the holotype of *Anthinus synchondrus*, in this case a 'cesarean' (a small window) needed to be done in the last whorl. SEM work on the material is standard, with pieces metalized by gold, and examined in the Laboratory of Electron Microscopy of the MZSP. Anatomical terminology, particularly of the odontophore muscles, follows Simone [13]. The collector material studied and described in this work was collected by a team worksworking for a Femorale, a private company called Femorale [www.femorale.com;[www.femorale.com; http://www.femorale.com/femorale/index.asp].http://www.femorale.com/femorale/index.asp]. The places of collection are not within protected areas, which doand as such collection activity did not require special permits. TheNonetheless, the collections, anyway, are supported by the were made under general/permanent license IBAMA-Sisbio 10560-2, which permits extraction of wildlife samples for scientific purposes. As most of the studied material was collected by non-scientific expeditions, no further data beyond coordinates and place names were available. Thus, details on vegetation, climate, soil, rainfall, etc., were not available, but, when possible and relevant, these data were extracted from the literature, digital online resources, or official websites. For comparison of the presently studied species with those already known, the large MZSP collection was consulted, as a previous curator,including the material assembled by Dr. José L.M. Leme, was aa previous curator and strophocheilid specialist. Collections of other European and American museums were also consulted in my trips seeking type specimens, some of them illustrated in a catalogue [6]. However, despite high effort, types of not all *Anthinus* species were found. In these cases, voucher material of previous revisions was preferentially studied. Despite having material for a total review of the genus *Anthinus* available, the previous Bequaert's review [4] is so well done that nothing actually new could be informed at shell level. Despite being relatively antique, Bequaert's review [4] is still valid in species' level, and few additional information was published on *Anthinus* after that. Thus, the present paper focuses on the description of the new taxa and the anatomical ineditedunpublished features. The type species, however, is included, extending to anatomical features and improving the comparative taxonomic scenario. Although additional approaches can be added to any taxonomical paper, such as multivariate statistical analyses of shell measurements, molecular sequencing, etc., the presently presented anatomical investigation was so informative and convincing that it already bases very well the taxonomic conclusions. Of course, other approaches, molecular in particular, will be explored in future papers, considering an already well-based taxa provided by the present paper. It is important to emphasize that most of the land snails taxonomy is based only on their shells alone; the present paper goes further and presents convincing anatomical descriptions. The chosen model to do the taxonomic remarks on each described species is reuniting all of them in a single Discussion, which ends this paper. This is interpreted as a more intelligible, fluent, and economic manner to explore the distinctions and similarities of the addressed taxa. Rather than individual remarks after each descriptionThe taxonomical remarks on each species are all reunited in the Discussion item.

Phylogenetic analysis. The phylogenetic methodology is the same as reported by Simone [13, 23], that basically consists of the matrix mounted in Nexus, analyzed by programs TNT and PAUP. All of them resulted in a single cladogram shown below. Thus, beyond the species herein studied, the present preliminary phylogeny is based upon already published data of 14 species listed below, as well as additional examination of their voucher material deposited in MZSP in some unstudied structures, such as, e.g., the odontophore. The list of characters is in Appendix 1; and respective matrix in Appendix 2. It is important to emphasize that the shown phylogeny is not to be interpreted as "the phylogeny of the Strophocheilid". It has only the

Formatted: Font: Italic

intention of demonstrating that the description of the new genus – *Catracca* – is necessary. In the literature**Phylogenetic analysis.** In a family such as Strophocheilidae, that bears almost a hundred of known species, a phylogenetic study is only worthy if it includes a large number of representatives. This is presently not possible, as few species are known beyond their shells; and shells alone cannot base a well-resolved phylogeny [13]. Anyway, a small set of strophocheilids has their anatomy known in sufficient details for an initial phylogenetic inference. They are (1) *Mirinaba antoninensis* (Morretes, 1952) [14, 15]; (2) *Mi. cadeadensis* (Morretes, 1952) [15]; (3) *Strophocheilus debilis* Bequaert, 1948 [5]; (4) *Megalobulimus lopesi* Leme, 1984 [16]; (5) *Me. grandis* (Martens, 1885) [16]; (6) *Me. parafragilior* Leme & Indrusiak, 1990 [17]; (7) *Me. proclivis* (Martens, 1888) [18]; (8) *Me. dryades* Fontenelle, Simone & Cavallari, 2021 [19]; (9) *Me. oblongus* (Müller, 1774) and (10) *Me. conicus* (Bequaert, 1948) [20]. As remote outgroups, a bulimulid (*Leiostracus carnavalescus* Simone & Salvador, 2016 [21]) and a simpulopsid (*Rhinus botocudus* Simone & Salvador, 2016 [21]), and close outgroups a pair of African dorcasiids (*Dorcasia alexandri* Gray, 1838 and *Trigonephrus porphyrostoma* (Melvill & Ponsonby, 1891) [22]) were chosen. All six species studied herein were also included. The phylogenetic methodology is the same as reported by Simone [13, 23], that basically consists of the matrix mounted in Nexus, analyzed by programs TNT and PAUP. All of them resulted in a single cladogram shown below. Thus, beyond the species herein studied, the present preliminary phylogeny is based upon already published data of the above mentioned 14 species, as well as additional examination of their voucher material deposited in MZSP in some unstudied structures, such as, e.g., the odontophore. The list of characters is in Appendix 1; and respective matrix in Appendix 2. It is important to emphasize that the shown phylogeny is not to be interpreted as "the phylogeny of the Strophocheilid". It has only the intention of demonstrating that the description of the new genus – *Catracca* – is necessary.

Nomenclatural acts. The electronic edition of this article conforms to the requirements of the amended International Code of Zoological Nomenclature, and hence the new names contained herein are available under that Code from the electronic edition of this article. This published work and the nomenclatural acts it contains have been registered in ZooBank, the online registration system for the ICZN. The ZooBank LSIDs (Life Science Identifiers) can be resolved and the associated information viewed through any standard web browser by appending the LSID to the prefix "http://zoobank.org/". The LSID for this publication is: urn:lsid:zoobank.org:pub:FC4DD323-EF6A-404B-9755-F124F9DBB6D4. The electronic edition of this work was published in a journal with an ISSN, and has been archived and is available from the following digital repositories: PubMed Central, LOCKSS, ResearchGate.

Formatted: Font: Not Bold

It is important to state that funders had no role in study design, data collection and analysis, decision to publish, or preparation of the manuscript. Addicionally, the author received no specific funding for this work.

Abbreviations in the Figures: **aa**, anterior aorta; **ac**, albumen chamber; **ad**, albumen gland duct; **af**, anal folded subterminal portion; **ag**, albumen gland; **an**, anus; **as**, accessory genital gland; **au**, auricle; **bc**, bursa copulatrix; **bd**, bursa copulatrix duct; **bg**, buccal ganglion; **bm**, buccal mass; **br**, subradular membrane; **bv**, blood vessel; **cc,** cerebral commissure; **ce**, cerebral ganglion; **cd**, cerebral node; **cn**, cerebro-pedal and cerebro-pleural connectives; **cv**, pulmonary (efferent) vein; **da**, digestive gland anterior lobe; **dc**, dorsal chamber of buccal mass; **dd**, gastric duct to digestive gland; **df**, dorsal folds of buccal mass; **dg**, digestive gland posterior lobe; **di**, diaphragm or pallial floor; **dl**, left esophageal duct to digestive gland; **dr**, right esophageal duct to digestive gland; **eh**, epiphallus; **eo**, spermoviduct; **es**, esophagus; **ey**, eye; **fo**, free oviduct; **fp**, genital pore; **ft**, foot; **gm**, genital muscle; **go**, gonad; **hd**, hermaphrodite duct; **hp**, hump of free oviduct; **in**, intestine; **jw**, jaw; **kf**, kidney anterior fold; **ki**, kidney; **lf**, lateral fold of buccal mass; **m1–m10**, extrinsic and intrinsic odontophore muscles; **mb**, mantle border (edge); **mf**, mantle

fold; **mj**, jaw and peribuccal muscles; **mo**, mouth; **mp**, prerectal muscle; **ne**, nephrostome; **nr**, nerve ring; **oc**, odontophore cartilage; **od**, odontophore; **om**, ommatophore; **on**, optical nerve; **ou**, ommatophore muscle; **pa**, penis anterior chamber; **pc**, pericardium; **pe**, penis; **pf**, penis fold; **pg** pedal gland; **pm**, penis muscle; **pn**, pneumostome; **pp**, pedal ganglion; **pr**, penis aperture; **ps**, penis posterior chamber; **pt**, prostate; **pu**, pulmonary cavity; **pv**, pneumostome inner flap; **ra**, radula; **rn**, radular nucleus; **rs**, radular sac; **rt**, rectum; **sa**, salivary gland aperture; **sc**, subradular cartilage; **sd**, salivary gland duct; **se**, septum between esophagus and odontophore; **sf**, stomach inner fold; **sg**, salivary gland; **sh**, penis central sphincter; **sm**, secondary cephalic muscles; **sp**, sperm inner longitudinal fold; **sr**, seminal receptacle; **st**, stomach; **sw**, stomach muscular walls; **te**, ventral cephalic tentacle; **tg**, integument; **te**, tentacle; **tm**, tentacle muscle; **tn**, tentacular nerve; **un**, union of mantle border with nuchal surface; **ur**, urinary gutter; **ut**, uterus; **vd**, vas deferens; **ve**, ventricle; **vf**, vaginal fold; **vg**, vagina.

Additionally in the text, the following abbreviations are used: **L**, length; **sh**, empty dry shell; **spm**, complete specimen (shell and soft parts); **W**, width. Institutions: **MNRJ**: Museu Nacional da Universidade Federal do Rio de Janeiro; **MZSP**: Museu de Zoologia da Universidade de São Paulo; **USNM**: National Museum of Natural History, Smithsonian Institution.

Results

Comparative conchology and anatomy

Systematics

Genus *Anthinus* Albers, 1850

Bulimus (*Anthinus*) Albers, 1850[1]: 148; Martens, 1860[3]: 189.

Auris (*Anthinus*): Pilsbry, 1895[24]: 95.

Gonyostomus (*Anthinus*): Bequaert, 1948[4]: 196; Oliveira et al., 1968[8]: 10.

Anthinus: Morretes, 1949[25]: 145; Leme, 1973[5]: 332; Simone, 2006[6]: 201.

Diagnosis. Shell of ~30 mm, apex blunt, aperture ~half of shell length. Protoconch of 3-4 whorls, sculptured by delicate reticulation of spiral and axial lines. Color mosaic of brown, beige and cream wide spots, with tendency of spiral organization in squared irregular spots. Head-foot lacking labial flange. Lung with not-protruded vessels, dark pigmented. Odontophore pair m5 ~half originated from cartilages and ~half from m4, free from m2. Pair of anterior (esophageal) ducts to digestive gland, located posteriorly, practically from stomach; both components (dr, dl) with about same caliber, left duct much longer. Prerectal pallial muscle (mp) usually present. Anus turned externally in pneumostome right corner. Seminal receptacle strongly curved. Spermoviduct accessory gland (as) present. Penis with single inner chamber; epiphallus short, rounded, vas deferens insertion subterminal, inner surface papillate; inner main chamber with main pair of strong longitudinal folds, sulcus between these folds continuous to epiphallus. Other details in [4, 5].

List of included species. *Anthinus multicolor* (Rang, 1831) (type species); *A. albolabiatus* (Jaeckel, 1927); *A. miersi* (Sowerby, 1838); *A. turnix* (Gould, 1846); *A. synchondrus* new species; *A. vailanti* new species; *A. morenus* new species; *A. savanicus* new species.

Taxonomic discussion. see Discussion item.

Anthinus multicolor (Rang, 1831) Figs. 1-4

Previous synonymy see Bequaert (1948[4]: 196-197). Complement:

Helix (*Cochlogena*) *multicolor* Rang, 1831[2]: 55 (pl. 3, fig. 1).

Gonyostomus (*Anthinus*) *multicolor:* Bequaert, 1948[4]: 196-198 (pl. 4, fig. 5; pl. 9, figs 3, 7; pl. 13, fig. 6; Pl. 17, fig. 4).

Anthinus multicolor: Morretes, 1949[25]: 145; Leme, 1973[5]: 332; Abbott, 1989[26]: 76 (fig.); Salgado & Coelho, 2003[27]: 156; Simone, 2006[6]: 202 (fig. 760).

Gonyostomus multicolor: Oliveira et al., 1968[8]: 10; 1981[28]: 341.

Types. whereabouts unknown [4].

Type locality. São Paulo State and near Corcovado, Rio de Janeiro.

Diagnosis. Shell about 35 mm; color usual mosaic of brown and beige spots, lacking spiral alignment; spire angle ~40°, protoconch of 2.5 whorls; peristome ~52% of shell length and ~55% of width; width ~46% of length. Mantle edge with simple, rounded fold. Reno-pericardial area small-sized. Pre-rectal pallial muscle absent. Odontophore with cartilages ~90% fused with each other; pair m4 ~50% originated from cartilage, remaining on m4. Jaw plate broad, thick. Radula with short, hook-like mesocone, ~~rachidial~~rachidian different from neighboring teeth. Seminal receptacle widely curved, hermaphrodite duct inserting in its base, curved. Accessory genital gland in spermoviduct small; with pair of similar-shaped sperm-grooves. Free oviduct with distal hump. Penis length ~50% of spermoviduct; penis muscle mostly inserted to penis' tip, with small branch to epiphallus and another small branch to adjacent vas deferens; epiphallus papillae tall, uneven, extending along entire penis chamber; large pair of penial inner folds wide and low, covered by papillae.

Redescription

Shell. (Figs. 1A-E) proper description in [4]. Complement: Adult shell around 35 mm, conical-oval; apex blunt; wider on last whorl; width ~46% shell length. Walls relatively thin. Color sometimes entirely brown ([6]: 202), but usually with reddish beige spiral band along inferior half of each spire whorl, or central region of body whorl (Figs. 1A-C); white spots randomly splayed, overlapping remaining colors, usually coalescent near superior suture, and also inferior third of last whorl (Figs. 1B-C), barely forming irregular axial bands; small white punctiform spots splayed in inferior half of spire whorls and middle region of body whorl. Spire angle ~40°. Protoconch of 2.5 whorls (Figs. 1D-E), ~12% of length, width of ~6.1 mm, slightly taller than wide; first whorl smooth, gradually spiral narrow aligned punctuations appearing (Figs. 1D-E), lacking axial cords except for relatively regular axial undulations, ~3 per mm, last protoconch whorl with spiral sculpture more spaced, ~8 in last protoconch whorl. Teleoconch of ~2 whorls, profile slightly convex; sculpture similar to that of protoconch last whorl, spiral punctuations (10 in penultimate whorl) and axial undulations; last 1.5 whorls lacking spiral punctuations, only having smooth growth lines (Fig. 1C). Whorls profile weakly convex (Figs. 1A-C). Suture well-marked, not channeled (Fig. 1C). Aperture slightly prosocline (~10° from longitudinal general axis) (Fig. 1B), elliptic; ~52% of shell length, ~55% of shell width. Peristome continuous, reflected, thick, color red (Fig. 1A); outer lip arched, lacking middle tooth; inner lip with inferior half almost straight, superior half as broad, weak callus with ~2/5 of peristome length (Fig. 1A),

weakly convex. Inferior half of inner lip covering umbilicus. Umbilicus relatively wide, ~10% of inferior area. Body whorl ~70% shell length; uniform with spire.

Head-foot. (Fig. 2B) of normal shape. Color uniformly pale. Columellar muscle thick, 1.5 whorls in length. Inner arrangement of columellar annexed muscles relatively complex. Main columellar bundle (cm) occupying ventral floor of haemocoel, relatively flat, wide slightly broader than half of foot width. Pair of secondary cephalic muscles (sm) with middle and anterior regions approximately double thickness and half width those of columellar main bundle (left muscle slightly broader than right one); originating gradually along dorsal surface of main columellar bundle, running anteriorly on main bundle; anterior third of each muscle with four parallel branches, two medial branches broad (~2/3 of its basal width) inserting in ventral region of perioral region. Branches 3 and 4 respectively tentacular and ommatophore muscles; ommatophore muscles (ou) narrower, mode lateral, inserting in tip of ommatophore; tentacular muscle (tm) longer, inserted in tip of tentacles. Pedal gland (pg) protruding in posterior region of buccal area.

Mantle organs. (Fig. 2A) Mantle border thick, lacking pigments. Pneumostome (pn) protected by simple right ventral flap (pv), width ~1/5 of aperture length. Dorsal fold well developed (mf), occupying ~1/3 of dorsal mantle edge length; rounded. Pneumostome (pn) ~1/10 of shell aperture length, bearing exclusively air entrance and urinary gutter. Anus (an) separate aperture located at right, adjacent to pneumostome. Lung of 1.2 whorls in length, wide and elongated; right side ~1.5 times longer than left side. Pulmonary venation well-developed; posterior region of pulmonary vein (cv) protruded; left half only having longitudinal vessels, right half mostly having perpendicular vessels, except for oblique vessels in region preceding pneumostome. Pulmonary vein (cv) running longitudinally across pallial cavity roof medially or towards right, anterior end trifurcated. Reno-pericardial area beige, slightly triangular, located posteriorly within pallial cavity, its posterior abutting wall of visceral cavity, occupying ~10% of cavity length and ~40% of its width (details below). Rectum (rt) wide. Urinary gutter (ur) broad, smooth, lacking clear transverse folds; running along rectum; anterior urinary gutter surrounding left anal aperture.

Visceral mass. (Fig. 2A) ~3 whorls in length. Both digestive gland lobes brown in color; anterior lobe (da) flattened, occupying ~1/5 of visceral volume, located just posteriorly to pallial cavity, continuous to kidney. Posterior lobe (dg) larger, extending 2 spiral whorls, occupying ~60% of visceral volume. Stomach ~1/15 of visceral volume, located between both digestive gland lobes, about 3/4 whorl posterior to pallial cavity (st). Digestive tubes (described below) surrounding anterior lobe of digestive gland. Gonad multi-lobed, cream color, encased between posterior lobe of digestive gland and columella, occupying ~1/3 whorl, ~1/10 of visceral volume.

Circulatory and excretory systems. (Fig. A) Pericardium (pc) ~twice as long as wide, located obliquely between middle and left thirds of posterior end of pallial roof, appressed against right lateral side of kidney; occupying ~5% of lung area. Auricle located anteriorly, as continuation from pulmonary vein (cv); ventricle located posteriorly, larger. Kidney (ki) simple, dorso-ventrally flattened; size reported above; somewhat triangular, width ~2/3 of length; internally organized as two distinct regions – a longitudinal hollow cavity on left; filled by sponge-like renal tissue, clearly U-shaped (convexity left) in section. Nephropore (ne) small, longitudinal slit at anterior-left corner of kidney, directed towards right; protected by curved anterior projection of kidney (kf). Urinary gutter (ur) reported above.

Digestive system. (Figs. 2A) Mouth (mo) and oral tube wide, muscular. Jaw plate (Figs. 1G-H) thick; cutting edge blunt, sigmoid; lacking inner reinforcement, immersed in jaw thickness. Buccal mass spherical, occupying~1/5 of haemocoel volume. Dorsal surface of oral cavity with well-developed pair of dorsal folds, width of each ~1/3 of dorsal wall width; separated from each other by dorsal chamber (dc) as wide as folds, but broader anteriorly. Odontophore (od) with

~60% of buccal mass volume. Odontophore muscles: **mj**, jaw and peribuccal muscles originating in outer-ventral surface of odontophore cartilages, running towards dorsal, splaying in dorsal wall of oral tube; **m1**, jugal muscles covering entirely haemocoelic structures, more concentrated close to mouth; **m1v**, small pair of ventral protractors jugal muscles, originating in ventral surface of haemocoel close to mouth, running towards posterior, inserting in ventral-posterior region of odontophore close to m2 insertion; **m2**, strong pair of retractor muscles of buccal mass, or radular muscles, originating as single bundle in columellar muscle posterior end, running anteriorly close to median line along ~60% of haemocoel length, inserting as two different bundles in ventro-posterior edge of odontophore, surrounding at some distance radular nucleus; **m3d**, thin layer of longitudinal fibers immersed in dorso-posterior wall of odontophore, preceding esophageal origin; **m4**, main pair of dorsal tensor muscles of radula, very thick, originating in postero-medial region of odontophore cartilages, surrounding outside and medially cartilages, inserting in subradular membrane in its region correspondent to buccal cavity; **m5**, pair of thick auxiliary dorsal tensor muscles of radula, originating half on posterior-medial surface of m4, and half on portion in postero-ventral region of odontophore cartilages, running towards median line covering m4, inserting in subradular membrane by side of m4 insertion; **m6**, horizontal muscle absent; **m7**, absent; **m10**, pair of narrow ventral odontophore protractor muscles, originating in ventro-anterior region of haemocoel, just ventral to mouth, running towards posterior along buccal mass length, inserting in latero-posterior surface of odontophore close to m2 insertions; **m11**, pair of narrow ventral tensor muscles of radula absent. Odontophore non-muscular structures: oc, pair of odontophore cartilages flattened, rather elliptical, anterior region slightly projected anteriorly close to median line, ~1.2 times longer than wide, fused with each other along ~90% in their anterior-medial edge, posterior end roughly rounded; **sc**, subradular cartilage, with expanding region in buccal cavity protecting subradular membrane. Radular sac short, not extending beyond odontophore.

Radula. (Fig. 3) Slightly longer than odontophore; with rachidian teeth, and ~30 pairs of lateral/marginal teeth; no clear distinction between lateral and marginal teeth (Fig. 3A), marginal teeth only slightly narrower than lateral teeth, and more inclined medially (Figs. 3A, B: left side); all teeth with base as long as mesocone, articulating neighboring rows (Figs. 3C); mesocone arched, curved inwards and posterior, ~1.5 longer than wide, apex rounded, base reinforcement slightly more developed, subterminal; no secondary cusps. **Rachidian** tooth (Figs 3B, C: arrow) as large as lateral teeth; base ~twice longer than wide, slightly flattened, barely triangular, with arched posterior edge articulating with neighbor tooth and pair of distal reinforcements; mesocone reduced, ~half size of those of lateral teeth, rounded, covering ~1/3 of base; width and length similar to those of lateral teeth. **Lateral teeth** similar to rachidian, except in being slightly longer, longer asymmetrical mesocone, arched towards medial region; base trapezoid, with medial concavity articulating with neighbor medial tooth and single distal reinforcement (Fig. 3C). **Marginal teeth** starting with no clear boundary with lateral teeth (Figs. 3A, B), occupying ~1/3 of each side; shaped similarly to lateral teeth, except for being slightly narrower and more inclined medially, row practically straight (Fig. 3A: left side).

Salivary glands small, covering ~1/5 of esophagus length, in its anterior quarter region (Fig. 2A: sg), forming two rounded, white, thin masses. Each salivary duct differentiable in middle and anterior side of glands, with ~1/12 of esophageal width (sd). Salivary duct running in both sides of esophageal origin, penetrating buccal mass wall in region close to buccal ganglia, running immersed in buccal dorsal wall along ~1/3 its length. Salivary ducts opening as small slits in located in posterior region of lateral edges of wide dorsal folds.

Esophagus ~1-whorl long, with thin, flaccid walls (Fig. 2A: es); anterior 1/2 weakly broader, crop-like; inner surface simple, smooth. Stomach (st) narrow, curved, weakly bulging; position and size described above (visceral mass); gastric walls thin, weakly muscular; inner surface

mostly smooth, but with chitinous inner cover well-developed. Esophageal insertion on right side, intestinal origin on left side, both close to columella. Duct to anterior lobe of digestive gland at short distance from esophagus and intestine intersection (dr) narrow, running shortly towards right, bifurcating; additional left duct (dl) originated in main duct base with ~1/3 of its width, running narrowly towards left and anterior along digestive gland lobe compressed by first intestinal loop (da), periodically possessing short ramifications along its length. Duct to posterior lobe of digestive gland located short distance from intestinal origin, posterior to above-described duct, directed towards opposite side (dd) as wide as anterior duct. Intestine (in) initially as wide as esophageal insertion, gradually narrowing up to ~half that width along its wide sigmoid loop in anterior lobe of digestive gland (in). Rectum and anus position described above (pallial cavity) (rt, an). Anus sessile, as slit in right end of mantle edge directly turned outside; inner surface with 8–10 simple longitudinal, relatively tall folds.

Reproductive system. (Fig. 2C, 4) Gonad position described above (visceral mass), composed of 8–10 lobes with minute digitiform acini. Hermaphroditic duct (Fig. 2C: hd) broad; coiled portions occupying anterior 2/3, with very strong coils; insertion strongly curved (Figs. 2C, 4B: hd). Seminal receptacle (Figs. 2C, 4B: sr) relatively large, softly and amply curved (convexity left, wide), insertion of hermaphroditic duct more anterior, at middle level of seminal receptacle duct. Fertilization complex simple, located at narrow and elongated base of seminal receptacle as duct of seminal receptacle; slightly shorter than length of receptacle. Fertilization complex totally immersed in albumen gland, inserting in posterior end of spermoviduct, by side of albumen gland duct, relatively wide, twice wider than receptacle's duct. Albumen gland (ag) solid, white, elliptical, more-or-less same size as gonad (~1/3 whorl). Albumen gland duct subterminal, connected to distal end of spermoviduct (Fig. 4B), in lateral, small albumen chamber (Fig. 4D: ac); narrowly connected to distal end of spermoviduct (Fig. 4B: eo). Spermoviduct (Figs. 2C, 24A-B: eo) of ~1.5 whorl in length, slightly narrower than albumen gland, ~10 times longer than wide; prostate wide (pt); glandular walls in uterus only along lateral side (Figs. 4B, E: ut), remaining ~2/3 of uterine inner surface relatively thin-glandular. Presence of accessory genital gland (Figs. 2B, E: as) lying along spermoviduct, flanking masculine furrows (sp), bulging along prostate (pt) side. Pair of masculine furrows lying along accessory genital gland (Figs. 4B, E: sp). Vas deferens originating in simple aperture slightly posterior to end of uterine level (Fig. 4A: vd). Vas deferens not coiled (Figs. 2C, 4C: vd). Genital muscles not detectable. Penis slightly shorter than half of spermoviduct length (Fig. 2C: pe); penis muscle inserted only between penis and epiphallus (Fig. 2C: pm), with small branch connected to vas deferens in its region preceding its insertion (Fig. 4C: pm). Epiphallus (eh) ~1/6 penis' length; vas deferens also inserted in middle level of outer epiphallus wall (Fig. 4C: vd). Epiphallus inner surface with high layer of papillae (Fig. 4D: eh). Internal penial arrangement of folds similar, but with fewer and wider folds, pair of more developed (main) folds (Fig. 4D: pf) not so developed, being wider and lower; papillae slightly smaller than those inside epiphallus also lying along entire distal 2/3 of penis (Figs. 2F, 4D).

Central nervous system. (Fig. 2A: nr) More detailed in following studied species, being noteworthy cerebral commissure wide; cerebral node (cd) not so developed; all connectives slightly shorter and all ganglia slightly larger.

Distribution. Apparently restricted to Rio de Janeiro region. Remaining records to other states possibly are misidentifications (personal observation, upcoming study).

Habitat. Atlantic Rainforest.

Measurements. (in mm) MZSP 152925#2 (Figs. 1A-C): 30.2 by 13.3.

Material examined. BRAZIL. **Rio de Janeiro**; São Fidélis, Serra da Sapateira, 21°39'S 41°46'W, MZSP 152925, 9 specimens (2 with only shells) (Femorale leg., 2010).

Taxonomic remarks. See Discussion item.

Anthinus synchondrus new species (Figs. 5–8)

ZooBank: urn:lsid:zoobank.org:act:64ABD2D2-71C4-49D7-AA69-4321AA8595EB

Types. Holotype MZSP 152074. Paratypes: MZSP 152252, 2 shells, MZSP 152075, 1 shell from type locality. BRAZIL. **Minas Gerais**; Unaí (W.V. Matos col, iv/2020), Gueda, 16°16'04"S 46°39'43"W, 647-650 m altitude, MZSP 152236, 1 shell.

Type locality. BRAZIL. **Minas Gerais**; Unaí, Pedra da Fartura, 16°31'37"S 46°49'18"W, 628-750 m altitude [Weslley Vailant de Matos col, iv.2020].

Etymology. The specific epithet is derived from Greek words *syn*, a prefix meaning "together", and *chondros*, meaning "gristle, cartilage". This is an allusion to the total fusion of the odontophore cartilages (Fig. 6D).

Diagnosis. Shell about 40 mm; color mosaic of brown-beige spots; spire angle ~35°, protoconch of 3.2 whorls; peristome ~48% of shell length and ~50% of width; width ~54 of length. Mantle edge with projected fold. Reno-pericardial area large. Pre-rectal pallial muscle present. Odontophore with cartilages totally fused; pair m4 ~50% originated from cartilage, remaining on m4. Jaw plate narrow. Radula with elongated, spatula-like mesocone, rachidial slightly different from neighboring teeth. Seminal receptacle tightly curved, hermaphrodite duct inserting in its base perpendicularly. Accessory genital gland in spermoviduct large; with two similar sperm-grooves. Penis ~60% of spermoviduct; penis muscle with pair of small branches in epiphallus; epiphallus papillae low, uniform, restricted to it; pair of larger folds composed of transverse subfolds.

Distinctive description

Shell. (Figs. 5A–L) Adult shell around 40 mm, conical-oval; apex blunt; wider at middle of last whorl; width ~54% of shell length. Walls weakly thick. Dorso-ventrally weakly flattened (Fig. 5E). Color light beige and brown squared spots, both predominating (Fig. 5C) or slightly brown predominating (Figs. 5H, J); brown spots slightly randomly distributed along 4-5 spiral, wide, rather isometric bands in last whorl, 2-3 spiral bands in penultimate whorl; usual concentration of brown pigment preceding peristome. Spire angle ~35°. Protoconch of 3.2 whorls (Figs. 5B, J, K), ~23% of length, width of ~7 mm, ~twice taller than wide; first whorl smooth, gradually axial and spiral narrow cords appearing (Figs. 5K, L), initially both predominating, gradually axial cords slightly stronger, from suture to suture, interspaced slightly broader than each cord, ~10 per mm; transition to teleoconch clear, slightly prosocline (Figs. 5B, H, K). Teleoconch sculpture similar to that of protoconch last whorl, as axial, uniform, complete cords and growth lines, gradually fading in last whorl (Figs. 5C, H, J); of ~2.3 whorls. Whorls profile weakly convex (Figs. 5G-J) (Figs. 5A-C, F, I, J). Suture well-marked, weakly channeled (Figs. 5J, K, L). Aperture slightly prosocline (~5° from longitudinal general axis) (Fig. 5B), oval; ~48% of shell length, ~50% of shell width. Peristome well-reflected, thick, color variating from pale beige (Figs. 5A, G) to white (Fig. 5I); outer lip arched, lacking middle tooth; inner lip with inferior half widely concave, superior half as narrow, weak callus with ~1/3 of peristome length (Figs. 5A, I), usually concave, rarely convex (Fig. 5G). Inferior half of inner lip intensely covering umbilicus (Figs. 5A, D, G, I). Umbilicus narrow (Fig. 5B). Body whorl ~55% of shell length; usually uniform with spire (Figs. 5A, G, I).

Head-foot. Similar features as *A. multicolor*, including columellar muscle having only two layers; i.e., secondary head muscles (ou, tm) originating direct from pair of secondary muscles (tm).

Mantle organs. (Fig. 6A) most characters similar to *A. multicolor*. Distinctions and remarks following. Mantle edge dorsal fold well developed (mf), with elongated edge pointed to left. Anus directly opening externally (ventrally), preceded by right longitudinal folds (Fig. 5N: af). Urinary gutter (ur) slightly broader, with slightly taller perpendicular folds.

Visceral mass. Similar features as preceding species.

Circulatory and excretory systems. (Fig. 6A) Most features similar to *A. multicolor*; remarks following. Pericardium (pc) lightly more elongated, ~3-times as long as wide. Kidney (ki) tissue clearly U-shaped (convexity left). Nephropore (ne) also protected by curved anterior projection of kidney. Urinary gutter (ur) slightly broader, mainly along its middle portion. Narrow urinary gutter surrounding left edge of anus (an) also present.

Digestive system. (Figs. 6C–8A) General features similar to those of *A. multicolor*, distinctions and interesting data following. Jaw slender, with uniform width along its length (Figs. 5M, 8A: jw), weak central beak; transverse ribs very weak, almost imperceptible; commarginal sculpture wanting. Buccal mass and odontophore (Fig. 6D) lacking m1a, m1v; **m3**, only detected in latero-dorsal region (Figs. 6C, 8A: m3); **m2**, slightly thinner, insertion narrow on m4 base (Fig. 6D); **m4**, very thick, relatively short; **m5**, thin and elongated, origin about half on m4 and half on posterior region of cartilages; **m7**, small narrow pair, origin in posterior end of cartilages, running inside radular sac, splaying along its inner surface. Pair of odontophore cartilages entirely fused with each other along their inner-ventral edge (Fig. 6D: oc). **Radula** (Fig. 7) with same general features of preceding species; with rachidian teeth, and ~40 pairs of lateral/marginal teeth; no clear distinction between lateral and marginal teeth (Fig. 7C); differing in having longer, rather rectangular mesocone, ~3 times longer than wide, covering ~1/3 of posterior tooth (Figs. 7B-C), mesocone apex rounded; long mesocone reinforced by longitudinal fold in inner concavity, continuous with base; base with similar characters as preceding species. **Rachidian** tooth (Figs 7A-C: arrow) slightly narrower than lateral teeth, with mesocone with ~half size of neighboring mesocones (Fig. 7C). **Lateral teeth** with weak medial inclination, rarely with bifid cutting edge (Fig. 7A: right side). **Marginal teeth** starting with no clear boundary with lateral teeth; shaped similarly to lateral teeth, except for being weakly smaller and narrower, row slightly more inclined (Fig. 7A: left side) along ~1/3 of each side. Salivary glands apertures as long longitudinal slits (Fig. 8A: sa), located in posterior region of lateral edges of wide dorsal folds (df). Esophagus broader all along its length (Fig. 6C: es). Stomach (st) ~50% larger, walls with considerable muscular walls; stomach inner surface (Fig. 6B) with fold (sf) running between anterior duct to digestive gland (dr) and intestine (in) more developed. Esophageal-anterior duct to digestive gland also bifid (Fig. 6C), left duct (dl) almost as wide as right duct (dr), much longer. Anus turned externally (Fig. 6A: an); inner surface also with 8–10 simple, longitudinal, subterminal, tall folds (Fig. 5N: af).

Reproductive system. (Figs. 8B-F) General characters similar to those described for *A. multicolor*, distinctions and remarks following. Hermaphroditic duct (Fig. 8B: hd) with broader coiled portions along its middle and distal thirds, with fewer but wider coils; insertion lacking curve (Fig. 8C). Seminal receptacle (Figs. 8B-C: sr) large, strongly and tightly curved (convexity left), forming distal blunt beak disposed pointing to hermaphroditic duct insertion. Spermoviduct (Figs. 8B-C: eo) with glandular walls in uterus only in ventral side (Fig. 8F: ut), remaining walls relatively thin-glandular. Presence of accessory genital gland (Figs. 8D, F: as) lying along spermoviduct, flanking masculine furrows (sp), bulging along prostate (pt) side. Pair of masculine furrows narrow, one lying along accessory genital gland, and other along its medial edge, ~half deep (Figs. 8D, F: sp). Vas deferens (vd) originating in simple junction of masculine furrows (Fig.

8D: vd), no vaginal fold. Vas deferens relatively broad, intensely coiled in its portion on free oviduct and vagina (Fig. 8B: vd). Penis slightly longer than half of spermoviduct length (Fig. 8B: pe); penis muscle inserting terminally, short, broad (Figs. 8B, E: pm), with pair of narrow branches connected to epiphallus. Epiphallus (eh) ~1/5 penis' length; vas deferens inserted subterminally on middle level of epiphallus wall (Fig. 8E: vd). Epiphallus inner surface as thick glandular, uniform layer of papillae. Internal penial surface with single chamber (Fig. 8E); arrangement of inner folds as in Fig. 8E, with 8-10 longitudinal wide folds, almost no interspace, in basal half; these folds gradually coalescent in middle penial region, additionally appearing narrower transverse folds in distal region; these folds forming two aligned bulged portions in penis side, producing pair of longitudinal cancelled wide folds (Fig. 8E: pf), separated by narrow furrow continuous with epiphallus aperture.

Central nervous system. Nerve ring located across buccal mass (Fig. 6C: nr). Pair of cerebral ganglia (ce) almost fused; cerebral commissure slightly narrower than ganglia; each ganglion about as wide as adjacent esophageal section; several wide nerves originating in cerebral antero-lateral region. Cerebral node or gland (cd) located in postero-medial quadrant, with ~1/8 each ganglion's size. On each side, parallel, rather narrow connectives (cn) running between cerebral ganglion and ventrally located fused pedal and pleural ganglia (pp), accompanied by blood vessels (bv) issuing from anteriorly-directed aorta. At least six pairs of nerves originating from anterior side of pedal-visceral ganglion complex. Pair of statocysts not seen.

Distribution. So far known in region of Unaí, Minas Gerais, Brazil.

Habitat. Cerrado biome, semi-dry forest, 628-750 m altitude.

Measurements. (length and width in mm) Holotype MZSP 152074 (Figs. 5A-F): 36.9 by 19.7; paratype MZSP 152252 (Figs. 5I-J): 36.4 by 19.6.

Material examined. Types (reported above).

Taxonomic remarks. See Discussion item.

***Anthinus vailanti* new species** (Figs. 9-12)

ZooBank: urn:lsid:zoobank.org:act:5CF7693D-4C22-4E66-9FCA-B0B821981E0B

Types. Holotype MZSP 154382. Paratypes: MZSP 152118, 3 spm, MZSP 152244, 2 shells, MZSP 152119, all from type locality.

Type locality. BRAZIL. **Minas Gerais**; North of Brasilândia de Minas, 16°59'29"S 46°00'29"W, 630-635 m altitude [Weslley Vailant de Matos col, iv.2020].

Etymology. The specific epithet is in honor of Weslley Vailant de Mattos, from Alcobaça, Bahia, the field collector responsible for most of the new discoveries.

Diagnosis. Shell about 30 mm; color mostly monochromatic; spire angle ~50°, protoconch of 3 whorls; peristome ~55% of shell length and ~60% of width; width ~60 of length. Mantle edge with pointed fold. Reno-pericardial area medium-sized. Pre-rectal pallial muscle present. Odontophore with cartilages ~90% fused with each other; pair m4 ~80% originated from cartilage, remaining on m4. Jaw plate broad, with central fold. Radula with elongated, spatula-like mesocone, rachidial slightly different from neighboring teeth. Seminal receptacle widely curved, hermaphrodite duct inserting in its middle region, perpendicularly. Accessory genital gland in spermoviduct large; with single, coiled sperm-groove. Penis ~60% of spermoviduct; penis muscle with pair of small branches in epiphallus; epiphallus papillae of medium size, not-

uniform, extending to penial chamber; pair of larger folds simple, fused with each other proximally.

Distinctive description

Shell. (Figs. 9A–J) Adult shell around 30 mm, conical-oval; apex blunt; wider at middle of last whorl; width ~60% of shell length. Walls thin. Dorso-ventrally weakly flattened (Fig. 9J). Color uniform reddish brown in ~half of specimens (Figs. 9A-G); to sparse squared pale brown spots on uniform cream base in other half of specimens (Figs. 9H-J), spots less dense than preceding species, but with similar remaining features, with areas denser (Fig. 9J) and areas almost lacking spots (Fig. 9I). Periostracum heavy, deciduous in some areas, mainly in apex (Figs. 9A-G). First spire whorls white in superior half, gradually becoming pale brown in inferior half (Figs. 9D-E). Spire angle ~50°. Protoconch of 3.0 whorls (Figs. 9B, E), ~20% of length, width of ~8.8 mm, slightly taller than wide; first whorl smooth, gradually axial and spiral narrow cords appearing (Figs. 9D-E), initially both predominating, gradually axial cords slightly stronger, from suture to suture, interspaced slightly broader than each cord, ~6 per mm, last protoconch whorl with spiral sculpture more spaced, producing rectangular spaced with axial lines; transition to teleoconch clear, slightly prosocline (Fig. 9E). Teleoconch sculpture similar to that of protoconch last whorl, as axial, uniform, complete cords and growth lines, gradually small punctuations appearing on axial lines before penultimate whorl, aligned in order to produce spiral punctuated lines, ~20 in penultimate whorl (Fig. 9E), lying next to peristome (Figs. 9C, F, J); of ~2.2 whorls. Whorls profile weakly convex (Figs. 9A-J). Suture well-marked, weakly channeled (Figs. 9I). Aperture slightly prosocline (~20° from longitudinal general axis) (Fig. 9B), elliptic; ~55% of shell length, ~60% of shell width. Peristome reflected, thick, color white (Figs. 9A, F, H); outer lip arched, lacking middle tooth; inner lip with inferior half weakly concave, superior half as narrow, weak callus with ~2/3 of peristome length (Figs. 9A, F, H), almost straight. Inferior half of inner lip covering umbilicus. Umbilicus relatively wide (Fig. 9J). Body whorl ~80% of shell length; usually uniform with spire (Figs. 9F-J) to slightly broader (Figs. 9A-C).

Head-foot. Similar features as *A. multicolor*, except for more developed pedal glands, bulging in haemocoel floor.

Mantle organs. Most characters similar to *A. multicolor*. Except for urinary gutter ~5% broader,

Visceral mass. Similar features as preceding species.

Circulatory and excretory systems. Most features similar to *A. multicolor*; except for renal anterior fold proportionally smaller, ~half size. Renal glandular lobe also U-shaped, but with ventral branch with double thickness of dorsal branch.

Digestive system. General features similar to those of *A. multicolor*. Remarks following. Jaw plate (Fig. 9K-L) much broader and thicker; cutting edge blunt, with median transverse reinforcement (Fig. 9L) in its ventral side. Buccal mass possessing m1v pair. Pair of salivary ducts slightly broader. Odontophore (Fig. 10A) differing in having **m5** pair only ~20% originating from cartilages, remaining originating on m4; **m7** pair originated in dorsal-inner surface of posterior region of cartilages; odontophore cartilages (oc) only ~90% fused, with small posterior remaining notch. **Radula** (Fig. 11) similar to that of *A. synchondrus*, with ~50 pairs of lateral/marginal teeth (Figs. 11A, D); distinctions following. **Rachidian** tooth (Figs. 11B, E: arrow) further smaller and narrower; mesocone slightly claviform. **Lateral teeth** not inclined, pointing posteriorly only; mesocone slightly more elongated, covering ~1/3 of neighboring posterior tooth. **Marginal teeth** inclined outside, located in more arched row (Figs. 11C, D: left side). Anus subterminal folds taller and stronger, 4-5 in number only; occupying ~10% of rectum length.

Reproductive system. (Figs. 10B-D, 12A–B) General characters similar to those described for *A. multicolor*, distinctions and remarks following. Hermaphroditic duct (Fig. 10C: hd) also broad; coiled portions occupying almost its entire length, except for short stretch at both ends; insertion lacking curve (Fig. 10B). Seminal receptacle (Figs. 10B–C: sr) relatively small, strongly and widely curved (convexity left), insertion of hermaphroditic duct in middle of concavity. Spermoviduct (Figs. 10B, C: eo) with narrower prostate (pt); glandular walls in uterus only along latero-ventral side (Figs. 10D, 12A: ut), remaining ~1/3 of uterine inner surface relatively thin-glandular. Accessory genital gland (Figs. 10B, D, 12A: as) also present and large. Only single detectable masculine furrow lying along accessory genital gland (Figs. 10D, 12A: sp). Vas deferens originating in simple aperture in end of uterine level (Fig. 12A: vd), also lacking vaginal fold. Vas deferens also coiled in its portion on feminine tubes, but less intensely (Fig. 10C: vd). Presence of pair of genital muscles (Fig. 10C: gm) in ventral side of penis base. Penis slightly longer than half of spermoviduct length (Fig. 10C: pe); penis muscle also with pair of narrow branches connected to epiphallus (Figs. 10C, 12B: pm). Epiphallus (eh) ~1/6 penis' length; vas deferens also inserted subterminally on middle level of epiphallus wall (Fig. 12B: vd). Epiphallus inner surface with high layer of papillae (Fig. 12B: eh). Internal penial arrangement of folds similar, but with pair of more developed (main) folds (Fig. 12B: pf) narrower and slightly taller; papillae similar to those inside epiphallus also lying at side of these main folds (Fig. 12B).

Central nervous system. With similar attributes as preceding species.

Distribution. So far known from type locality.

Habitat. Cerrado biome, semi-dry forest, 630-635 m altitude.

Measurements. (in mm) Holotype MZSP 154382 (Figs. 9A-E): 29.0 by 17.7; paratypes MZSP 152118 (Figs. 9F-G): 27.9 by 17.7; MZSP 152244 (Figs. 9H-J): 29.5 by 16.5.

Material examined. Types (reported above).

Taxonomic remarks. See Discussion item.

Anthinus morenus new species (Figs. 13–15)

ZooBank: urn:lsid:zoobank.org:act:A34D18E0-47B6-40C8-A3C8-7FF3367E3AE5

Types. Holotype MZSP 151891. Paratypes: MZSP 152047, 12 spm from type locality (3 with only shells), MZSP 152048, 2 shells. BRAZIL. **Minas Gerais**; Paracatu, Gruta Sapezal, 16°48'03"S 46°53'47"W, 700-710 m altitude, MZSP 152195, 3 shells (WV Matos col., iv.2020).

Type locality. BRAZIL. **Minas Gerais**; Paracatu, São José do Sapezal, 16°48'03"S 46°53'47"W, 700-710 m altitude [Weslley Vailant de Matos col, iv.2020].

Etymology. The specific epithet is in apposition, a Latinization based on the Portuguese word "moreno", meaning dark-brown colored, an allusion to the dark shell color.

Diagnosis. Shell about 35 mm; color usual mosaic of brown and beige spots; spire angle ~50°, protoconch of 3 whorls; peristome ~51% of shell length and ~51% of width; width ~60 of length. Mantle edge with simple, rounded fold. Reno-pericardial area small-sized. Pre-rectal pallial muscle present. Odontophore with cartilages ~50% fused with each other; pair m4 ~40% originated from cartilage, remaining on m4. Jaw plate narrow, lacking central beak. Radula with elongated, spatula-like mesocone, rachidial rather similar to neighboring teeth. Seminal receptacle tightly curved, hermaphrodite duct inserting in its base, perpendicularly. Accessory

genital gland in spermoviduct large; with pair of thick-walled sperm-grooves. Penis ~50% of spermoviduct; penis muscle mostly inserted to epiphallus; epiphallus papillae tall, restricted to it; single, large inner fold.

Description

Shell. (Figs. 13A-H, J-K) Adult shell around 35 mm, conical-oval; apex blunt; greatest width on last whorl; width ~60% shell length. Walls relatively thin. Color beige, with three spiral, equidistant brown bands up to penultimate whorl, five in last whorl; mosaic of sparse, apparently random transverse (axial) spots, irregular in size, normally limited to two neighboring spiral bands, but sometimes extending further (Fig. 13C, K). Spire angle ~50°. Slightly flattened dorso-ventrally, dorso-ventral axis ~80% of latero-lateral axis (Fig. 13E). Protoconch of 3 whorls (Figs. 13B, F, G, K), ~20% of length, width of ~7 mm; first whorl smooth, gradually axial narrow cords appearing (Figs. 13G-H), from suture to suture, interspaced similar to each cord, ~10 per mm; transition to teleoconch clear, slightly prosocline (Figs. 13B, F, G, K). Teleoconch of ~2.5 whorls; sculpture similar to that of protoconch last whorl in its first whorl, as axial, uniform, complete cords and growth lines (Fig. 13F); after first teleoconch whorl axial cords gradually disappearing, being substituted by rather irregular axial undulations and minute spiral aligned punctuations (Figs. 13C, F, J), ~25 spiral lines in penultimate whorl, interval between lines equivalent to their width up to double of it; spiral punctuated lines relatively uniformly covering surface up to peristome (Figs. 13B, C, K). Whorls profile slightly convex. Suture well-marked, slightly channeled (Figs. 13B, F, J). Aperture slightly prosocline (~7° from longitudinal general axis) (Fig. 13B), elliptic; ~51% of shell length, ~51% of shell width. Peristome reflected, thick, pure white (Figs. 13A, B) to pale beige (Fig. 13J); outer lip arched, lacking tooth (Figs. 13A, J); inner lip uniformly arched, concave; callus narrow, in superior third (Figs. 13A, J). Inferior 2/3 of inner lip partially covering umbilicus. Umbilicus relatively narrow (Fig. 13D). Body whorl ~84 shell length; usually looking wider than if spire had uniform growth (Figs. 13C, K).

Head-foot. (Fig. 14A) of normal shape, with characters similar to *A. multicolor*. Differing in pair of secondary cephalic muscles thicker, broader, and separated from each other almost up to their posterior end. Pedal gland not protruding in haemocoel.

Mantle organs. (Fig. 13L) most features similar to *A. multicolor*, differences following. Pallial roof uniformly pigmented dark (pu). Except for pulmonary vein (cv), all remaining pulmonary vessel not protruded, only visible with light passing through roof. Secondary mantle edge fold (mf) smaller, not pointed at left. Lung longer, almost 2 whorls. Reno-pericardial structures smaller, occupying ~10% of roof area. Urinary gutter smooth, lacking transverse folds.

Visceral mass. of similar attributes of preceding species.

Circulatory and excretory systems. (Fig. 13L) with same features as preceding species, except in being ~40% smaller.

Digestive system. General features similar to those of preceding species. Remarks following. Jaw plate (Fig. 13I) narrow, relatively thinner, including its cutting edge. Pair of salivary glands ~30% less developed. Odontophore (Fig. 14B) differing in having **m5** pair with narrower origin, ~40% originating from cartilages, remaining originating on m4; **m7** absent; odontophore cartilages (oc) only ~50% fused, with long posterior notch separating both. **Radula** (Fig. 15) mostly similar to *A. synchondrus*, with ~60 pairs of lateral/marginal teeth; distinctions and remarks following. Mesocone inner reinforcement clearly shown in Fig. 15C (central column, with teeth in profile), running along mesocone, becoming thicker and attaching to base. All teeth narrower and more elongated, ~4-5 times longer than wide (Figs. 15B, F); some rare column bifid (Fig. 15C: right side). **Rachidian** tooth of difficult visualization (Figs. 15A, B, E), ~1/3 narrower and shorter than lateral tooth, and slightly dislocated anteriorly in relation to its row (Fig. 15D: arrow). **Lateral**

teeth with very elongated mesocone, widely curved posteriorly (Fig. 15D). **Marginal teeth** with no clear boundary with lateral teeth, as gradual diminishment in lateral regions (Figs. 15E-F, inferior region).

Reproductive system. (Figs. 14C-F) General characters similar to those described for preceding species, distinctions and remarks following. Hermaphroditic duct (Fig. 14D: hd) also broad; coiled portions occupying almost its entire length, except for short stretch at both ends (Fig. 14E). Seminal receptacle (Figs. 14D-E: sr) relatively small, strongly and tightly curved (convexity left), insertion of hermaphroditic duct in receptacle base, lacking curve (Fig. 14E). Spermoviduct (Figs. 14D, E: eo) with glandular walls in uterus along its entire outer side (Figs. 14E, F: ut). Accessory genital gland (Figs. 14E, F: as) also present and large. Pair of masculine furrows narrow, one lying along accessory genital gland, and other along its medial edge, ~half deep (Figs. 14E, F: sp). Vas deferens originating in simple aperture in end of uterine level, also lacking vaginal fold. Vas deferens also coiled in its portion on feminine tubes, but less intensely (Fig. 14D: vd). Presence of single genital muscle (Fig. 14D: gm) in ventral side of vaginal base. Penis ~half of spermoviduct length (Fig. 14D: pe); penis muscle mostly inserted in epiphallus (Figs. 14D: pm) with additional small portion connected to penis distal end. Epiphallus (eh) ~1/4 penis' length; vas deferens also inserted subterminally in middle level of epiphallus wall (Figs. 14C, D: vd). Epiphallus inner surface with high layer of papillae (Fig. 14C: eh). Internal penial arrangement of folds similar, but with single more developed (main) fold (Fig. 14C: pf) broad and slightly taller; penial wall substantially thick muscular, inner set of folds (Fig. 14C) softer and simpler.

Central nervous system. With equivalent attributes as preceding species; except having longer cerebral commissure

Distribution. Paracatu region, Minas Gerais.

Habitat. Cerrado biome, semi-dry forest, 700-710 m altitude.

Measurements. (length and width in mm) Holotype MZSP 152891 (Figs. 13A-H): 35.8 by 21.0; paratype MZSP 152047#1 (Figs. 13I-L): 34.8 by 20.8.

Material examined. Types (reported above).

Taxonomic remarks. See Discussion item.

Anthinus savanicus **new species** (Figs. 16-19)

ZooBank: urn:lsid:zoobank.org:act:7816D208-22FE-4C19-BEE5-ED2DD68B3FAD.

Types. Holotype MZSP 154391. Paratypes MZSP 153897, 6 spm, MZSP 152171, 4 shells, all from type locality.

Type locality. BRAZIL. **Goiás**; north of Formosa, 15°21'50"S 47°27'08"W [Weslley Vailant de Matos col, ix.2012].

Etymology. The specific epithet is derived from the regional biome, called *Savana Acidentada* (rugged savanna).

Diagnosis. Shell about 32 mm; color usual mosaic of brown and beige spots; spire angle ~50°, protoconch of 2.8 whorls; peristome ~48% of shell length and ~51% of width; width ~60% of length. Mantle edge with projected fold. Reno-pericardial area large-sized. Pre-rectal pallial muscle present. Odontophore with cartilages ~85% fused with each other; pair m4 ~50% originated from cartilage, remaining on m4. Jaw plate broad, thick. Radula with elongated, spatula-like mesocone, rachidial rather similar to neighboring teeth. Seminal receptacle tightly

curved, hermaphrodite duct inserting in its base, curved. Accessory genital gland in spermoviduct small; with pair of sperm-grooves, one being T-shaped. Penis length ~80% of spermoviduct; penis muscle mostly inserted in penis distal end, with thick branch to epiphallus; epiphallus papillae tall, restricted to it; large pair of penial inner folds at end of secondary folds.

Distinctive description

Shell. (Figs. 16A–F, I–K) Adult shell around 32 mm, conical-oval; apex blunt; wider at middle of last whorl; width ~60% of shell length. Walls slightly thick. Dorso-ventrally slightly flattened (Figs. 16D, E). Color light beige and brown squared spots, both predominating; brown spots randomly distributed along 4-5 spiral, wide, rather isometric bands in last whorl, 2-3 spiral bands in penultimate whorl; usual concentration of brown pigment preceding peristome (Figs. 16C, J). Spire angle ~50°. Protoconch of 2.8 whorls (Figs. 16B, D, F, J), ~19% of length, width of ~7.7 mm, slightly taller than wide; 5-6 narrow, spiral lines present since first whorl, equally spaced, gradually more spiral and axial narrow lines appearing (Fig. 16F), initially spiral lines predominating, gradually in last whorl both predominating, gradually axial cords slightly stronger, from suture to suture, interspaced by similar width as lines', ~7 per mm; transition to teleoconch clear, slightly prosocline (Figs. 16B, F, J). Teleoconch sculpture similar to that of protoconch's last whorl, as axial, uniform, complete cords and growth lines; cords containing punctuations, aligned with their neighboring cords producing spiral punctuated cords (Figs. 16A, B, I, J); ~18 lines in penultimate whorl. Suture well-marked, weakly channeled (Figs. 16J, K). Aperture slightly prosocline (~10° from longitudinal general axis) (Fig. 16B), elliptical; ~48% of shell length, ~51% of shell width. Peristome reflected, thick, color white (Figs. 16A, I, K); outer lip arched, lacking middle tooth; inner lip with inferior half shallowly concave, superior half as narrow, weak callus with ~2/5 of peristome length (Figs. 16A, I, K), usually convex. Inferior half of inner lip intensely covering umbilicus. Umbilicus relatively wide (Fig. 16E). Body whorl ~80% of shell length; uniform with spire (Figs. 16C, J, K).

Head-foot. (Fig. 16H) Similar features as preceding species.

Mantle organs. (Fig. 16H) most characters similar to preceding species. Distinctions and remarks following. Pallial cavity ~0.75 of whorl. Mantle edge dorsal fold (mf) with very elongated edge pointed to left. Urinary gutter (ur) slightly narrow, transverse folds low.

Visceral mass. Similar features as preceding species.

Circulatory and excretory systems. (Fig. 16H) Most features similar to preceding species; except in being proportionally larger, occupying ~40% of pallial cavity (pc, ki).

Digestive system. (Figs. 17A-B) General features similar to those of preceding species, distinctions and interesting data following. Jaw strong, well-arched, with central inner reinforcement (Fig. 16G), weak central beak; transverse ribs very weak, almost imperceptible; commarginal sculpture wanting. Buccal mass and odontophore (Fig. 17B) lacking m1a, m1v and m3d; **m3p**, well-developed, extending along base of esophageal origin; **m4**, very thick, relatively short; **m5**, thin and short, origin about half on m4 and half on posterior region of cartilages; **m7**, absent. Pair of odontophore cartilages ~85% fused, with small posterior medial notch separating both cartilages (Fig. 17B: oc). **Radula** (Fig. 18) virtually similar to preceding *A. morenus*, with ~50 pairs of lateral teeth; but with all teeth slightly shorter and wider (Figs. 18A, C, D), mesocone reinforcement (Fig. 18B) and bifid column (Fig. 18A: center) also detected. **Rachidian** tooth (Fig 18E: arrow) similar to lateral teeth, except in being slightly smaller. **Marginal teeth** also turned outside, but proportionally larger, more similar to lateral teeth (Fig. 18E: left side). Salivary ducts narrow, elongated (Fig. 17A: sd). Esophagus broader all along its length, inner folds longitudinal, rather irregular (Fig. 17A: es). Stomach (st) large, walls considerably muscular; stomach inner

surface (Fig. 17A) lacking developed fold, only having small transverse folds (sf) close to duct to digestive gland (dl). Esophageal-anterior left duct (dl) also broad and long.

Reproductive system. (Figs. 17C, D, 19A–C) General characters similar to those described for preceding species, distinctions and remarks following. Hermaphroditic duct (Fig. 17C: hd) with coiled portions only in distal half, with fewer and simpler coils; insertion with strong curve (Figs. 17C, 15A: hd). Seminal receptacle (Figs. 17C, 19A: sr) large, strongly and tightly curved (convexity left), forming distal blunt beak pointing to hermaphroditic duct insertion. Spermoviduct (Figs. 17C, 19A, B: eo) with glandular walls in uterus relatively low and splayed (Fig. 17D: ut). Accessory genital gland relatively smaller (Figs. 17D, 19B: as), only present in basal 2/3 of spermoviduct, distal third lacking this gland (Fig. 19A); masculine furrows composed of pair of separated folds flanking wide groove (Figs. 17D, 19B: sp). Vas deferens (vd) originating in simple junction of masculine furrows (Fig. 19B: vd), no vaginal fold. Vas deferens relatively broad, coiled only in some sparce portions (Fig. 17C: vd); becoming very narrow along penial region. Penis almost as long as spermoviduct length (Fig. 17C: pe); penis muscle inserting as pair of relatively same-sized wide branches attached both, to epiphallus and to distal penial end (Fig. 17C: pm). Epiphallus (eh) ~1/6 penis' length; vas deferens inserted subterminally on middle level of epiphallus wall. Epiphallus inner surface as thick glandular, uniform layer of papillae (Fog. 10C: eh. Internal penial surface with single chamber (Fig. 19C); arrangement of inner folds as in Fig. 19C, with 8-10 longitudinal wide, low folds, almost no interspace, along entire penis length; these folds distally as pair of longitudinal multilobed folds (Fig 19C: pf) restricted to single side, with wide, smooth groove separating them; this groove continuous to epiphallus aperture (Fig. 19C).

Central nervous system. with equivalent attributes as preceding species, except for cerebral pair of ganglia more closely located, with wider commissure; and for slightly more antero-posteriorly elongated cerebral and pedal pair of ganglia.

Distribution. So far known the type locality.

Habitat. Cerrado biome.

Measurements. (length and width in mm) Holotype MZSP 154391 (Figs. 16A–C): 32.5 by 20.3; Paratype MZSP 153897 (Figs. 16I–L) 31.5 by 18.7; Paratype MZSP 152171 (Fig. 16K): 35.3 by 20.1.

Material examined. Types (reported above).

Taxonomic remarks. See Discussion item.

Genus *Catracca* new genus

ZooBank. urn:lsid:zoobank.org:act:C9AEAD6E-EA30-4584-A884-AB967AE8CE6A

Diagnosis. shell of relatively thick walls; spire tall, with blunt tip; narrowly umbilicate. Protoconch of three whorls; first whorl smooth; narrow close-placed axial cords uniformly disposed in remaining whorls, from suture to suture. Peristome deflected, callus narrow. Color uniform, lacking spots. Pallial cavity lacking septum, weak vascular network. Kidney with anterior projection protecting nephrostome. Odontophore with pair m5 originated part from pair m4 and part from pair m2. Stomach with walls relatively muscular. Seminal receptacle balloon-like, non-curved. Spermoviduct lacking accessory genital gland. Epiphallus short, rounded, amply connected to penis. Penis internally comprising two chambers separated by strong and

complex folded, transverse, ring-like, its distal edge comprising tall fold with well-developed sphincter.

Type species. ***Catracca uhlei*** new species.

List of included taxa. ***Catracca uhlei***.

Etymology. The genus' name is in apposition and is derived from the Portuguese word for ticket gate, *catraca*, as a make-up word; anyhow, its central region usually has a reinforced core sustaining the bars, this piece resembles the described shell. Gender: feminine.

Taxonomic discussion. see Discussion item.

Catracca uhlei **new species** (Figs. 20–24)

ZooBank: urn:lsid:zoobank.org:act:F8EEDDB8-F57F-40C2-831E-5F667C529BCF

Type specimens. Holotype MZSP 151900. Paratypes: MZSP 151901, 17 shells, MNRJ 23647, 1 shell, USNM 1661730, 1 shell, all from type locality. BRAZIL. **Minas Gerais**; Itacarambi (W.V. Matos col), entrance of Parque Nacional do Peruaçu (ii.2019), 15°10'30"S 44°13'24"W, 530 m altitude, MZSP 152161, 21 shells, MZSP 152222, 15 shells, MZSP 152242, 11 shells, Vargem Grande, 15°00'50"S 44°04'34"W, 466 m altitude, MZSP 151871, 3 specimens (7.ii.2020); Januária, border of Parque Nacional do Peruaçu, 14°57'55"S 44°04'21"W, 485 m altitude, MZSP 151893, 1 specimen, MZSP 151892, 10 shells (i.2020).

Type locality. BRAZIL. **Minas Gerais**; West of Itacarambi, 15°00'38"S 44°07'10"W, 540-550 m altitude [Weslley Vailant de Matos col, ii.2019].

Etymology. The specific epithet is in honor of Mauricio Sergio Uhle, São Paulo, a shell collector and sponsor of expeditions.

Description

Shell. (Figs 20A–I) Adult shell around 45 mm, conical-oval; apex blunt; wider on last whorl; width ~1/2 shell length. Walls relatively thick. Color uniform light beige or yellowish pale beige; some few specimens with reddish-brown tone in inferior third of spire whorls (Fig. 20F). Spire angle ~40°. Protoconch of 3 whorls (Figs. 20B, E), ~23% of length, width of ~9 mm; first whorl smooth, gradually axial narrow cords appearing (Figs. 20H, I), running from suture to suture, interspaces similar to each cord's width, ~10 per mm; transition to teleoconch distinct, slightly prosocline (Figs. 20E, H). Teleoconch of ~2 whorls, profile slightly convex; sculpture similar to that of protoconch last whorl, as axial, uniform, complete cords and growth lines, slightly more irregular on last whorl (Figs. 20A, C, D, G). Suture well-marked, slightly channeled (Figs. 20B, C, E, G). Aperture slightly prosocline (~10° from longitudinal general axis) (Fig. 20B), oval; ~45% of shell length, ~50% of shell width. Peristome continuous, reflected, thick, same color as remaining shell; outer lip arched, usually with small, blunt tooth medially (Figs. 20A, D, G) absent in younger adults (Fig. 20F); inner lip with inferior half almost straight, superior, parietal half appressed to body whorl, with thick callus, sometimes planar in last whorl (Figs. 20D, F) sometimes flanked by shallow outer furrow (Figs. 20A, F). Inferior half of inner lip partially covering umbilicus. Umbilicus relatively wide (Figs. 20A, D, G), ~10% of inferior area. Body whorl ~2/3 shell length; usually regularly increasing in size (Figs. 20A, D, F), more rarely wider (Fig. 20G).

Head-foot. (Fig. 21B) of normal shape. Color uniformly pale. Columellar muscle thick, 1.5 whorls in length. Inner arrangement of columellar annexed muscles relatively complex. Main

columellar bundle (cm) occupying ventral floor of haemocoel, relatively flat, wide slightly broader than half of foot width. Pair of secondary cephalic muscles (sm) with middle and anterior regions approximately double thickness and half width those of columellar main bundle (left muscle slightly broader than right one); originating gradually along dorsal surface of main columellar bundle, running anteriorly on main bundle, inserting in ventral region of perioral region. Pair of tentacular/ommatophore muscles originating between posterior and middle thirds of main columellar muscle bundle, between both secondary cephalic muscles; gradually thickening and bifurcating towards anterior; strong bifurcation halfway between their detected origin and anterior end, dorso-lateral branches becoming short and strong ommatophore muscles (ou), inserting near tip of ommatophore (om); ventro-medial branches running towards anterior, dorsally to secondary cephalic muscles, halfway newly bifurcating, lateral branch becoming short tentacular muscles (tm), inserting in tip of tentacles (te); medial branch running further towards anterior, inserting dorsally to insertion of secondary cephalic muscles. Pedal gland large, curved, protruding in ventral region of buccal area (Fig. 21C: pg).

Mantle organs. (Fig. 21A) Mantle border thick, lacking pigments. Pneumostome (pn) protected by simple right ventral flap (pv), width ~1/5 of aperture length. Dorsal fold well developed (mf), occupying ~1/3 of dorsal mantle edge length; left end pointed, projected beyond its base. Pneumostome (pn) ~1/10 of shell aperture length, bearing exclusively air entrance and urinary gutter. Anus (an) separate aperture located at right, adjacent to pneumostome. Lung of 1.5 whorls in length, wide and elongated; right side ~1.5 times longer than left side. Pulmonary vessels conspicuous, but low; 6-7 stronger vessels perpendicularly disposed at right; 6-7 main vessels at left radially disposed; all pulmonary vessels visible, but low, i.e., weakly protruded. Pulmonary vein (cv) running longitudinally across pallial cavity roof medially or towards right, anterior end trifurcated. Reno-pericardial area beige, slightly triangular, located posteriorly within pallial cavity, its posterior abutting wall of visceral cavity, occupying ~30% of cavity length and ~40% of its width (details below). Rectum (rt) wide. Urinary gutter (ur) narrow, running along rectum; anterior urinary gutter surrounding left anal aperture.

Visceral mass. (Fig. 21C) ~3 whorls in length. Both digestive gland lobes brown in color; anterior lobe (da) flattened, occupying ~1/5 of visceral volume, located just posteriorly to pallial cavity, continuous to kidney. Posterior lobe (dg) larger, extending 2 spiral whorls, occupying ~60% of visceral volume. Stomach ~1/15 of visceral volume, located between both digestive gland lobes, about 3/4 whorl posterior to pallial cavity (st). Digestive tubes (described below) surrounding anterior lobe of digestive gland. Gonad multi-lobed, cream color, encased between posterior lobe of digestive gland and columella, occupying ~1/3 whorl, ~1/10 of visceral volume.

Circulatory and excretory systems. (Fig. 21A) Pericardium (pc) ~twice as long as wide, located obliquely between middle and left thirds of posterior end of pallial roof, appressed against right lateral side of kidney; occupying ~5% of lung area. Auricle (au) located anteriorly, as continuation from pulmonary vein (cv); ventricle (ve) located posteriorly, larger. Kidney (ki) simple, dorso-ventrally flattened; size reported above; somewhat triangular, width ~2/3 of length; internally organized as two distinct regions – a longitudinal hollow cavity on left; filled by sponge-like renal tissue on right ~2/3 from posterior to anterior ends, being gradually broader towards anterior. Nephropore (ne) small, longitudinal slit at anterior-left corner of kidney, directed towards right; protected by curved anterior projection of kidney (kf). Urinary gutter (ur) composed of small folds perpendicular to rectum axis, flanking almost entire rectum left edge, starting at short distance from pulmonary postero-right end, ending in pneumostome as narrow urinary groove flanking left edge of anus (an).

Digestive system. (Figs. 21C–23B) Oral tube wide, muscular (Figs. 21E: mj). Radula features (Fig. 22) below. Jaw simple, thick, dark brown, exposed portion horseshoe-shaped, thicker at middle (Fig. 20J); surface having only coaxial low undulations and growth lines; located ai

anterior end of buccal dorsal chamber (Fig. 23B: jw). Buccal mass spherical, occupying~1/5 of haemocoel volume (Fig. 21C: bm). Dorsal surface of oral cavity with well-developed pair of dorsal folds (Fig. 23B: df), width of each ~1/3 of dorsal wall width; separated from each other by dorsal chamber (dc) as wide as folds, but broader anteriorly. Odontophore with ~60% of buccal mass volume (Figs 21E, 23B: od). Odontophore muscles (Figs 21E–23B): **mj**, jaw and peribuccal muscles originating in outer-ventral surface of odontophore cartilages (Fig. 23A: mj), running towards dorsal, splaying in dorsal wall of oral tube (Fig. 21E: mj); **m1**, jugal muscles covering entirely haemocoelic structures, more concentrated close to mouth; **m1a**, small pair of lateral muscles, origin in latero-ventral region of haemocoel, running dorsally short distance, inserting in lateral surface of odontophore; **m1l**, small pair of lateral protractor muscles, origin in lateral region of buccal area, running towards posterior along 2/3 buccal mass length, inserting in lateral region of odontophore, anterior to m1a; **m1v**, small pair of ventral protractors jugal muscles, originating in ventral surface of haemocoel close to mouth, running towards posterior, inserting in ventral-posterior region of odontophore close to m2 insertion (Fig. 21E: m1v); **m2**, strong pair of retractor muscles of buccal mass, or radular muscles, originating as single bundle in columellar muscle posterior end (Fig. 21B: m2), running anteriorly close to median line along ~60% of haemocoel length, inserting as two different bundles in ventro-posterior edge of odontophore, surrounding at some distance radular nucleus (Fig. 23A: m2); **m3d**, thin layer of longitudinal fibers immersed in dorso-posterior wall of odontophore, preceding esophageal origin (Fig. 21E); **m3p**, thin layer of dorso-ventral fibers covering posterior odontophore wall, ventral to esophagus (Fig. 21E); **m4**, main pair of dorsal tensor muscles of radula, thick, originating in postero-medial region of odontophore cartilages (Fig. 23A: m4), surrounding outside and medially cartilages, inserting in subradular membrane in its region correspondent to buccal cavity; **m5**, pair of thick auxiliary dorsal tensor muscles of radula, originating on posterior-medial surface of m4 and short portion in postero-ventral region of odontophore cartilages (Fig. 23A: m5), , running towards median line covering m4, inserting in subradular membrane by side of m4 insertion; **m6**, horizontal muscle absent (Fig. 23A); **m7**, absent; **m10**, pair of narrow ventral odontophore protractor muscles, originating in ventro-anterior region of haemocoel, just ventral to mouth, running towards posterior along buccal mass length (Fig. 21E: m10), inserting in latero-posterior surface of odontophore close to m2 insertions; **m11**, pair of narrow ventral tensor muscles of radula absent. Odontophore non-muscular structures (Figs. 23A-B): **oc**, pair of odontophore cartilages flattened, rather elliptical, anterior region slightly projected anteriorly close to median line, ~1.2 times longer than wide, fused with each other along ~half in their anterior-medial edge (Fig. 23A: oc), posterior end roughly rounded; **sc**, subradular cartilage, with expanding region in buccal cavity protecting subradular membrane. Radular sac short, not extending beyond odontophore.

Radula. (Fig. 22) Slightly longer than odontophore; with rachidian teeth, and ~45 pairs of lateral/marginal teeth; no clear distinction between lateral and marginal teeth (Fig. 22A), marginal teeth only slightly narrower than lateral teeth, and more inclined medially (Fig. 22C: right side); all teeth with base as long as mesocone, articulating neighboring rows (Figs. 22D, F); mesocone arched, curved inwards and posterior, ~twice longer than wide, apex bluntly pointed; no secondary cusps. **Rachidian** tooth (Figs. 22E-F: arrow) as large as lateral teeth; base ~twice longer than wide, slightly flattened, barely triangular, with arched posterior edge articulating with neighbor tooth and pair of distal reinforcements; mesocone ~half of base, symmetrical, located slightly dislocated posteriorly from adjacent lateral teeth row (Figs. 22E-F). **Lateral teeth** similar to rachidian (Figs. 22B, D), except in being slightly longer, asymmetrical, arched towards medial region; base trapezoid, with medial concavity articulating with neighbor medial tooth and single distal reinforcement (Fig. 22F). **Marginal teeth** starting with no clear boundary with lateral teeth (Figs. 22A, C) occupying ~1/3 of each side; shaped similarly to lateral teeth, except for being slightly narrower and more inclined medially (Fig. 22C).

Salivary glands covering esophagus in its region preceding its anterior quarter (Fig. 21C: sg), forming two elongated, white, thin masses. Each salivary duct differentiable in middle and anterior side of glands, with ~1/12 of esophageal width (Figs. 21C, E, 4B: sd). Salivary duct running in both sides of esophageal origin, penetrating buccal mass wall in region close to buccal ganglia (Figs. 21E, 23B: sd), running immersed in buccal dorsal wall along ~1/3 its length (Fig. 23B: sd). Salivary ducts opening as small slits in middle level of dorsal folds, on their lateral side (Fig. 23B: sa).

Esophagus ~1-whorl long, with thin, flaccid walls (Fig. 21C: es); anterior 1/3 clearly broader, crop-like; posterior 2/3 narrower; inner surface simple, with 5–10 simple longitudinal folds. Stomach (Fig. 21C: st) narrow, curved, almost not bulging; position and size described above (visceral mass); gastric walls relatively thick muscular (Fig. 21D: sw); inner surface mostly smooth (Fig. 21D), except for narrow longitudinal fold along smaller curvature (sf), connecting esophageal duct to digestive gland with intestinal origin; series of low, right folds flanking anterior side of longitudinal fold. Esophageal insertion on right side, intestinal origin on left side, both close to columella. Duct to anterior lobe of digestive gland at short distance from esophagus and intestine intersection (Figs. 21C, D: dr) wide, running shortly towards right, bifurcating; additional left duct (Fig. 21C: dl) originated in main duct base with ~1/3 of its width, running narrowly towards left and anterior along digestive gland lobe compressed by first intestinal loop (da), periodically possessing short ramifications along its length. Duct to posterior lobe of digestive gland located short distance from intestinal origin, posterior to above-described duct, directed towards opposite side (Figs. 21C, D: dd) slightly wider than anterior duct. Intestine (in) initially as wide as esophageal insertion, gradually broadening up to ~double that width along its wide sigmoid loop in anterior lobe of digestive gland (Fig. 21C: in). Rectum and anus position described above (pallial cavity) (Fig. 21A: rt, an). Anus sessile, as slit in right end of mantle edge directly turned outside; inner surface with 8–10 simple longitudinal, relatively tall folds.

Reproductive system. (Fig. 23C-24B) Gonad position described above (visceral mass), composed of 8–10 lobes with minute digitiform acini (Fig. 21C: go). Hermaphroditic duct (Fig. 23C: hd) narrow and weakly coiled in posterior half, abruptly becoming wider (up to 3-times wider), more intensely coiled in anterior half, coiling diminishing only close to insertion (Fig. 24D: hd). Seminal receptacle (Figs. 23C, D: sr) large, oval, balloon-like sac, ~twice longer than wide, ~three times wider than hermaphroditic duct. Fertilization complex simple, located at narrow and elongated base of seminal receptacle (Fig. 23D) as duct of seminal receptacle; slightly shorter than length of receptacle. Fertilization complex totally immersed in albumen gland, inserting in posterior end of spermoviduct, by side of albumen gland duct, relatively wide, twice wider than receptacle's duct. Albumen gland (Fig. 23C, D: ag) solid, white, elliptical, more-or-less same size as gonad (~1/3 whorl). Albumen gland duct subterminal, connected to distal end of spermoviduct (Fig. 23D: ad), in lateral, large albumen chamber (Fig. 23D: ac); widely connected to distal end of spermoviduct (Fig. 23D: eo). Spermoviduct (eo) of ~1.5 whorl in length, slightly narrower than albumen gland, ~10 times longer than wide. Prostate gland occupying ~3/4 of spermoviduct surface and ~1/2 its volume (Figs. 23C, E: pt). Uterus occupying ~3/4 of spermoviduct space, external walls thick-glandular (Figs. 23C, E: ut), inner surface mostly covered by wide oblique folds (Fig. 23D: ut). Sperm groove entirely composed of two tall folds (Figs. 23D, E: sp), becoming vas deferens some distance posterior to genital pore (~1/15 of remaining spermoviduct length) (Figs. 23C, F: vd), with ~1/10 of anterior spermoviduct width, running outside of penis shield (Fig. 23C). Vagina ~1/20 spermoviduct length (Figs. 23C, F: vg); inner surface simple, with 8-10 longitudinal, low, wide folds (Fig. 23F: vg). Bursa copulatrix almost as long as spermoviduct length; bursa duct ~half of adjacent spermoviduct width in its origin, gradually narrowing towards posterior end (Fig. 23C: bd); bursa oval, ~1/3 of albumen gland size (Fig. 23C: bc), located encased between pericardium and adjacent intestinal loop. Penis ~3/4 of spermoviduct length, ~2/3 its anterior width (Fig. 23C: pe); penis muscle inserting

terminally, short, broad (Figs. 23C, 24A: pm), with no branch on epiphallus. Epiphallus ~1/7 penis' length, tip rounded, located laterally (Fig. 24A: eh) inner surface with smooth, widely opening to penis posterior chamber (Fig. 24B: eh). Vas deferens inserted subterminally in penis tip, in intersection with epiphallus (Figs. 24A, B: vd). Internal penial surface with two clear chambers (Fig. 24B), strong transverse septum dividing penis in about similar-sized posterior (ps) and anterior (pa) chambers; transverse septum having strong sphincter in edge (sh), and proximal blunt projection bulging in a side of anterior chamber (pf); this transverse septum swelling middle portion of penis externally (Figs. 23C, 24A); posterior penis chamber with wide furrow running from opposite side of epiphallus aperture, up to region close to transverse septum (Fig. 24B: ps); anterior penis chamber with strong, wide longitudinal fold located at base of blunt projection (pf), tapering towards anterior; low, wide longitudinal folds covering entire surface of anterior penis chamber (pa). Genital pore with radial arrangement of muscles, with pair of more developed muscles (Fig. 23C: gm) running dorsally and posteriorly, connecting with middle region of columellar muscle.

Central nervous system. (Fig. 24C) Nerve ring located across buccal mass (Fig. 21C: nr). Pair of cerebral ganglia (ce) almost fused; cerebral commissure slightly narrower than ganglia; each ganglion about as wide as adjacent esophageal section; several wide nerves originating in cerebral antero-lateral region. Cerebral node or gland (cd) located in postero-medial quadrant, with ~1/8 each ganglion's size. On each side, parallel, rather narrow connectives (cn) running between cerebral ganglion and ventrally located fused pedal and pleural ganglia (pp), accompanied by blood vessels (bv) issuing from anteriorly-directed aorta. At least six pairs of nerves originating from anterior side of pedal-visceral ganglion complex. Pair of statocysts not seen.

Distribution. Minas Gerais region around Parque Nacional Cavernas do Peruaçu, so far from Itacarambi (north) to São João da Ponte (south).

Habitat. Limestone outcrops, Cerrado forest, 466-759 m altitude.

Measurements. (length and width in mm) holotype MZSP 151900 (Figs. 20A-C): 44.2 by 23.5 (Figs. 20A-C); paratypes MZSP 151901#1 (Fig. 20D): 42.8 by 20.7; #2: 43.0 by 24.4 (Fig. 20G); MZSP 151893: 47.1 by 25.3 (Fig. 20F).

Material examined. Types (reported above). BRAZIL. **Minas Gerais** (W.V. Matos col); Itacarambi, Vargem Grande, 15°00'50"S 44°04'34"W, 466 m altitude, MZSP 151869, 3 shells (7.ii.2020); São João da Ponte, near Olímpio Campos, 15°50'44"S 44°00'03"W, 759 m altitude, MZSP 151811, 1 shell (i.2020).

Taxonomic remarks. See Discussion item.

Discussion

The taxon descriptions presented above do not include comparative remarks, which ~~shows~~are reported in this item. However, the ~~detailed~~descriptions are presented in a comparative scenario with the preceding ones, evidencing the main differences amongst the taxa herein studied. ~~This is particularly evidenced in given~~Besides, there are the diagnoses, which bring the set of character necessary to identify each taxon. The present section is, thus, more focused on the most interesting features and those more relevant to taxonomy and present/future phylogenetic approaches to resolving relationships among South American strophocheilids.

Generic debate

As reported above, *Anthinus* appears to encompass the smallest strophocheilids [6], and those with a slightly "Bulimulus"-like, bulimoid shell, i.e., a fusiform shell with a relatively small aperture. These features easily differentiate the genus from the remaining cofamiliar genera that are bigger and with proportionally larger aperture. *Catracca*, introduced here, actually breaks this rule, as it is similar to *Anthinus* in these features, so much so that samples were initially identified as belong to that genus. Despite the *Catracca* shell lacking the colorful pattern of *Anthinus*, the discovery of *A. vailanti*, which also possesses a rather monochromatic shell, affects the generic separation only based on conchology. The anatomical features were fundamental to determine ~~both as separate~~that these are distinct genera.

The set of characters reported here in diagnoses of both genera are sufficient to individualize them amongst the strophocheilids. However, it is recognized that the remaining genera do not have the same level of details known. Despite Leme [5] having redefined all strophocheilid (and megalobulimid) genera, including their anatomy, the level of details was not the same as those reported herein. Contrary to Leme's [5] definition of *Anthinus*, some characteristics were not confirmed in the present study. In particular, the pair of colored bands of the antero-dorsal region of the animal were not found. Further, all examined *Anthinus* species possessed the genital accessory gland (e.g., Figs. 4E, 10D, 14F: as) that was considered absent, and being an exclusive feature of the megalobulimines. Other characteristics reported in Leme's [5] diagnosis are accurate, such as the weakly muscular stomach lacking inner folds (e.g., Fig. 6B), the curvature of the seminal receptacle (e.g., Figs. 8C, 24B: sr) (which is also shared with other strophocheilines), and the shape of the epiphallus (e.g., Fig. 4C: eh). Other interesting characteristics that now can be added to the *Anthinus* diagnosis include the low profile of the pulmonary vessels, usual for species of smaller size, dark pigmented (e.g., Fig. 13L); the double origin of the odontophore muscle pair m5 (e.g., Fig. 6D); the posterior insertion of the anterior (esophageal) duct to the digestive gland, inserted practically into the stomach (e.g., Fig. 6B: dr), its bifurcation, with both components of similar caliber (e.g., Fig. 6C: dr, dl); the presence of the pallial, pre-rectal muscle (another supposed exclusive character of the megalobulimines); and the penis inner arrangement of folds forming a pair of stronger posterior folds, the furrow between which is continuous with the epiphallus aperture.

The new genus *Catracca* shares some characteristics with *Anthinus*, distinguishing both from other strophocheilids, such as the relatively small shell aperture; the small but distinct umbilicus (Figs. 20A, D); the configuration of the robust head musculature (Fig. 21B); the posterior position of the anterior duct of the digestive gland (Fig. 21C, despite the left branch, dl, is much narrower); and of the nerve ring (Fig. 24C). However, *Catracca* has a set of exclusive characters that justify its introduction, reported in its diagnosis above, some commented below. In some aspects, the *Catracca* protoconch resembles those of *Megalobulimus* Miller, 1878 in being blunt, wide and with uniform axial sculpture (Figs. 20E, H, I). It differs, however, in being much more elongated and of smaller absolute size. The other strophocheilid genera have the protoconch with delicate reticulated sculpture and much more acuminate [4, 6]; *Catracca* actually has the bluntest perspective, with a proportionally widest protoconch amongst the strophocheilines, producing a somewhat mamillated shell. From the anatomical diagnostic characters of *Catracca*, which particularly distinguish it from *Anthinus*, the more interesting are the thick muscular stomach (Fig. 21D: sw), which in muscular development is inferior only to the gizzard-like stomach of *Megalobulimus* [5]; the odontophore pair m5 entirely originated from the pair m4; the sac-like, non-curved seminal receptacle (Fig. 23D: sr); the absence of accessory genital gland in the spermoviduct (Fig. 23E); and by the pair of vaginal folds (Fig. 23F: vf). However, the more outstanding distinctive feature of *Catracca* is its inner penial arrangement (Fig. 24B). The large transverse septum with well-developed sphincter in its edge (sh) is unique amongst the strophocheilids. A structure approaching this was only found only in *Mirinaba* Morretes, 1955, particularly in *M. planidens* (Michelin, 1831)

([5]: fig. 54). However, in the latter, the transverse septum is much lower, thicker, and lacks the sphincter. Besides, the *Mirinaba* copulatory organ has an elongated epiphallus, more similar to those of *Megalobulimus*. The epiphallus in *Catracca* is most similar to that of *Anthinus*, short and rounded (Fig. 23A: eh); it differs, however, in lacking inner papillae cover (e.g., Fig. 14C, 20C: eh). Moreover, in *Catracca* the vas deferens inserts in the border between the epiphallus and penis (Figs. 23A-B), while in *Anthinus* the vas deferens inserts in the middle level of the outer epiphallus wall (e.g., Fig. 8E: vd). The penis muscle insertion is restricted to the penis wall in *Catracca* (Fig. 23A), while some branches of it insert on the epiphallus in *Anthinus* (e.g., Figs. 8B, 17C: pm).

Interestingly, in *Anthinus*, the radula of the type species *A. multicolor* looks more like that of *Catracca uhlei* than those of remaining studied congeners. ~~Both,~~ *A. multicolor* and *C. uhlei*, share short, blunt mesocones, particularly those of the rachidian teeth (Figs. 3C, 22F: arrow), while the mesocone of the remaining *Anthinus* species are much more elongated, flattened, spatula-like, with the rachidian more similar to lateral. The radulae of both genera differ from those of the megalobulimines in lacking broad mesocones, of almost the same size as the bases. The radulae of the other strophocheilid genera are not known.

The presence of supposedly megalobulimine characteristics in *Anthinus*, such as the pre-rectal pallial muscle (e.g., Fig. 17A: mp) and the accessory genital gland (e.g., Fig. 17D: as); as well as the megalobulimine supposed exclusive characters in *Catracca*, such as the protoconch (Fig. 20E) and the sac-like seminal receptacle (Fig. 23D: sr) are of difficult interpretation in respect to generic interrelationships. In the provisional phylogenetic treatment below, the accessory genital gland resulted in a convergence between megalobulimines and *Anthinus*. The same result (convergence) was obtained for the regular narrow axial cords in the protoconch shared between *Catracca* and some megalobulimines. The sac-like seminar receptable, on the other hand, appears to be plesiomorphic in the family.

Specific debate

A gallery of shells of all known *Anthinus* species are assembled in Figs. 25A–H, 26A–H, based on representatives (mostly holotypes – D–H), as well as of *Catracca uhlei* (Fig. 25I, 26I holotype). In that, the most outstanding feature is the monochromia of *A. vailanti* (Fig. 25F, 26F) and of *C. uhlei*, which already sets them apart from the remaining that possess characteristic beige-brown spots or blotches forming weakly-defined, spiral interrupted bands, i.e., pale spots or blotches on the beige-brown background. However, some specimens of *A. vailanti* possess some evidence of such banding (Figs. 9H-I). After that, the present debate focuses on *Anthinus* species, considering that the diagnosis of each species already brings the set of characters exclusive of each species that supported their description. The present discussion focuses in the characters, every numerical parameter is based on a N from 10 to 20 specimens and is an integer value. Related to the shell elongation, *A. multicolor* is the slenderer (width/length W/L index = 46%) (Figs. 1A–C, 25C), with a group orbiting W/L ~50%, such as *A. synchondrus* (Fig. 25E – 54%), *A. albolabiatus* (Fig. 25A – 49%), *A. miersi* (Fig. 25B – 51%) and *A. turnix* (Fig. 25D – 55%); while another group has shells broader, encompassing species with W/L = 60%, such as *A. vailanti*, *A. morenus* (Fig. 25G) and *A. savanicus* (Fig. 25H). In respect to the spire angulation, *A. synchondrus* has the sharpest pointed spire, ~35°, followed by *A. multicolor* ~40°, and *A. albolabiatus* ~42°; *A*~~,~~. *turnix* has the broader spire, with ~58°, followed by *A. miersi* ~55°; while the remaining species (*A. morenus, A. vailanti* and *A. savanicus*) have ~50°. The number of protoconch whorls is relatively constant amongst the species, with ~3 whorls; however, that of *A. synchondrus* looks slightly longer, with 3.2 whorls (Figs. 5K, L); while those of *A. multicolor* and *A. turnix* look shorter, with ~2.5 whorls, followed by *A. savanicus* with ~2.8 whorls (Fig. 16F). The protoconch absolute size, in mm, is also an important parameter, as usually is relatively constant within each species; the largest

protoconch is that of *C. uhlei*, with 9 mm of diameter; it is followed by that of *A. vailanti*, with 8.8 mm; in the range of ~7 mm there are *A. savanicus* – 7.7 mm, *A. albolabiatus* – 7.5 mm, and *A. synchondrus* and *A. morenus* with 7.0 mm; the range of ~6 mm starts with *A. turnix* – 6.8 mm. *A. miersi* – 6.3 mm, ending with the smallest protoconch of *A. multicolor* – 6.1 mm. In relation to the aperture, the amplest is that of *A. turnix*, with aperture length occupying 60% if shell length, and 67% of shell width; it is followed by *A. miersi* – 58%-60% respectively; the smallest proportional peristome is that of *C. uhlei* – 45%-50%, followed by *A. synchondrus* – 48%-50% and *A. savanicus* – 48%-51%; the remaining species are in the range of peristome size about half of the shell's size, as follows: *A. morenus* – 51%-51%, *A. albolabiatus* – 52%-54%, *A. multicolor* – 52%-55%, and *A. vailanti* – 55%-60%. The shell's absolute size is also an interesting parameter, with *A. miersi* being the larger, with an average of 53 mm, followed by *A. turnix* – 50 mm; the smallest species is *A. vailanti*, with average size of 30 mm, followed by *A. savanicus* – 32 mm, and *A. morenus* – *A. multicolor*, both with 35 mm; *A. synchondrus* – 40 mm, and *A. albolabiatus* – 45 mm, are intermediary. The average size of *C. uhlei* is 45 mm.

The Table 1 summarizes the main conchological differences among the species studied herein.

Table 1. Summary of main conchological differences among the species studied in this paper (all measures are approximations of average measures of several specimens)

species	Shell size (mm)	Shell color	Spire angle	Protoconch whorls	Peristome x length	Width x length	Apex
Anthinus multicolor	35	mosaic	40°	2.5	52%	46%	narrow
Anthinus synchondrus	40	mosaic	35°	3.2	48%	54%	narrow
Anthinus vailanti	30	Monoch-romatic	50°	3	55%	60%	narrow
Anthinus morenus	35	mosaic	50°	3	51%	60%	narrow
Anthinus savanicus	32	mosaic	50°	2.8	48%	60%	narrow
Catracca uhlei	45	Monoch-romatic	40°	3	45°	50%	blunt

Regarded to *Anthinus multicolor*, a more detailed discussion on the loss of the types, geographic distribution, and shell variation, is found elsewhere [4]. In the original description [29], Rang reported that the examined material came from Brazil, particularly Saint-Paul province (the State of São Paulo), and from near Corcovado, which today is a place inside the city of Rio de Janeiro, State of Rio de Janeiro. Bequaert [4] reported the species as occurring in these two states (Rio de Janeiro and São Paulo), and figured a topotype ([4]: pl. 4, figs. 3, 7; MCZ 74441, from Corcovado), which is similar to the original illustration ([29]: pl 3, fig. 1). The specimen has irregular dark spots in its shell, lacking apparent spiral alignment as usually have the other species (Fig. 25). The shell illustrated by Simone [6] (Fig. 25C) was collected in Teresópolis, located close to Serra dos Órgãos, contiguous with the Corcovado rainforest. It also shows the same spot patterns of the previous illustrations [4, 29]. The presently studied sample were collected in São Fidélis, a place ~80km far from Corcovado, but also apparently in the same biome, as it is close to the Desengano State Park. The shells of the presently studied sample (Figs. 1A-C) also have the irregular, not spirally aligned spots, but they possess a wide spiral clear band along the middle level of the last whorl. This band is not present in all specimens of that lot, and, thus, is interpreted as variation. The non-alignment of the dark spots is, thus, an interesting species' distinctive character.

Related to anatomy, beyond the species described herein, there is some information only on *A. miersi* (radula, pallial roof and genitalia [7]) and the generic statement by Leme [5]. The anatomical discussion on the other species is, thus, precluded, and even on *A. miersi* the details are not equivalent. The mantle edge of the studied *Anthinus* has the usual pattern of most pulmonates, which includes a small left fold from the pneumostome (e.g., Fig. 21A: mf, which is pointed to the left); this fold has shown relatively constant in each species and of taxonomical value, in *A. synchondrus* and in *A. savanicus*, it has an elongated projection (Figs. 6A, 16H: mf); it is pointed in *A. vailanti*, and simply rounded in *A. morenus* and *A. multicolor* (Figs. 13L, 22A). The pallial cavity is interestingly darkly pigmented in all species (e.g., Fig. 134H: pu), in which the pulmonary vessels are difficult to discern. The different pattern of lung vascularization is evident in the drawings represented for each species. Furthermore, the relative size of the reno-pericardial area varies: *C. uhlei* and *A. vailanti* (Fig. 21A), considered of medium size (~1/4-1/5 of total cavity area); while *A. synchondrus* (Fig. 6A) and *A. savanicus* (Fig. 16H) can be considered large, occupying ~1/3 of lung area; and *A. morenus* (Fig. 13L), and *A. multicolor* (Fig. 2A), can be considered small (about 1/5 of lung area). The pallial pre-rectal muscle, as referred above, is supposedly a megalobulimine characteristic [5], but found in all here studied *Anthinus* examined here (Figs. 6C, 17A: mp) except for *A. multicolor* (Fig. 2A). In the digestive system, the jaw plate shows interesting comparative data; it is deeper and broad in *C. uhlei* (Fig. 20J), *A. vailanti* (Figs. 9K-L), *A. savanicus* (Fig. 16G), *A. multicolor* (Figs. 1G-H), and possibly in *A. miersi* [7]; however, it is shallow and wide in *A. synchondrus* (Fig. 5M), and *A. morenus* (Fig. 13I); moreover, a ventral additional medial fold reinforces the jaw plate exclusively in *A. vailanti* (Fig. 9L), and the jaw lacks a median anterior projection or beak only in that of *A. morenus* (Fig. 13I). Related to the odontophore intrinsic and extrinsic musculature, they have shown lightly similar; the most variable pairs are the m1, m3 and m7, which can be modified, present or absent according to the species (see descriptions), however, the variation found in pair m5 appears to be more interesting; it is 100% originated from m4 in *C. uhlei*, but it is ~50% originated from m4 and 50% from cartilages in *A. synchondrus, A. savanicus* and *A. multicolor*, it is ~60% originated from m4 in *A. morenus*, and ~20% in *A vailanti* (Fig. 10A). The degree of odontophore cartilages fusion along the ventral-inner edge is also of taxonomic interest, both cartilages are ~50% fused in *C. uhlei* (Fig. 23A) and *A. morenus* (Fig. 14B); ~90% fused in *A. vailanti* (Fig. 10A) and *A. multicolor*; ~85% fused in *A. savanicus* (Fig. 17B); while the cartilages are entirely fused in *A. synchondrus* (Fig. 6D). The radular teeth's mesocone is short, compared to those of the other genera of the family, in *C. uhlei* (Fig. 22), *A. multicolor* (Fig. 23), and *A. miersi* [7]; however, it is elongated, spatula-like in *A. synchondrus* (Fig. 7), *A. vailanti* (Fig. 11), *A. morenus* (Fig. 15), and *A. savanicus* (Fig. 18). This mesocone elongation looks something exclusive of this subgroup of *Anthinus* amongst the known strophocheilids. An additional character is easily differentiating the rachidian tooth and its neighbors; the species with short mesocone, the differentiation between both is easy, while those with elongated mesocone, the rachidian is very similar to the neighboring teeth, being of difficult differentiation; from those, *A. synchondrus* and *A. vailanti* with certain effort it is possible to point out which is the rachidian column; however, this is very difficult in *A. morenus* and *A. savanicus*. Another interesting feature of the examined species is the posterior position of the anterior duct to the digestive gland, called the esophageal duct, which connects to the anterior lobe of the digestive gland. This esophageal duct originates practically from the stomach, while at least in *Megalobulimus*, it is far from the stomach; this feature is not known in the other strophocheilid genera. Beyond the odd position of the esophageal duct to digestive gland, there is the strong bifurcation of it, i.e., it bifurcates shortly after its origin. This characteristic is found both, in *Catracca* and in *Anthinus*, being only different in size; both branches have relatively similar caliber in *Anthinus* (e.g., Fig. 6C: dr, dl), while the left branch is narrower in *Catracca*; this does not occur at least in *Megalobulimus*, but this is unknown in other related genera.

The genital system usually is very informative in heterobranchs, but it has shown relatively conservative in strophocheilids, differing only in small details reported below. The presence of the supposedly exclusive megalobulimine accessory genital gland in spermoviduct (e.g., Figs. 8D, F: as) of the *Anthinus* is extraordinary. The seminal receptacle offers some distinction of the examined species, being balloon-like in *Catracca*, as well in megalobulimines [5], and strongly curved in *Anthinus*; however, the seminal receptacle is amply, widely curved in *A. vailanti* (Fig. 10B: sr) and *A. multicolor* (Fig. 4B); while it is tightly curved in *A. synchondrus* (Fig. 8C), *A. morenus* (Fig. 14E), and *A. savanicus* (Fig. 19A). The insertion of the hermaphrodite duct (hd) in the seminal receptacle is in its base in all studied species, except for *A. vailanti*, in which it is inserted in the middle of its concave edge; this insertion is strongly curved in *C. uhlei*, *A. savanicus* and *A. multicolor*, while it is straight, rather perpendicular in *A. synchondrus*, *A. vailanti* and *A. morenus*. The penis relative size is ~80% of the spermoviduct in *A. savanicus* (Fig. 17C: pe), ~60% in *A. synchondrus* (Fig. 8B) and *A. vailanti* (Fig. 10C), and ~50% in *C. uhlei* (Fig. 23C), *A. morenus* (Fig. 14D), *A. multicolor* (Fig. 2C), and *A. miersi* [7]. The accessory genital gland in the spermoviduct is relatively small in *A savanicus* (Fig. 4B: as) and *A. multicolor*, while it is large in the other three species. The number and form of the spermgrooves in the spermoviduct is also variable according to the species; they are double and similar-sized in *C. uhlei* (Fig. 23E: sp) and *A. multicolor* (Fig. 4E), they are also double, but asymmetrical in *A. synchondrus* (Fig. 8F), it is single and with a spiral fold in *A. vailanti* (Fig. 10D), they are double but of different thickness in *A. morenus* (Fig. 14F), they are double, but one of them is T-shaped in *A. savanicus* (Fig. 17D). In the vaginal-free oviduct cavity, *C. uhlei* has a double vaginal fold (Fig. 4F: vf), while *A. multicolor* has a hump (Fig. 4C: hp); the internal pattern of folds is different in each species, and are schematized in their respective drawings (Figs. 4A, 8D, 12A, 19B). The insertion of the penis muscle shows also interesting distinctions, it is only inserted in the penis wall in *C. uhlei* (Fig. 24A: pm), it has two small branches additionally connected to epiphallus in *A. synchondrus* (Fig. 8B), and *A. vailanti* (Fig. 10C), it has a larger component connected to epiphallus in *A. morenus* (Fig. 14D), it has a single and broad branch to epiphallus in *A. savanicus* (Fig. 17C), and it has a small branch connected to epiphallus and another to vas deferens in *A. multicolor* (Figs. 4C-D). As told above, all *Anthinus* have a papillae cover in epiphallus lumen, but the papillae are small and uniform in *A. synchondrus* (Fig. 8E), of medium size in *A. vailanti* (Fig. 12B), and tall in *A. morenus* (Fig. 14C), *A. savanicus* (Fig. 19C) and *A. multicolor* (Fig. 4D); additionally, the papillae extend further along the penial chamber in *A. multicolor*, and along an adjacent area in *A. vailanti*; besides, the papillae of *A. multicolor* are not uniform. The internal pattern of penial folds is also distinctive, *A. morenus* is the only species in having a single and simple large posterior fold (Fig. 14C: pf); all remaining *Anthinus* have a pair of parallel folds in a side: *A. synchondrus* has these folds composed of a series of transverse threads (Fig. 8E); *A. vailanti* is the only in having this pair fused in the proximal region (Fig. 12B); *A. savanicus* has this pair formed by the end of the secondary folds of the remaining penial inner surface (Fig. 19C); while *A. multicolor* has these folds wide and covered by papillae (Fig. 4D). Interestingly, this pair of folds has its intermediary furrow continuous with the aperture of the epiphallus. All these internal structures are exteriorized through copulation, including the complex structure of *Catracca* (Fig. 24B). To understand how they work during copula is a fascinating issue for future investigation.

Anatomical and broader taxonomical debate

As referred above, details of the some remaining strophocheilid genera are still unknown, and what is known is mostly focused in megalobulimines. This precludes further inferences on the intrinsic and extrinsic interrelationships and phylogeny of the taxa. Leme (e.g., [5, 10, 22]) had that as a main professional goal, but it remains unfinished. The intention is to find a character or a set of characters supporting each taxon, which, based on lack of studies, so far is impossible. Megalobuliminae, firstly introduced as a family [5] really looks like a

subdivision of Strophocheilidae, possessing the largest species (regarded as an anti-predatory strategy [30]), and supported at least by the pulmonary septum, buccal flange, pre-rectal septum in intestine, pre-rectal pallial muscle and accessory genital gland in the spermoviduct [5]. However, the presence of these two last characteristics in *Anthinus*, here presented, shows that these concepts must be reevaluated. Within strophocheilids, only *Megalobulimus* (so far the single genus of Megalobuliminae, with almost a hundred species), and *Anthinus* (this study) have samples with anatomical description sufficiently detailed for supporting more detailed comparative analyses like phylogeny. The other six genera [5, 6] are totally unknown or only have some few structures known.

The central nervous system (nerve ring) has shown relatively conservative in both genera studied here, all of them are similar (e.g., Fig. 24C), varying only in some small details and proportions, as reported above. At least in its main features, the nerve ring of the studied species is very similar to that of the megalobulimines [10] and even of the Bulimulidae (e.g., [31]).

A phenotypical base to understand the relationship of the Strophocheilidae with the other related families is also difficult for the same reason – lack of anatomical knowledge. At the time Strophocheilidae was only closely related to Dorcasiidae (African) and Acavidae (Asian) within Acavoidea [9], a group that apparently lays large eggs, produced by a thick walled, glandular spermoviduct, the phenotypic analysis was difficult. The scenario became more complicated with the present conception of these families as part of Rhytidoidea, with five additional families, based on molecular approaches [11, 12, 32]. The plan is to perform studies on other genera of all these families in a similar level of details of the present one, to produce a morphological database enough for a phylogenetic analysis. Only then a confrontation of the present taxonomy will be possible, and, possibly, to provide morphological bases for all subdivisions. The traditional Acavoidea above mentioned looks to be a morphologically well-supported taxon by above mentioned characters. However, the present Rhytidoidea still needs a better phenotypic support, as the fusion of Acavoidea with Rhytidoidea [11] is only based upon two papers with incongruent results, one of them did not have the Acavoidea as a focus [32], and another is an unpublished dissertation [33].

The Table 2 summarizes the main anatomic differences among the species studied herein.

Table 2. Summary of main anatomic differences among the species studied in this paper (all measures are approximations of average measures of several specimens)

species	Mantle edge folds	Reno-pericardial area	Pre-rectal pallial muscle	fusion of odontophore cartilages	Origin of m4 on cartilages	Jaw plate	Radula mesocone	Radular rachidian and neighboring	Seminal receptacle
Anthinus multicolor	rounded	small	absent	90%	50%	broad, thick	hook-like	similar	widely curved
Anthinus synchondrus	projected	large	present	100%	50%	narrow	Spatula-like	different	tightly curved
Anthinus vailanti	with pointed fold	medium	present	90%	80%	broad, central fold	Spatula-like	different	widely curved
Anthinus morenus	rounded	small	present	50%	40%	narrow	Spatula-like	similar	tightly curved
Anthinus savanicus	projected	large	present	85%	50%	broad	Spatula-like	similar	tightly curved
Catracca uhlei	projected	large	absent	50%	100%	Ticker at middle	hook-like	different	Baloon-like

species	Insertion of hermaphrodite duct	Accessory genital gland	Inner masculine folds	Free oviduct	Length penis x spermoviduct	Insertion of penis muscle	epiphallus papillae type	epiphallus papillae extension	penial inner folds
Anthinus multicolor	At base, curved	small	pair of similar - shaped	With distal hump	50%	Mostly in penis tip	tall, uneven	along entire penis chamber	wide and low, with papillae
Anthinus synchondrus	At base, perpendicular	large	pair of similar - shaped	usual	60%	Pair on epiphallus	Low, uniform	Only inside	Larger folds composed of transverse subfolds
Anthinus vailanti	At middle, perpendicular	large	Single, coiled	usual	60%	Pair on epiphallus	Medium, not uniform	Along penial chamber	Pair simple, part fused
Anthinus morenus	At base, perpendicular	large	Pair, thick	Usual	50%	Mostly on epiphallus	tall	Only inside	Single, large
Anthinus savanicus	At base, curved	small	Pair, one T-shaped	Usual	80%	Thick branch to epiphallus	tall	Only inside	Pair, large, secondary folds
Catracca uhlei	At base, curved	absent	Pair, thick	With genital fold	75%	Penis only	absent	__	Transverse septum

Phylogenetic analysis

Once more it is important to emphasize that the present phylogenetic analysis is not concerned with resolving the family Strophocheilidae, nor any other higher taxa. The main intention, using already published data, is to demonstrate that a new genus was necessary to be introduced, and the species does not fit in any of the known genera.

The computational processing of the Matrix (Appendix 2), which was based on the given list of 56 characters/126 states (Appendix 1) (see Material and Methods and [13]), resulted in a single cladogram (Fig. 28) with the following indices: length (L)= 283; consistency index (CI)= 68; retention index (RI)= 89.

In the cladogram (Fig. 28), the position of *Catracca uhlei* (after node 3) clearly shows that it does not belong to any known genera. It is recognized that some strophocheilid genera are not represented in the cladogram, but all of them (i.e., *Gonyostomus* Beck, 1837; *Speironepion* Bequaert, 1948; and the Andean *Austroborus* Parodiz, 1949 and *Chiliborus* Pilsbry, 1926) are beforehand reasonably different. The isolated position of *Catracca* in the base of Strophocheilidae (node 3), with two autapomorphies (character 7: *Megalobulimus oblongus*-like protoconch; character 52: transverse septum in the penis) is compatible with the generic separation.

Additionally, some few provisional conclusions are possible. The genus *Anthinus* resulted monophyletic (node 7), supported by six synapomorphies, such as the mosaic shell color (character 4); special arrangement of head muscles (ch. 11); the spatula-like radular mesocone (ch. 29), with rounded tip (ch. 30); the loss of the talon (ch. 42), and of the spermoviduct accessory gland (ch. 44). *Anthinus multicolor*, the type species, resulted as first branch, with two autapomorphies, and separated from the other congeners by three

autapomorphies. The other four *Anthinus* species introduced herein resulted in successive branches, with *A. vailanti* at base, and *A. synchondrus*–*A. morenus* together as terminals.

Other interesting provisional results are the monophyly of the South American Strophocheilidae (node 2 – 14 synapomorphies), separated from the African Dorcasiidae (node 15 – 3 synapomorphies). The dichotomy of the family into Megalobuliminae (node 11 – 15 synapomorphies) and Strophocheilinae (node 3 – 5 synapomorphies) is also corroborated. Also noticeable is the division of the megalobulimines, in which what Leme [5] informally called as "*M. ovatus* group" (node 14 – 1 synapomorphy) and *"M. oblongus* group (node 12 – 2 synapomorphies). These possibly can be an indicative of genetic separation, an issue that is in analysis.

The present provisional cladogram achieved its goals, mainly concerned to *Catracca* allocation, as well as shows that *Anthinus* is monophyletic and has a perceptible internal arrangement that can arouse future subgeneric divisions. The increment of this cladogram with more taxa is an in-progress project.

Ecologic-distribution information

The discovery of several new taxa in central regions of Brazil has indicated how weak the knowledge level on the Brazilian malacofauna is. With human activities, possibly species have been lost even before they were at least known. Several Brazilian land snails have shown to be endemic from small areas (personal observations), which is an important factor for conservation efforts and politics. The taxa described in this paper are examples that lots of Brazilian species and even genera still need to be discovered or better-known. Knowing the fauna is the first step to raise protective rules to them and their environments. Thus, their descriptions are relatively urgent.

The type localities of all *Anthinus* and *Catracca* are shown in the map of Fig. 27. In that it is possible to see that there is a concentration in the northwest region of Minas Gerais (MG) (and adjacent region of Goiás – GO), and another in Rio de Janeiro (RJ). The single type locality far from those is that of *A. albolabiatus*, in east of Rio Grande do Sul (RS). Reports to *Anthinus* in literature and collections are not common, but samples and citations of *Anthinus* have been collected from south of Minas Gerais to North Argentina and Uruguay, sometimes even identified at a specific level. As the areas in such the *Anthinus* samples were better studied – Rio de Janeiro and NW Minas Gerais (Fig. 27) show that several species with restricted distribution occur in relatively small areas, the question arises: are the *Anthinus* from the other regions belonging to undescribed species? As in the moment there is no possibility of checking this, no answer is possible.

Acknowledgements

A special thanks to José Coltro Jr and his team of collectors for the collect and donation of most studied samples of the present paper. To Lee Kremer for the thoughtful correction of the text. To Gary M. Barker and the other two anonymous referees, and the Editor for the comments, criticisms and improvements. To Lara Guimarães, MZSP, for the assistance in SEM examinations. The collects were performed under the IBAMA-Sisbio license 10560-2.

References

1. Albers JC. Die Heliceen, nach natürlicher Verwandtschaft. Verlag von Th.Chr.Fr. Enslin. Berlin, 262 pp; 1850.
2. Rang S. Descriptions des coquilles terrestres recueillies pedant un voyage à la côte occidentale d'Afrique, et au Brésil. Ann Sci Natur. 1831; 24: 1-63, pls. 1-3.
3. Martens Ev. Albers Die Heliceen, nach natürlicher Verwandtschaft (second edition). Verlag von Wilhelm Engelmann. Leipzig, 359 pp; 1860.
4. Bequaert JC. Monograph o the Strophocheilidae, a Neotropical family of terrestrial mollusks. Bull Mus Comp Zool. 1948, 100(1): 1–210, 32 pls.
5. Leme JLM. Anatomy and systematics of the Neotropical Strophocheiloidea (Gastropoda, Pulmonata) with the description of a new family. Arq Zool. 1973; 23(5): 295–337.
6. Simone LRL. Land and freshwater Molluscs of Brazil. São Paulo, EGB/ FAPESP; 2006.
7. Oliveira MP, Almeida EL, Vieira I, Oliveira MH. Comunicação n° 3: sobre o *Gonyostomus* multicolor miersii (Sowerby). Trab Setor Malac Univ Fed Juiz de Fora. 1968: 7-20.
8. Salvador RB, Colley E, Simone LRL. Terrestrial mollusks from the region of Corumbá and Maciço de Urucum, SW Brazil. J Conch. 2018; 43(1): 71–88.
9. Fontenelle JH, Miranda MS. Aspects of biology of *Megalobulimus paranaguensis* (Gastropoda, Acavoidea) in the coastal plain of the Brazilian southeast. Iheringia sér Zool. 2017; 107: e2017004; DOI: 10.1590/1678-4766e2017004.
10. Simone LRL, Leme JLM. Two new species of Megalobulimidae (Gastropoda, Strophocheiloidea) from north São Paulo, Brazil. Iheringia sér Zool. 1998; 85: 189-203.
11. Bouchet P, Rocroi JP, Hausdorf B, Kaim A, Kano Y, Nützel A, Parkhaev P, Schrödl M, Strong EE. Revised classification, nomenclator and typification of gastropod and monoplacophoran families. Malacologia. 2017; 61(1-2): 1–526.
12. MolluscaBase eds. MolluscaBase. Rhytidoidea Pilsbry, 1893. Website accessed at: http://www.molluscabase.org/aphia.php?p=taxdetails&id=890459 on 2021-02-10; 2021.
13. Simone LRL. Phylogeny of the Caenogastropoda (Mollusca), based on comparative morphology. Arq Zool. 2011; 42: 161–323.
14. Leme JLM, Castro RL, Indrusiak LF. Contribuição anatômica e histológica para o conhecimento de *Mirinaba antoninensis* (Morretes, 1952) (Gastropoda, Strophocheilidae). Pap Avul Zool. 1979; 32(14): 183–191.
15. Indrusiak LF, Leme JLM. Anatomia comparada de três espécies de *Mirinaba* Morretes, 1952 (Gastropoda, Strophocheilidae) do Estado do Paraná, Brasil. Acta Biol Paranaense 1985; 14: 163–180.
16. Leme JLM. *Megalobulimus lopesi* sp.n., uma nova espécie de Pulmonata terrestre da mata atlântica brasileira (Mollusca, Gastropoda, Megalobulimidae). Mem Inst Oswaldo Cruz 1989; 84(suppl. 4): 313–318.
17. Leme JLM, Indrusiak LF. *Megalobulimus parafragilior*, sp.n., uma nova espécie de Pulmonata terrestre da Serra do Mar (Gastropoda, Megalobulimidae). Pap Avul Zool 1990; 37(5): 97–105.
18. Leme JLM, Indrusiak F. Anatomia e considerações sobre *Megalobulimus proclivis* (Martens, 1888) (Gastropoda, Megalobulimidae). Iheringia (Zool) 1995; 78: 19–27.
19. Fontenelle JH, Simone LRL, Cavallari DC. *Megalobulimus dryades*, a new species from the Atlantic Forest in southeastern Brazil, and redescription of *Megalobulimus gummatus* (Gastropoda: Strophocheilidae). Pap Avul Zool 2021; 61: e20216144. Doi 10.11606/1807-0205/2021.61.44.
20. Silva FS, Simone LRL. Anatomy of two species of *Megalobulimus* (Strophocheilidae, Megalobuliminae) from Center-South of Bahia, Brazil. Pap Avul Zool (in press).
21. Simone LRL, Salvador RB. Taxonomical study on a sample of land snails from Nanuque (Minas Gerais, Brazil), with descriptions of three new species. Stutt Beit Naturk A 2016; N Ser 9: 9–30.

Formatted: English (United States)

22. Leme JLM. Sobre a posição taxonômica de *Dorcasia* e *Trigonephrus* (Gastropoda, Pulmonata). Pap Avul Zool. 1980; 34(2): 11–19.
23. Simone LRL. Bosquejos de filogenia. Clube de Autores. Curitiba, 60 pp. https://www.google.com.br/books/edition/Bosquejos_De_Filogenia/b5TtDwAAQBAJ?hl=pt-BR&gbpv=0
24. Pilsbry HA. American bulimi and bulimuli – *Strophocheilus, Plekocheilus*, Auris, *Bulimulus*. Man Conch.1895-1896; (2)10: 1-213, 51 pls.
25. Morretes FL. Ensaio de catálogo dos moluscos do Brasil. Arq Mus Paran. 1949; 7: 1–216.
26. Abbott RT. Compendium of landshells. American Malacologists. Hong Kong, 240 pp; 1989
27. Salgado NC, Coelho ACS. Moluscos terrestres do Brasil (gastrópodes operculados ou não, exclusive Veronicellidae, Miladicae e Limacidae). IN Barrientos, Z & Monge-Nájera, J. [ed.] Malacología Latinamericana. Rev Biol Trop. 2003; 51(suppl.3): 149–189.
28. Oliveira MP, Rezende GJR, Castro GA. Catálogo dos moluscos da Universidade Federal de Juiz de Fora. Sinonímia de família, gênero e espécie. MEC, UFJF. Juiz de Fora, 520 pp, 1981.
29. Rang S. Description des coquilles terrestres recucillies pendand un voyage à la côte occidentale d'Afrique, et au Brésil. Ann Sci Nat. 1831: 24: 5–63.
30. Simone LRL. Physical defense strategies of South American land snails. Malacopedia. 2018; 1: 3–11. http://www.moluscos.org/trabalhos/Malacopedia/01-02%20Simone%202018%20Physical%20strategies%20of%20defense.pdf
31. Simone LRL, Belz CE, Gernet MV. A new species of *Drymaeus* endemic from Currais Archipelago, Paraná, Brazil (Pulmonata, Bulimulidae). Pap Avul Zool. 2020; 60: e20206057. DOI 10.11606/1807-0205/2020.60.57.
32. Herbert DG, Moussalli A, Griffiths OL. Rhytididae (Eupulmonata) in Madagascar: reality or conjecture? J Moll Stud. 2015; 81(2): 259–268
33. Teasdele LC. Phylogenomics of the pulmonate land snails. PhD Thesis, University of Melbourne. 199 pp. http://hdl.handle.net/11343/128240; 2017.

Legends

Fig 1. *Anthinus multicolor*, shell and some anatomical structures, sample MZSP 152925. (A-C) shell of specimen #2 (L 30.2 mm), frontal, right and dorsal views. (D) same, detail of apex, apical-slightly dorsal views. (E) same, protoconch and part of first teleoconch whorl in profile. (F) penis, inner surface, detail of middle and distal thirds showing papillae. (G) jaw, ventral view. (H) same, anterior view. Scales= 1 mm.

Fig 2. *Anthinus multicolor*, anatomy. (A) pallial cavity, partially uncoiled visceral mass and digestive system, mostly ventral view, inner lip of pneumostome sectioned and deflected upwards. (B) head-foot, dorsal view, most of dorsal integument and inner organs removed, main concern to retractile/columelar musculature, some right-anterior structures sectioned. (C) genital system, mostly dorsal view. Scales = 3 mm.

Fig 3. *Anthinus multicolor*, radula in SEM, MZSP 152925. (A) wide view, scale= 200 µm; (B) detail of central region, scale= 50 µm; (C) same, higher magnification, scale= 30 µm. White arrows indicating rachidian column.

Fig 4. *Anthinus multicolor*, anatomy. (A) anterior half of genital tubes, opened longitudinally. (B) carrefour region of genitalia and posterior region of spermoviduct, albumen gland seen as transparent. (C) penis, ventral view. (D) same, opened longitudinally showing inner surface. (E) spermoviduct, transverse section in its middle region. Scales= 1 mm.

Fig 5. ***Anthinus synchondrus*****, shell of types and some anatomical photos**. (**A-F)** holotype MZSP 152074 shell (L 36.9 mm). (A) frontal view. (B) right view. (C) dorsal view. (D) anterior-slightly ventral view. (E) apical view (W 19.7 mm). (F) frontal view after extraction of specimen by an artificial hole. (G-H) paratype MZSP 152236, (L 40.8 mm), frontal and dorsal views. (I-J) 2 paratypes MZSP 152252 shell, frontal views (L 36.4, 36.2 mm). (K) holotype, detail of protoconch and part of first teleoconch whorl, profile. (L) same, detail of apical region. (M) jaw of holotype in situ, ventral view. (N) detail of right-anterior corner of pallial roof of holotype, ventral view, distal portion of rectum sectioned longitudinally. Scales= 1 mm.

Fig 6. ***Anthinus synchondrus*****, anatomy**. (A) pallial cavity roof, ventral view, pneumostome ventral lip sectioned and deflected upwards, portion of pallial floor also shown. (B) stomach opened longitudinally, ventral view. (C) digestive tubes mostly as in situ, ventral view, topology of some adjacent structures also shown. (D), odontophore, dorsal view, superficial layer of muscles and structures removed, left muscles deflected, radula deflected to left. Scales= 2 mm.

Fig 7. ***Anthinus synchondrus*****, radula in SEM**, holotype MZSP 152074. (A) wider view, scale= 200 µm; (B) detail of central region, scale= 200 µm; (C) same, higher magnification, scale= 50 µm. White arrows indicating rachidian column.

Fig 8. ***Anthinus synchondrus*****, anatomy**. (A) foregut opened longitudinally, ventral view, odontophore deflected to right. (B) genital system, mostly dorsal view. (C) same, detail of carrefour, ventral view, seen if albumen gland was transparent. (D) genital system, anterior end opened longitudinally, penis only partially shown. (E) penis and epiphallus opened longitudinally. (F) spermoviduct, transverse section in its middle level. Scales = 2 mm.

Fig 9. ***Anthinus vailanti*****, shells and jaw**. (A-E) holotype MZSP 152891 (L 29.0 mm). (A) frontal view. (B) right view. (C) dorsal view. (D) detail of apex, right-slightly apical view. (E) protoconch and adjacent teleoconch whorl, right-slightly apical view. (F-G) paratype MZSP 152188 (L 27.9 mm) frontal and dorsal views. (H-J) paratype MZSP 152244 (L 29.5). (H) frontal view. (I) dorsal view. (J) anterior-slightly left view showing umbilicus. (K) holotype's jaw plate, ventral view. (L) same, anterior view. Scales= 0.5 mm

Fig 10. ***Anthinus vailanti*****, anatomy**. (A) odontophore, dorsal view, superficial layer of muscles and structures removed, most muscles deflected, radular ribbon deflected to left. (B) middle region of genital system isolated, only topology of albumen gland shown. (C) genital system, mostly ventral view. (D) spermoviduct, transverse section in its middle level. Scales= 2 mm.

Fig 11. ***Anthinus vailanti*****, radulae in SEM**, Paratypes MZSP 152118, 2 specimens. (A) wider vision, scale= 200 µm; (B) detail of central region, scale= 50 µm; (C) wide left view, scale= 100 µm; (D) wide view of another specimen, scale= 200 µm; (E) detail of central region, scale= 50 µm; (F) wide left view, scale= 200 µm. White arrows indicating rachidian column.

Figs 12. ***Anthinus vailanti*****, anatomy**. (A) genital system, detail of anterior region opened longitudinally, penis and other adjacent structures only partially shown. (B) penis and epiphallus opened longitudinally. Scales= 2 mm.

Fig 13. ***Anthinus morenus*****, shell of some types and anatomy**. (A-H) Holotype MZSP 152891 (L 35.8 mm). (A) Frontal view. (B) Right view. (C) Dorsal view. (D) Anterior view (W 21.0 mm). (E) Apical view. (F) Detail of protoconch and fist teleoconch whorl, profile. (G) Same, higher magnification. (H) Same, detail of apex. (I-L) Paratype MZSP 152047#1. (I) Jaw, in situ, ventral view. (J) Shell, frontal view (L 34.8 mm). (K) Shell, dorsal view. (L) Pallial cavity, ventral view, dorsal part of mantle edge and deflected to right, part of mantle floor also shown. Scales= 2 mm.

Fig 14. *Anthinus morenus*, anatomy. (A) Head-foot, head, digestive and genital structures removed, dorsal integument removed, main concern to main head-foot musculature. (B) Odontophore, dorsal view, superficial layer of muscles and structures removed, most muscles deflected. (C) Penis, sectioned longitudinally to show inner surface. (D) Genital system, mostly ventral view. (E) Middle region of genital system isolated, only topology of albumen gland shown. (F) Spermoviduct, transverse section in its middle level. Scales= 2 mm.

Fig 15. *Anthinus morenus*, Radula in SEM, paratype MZSP 152047. (A) wide view, scale= 200 µm; (B) detail of central region, scale= 200 µm; (C) detail of damaged region, with teeth in profile, scale= 50 µm; (D) detail of right region, scale= 50 µm; (E) wide view, scale= 500 µm; (F) detail of left region, scale= 200 µm. White arrows indicating rachidian column.

Fig 16. *Anthinus savanicus*, shell of types and anatomical details. (A-H) holotype MZSP 154391 (L 32.5 mm). (A) shell, frontal view. (B) right view. (C) dorsal view. (D), apical view. (E) inferior (umbilical) view. (F) protoconch and part of first teleoconch, profile, scale= 2 mm. (G) jaw, ventral view (L 1.7 mm). (H) almost entire specimen, mostly dorsal view, pallial cavity removed and deflected right (ventral view), head-foot deflected below, visceral mass slightly uncoiled, scale= 5 mm. (I-J) paratype MZSP 153897 (L 31.5 mm), frontal and dorsal views. (K) paratype MZSP 152171 (L 35.3 mm), frontal view.

Fig 17. *Anthinus savanicus*, anatomical drawings. (A) midgut as in situ, ventral view, some adjacent structures or their topology also shown, stomach and esophagus opened longitudinally. (B) odontophore, dorsal view, outer layer of structures removed, pair of odontophore cartilages deflected, both m4 and left m5 (right in Fig.) also deflected. (C) genital structures, mostly ventral view, most vas deferens (dv) detached. (D) spermoviduct, transverse section of its middle level. Scales= 2 mm.

Fig 18. *Anthinus savanicus*, radulae in SEM. (A) holotype MZSP 154391. Detail of central region, scale= 50 µm; (B) same, detail of column in profile, scale= 50 µm; (C) same, detail of right region, scale= 200 µm; (D) paratype MZSP 153897, detail of right region, scale= 50 µm; (E) same, central region, scale= 100 µm. White arrows indicating rachidian column.

Fig 19. *Anthinus savanicus*, anatomy, genital system. (A) carrefour region, ventral view, seen if albumen gland (ag) was transparent, spermoviduct (eo) transversely sectioned in its posterior region; (B) anterior end, ventral view, entirely opened longitudinally, portions of adjacent structures also shown. (C) penis, ventral view, entirely opened longitudinally, portions of adjacent structures also shown. Scales= 2 mm.

Fig 20. *Catracca uhlei*, shells and jaw. (A-C) Holotype MZSP 151900 (L 44.2 mm). (A) Frontal view. (B) right view. (C) dorsal view. (D) Paratype MZSP 151901#1 (L 42.8 mm), frontal view; (E) holotype protoconch in profile, scale= 3 mm. (F) paratype 151893, shell of one of dissected specimens, frontal view (L 47.1 mm). (G) Paratype MZSP 151901#2 (L 43.0 mm). (H) Paratype MZSP 151871, young specimen, detail of apex, profile-slightly apical view, scale= 2 mm. (I) Same, apical view, scale= 2 mm. (J) Jaw plate, ventral view (slightly broken in left end, right in Fig.), scale= 1 mm.

Fig 21. *Catracca uhlei*, anatomy. (A) Pallial cavity roof, ventral view, pneumostome ventral lip sectioned and deflected upwards. (B) Head-foot, head, digestive and genital structures removed, anterior integument sectioned and deflected to left (right in Fig.), main concern to main head-foot musculature. (C) Fore- and midgut, mostly ventral view, shown as in situ, topology of some adjacent structures also shown. (D) Stomach, ventral view, mostly sectioned longitudinally to show inner surface. (E) Buccal mass, left view. Scales= 5 mm.

Fig 22. ***Catracca uhlei*****, radula in SEM**, holotype MZSP 151893. (A) wide view, scale= 300 µm; (B) detail of central region, scale= 100 µm; (C) detail of lateral region, scale= 100 µm; (D) detail of central region, scale= 50 µm; (E) same, higher magnification, scale= 30 µm; (F) same, higher magnification, scale= 10 µm. White arrows indicating rachidian column.

Fig 23. ***Catracca uhlei*****, anatomy**. (A) Odontophore, dorsal view, superficial layer of muscles and structures removed, left muscles deflected. (B) Buccal mass, ventral view, sectioned longitudinally, odontophore deflected to left. (C) Genital system, mostly ventral view. (D) Middle region of genital system isolated. (E) Spermoviduct, transverse section in its middle level. (F) Anterior region of genital system, ventral view, female portion sectioned longitudinally to show inner surface. Scales= 2 mm.

Fig 24. ***Catracca uhlei*****, anatomy**. (A) Penis, dorsal view. (B) Same, sectioned longitudinally to show inner surface. (C) Nerve ring, dorsal view. Scales= 2 mm.

Fig. 25. Gallery of shells of all ***Anthinus*** **species**, frontal and dorsal views. (A). *A. albolabiatus*, MZSP 7958 (L 50 mm). (B) *A. miersi*, MZSP 7963 (L 53 mm). (C) *A. multicolor*, MZSP 29620 (L 26 mm). (D) *A. turnix*, holotype USNM 712805 (L 50 mm). (E) *A. synchondrus* holotype MZSP 152074 (L 36.9 mm); (F) *A. vailanti* holotype MZSP 152891 (L 29.0 mm). (G) *A. morenus* holotype MZSP 152891 (L 35.8 mm). (H) *A. savanicus* holotype MZSP 154391 (L 32.5 mm). (I) *Catracca uhlei* holotype MZSP 151900 (L 44.2 mm). A to D extracted from Simone (2006).

Fig. 26. Gallery of shells of all ***Anthinus*** **species in same scale**, frontal views. (A). *A. albolabiatus*, MZSP 7958. (B) *A. miersi*, MZSP 7963. (C) *A. multicolor*, MZSP 29620. (D) *A. turnix*, holotype USNM 712805. (E) *A. synchondrus* holotype MZSP 152074. (F) *A. vailanti* holotype MZSP 152891. (G) *A. morenus* holotype MZSP 152891. (H) *A. savanicus* holotype MZSP 154391. (I) *Catracca uhlei* holotype MZSP. A to D extracted from Simone (2006). Scale= 10 mm. – verificar citação

Fig. 27. Brazil map with states outlined and type localities of each *Anthinus* (*A.*) and *Catracca* shown. New localities in dark blue. Previous localities in light blue. States abbreviations: GO: Goiás; MG: Minas Gerais; RJ: Rio de Janeiro; RS: Rio Grande do Sul.

Fig. 28. Single cladogram obtained analyzing the matrix (Appendix 2) of the species assembly reported in Material and Methods (written black), plus the species studied herein (written brown). Both branches below are far outgroups, a bulimulid (*Leiostracus*) and a simpulopsid (*Rhinus*). Branch *15* as close outgroups, two African dorcasiids. Each branch with synapomorphies shown. Each symbol as in the box; above number the character, below number the state. Branch numbers for discussion in blue italics. Method: parsimony; L= 283; CI= 68; RI= 89.

Formatted: Highlight

Formatted: Highlight

Formatted: Highlight

Appendix 1 – list of characters that base the phylogenetic analysis

1. Shell walls: 0= thin; 1= thick.
2. Shell form: 0= bulimoid; 1= discoid.
3. Shell size: 0= 2-3 cm; 1= 4-5 cm; 2= over 6 cm.
4. Shell color: 0= monochromatic; 1= mosaic of spots.
5. Periostracum: 0= permanent; 1= deciduous.
6. Aperture: 0= smaller than half of shell length; 1= longer than half of shell length.
7. Protoconch sculpture: 0= smooth or reticulate; 1= reticulate with axial predominance; 2= irregular axial threads; 3 regular narrow axial cords.
8. Protoconch form: 0= pointed; 1= blunt.
9. Head peri-oral flange: 0= absent; 1= present.
10. Haemocoel layers of head rectractor muscles: 0= two; 1= single.
11. Inner layer of head retractors: 0= only present in ommatophores; 1= ommatophores and peribuccals separated; 2= ommatophore and peribuccal fused.
12. Mantle edge inner pneumostome fold: 0= simple; 1- angulous.
13. Differentiated vessel close to pneumostome: 0= absent; 1= present.
14. Afferent pallial pulmonary vessels: 0= single; 1= a pair.
15. Pulmonary septum: 0= absent; 1= present.
16. Nephropore: 0= protected in primary ureter; 1= exposed in pallial cavity.
17. Nephropore: 0= simple; 1= protected by anterior renal fold (? in outgroup).
18. Kidney: 0= smooth; 1= covered by anastomosed net of vessels.
19. Urinary pallial way: 0=via duct; 1= via groove.
20. Ureter groove: 0= smooth; 1= transversely folded.
21. Jaw plate: 0= smooth; 1= transversely folded; 2: narrow.
22. Horizontal muscle (m6): 0= present; 1= absent.
23. Insertion of pair m5: 0= on pair m4; 1= partially on cartilages.
24. Pair of muscles inside radular sac (m7): 0= present; 1= absent.
25. Inner edge of cartilages (anterior to fusion): 0= thick; 1= thin.
26. Radular teeth: 0= rachidial similar to lateral; 1= different.
27. Radular teeth: 0= bi- or multicuspid; 1= unicuspid.
28. Radular mesocone: 0= smaller than base; 1= as long as base; 2= longer than base.
29. Radular mesocone: 0= stubby; 1= flattened, spatula-like.
30. Radular mesocone tip: 0= pointed; 1= rounded.
31. Tissue on radula at end of radular sac (to): 0= simple; 1=reinforced.
32. Salivary duct between salivary gland and buccal mass: 0= long; 1= short.
33. Esophageal duct to anterior lobe of digestive gland: 0= far from stomach; 1= close to stomach.
34. Esophageal duct to anterior lobe of digestive gland: 0= single; 1= closely bifid
35. Stomach: 0= non-muscular; 1= weakly muscular; 2= highly muscular; 3= high muscular connected to typhlosole (add).
36. Stomach inner folds in its smaller curve: 0= absent; 1= present; 2= extending to remaining stomach (add).
37. Pre-rectal valve: 0= absent; 1= present.
38. Pre-rectal pallial muscle: 0= absent; 1= present.
39. Anus: 0= turned outside; 1= opening inside pallial cavity.
40. Seminal receptacle: 0= balloon-like; 1= U-shaped.
41. U-shaped seminal receptacle: 0= widely curved; 1- tightly curved.

42. Talon (swollen end of hermaphroditic duct) in carrefour; 0= absent; 1= present.
43. Duct of albumen gland: 0= connected in spermoviduct; 1= connected directly to albumen chamber.
44. Spermoviduct accessory gland: 0= absent; 1= small; 2= large.
45. Prostate: 0= narrow (~1/3 of diameter); 1= wide (~1/2).
46. Vaginal appendix: 0= absent; 1= present.
47. Epiphallus: 0= conic, long; 1= short, rounded; 2= pseudo-epiphallus; 3= conic, short.
48. Flagellum: 0= absent; 1= single and small.
49. Penis shield: 0= present; 1= absent.
50. Penis retractor muscle: 0= simple; 1= multiple.
51. Penis retractor muscle insertion: 0= in penis tip only; 1= branches in epiphallus.
52. Penis with transverse fold ("penial papilla"): 0= absent; 1= present, simple; 2= with sphincter.
53. Pair of parallel inner folds connecting epiphallus and penis: 0= absent; 1= present.
54. Penis inner surface: 0= smooth; 1= papillate; 2= with large longitudinal fold in a side (pilar).
55. Spawn: 0= minute and numerous capsules; 1= few, large capsules.
56. Capsules cover: 0= thin, weakly calcified; 1= thick, well-calcified (egg like).

Appendix 2 – matrix of characters that base the phylogenetic analysis

Species\ character	1	2	3	4	5	5
	1234567890	1234567890	1234567890	1234567890	1234567890	123456
Mirinaba antoninensis	1010011000	1100011010	0111101100	0111110001	?100001011	111111
Mirinaba cadeadensis	1010011000	1100011010	0111101100	0111110001	?100001011	111111
Megalobulimus lopesi	1020002111	0010110111	1101011100	1100221110	?102003010	000211
Megalobulimus grandis	1020012111	0010110111	1101011100	1100221110	?102001010	100211
Megalobulimus parafragilior	1020012111	0010110111	1101011100	1100221110	?102001010	100211
Megalobulimus proclivis	1020012111	0010110111	1101001100	1100321110	?002011010	000211
Megalobulimus oblongus	1020103111	0010110111	1101001100	1100321110	?002110110	000211
Megalobulimus conicus	1020103111	0010110111	1101001100	1100321110	?002110110	000211
Megalobulimus driades	1020003111	0010110111	1101001100	1100321110	?002110110	000211
Strophocheilus debilis	1010002000	0100011010	0111101100	0111110000	?101002010	010111
Catracca uhlei	1010003100	1100011011	0101001100	0111110000	?000002010	020111
Anthinus multicolor	1001001000	2000011010	0111101111	0111110001	0002001010	101111
Anthinus synchondrus	1011001000	2100011010	2111101211	0111110101	1012001010	101111
Anthinus vailanti	1010001000	2100011010	0111101211	0111110101	1002001010	101111
Anthinus morenus	1011001000	2100011010	2111101211	0111110101	1012001010	101111
Anthinus savanicus	1011001000	2100011010	0111101211	0111110101	1012001010	101111
Dorcasia akexandri	0100000000	0001011010	000?000000	0000010000	?002000000	000011
Trigonephrus porphyrostoma	0100?00000	0001011010	0????00000	0000010000	?0?2000000	000011
Leiostracus carnavalescus	0000000000	0000000000	0000000000	0000000000	?000000000	000000
Rhinus botocudus	0000000000	0000000000	0000000000	0000000000	?000000000	000000

PLOS ONE <em@editorialmanageAnswer:com>

7 de maAnswer: de 2022 13:02 (há 7 dias)

para mim

PONE-D-22-00958
Additions to the genus Anthinus occurring in Minas Gerais and Goiás regions, Brazil, with description of five new species, one of them in the new related genus Catracca (Gastropoda, Eupulmonata, Strophocheilidae).
PLOS ONE

Dear D: Simone,

Thank you for submitting your manuscript to PLOS ONE. After careful consideration, we feel that it has merit but does not fully meet PLOS ONE's publication criteria as it currently stands. Therefore, we invite you to submit a revised version of the manuscript that addresses the points raised during the review process.

Please submit your revised manuscript by Apr 21 2022 11:59PM. If you will need more time than this to complete your revisions, please reply to this message or contact the journal office at plosone@plos.org. When you're ready to submit your revision, log on to https://www.editorialmanageAnswer:com/pone/ and select the 'Submissions Needing Revision' folder to locate your manuscript file.

Please include the following items when submitting your revised manuscript:

- A rebuttal letter that responds to each point raised by the academic editor and reviewer(s). You should upload this letter as a separate file labeled 'Response to Reviewers'.
- A marked-up copy of your manuscript that highlights changes made to the original version. You should upload this as a separate file labeled 'Revised Manuscript with Track Changes'.
- An unmarked version of your revised paper without tracked changes. You should upload this as a separate file labeled 'Manuscript'.

If you would like to make changes to your financial disclosure, please include your updated statement in your cover letter. Guidelines for resubmitting your figure files are available below the reviewer comments at the end of this letter.

If applicable, we recommend that you deposit your laboratory protocols in protocols.io to enhance the reproducibility of your results. Protocols.io assigns your protocol its own identifier (DOI) so that it can be cited independently in the future. For instructions see: https://journals.plos.org/plosone/s/submission-guidelines#loc-laboratory-protocols. Additionally, PLOS ONE offers an option for publishing peer-reviewed Lab Protocol articles, which describe protocols hosted on protocols.io. Read more information on sharing protocols at https://plos.org/protocols?utm_medium=editorial-email&utm_source=authorletters&utm_campaign=protocols.

We look forward to receiving your revised manuscript.

Kind regards,

Michael Schubert
Academic Editor
PLOS ONE

Journal Requirements

1. When submitting your revision, we need you to address these additional requirements.

Please ensure that your manuscript meets PLOS ONE's style requirements, including those for file naming. The PLOS ONE style templates can be found at https://journals.plos.org/plosone/s/file?id=wjVg/PLOSOne_formatting_sample_main_body.pdf and https://journals.plos.org/plosone/s/file?id=ba62/PLOSOne_formatting_sample_title_authors_affiliations.pdf

2. We noted in your submission details that a portion of your manuscript may have been presented or published elsewhere.
(previous submission (PONE-D-21-27031) edited by Michael Schubert)

Please clarify whether this [conference proceeding or publication] was peer-reviewed and formally published. If this work was previously peer-reviewed and published, in the cover letter please provide the reason that this work does not constitute dual publication and should be included in the current manuscript.

ANSWER: I have not seen any part of the MS published elsewhere. I only used some already published data of some papers to help the phylogenetic analysis only. But this is a usual procedure.

3.Thank you for stating the following financial disclosure:
(No)

At this time, please address the following queries:

a) Please clarify the sources of funding (financial or material support) for your study. List the grants or organizations that supported your study, including funding received from your institution.

ANSWER: the study was only supported by my institution; in such I am employee. No additional funding exists. We do not use to put that in Acknowledgements, as looks dispensable.

b) State what role the funders took in the study. If the funders had no role in your study, please state: “The funders had no role in study design, data collection and analysis, decision to publish, or preparation of the manuscript.”

ANSWER: okay. Stated in M&M (paragraph 5).

c) If any authors received a salary from any of your funders, please state which authors and which funders.

ANSWER: not applicable.

d) If you did not receive any funding for this study, please state: “The authors received no specific funding for this work.”

ANSWER: okay. Stated in M&M (paragraph 5).

Please include your amended statements within your cover letter; we will change the online submission form on your behalf.

3. Thank you for stating the following in your Competing Interests section:
(NO authors have competing interests)

Please complete your Competing Interests on the online submission form to state any Competing Interests. If you have no competing interests, please state "The authors have declared that no competing interests exist.", as detailed online in our guide for authors at http://journals.plos.org/plosone/s/submit-now
This information should be included in your cover letter; we will change the online submission form on your behalf.

4.Please take this opportunity to be sure you have met all of our guidelines for new species. When publishing papers that describe a new botanical taxon, PLOS aims to comply with the requirements of the International Code of Nomenclature for algae, fungi, and plants (ICN). In association with the International Plant Names Index (IPNI), the following guidelines for publication in an online-only journal have been agreed such that any scientific botanical name published by us is considered effectively published under the rules of the Code. Please note that these guidelines differ from those for zoological nomenclature, and apply only to seed plants, ferns, and lycophytes.
Effective January 2012, "the description or diagnosis required for valid publication of the name of a new taxon" can be in either Latin or English. This does not affect the requirements for scientific names, which are still to be Latin.
Also effective January 2012, the electronic PDF represents a published work according to the ICN for algae, fungi, and plants. Therefore the new names contained in the electronic publication of a PLOS ONE article are effectively published under that Code from the electronic edition alone, so there is no longer any need to provide printed copies.
For proper registration of the new taxon, we require two specific statements to be included in your manuscript.
a) In the Methods section, include a sub-section called "Nomenclature" using the following wording:
The electronic version of this article in Portable Document Format (PDF) in a work with an ISSN or ISBN will represent a published work according to the International Code of Nomenclature for algae, fungi, and plants, and hence the new names contained

in the electronic publication of a PLOS ONE article are effectively published under that Code from the electronic edition alone, so there is no longer any need to provide printed copies.
In addition, new names contained in this work have been submitted to IPNI, from where they will be made available to the Global Names Index. The IPNI LSIDs can be resolved and the associated information viewed through any standard web browser by appending the LSID contained in this publication to the prefix http://ipni.org/. The online version of this work is archived and available from the following digital repositories: [INSERT NAMES OF DIGITAL REPOSITORIES WHERE ACCEPTED MANUSCRIPT WILL BE SUBMITTED (PubMed Central, LOCKSS etc)].
All PLOS ONE articles are deposited in PubMed Central and LOCKSS. If your institute, or those of your co-authors, has its own repository, we recommend that you also deposit the published online article there and include the name in your article.

b) In the Results section, the globally unique identifier (GUID), currently in the form of a Life Science Identifier (LSID), should be listed under the new species name, for example:
Solanum aspersum S.Knapp, sp. nov. [urn:lsid:ipni.org:names:77103633-1] Type: Colombia. Putumayo: vertiente oriental de la Cordillera, entre Sachamates y San Francisco de Sibundoy, 1600-1750 m, 30 Dec 1940, J. Cuatrecasas 11471 (holotype, COL; isotypes, F [F-1335119], US [US-1799731]).
PLOS ONE staff will contact IPNI to obtain the GUID (LSID) after your manuscript is accepted for publication, and this information will then be added to the manuscript at that time. You may indicate where the number or numbers will be added using XXXXX or an equivalent placeholder:
A complete explanation of our guidelines for publishing new species can be found on our website: http://www.plosone.org/static/guidelines#botanical

ANSWER: The present paper only deals with animal taxa, not botanic. For the ICZN, only the register in Zoobank http://www.zoobank.org/ is necessary for new taxa even in electronic (PDF) publications. This procedure was done in the present paper, and correspondent links are in Abstract and in each new taxon described.

5. We note that Figure 27 in your submission contain map images which may be copyrighted. All PLOS content is published under the Creative Commons Attribution License (CC BY 4.0), which means that the manuscript, images, and Supporting Information files will be freely available online, and any third party is permitted to access, download, copy, distribute, and use these materials in any way, even commercially, with proper attribution. For these reasons, we cannot publish previously copyrighted maps or satellite images created using proprietary data, such as Google software (Google Maps, Street View, and Earth). For more information, see our copyright guidelines: http://journals.plos.org/plosone/s/licenses-and-copyright.

We require you to either (1) present written permission from the copyright holder to publish these figures specifically under the CC BY 4.0 license, or (2) remove the figures from your submission:

a. You may seek permission from the original copyright holder of Figure 27 to publish the content specifically under the CC BY 4.0 license.

We recommend that you contact the original copyright holder with the Content Permission Form (http://journals.plos.org/plosone/s/file?id=7c09/content-permission-form.pdf) and the following text:
“I request permission for the open-access journal PLOS ONE to publish XXX under the Creative Commons Attribution License (CCAL) CC BY 4.0 (http://creativecommons.org/licenses/by/4.0/). Please be aware that this license allows unrestricted use and distribution, even commercially, by third parties. Please reply and provide explicit written permission to publish XXX under a CC BY license and complete the attached form.”

Please upload the completed Content Permission Form or other proof of granted permissions as an ""Other"" file with your submission.

In the figure caption of the copyrighted figure, please include the following text: “Reprinted from [ref] under a CC BY license, with permission from [name of publisher], original copyright [original copyright year].”

b. If you are unable to obtain permission from the original copyright holder to publish these figures under the CC BY 4.0 license or if the copyright holder’s requirements are incompatible with the CC BY 4.0 license, please either i) remove the figure or ii) supply a replacement figure that complies with the CC BY 4.0 license. Please check copyright information on all replacement figures and update the figure caption with source information. If applicable, please specify in the figure caption text when a figure is similar but not identical to the original image and is therefore for illustrative purposes only.
The following resources for replacing copyrighted map figures may be helpful:

USGS National Map Viewer (public domain):
http://vieweAnswer:nationalmap.gov/viewer/
The Gateway to Astronaut Photography of Earth (public domain):
http://eol.jsc.nasa.gov/sseop/clickmap/
Maps at the CIA (public domain): https://www.cia.gov/library/publications/the-world-factbook/index.html and https://www.cia.gov/library/publications/cia-maps-publications/index.html
NASA Earth Observatory (public domain): http://earthobservatory.nasa.gov/
Landsat: http://landsat.visibleearth.nasa.gov/
USGS EROS (Earth Resources Observatory and Science (EROS) Center) (public domain): http://eros.usgs.gov/#
Natural Earth (public domain): http://www.naturalearthdata.com/

ANSWER: this Figure 27 is not copyrighted. I drew it by myself.

[Note: HTML markup is below. Please do not edit.]

Reviewers' comments:

Reviewer's Responses to Questions

Comments to the Author

1. Is the manuscript technically sound, and do the data support the conclusions?

The manuscript must describe a technically sound piece of scientific research with data that supports the conclusions. Experiments must have been conducted rigorously, with appropriate controls, replication, and sample sizes. The conclusions must be drawn appropriately based on the data presented.

Reviewer #1: Partly

Reviewer #2: Yes

Reviewer #3: Yes

---

2. Has the statistical analysis been performed appropriately and rigorously?

Reviewer #1: N/A

Reviewer #2: I Don't Know

Reviewer #3: Yes

---

3. Have the authors made all data underlying the findings in their manuscript fully available?

The PLOS Data policy requires authors to make all data underlying the findings described in their manuscript fully available without restriction, with rare exception (please refer to the Data Availability Statement in the manuscript PDF file). The data should be provided as part of the manuscript or its supporting information, or deposited to a public repository. For example, in addition to summary statistics, the data points behind means, medians and variance measures should be available. If there are restrictions on publicly sharing data—e.g. participant privacy or use of data from a third party—those must be specified.

Reviewer #1: Yes

Reviewer #2: Yes

Reviewer #3: Yes

---

4. Is the manuscript presented in an intelligible fashion and written in standard English?

PLOS ONE does not copyedit accepted manuscripts, so the language in submitted

articles must be clear, correct, and unambiguous. Any typographical or grammatical errors should be corrected at revision, so please note any specific errors here.

Reviewer #1: Yes

Reviewer #2: Yes

Reviewer #3: Yes

---

5. Review Comments to the Author

Please use the space provided to explain your answers to the questions above. You may also include additional comments for the author, including concerns about dual publication, research ethics, or publication ethics. (Please upload your review as an attachment if it exceeds 20,000 characters)

Reviewer #1: I have read the manuscript and the reviews with the Author's comments. The manuscript arouse a lively discussion between the Author and the Reviewers, the tension between them was clearly visible. Therefore, I hope my opinion will be helpful. After I read the manuscript, I had an impression it should be sent (after improvements mentioned below) rather to some purely taxonomic journal more than to PLOSone. I also think, the paper has rather poor chances to be cited. The Author has 165 papers indexed by SCOPUS, which is a huge number, of course. However, the h-index is 15. It is not much for such a numerous bibliography (sorry to rise this issue, but it was mentioned in the comments that the Author published 300 papers therefore I decided to check the indexing).

ANSWER: I am very grateful for the worries about my low Scopus H index. I am worried too, as much that I decided to publish in higher impact journals, like Plos-One in recent years. I have managed to do this and have recently published, for example, in Plos One itself, Cladistics, Zoological Journal, etc. (check in link below).

In respect to the previous submission, I think we must argue in scientific and polite ways in every criticism. If some clear tension was visible, I am sorry. That was not my intention.

The Scopus database is not the best one to be used in Zoology, it underestimates the zoologist's production. As demonstrated by the present referee, Taxonomy does not enjoy the highest prestige, thus, malaco-taxonomists use to publish in journals that are weakly covered by Scopus. This diminishes the Scopus' H index, but that does not necessarily indicate a bad or low-quality production. As it can be checked in http://lattes.cnpq.br/5020466945403853, I am the author of 274 papers published in peer-reviewed journals, 4 books, 38 book chapters, 50 popular papers, etc. Counting only these categories, I am the author of 366 published items. My H-index, for example, in Google is 22, and i10 index is 72. I recognize these are low if compared to applied areas like Medicine, Engineering, etc. But, for a fairer trial, they must be compared to other zoologists. In this scenario, I am far above average.

I think an explanation on the importance of the Taxonomy in biological areas, as it is at the base of any wildlife studies, is not necessary here. But it is important to emphasize that the present paper is not purely taxonomic. It also has lots of information and discussion on morphology, anatomy, biogeography, phylogeny, environmental issues, etc. All of them increase the chance of citations, as are almost inedit. The papers that go beyond the taxonomy difficultly are accepted in taxonomic journals, and are more indicated to journals on broader subjects, like Plos'.

In addition, to underestimate someone else's scientific field, his production, and to check his H-index in a report on his paper is not usual. This is hard to interpret except as a personal problem.

Graphs and photos are nicely done. I think the Author put a lot of effort to prepare the documentary. However, in my opinion, the "scale" problem was not solved properly. I do agree with the Reviewer that the scale is needed and helpful for a potential reader, and does not disturb the plates' aesthetics (which is a secondary issue, anyway). However, drawing just a line in on the graph and providing in the figure caption that e.g. "Scales= 2 mm" is vague. It would be better to provide a number next to the line in the graph, with additional information about the unit in the caption e.g. "Scale bars in mm". This is of course just a small point, which is easy to be solved.

ANSWER: I understand the present suggestion and I can attend it if the Editors also agree with it. However, as only this referee suggested that, and as do not put scale bars in images of entire shell is traditional in Malacology goo papers (they are put only in details, and in anatomical stuff, as done in present paper), I ask permission to maintain the plates as they are. I understand that scales in all images with a number on them have their advantages, but they are terrible for the esthetic of the plate. And esthetic and beauty of the illustrations are equally important, mainly in a wide journal like Plos. I emphasize that nothing is missing in present way of presentation, as all pertinent information are in the legends.

I also think, the Author should provide specification of the optical equipment used for all measurements and photographic documentation.

ANSWER: Done (beginning of paragraph of "Methods and scope").

About the molecular analyses – I also agree with the Reviewer that such the study should be done and presented. The Author mentioned in the Introduction that molecular analyses are scheduled for the future. Such the statement indicate that the study are not finished. Molecular analyses may bring some interesting results changing the currently presented results. I would rather recommend to perform the analyses and presented them with conchological and anatomical approach in a single manuscript.

ANSWER: The present referee agrees that additional molecular approach is worthy, but the other two referees do not, the Reviewer 2, for example, clearly stated that "I do not feel molecular work is necessary for new species descriptions (at least in molluscs)", and the Reviewer 3 neither comment on that. Thus, is there an antagonism between the two opinions, with 2x1 score.

I agree that the inclusion of molecular results would give more data for discussions, as well as any other myriad of additional approaches that could be added (e.g., histological, physiological, ecological, etc.). However, we, I think, need to define the scope of every paper and show the best scenario and result based on that. As the Science progresses, other approaches and scenarios will be added, and the conclusions can or cannot change.

Thus, I ask for permission to maintain the paper based "only" on conchology, anatomy, morphology, biogeography and phylogeny of these taxa, expressing the highest respect to the Reviewer 1 opinion. I repeat that most (more than 90%) of the molluscan taxa were defined only by their shells, and the present paper goes further beyond that.

I also feel that providing an information that "Shell about 40 mm" is too general.

ANSWER: Malacologists usually apply a still more general way, calling the shell "large", "medium" or "small", which is further subjective. This way, putting an approximate average size, I think is more scientific. Additional explanations are in M&M.

The Author should prepare a summarizing table (or two tables – one for conchological traits, the second – for anatomical traits) containing the number of shells and specimens analyzed, with the measurements range for each of the characteristics, mean value, standard deviation, as well as measurements for a holotype. Such the table would made easier to find the data about the number of shell measured and specimens dissected, as the information is, indeed, lost in the text.

ANSWER: Done.

The Author did not provide a number of dissected snails for all the species. In the main text I found the information on 9 specimens of A. multicolor and 2 specimens of C. uhlei, while figures containing anatomical characteristics are given for A. multicolor, A. synchondrus, A. vailanti, A. morenus, A. savanticus, and C. uhlei (In the caption of the Fig. 11 the Author mentioned that he used 2 specimens, but no data is available within the main document).

ANSWER: This is not totally accurate. A complete list of "Material examined" follow all descriptions. It has all examined "specimens" (i.e., shell + dissected) or "shells" of every species. In the new species, the lists include the type material, as indicated. The list of material examined are the examined assembly of specimens.

Overall recommendation: Although the Author put lot of effort to prepare the manuscript, I cannot recommend the paper to be published in PLOSone. In my opinion, the material presented is more adequate for some purely taxonomic journal. Apart of this suggestion, I think the data are too scanty - molecular analyses pending. The data are presented vaguely. I also feel that some summarization of all measurements, containing data on all material analyzed is extremely needed.

ANSWER: Everything attended.

Reviewer #2: Dear author and editor - I would suggest this paper is accepted for review with some very minor revisions.

The paper is a well written description of the new species and an important addition to our knowledge of Molluscs from this group/region. I have made a few suggestions to the text to make the article clearer to the readers and there are a few points I have asked for clarification where the meaning is not clear to me. Unlike previous reviewers I do not feel molecular work is necessary for new species descriptions (at least in molluscs) where there is a history and ongoing precedent of species descriptions based on shell only let alone anatomical characters. I thought the phylogenetic work was a nice addition to the paper.

The species descriptions are done to a high standard and are easily understandable to the malacological community. May I suggest a short (2-3 sentences) 'differential diagnosis' added to the end of each description to succinctly allow this species differences to be highlighted.

I feel that the discussion is very thorough but my feeling is it is overly long and complex and by adding some 'differential diagnosis' to the species and generic descriptions this could be condensed and simplified.
ANSWER: All species already have the initial item “diagnoses”, which brings the set of character that effectively differentiates the taxon from the others. All worthy characters are also reported in the Discussion, presented in a comparative scenario. I ask if would another item “differential diagnosis” put at end of the description be redundant? This is not a negative. If my argumentation is not convincing, I can easily obey this suggestion.

In the 'specific debate' there is a lot of shell information which whilst useful is difficult to follow. I wonder if a table showing data such as aperture size, W/L index and spire angle etc. could be used to shorten and simplify this?
Possibly more difficult but again could a table be used to highlight anatomical differences to shorten/simply or even add to the data presented.
ANSWER: done

I found the 'anatomical and broader taxonomic' section very informative and well structured.
ANSWER: Thanks.
I am not a phylogenetic specialist but the addition of this data was interesting and understandable to me.
ANSWER: Thanks.
The references are well structured and wide spread and the figures are well produced and clearly understandable. I do prefer scale bars in figures but I understand that when so many images are made available it would not be applicable in such a layout without it appearing messy.
ANSWER: Thanks.
If the author or editor have any further questions I am happy to contacted.

Reviewer #3: The revised ms is much improved, and has greatly benefited from inclusion of the anatomical data for the type species of Anthinus.
ANSWER: Thanks.

No page numbering on ms. Main comments refer to sections of text identified by numbers pencilled in the left margin of the annotated ms.
ANSWER: Sorry. Page numbers inserted.
Abstract
1. “A bulimulid, a simpulopsid and two dorcasiids are outgroups.”
2. “Mirinaba and Anthinus also resulted monophyletic (3 and 6 synapomorphies respectively.”
Suggest changing to “Mirinaba and Anthinus were also supported as monophyletic (3 and 6 synapomorphies respectively).
ANSWER: Done.
Introduction
3. “The description of the type species is, the remaining descriptions and illustrations, to minimize redundancy, are mostly presented compared to it.”
Sentence ambiguous. Re-work.
ANSWER: Done.
4. “The anatomical base of all of them will certainly be useful for future taxonomic revisions and for phylogenetic inferences.”
Suggest modifying as
The anatomical information presented on these new taxa will certainly be useful for future taxonomic revisions and for phylogenetic inferences.
ANSWER: Done.
5. “Despite these aspects are part of a large revisional project in such this study is inserted, an initial phylogenetic treatment is provided in this paper, based on the species with anatomy more detailly known in the literature and in the herein studied species. The main intention is not to present a “phylogeny of the Strophocheilidae”, as many more species would be necessary, but to provide a better scenario for a discussion and the base for the new taxa.
Suggest the following changes
Despite these aspects are part of a large revisional project in such this study is inserted, An initial phylogenetic treatment is provided in this paper, based on species for which anatomy is reasonably well known in the literature, coupled with new information on the species herein erected. The main intention is not to present a “phylogeny of the Strophocheilidae”, as anatomical investigation many more species would be necessary, but as the basis for erection of the new taxa, and to provide a better scenario for a comparative discussion.
ANSWER: Done.

Materials and methods

6. “The anatomical drawings are an average of all examined specimens. Color photos were obtained by digital cameras, either hand-held or attached to the dissecting microscope. Shell measurements were obtained with a digital caliper, in a minimum of 10 adult shells. The specimens were dissected by standard techniques [13] under dissecting stereomicroscopes, with the specimen immersed under the fixative. All drawings were obtained with the aid of a camera lucida; initially pencilled, afterwards inked; usually drawings produced for each species include data derived from several specimens. The type and voucher….”
Suggest a slight restructure of paragraph
Color photos were obtained by digital cameras, either hand-held or attached to the

dissecting microscope. Shell measurements were obtained with a digital caliper, for a minimum of 10 adult shells. The specimens were dissected by standard techniques [13] under dissecting stereomicroscopes, with the specimen immersed under the fixative. All drawings were obtained with the aid of a camera lucida; initially pencilled, afterwards inked; usually drawings produced for each species include data derived from several specimens. Thus, the anatomical drawings are an average (composite) of all examined specimens. The type and voucher ….
ANSWER: Done.
There is a potential issue arising with presentation composite illustrations rather than illustrating data gathered from individual specimens. Potentially information is lost on variation (if any) among the examined specimens. As a consequence, if there was variation between the examined type specimen [the name bearing specimen sensu Code of Zoological Nomenclature] and other examined specimens [non-name-bearing paratypes, topotypes, etc] then it may be ambiguous as to whether the composite encompasses anatomical information drawn from more than one species, such as can occur if one or more specimens were misidentified as belonging to the species in question. Is ambiguity can only be reduced if there is illustration of anatomical features for all examined specimens, or if the author explicitly states there was insignificant or no variation among these specimens.
Where only non-name-bearing specimens are examined (e.g. paratypes, topotypes, etc) then there will always be uncertainty as to whether this information also pertains to the species as defined by the name-bearing holotype.

ANSWER: This is a very interesting argumentation, which made me think. However, the anatomical illustrations, as well as the description/diagnosis, etc., are related to the SPECIES, and not of a specific specimen. Of course, some authors use to state “description of the holotype” as a formal species’ description. I do not apply that way as it is in disuse. I prefer to show an average character referent to the species, based on all specimens, in descriptions and illustrations, rather than simply show the exact features of the holotype. For sure, any important variation must also be reported, as well as, in that case, which variation is the holotype’s one. I do report these cases in my papers, but in the present paper the anatomical variation among the specimens can be classified as “insignificant”. I introduced this information in M&M (line 12).

7. “… The collector team works for a company called Femorale [www.femorale.com; http://www.femorale.com/femorale/index.asp]. The places of collection are not protected areas, which do not require special permits. The collections, anyway, are supported by the general/permanent license IBAMA-Sisbio 10560-2, which permits extraction of wildlife samples for scientific purposes. ….”
Suggest the following:
The material studied and described in this work was collected by a team working for Femorale, a private company [www.femorale.com; http://www.femorale.com/femorale/index.asp]. The places of collection are not within protected areas, and as such collection activity did not require special permits. Nonetheless, the collections were made under general/permanent license IBAMA-Sisbio 10560-2, which permits extraction of wildlife samples for scientific purposes.
ANSWER: Done.

8. “Thus, details on vegetation, climate, soil, rainfall, etc., were not available, but, when relevant, these data were extracted from the literature or official websites…”

Suggest
Thus, details on vegetation, climate, soil, rainfall, etc., were not available, but, when possible and relevant, these data were extracted from the literature, digital online resources, or official websites.
ANSWER: Done.

9. “For comparison of the presently studied species with those already known, the large MZSP collection was consulted, as a previous curator, DAnswer: José L.M. Leme, was a strophocheilid specialist.”
Suggest
For comparison of the presently studied species with those already known, the large MZSP collection was consulted, including the material assembled by DAnswer: José L.M. Leme, a previous curator and strophocheilid specialist.
ANSWER: Done.

10. “Although additional approaches can be added to any taxonomical paper, such as multivariate statistical analyses of shell measurements, molecular sequencing, etc., the presently presented anatomical investigation was so informative and convincing that it already bases very well the taxonomic conclusions. Of course, other approaches, molecular in particular, will be explored in future papers, considering an already well-based taxa provided by the present papeAnswer: It is important to emphasize that most of the land snails taxonomy is based only on their shells alone; the present paper goes further and presents convincing anatomical descriptions.”

This text has been added following adverse comments from previous reviewers, and is the author’s attempt to justify the methodology and pre-empt any future commentary. This text added by the author is unnecessary, redundant, as the published work should stand on its own merits.
ANSWER: Deleted.

11. “The chosen model to do the taxonomic remarks on each described species is reuniting all of them in a single Discussion, which ends this papeAnswer: This is interpreted as a more intelligible, fluent, and economic manner to explore the distinctions and similarities of the addressed taxa. Rather than individual remarks after each description.”
This text too was added by the author as a response to comments from previous reviewers. The criticism was not directed as the author’s model to aggregate the comparative remarks in the Discussion, but in the way that aggregation was done – too verbose, clumsy presentation of comparative morphometric information, lack of clarity about diagnostic features useful in species discrimination and identification, ….
I think this text in the main is redundant and should be replaced by a single sentence stating the fact that the taxonomic remarks are aggregated into the Discussion.
ANSWER: Ok. I redid that phrase for a simpler one. It is important to emphasize that all species bear a “Diagnosis” item, which already informs “about diagnostic features useful in species discrimination and identification”. The remaining parts of the paper are more concerned to discussion in taxonomic, comparative morphologic, and phylogenetic levels. These parts look verbose in a first view, but, as any scientific text, they must be complete, informative and exhaustive involving the literature.

12. “Phylogenetic analysis. In a family such as Strophocheilidae, …..
Elements of this text belongs in Results and Discussion. All that is needed here is a description of the methodology used.
“The phylogenetic methodology is the same as reported by Simone [13, 23], that basically consists of the matrix mounted in Nexus, analyzed by programs TNT and PAUP.” Is insufficient. Information needs to be added on phylogenetic analyses settings/search criteria etc. It is important that, given the data matrix, the results are reproducible [in as much as phylogenetic tree as estimates of a ‘true phylogeny’ are reproducible] based on the description given in Materials and Methods.
The Simone [13, 23] publications were consulted to determine what phylogenetic methodologies were used in those earlier publications. The description of the methods in Simone [13] are brief to the extreme, do not meet modern standards of reporting, and certainly are not informative about what analyses were actually undertaken for the present study, other than imply a maximum parsimony approach.
The link provided to Simone [23, Bosquejos de filogenia] only takes the reader to a small selection of pages that provide no relevant information. Evidently the full publication is not available for direct access on online, but can be requested.
ANSWER: Ok, fully corrected. Those initial phrases were transferred to the Discussion. The methodology was reported in a more direct manner as demanded.
In respect to the methodology of the phylogenetic analysis, it is really incredibly simple actually. No statistical juggle or maneuver were necessary. The given matrix is simply opened in the informed programs and read with minimum interference. The result is that presently shown, only that. I cannot see anything else to inform beyond what is presently informed or are present in the given references. Both are interesting to be reported: 13 has the technical and practical information (despite in being simple), 23 has the philosophical framework that I follow in phylogenetic analyses. All this given scenario permits the full reproducibility of the presented result, nothing is missing. Of course, if something else needs to be added, please, let me know, I put it quickly.

13. “Nomenclatural acts. The electronic edition of this article conforms …..”
This paragraph has been added by the author, apparent in response to some commentary by earlier reviewers about the merits or validity of erecting taxa in online/digital-only journals. Personally I think this text is unnecessary, but do share the concerns about taxa being erected in the digital-only format. In any event, the Commission has been strongly challenged about this aspect of the Code.
ANSWER: I totally agree, but this paragraph is a journal’s requirement.

14. “Material examined. Types (reported above).”
That is
“Types. Holotype MZSP 152074. Paratypes: MZSP 152252, 2 shells, MZSP 152075, 1 shell from type locality. BRAZIL. Minas Gerais; Unaí (W.V. Matos col, iv/2020), Gueda, 16°16’04”S 46°39’43”W, 647-650 m altitude, MZSP 152236, 1 shell.
Type locality. BRAZIL. Minas Gerais; Unaí, Pedra da Fartura, 16°31’37”S 46°49’18”W, 628- 750 m altitude [Weslley Vailant de Matos col, iv.2020].”
So which specimen(s) were used for the anatomical investigation?
ANSWER: All reported examined material was examined. Those all reported as “specimens” were dissected and studied both, in their anatomy and conchology. Those reported as “shells” were only examined in their conchology. Is necessary to report this in M&M?

Discussion

15. “The taxon descriptions presented above include comparative remarks, which shows the detailed differences amongst the taxa herein studied. This is particularly evidenced in given diagnoses. The present section is, thus, more focused on the most interesting features and those more relevant to taxonomy and present/future phylogenetic approaches to resolving relationships among South American strophocheilids.” Contrary to the author’s statement, the taxon descriptions do not include comparative remarks. Apparently this is a legacy of an earlier version of the ms.

ANSWER: I am sorry. It was rephrased.

16. “…The anatomical features were fundamental to determine both as separate genera.”

‘both as separate genera’ is grammatically lazy. Better would be ‘… to determine these are distinct genera’.

ANSWER: Done.

17. “… Both, A. multicolor and C. uhlei, share short, blunt mesocones, particularly those of the rachidian teeth …”

‘both’ redundant. Delete.

ANSWER: Done.

18. “… The present discussion focuses in the characters, every numerical parameter is based on a N from 10 to 20 specimens and is an integer value. Related to the shell elongation, A. multicolor is the slenderer (width/length W/L index = 46%) (Figs. 1A–C, 25C), with a group orbiting W/L ~50%, such as A. synchondrus (Fig. 25E – 54%), A. albolabiatus (Fig. 25A – 49%), A. miersi (Fig. 25B – 51%) and A turnix (Fig. 25D – 55%); while another group has shells broader, encompassing species with W/L = 60%, such as A. vailanti, A. morenus (Fig. 25G) and A. savanicus (Fig. 25H). In respect to the spire angulation, A. synchondrus has the sharpest pointed spire, ~35°, followed by A. multicolor ~40°, and A. albolabiatus ~42°; A, turnix has the broader spire, with ~58°, followed by A. miersi ~55°; while the remaining species (A. morenus, A. vailanti and A. savanicus) have ~50° …..”

In this paragraph, and to a least extent in subsequent paragraphs, the author has persisted with the same presentation style criticised by reviewers of the earlier version of the ms. Thus this section of the discussion continues to suffer in being too verbose, clumsy presentation of comparative morphometric information, lack of clarity about diagnostic features useful in species discrimination and identification, etc. Suggestions on some alternative formats for presentation were suggested earlier, but have not been taken up. This is the author’s call, but at some detriment to clarity.

ANSWER: It is important to emphasize that the set of characters useful in species discrimination and identification are already in the Diagnoses. Diagnoses has just this function, and are one of the ICZN requirements. I do not see the necessity to repeat those in the Discussion. This item is, I wonder, more concerned to comparations and discussions. They are usually read by a colleague that deals with these or related taxa. There is no way to make the text nicer in a technical report like this. Trying to attend this criticism, I introduced the Tables 1 and 2, that show the more important conchological (Table 1) and anatomical (Table 2) characters in synthetic way. However, the insertion of the table does not eliminate the need of that text.

6. PLOS authors have the option to publish the peer review history of their article (what does this mean?). If published, this will include your full peer review and any attached files.

If you choose "no", your identity will remain anonymous but your review may still be made public.

Do you want your identity to be public for this peer review? For information about this choice, including consent withdrawal, please see our Privacy Policy.

Reviewer #1: No

Reviewer #2: **Yes:** Jonathan Ablett

Reviewer #3: **Yes:** Gary M. Barker

[NOTE: If reviewer comments were submitted as an attachment file, they will be attached to this email and accessible via the submission site. Please log into your account, locate the manuscript record, and check for the action link "View Attachments". If this link does not appear, there are no attachment files.]

While revising your submission, please upload your figure files to the Preflight Analysis and Conversion Engine (PACE) digital diagnostic tool, https://pacev2.apexcovantage.com/. PACE helps ensure that figures meet PLOS requirements. To use PACE, you must first register as a useAnswer: Registration is free. Then, login and navigate to the UPLOAD tab, where you will find detailed instructions on how to use the tool. If you encounter any issues or have any questions when using PACE, please email PLOS at figures@plos.org. Please note that Supporting Information files do not need this step.
