# Supplementary material for: Additions to the genus Anthinus occurring in Minas Gerais and Goiás regions, Brazil, with description of five new species, one of them in the new related genus Catracca (Gastropoda, Eupulmonata, Strophocheilidae)
Source: PLoS One. 2022 Aug 24;17(8):e0273067. doi: 10.1371/journal.pone.0273067 (PMC9401170; doi:10.1371/journal.pone.0273067)
Supplement: Attachment — Submitted filename: ReviewerReport.docx [file pone.0273067.s003.docx]

No page numbering on ms. Main comments refer to sections of text identified by numbers pencilled in the left margin of the annotated ms.

**Abstract**

1. “A bulimulid, a simpulopsid and two dorcasiids are outgroups.”

2. “*Mirinaba* and *Anthinus* also resulted monophyletic (3 and 6 synapomorphies respectively.”

Suggest changing to “*Mirinaba* and *Anthinus* were also supported as monophyletic (3 and 6 synapomorphies respectively).

**Introduction**

3. “The description of the type species is, the remaining descriptions and illustrations, to minimize redundancy, are mostly presented compared to it.”

Sentence ambiguous. Re-work.

4. “The anatomical base of all of them will certainly be useful for future taxonomic revisions and for phylogenetic inferences.”

Suggest modifying as

The anatomical information presented on these new taxa will certainly be useful for future taxonomic revisions and for phylogenetic inferences.

5. “Despite these aspects are part of a large revisional project in such this study is inserted, an initial phylogenetic treatment is provided in this paper, based on the species with anatomy more detailly known in the literature and in the herein studied species. The main intention is not to present a “phylogeny of the Strophocheilidae”, as many more species would be necessary, but to provide a better scenario for a discussion and the base for the new taxa.

Suggest the following changes

~~Despite these aspects are part of a large revisional project in such this study is inserted,~~ An initial phylogenetic treatment is provided in this paper, based on species for which anatomy is reasonably well known in the literature, coupled with new information on the species herein erected. The main intention is not to present a “phylogeny of the Strophocheilidae”, as anatomical investigation many more species would be necessary, but as the basis for erection of the new taxa, and to provide a better scenario for a comparative discussion.

**Materials and methods**

6. “The anatomical drawings are an average of all examined specimens. Color photos were obtained by digital cameras, either hand-held or attached to the dissecting microscope. Shell measurements were obtained with a digital caliper, in a minimum of 10 adult shells. The specimens were dissected by standard techniques [13] under dissecting stereomicroscopes, with the specimen immersed under the fixative. All drawings were obtained with the aid of a camera lucida; initially pencilled, afterwards inked; usually drawings produced for each species include data derived from several specimens. The type and voucher….”

Suggest a slight restructure of paragraph

Color photos were obtained by digital cameras, either hand-held or attached to the dissecting microscope. Shell measurements were obtained with a digital caliper~~,~~ for a minimum of 10 adult shells. The specimens were dissected by standard techniques [13] under dissecting stereomicroscopes, with the specimen immersed under the fixative. All drawings were obtained with the aid of a camera lucida; initially pencilled, afterwards inked; usually drawings produced for each species include data derived from several specimens. Thus, the anatomical drawings are an average (composite) of all examined specimens. The type and voucher ….

There is a potential issue arising with presentation composite illustrations rather than illustrating data gathered from individual specimens. Potentially information is lost on variation (if any) among the examined specimens. As a consequence, if there was variation between the examined type specimen [the name bearing specimen *sensu* Code of Zoological Nomenclature] and other examined specimens [non-name-bearing paratypes, topotypes, etc] then it may be ambiguous as to whether the composite encompasses anatomical information drawn from more than one species, such as can occur if one or more specimens were misidentified as belonging to the species in question. Is ambiguity can only be reduced if there is illustration of anatomical features for all examined specimens, or if the author explicitly states there was insignificant or no variation among these specimens.

Where only non-name-bearing specimens are examined ( e.g. paratypes, topotypes, etc) then there will always be uncertainty as to whether this information also pertains to the species as defined by the name-bearing holotype.

7. “… The collector team works for a company called Femorale [www.femorale.com; http://www.femorale.com/femorale/index.asp]. The places of collection are not protected areas, which do not require special permits. The collections, anyway, are supported by the general/permanent license IBAMA-Sisbio 10560-2, which permits extraction of wildlife samples for scientific purposes. ….”

Suggest the following:

The material studied and described in this work was collected by a team working for Femorale, a private company [www.femorale.com; http://www.femorale.com/femorale/index.asp]. The places of collection are not within protected areas, and as such collection activity did not require special permits. Nonetheless, the collections were made under general/permanent license IBAMA-Sisbio 10560-2, which permits extraction of wildlife samples for scientific purposes.

8. “Thus, details on vegetation, climate, soil, rainfall, etc., were not available, but, when relevant, these data were extracted from the literature or official websites…”

Suggest

Thus, details on vegetation, climate, soil, rainfall, etc., were not available, but, when possible and relevant, these data were extracted from the literature, digital online resources, or official websites.

9. “For comparison of the presently studied species with those already known, the large MZSP collection was consulted, as a previous curator, Dr. José L.M. Leme, was a strophocheilid specialist.”

Suggest

For comparison of the presently studied species with those already known, the large MZSP collection was consulted, including the material assembled by Dr. José L.M. Leme, a previous curator and strophocheilid specialist.

10. “Although additional approaches can be added to any taxonomical paper, such as multivariate statistical analyses of shell measurements, molecular sequencing, etc., the presently presented anatomical investigation was so informative and convincing that it already bases very well the taxonomic conclusions. Of course, other approaches, molecular in particular, will be explored in future papers, considering an already well-based taxa provided by the present paper. It is important to emphasize that most of the land snails taxonomy is based only on their shells alone; the present paper goes further and presents convincing anatomical descriptions.”

This text has been added following adverse comments from previous reviewers, and is the author’s attempt to justify the methodology and pre-empt any future commentary. This text added by the author is unnecessary, redundant, as the published work should stand on its own merits.

11. “The chosen model to do the taxonomic remarks on each described species is reuniting all of them in a single Discussion, which ends this paper. This is interpreted as a more intelligible, fluent, and economic manner to explore the distinctions and similarities of the addressed taxa. Rather than individual remarks after each description.”

This text too was added by the author as a response to comments from previous reviewers. The criticism was not directed as the author’s model to aggregate the comparative remarks in the Discussion, but in the way that aggregation was done – too verbose, clumsy presentation of comparative morphometric information, lack of clarity about diagnostic features useful in species discrimination and identification, ….

I think this text in the main is redundant and should be replaced by a single sentence stating the fact that the taxonomic remarks are aggregated into the Discussion.

12. “Phylogenetic analysis. In a family such as Strophocheilidae, …..

Elements of this text belongs in Results and Discussion. All that is needed here is a description of the methodology used.

“The phylogenetic methodology is the same as reported by Simone [13, 23], that basically consists of the matrix mounted in Nexus, analyzed by programs TNT and PAUP.” Is insufficient. Information needs to be added on phylogenetic analyses settings/search criteria etc. It is important that, given the data matrix, the results are reproducible [in as much as phylogenetic tree as estimates of a ‘true phylogeny’ are reproducible] based on the description given in Materials and Methods.

The Simone [13, 23] publications were consulted to determine what phylogenetic methodologies were used in those earlier publications. The description of the methods in Simone [13] are brief to the extreme, do not meet modern standards of reporting, and certainly are not informative about what analyses were actually undertaken for the present study, other than imply a maximum parsimony approach.

The link provided to Simone [23, Bosquejos de filogenia] only takes the reader to a small selection of pages that provide no relevant information. Evidently the full publication is not available for direct access on online, but can be requested.

13. Simone LRL. 2011. Phylogeny of the Caenogastropoda (Mollusca), based on comparative morphology. Arq Zool. 2011; 42: 161–323.

23. Simone LRL. Bosquejos de filogenia. Clube de Autores. Curitiba, 60 pp. https://www.google.com.br/books/edition/Bosquejos_De_Filogenia/b5TtDwAAQBAJ?hl=pt-BR&gbpv=0

13. “Nomenclatural acts. The electronic edition of this article conforms …..”

This paragraph has been added by the author, apparent in response to some commentary by earlier reviewers about the merits or validity of erecting taxa in online/digital-only journals. Personally I think this text is unnecessary, but do share the concerns about taxa being erected in the digital-only format. In any event, the Commission has been strongly challenged about this aspect of the *Code*.

14. “Material examined. Types (reported above).”

That is

“Types. Holotype MZSP 152074. Paratypes: MZSP 152252, 2 shells, MZSP 152075, 1 shell from type locality. BRAZIL. Minas Gerais; Unaí (W.V. Matos col, iv/2020), Gueda, 16°16’04”S 46°39’43”W, 647-650 m altitude, MZSP 152236, 1 shell.

Type locality. BRAZIL. Minas Gerais; Unaí, Pedra da Fartura, 16°31’37”S 46°49’18”W, 628- 750 m altitude [Weslley Vailant de Matos col, iv.2020].”

So which specimen(s) were used for the anatomical investigation?

Discussion

15. “The taxon descriptions presented above include comparative remarks, which shows the detailed differences amongst the taxa herein studied. This is particularly evidenced in given diagnoses. The present section is, thus, more focused on the most interesting features and those more relevant to taxonomy and present/future phylogenetic approaches to resolving relationships among South American strophocheilids.”

Contrary to the author’s statement, the taxon descriptions do not include comparative remarks. Apparently this is a legacy of an earlier version of the ms.

16. “…The anatomical features were fundamental to determine both as separate genera.”

‘both as separate genera’ is grammatically lazy. Better would be ‘… to determine these are distinct genera’.

17. “… Both, *A. multicolor* and *C. uhlei*, share short, blunt mesocones, particularly those of the rachidian teeth …”

‘both’ redundant. Delete.

18. “… The present discussion focuses in the characters, every numerical parameter is based on a N from 10 to 20 specimens and is an integer value. Related to the shell elongation, *A. multicolor* is the slenderer (width/length W/L index = 46%) (Figs. 1A–C, 25C), with a group orbiting W/L ~50%, such as *A. synchondrus* (Fig. 25E – 54%), *A. albolabiatus* (Fig. 25A – 49%), *A. miersi* (Fig. 25B – 51%) and *A turnix* (Fig. 25D – 55%); while another group has shells broader, encompassing species with W/L = 60%, such as *A. vailanti*, *A. morenus* (Fig. 25G) and *A. savanicus* (Fig. 25H). In respect to the spire angulation, *A. synchondrus* has the sharpest pointed spire, ~35°, followed by *A. multicolor* ~40°, and *A. albolabiatus* ~42°; *A, turnix* has the broader spire, with ~58°, followed by *A. miersi* ~55°; while the remaining species (*A. morenus*, *A. vailanti* and *A. savanicus*) have ~50° …..”

In this paragraph, and to a least extent in subsequent paragraphs, the author has persisted with the same presentation style criticised by reviewers of the earlier version of the ms. Thus this section of the discussion continues to suffer in being too verbose, clumsy presentation of comparative morphometric information, lack of clarity about diagnostic features useful in species discrimination and identification, etc. Suggestions on some alternative formats for presentation were suggested earlier, but have not been taken up. This is the author’s call, but at some detriment to clarity.
