# Supplementary material for: Additions to the genus Anthinus occurring in Minas Gerais and Goiás regions, Brazil, with description of five new species, one of them in the new related genus Catracca (Gastropoda, Eupulmonata, Strophocheilidae)
Source: PLoS One. 2022 Aug 24;17(8):e0273067. doi: 10.1371/journal.pone.0273067 (PMC9401170; doi:10.1371/journal.pone.0273067)
Supplement: Attachment — Submitted filename: Response to Reviewers 07-2022.docx [file pone.0273067.s007.docx]

I thank to the editorial council and to the three referees for this time relatively positive analysis on my paper on *Anthinus* and *Catracca* from Brazil.

This new round of comments and criticisms were very pertinent and useful for further improving the paper. They are all fully accepted, and the new version that is now re-submitted has all of them integrally corrected.

I answer below only the more relevant questions IN RED, as all remaining ones were simply corrected, including those that were written directly on the manuscript.

Reviewer #1: However, I do insist to prepare an additional table with number of individuals studied for each of the species. The data are lost in the text. Please, in the tables you prepared (on shell and anatomical characteristics) add an additional column containing the data on how many (precisely) individuals you used for the measurements! The expression “several specimens” is not acceptable! I would prefer to have some data on the variation (e.g. SD) within the individuals you did collect and used for your study.

ANSWER: A Table was inserted in the “Material and methods” item as the referee demanded. It is the Table 1 now. This required a renumber of the remaining tables, being now Tables 2 and 3 in the new manuscript (MS). I also corrected the indicated remaining points on the new MS.

Reviewer #2: I think the manuscript is much improved in this revision. It is clearer and more concise.
I think the inclusion of table 1 and 2 are a great addition and make the differential diagnoses between species much easier to understand. However I think in places the discussion is overly long and could be condensed especially where points are repeated.

ANSWER: some parts of the text were removed as the referee demanded in order to condense it. However, that discussion is not a mere compilation of what is written in the table. It was also used to put the species’ distinctions into grades, ranks, which in a future will be useful in comparative and phylogenetic scenarios. Thus, the cut of the text was not too much. I hope the text can be maintained this way.

I hope the author and editor do not mind but I have made some suggestions to the grammar/sentence structure to improve the English in places (which is in general is of a high standard). There are a few areas where I am not sure what the author quite means and I have noted these for clarification.
ANSWER: Everything corrected.

Reviewer #3: Several minor editorial corrections and changes to text suggested in the returned annotated manuscript.

ANSWER: Everything corrected
